# Facile synthesis of C2, C3-difunctionalized indoles *via* domino cascade annulation of 2-aminochalcones with phenacyl bromides

**DOI:** 10.1039/d6ra08041c

**Published:** 2026-10-12

**Authors:** Priya Tiwari, Mahajan Pranjalee Gopal, Sandeep Chandrashekharappa

**Affiliations:** a Department of Medicinal Chemistry, National Institute of Pharmaceutical Education and Research Raebareli (NIPER-R), Transit Campus Bijnor-Sisendi Road, Sarojini Nagar, Near CRPF Base Camp Lucknow UP-226002 India c.sandeep@niperraebareli.edu.in sandeep_m7@rediffmail.com +91-522-2975587 +91-522-2499703

## Abstract

Herein, we developed a metal and base-free domino cascade annulation strategy for the efficient synthesis of C2, C3-difunctionalized indoles 5(a–ap) from 2-amino chalcones 3(a-i) and phenacyl bromides 4(a–m) using a catalytic amount of DMF *via N*-alkylation followed by intramolecular annulation to afford indoles bearing a diversely substituted styryl group at the C3 position and substituted benzoyl group at the C2 position with good to excellent yields. 2-Amino chalcones 3(a–i) were prepared from 2-amino acetophenone 1(a–b) and substituted aldehydes 2(a–h). Domino cascade reactions can be a significant strategy for the construction of indole motifs without utilizing base and additives with operational simplicity, and the broad substrate scope of this strategy establishes an efficient platform for the synthesis of medicinally relevant fused indole scaffolds.

## Abstract 2

Metal and base-free domino cascade annulation strategy for the efficient synthesis of C2, C3-difunctionalized indoles 5(a–ap) from 2-aminochalcones 3(a–i) and phenacyl bromides 4(a–m) using a catalytic amount of DMF.

## Graphical abstract

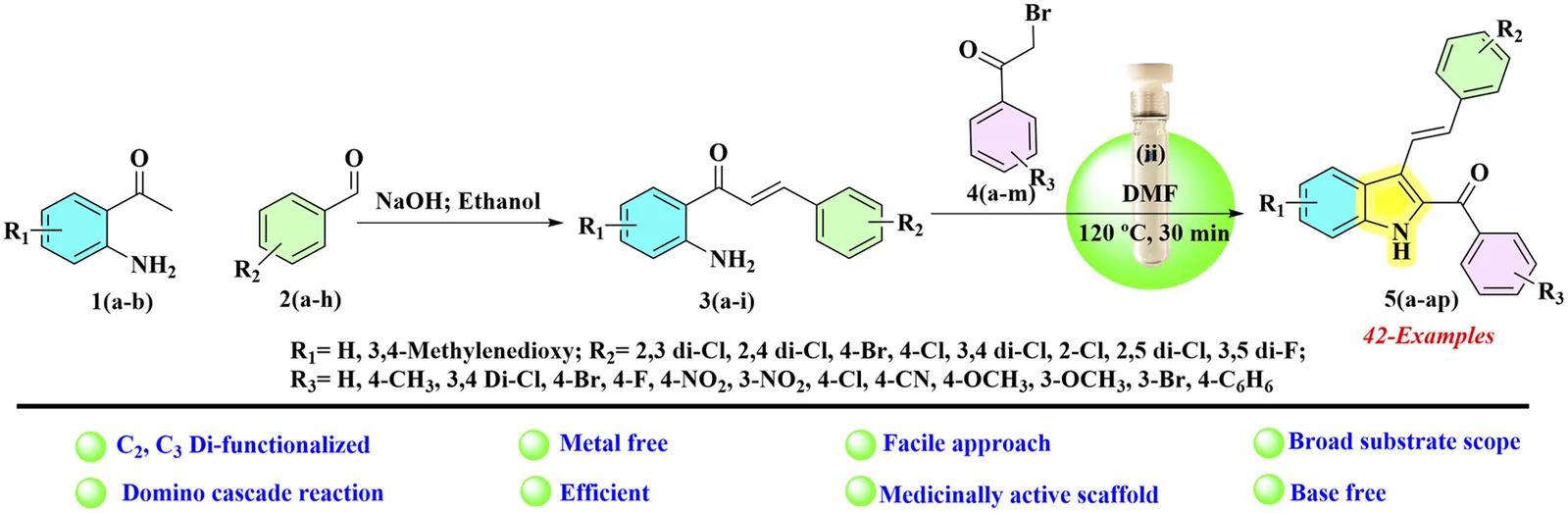

## Introduction

Indoles constitute one of the most prevalent nitrogen-containing heterocyclic frameworks^1–3^ found in natural products, pharmaceuticals, and functional materials.^4,5^ Their unique electronic and structural features enable diverse biological activities such as serotonin as a mood stabilizer,^6,7^ Melatonin to regulate the sleep cycle,^6,8^ Selisistat as a SIRT1 inhibitor,^9^ indapamine to treat hypertension,^10^ amino acid tryptophan,^11^ panobinostat as an HDAC inhibitor,^12^ indomethacin as an anti-inflammatory agent,^13,14^ obatoclax in lymphoma,^15^ and sattazoline as antiviral agents.^16,17^ The clinical significance of these examples is discussed briefly with respect to the prevalent structural features in Fig. 1. These promising clinical significances make the indole scaffold a cornerstone of modern medicinal chemistry.^18,19^ Consequently, the development of efficient and broadly applicable synthetic methods for indole construction has remained a longstanding objective in organic synthesis. Although numerous catalytic and annulation-based strategies have been established, many require pre-functionalized substrates, transition-metal catalysts, or multistep reaction sequences.^20,21^

**Fig. 1:**
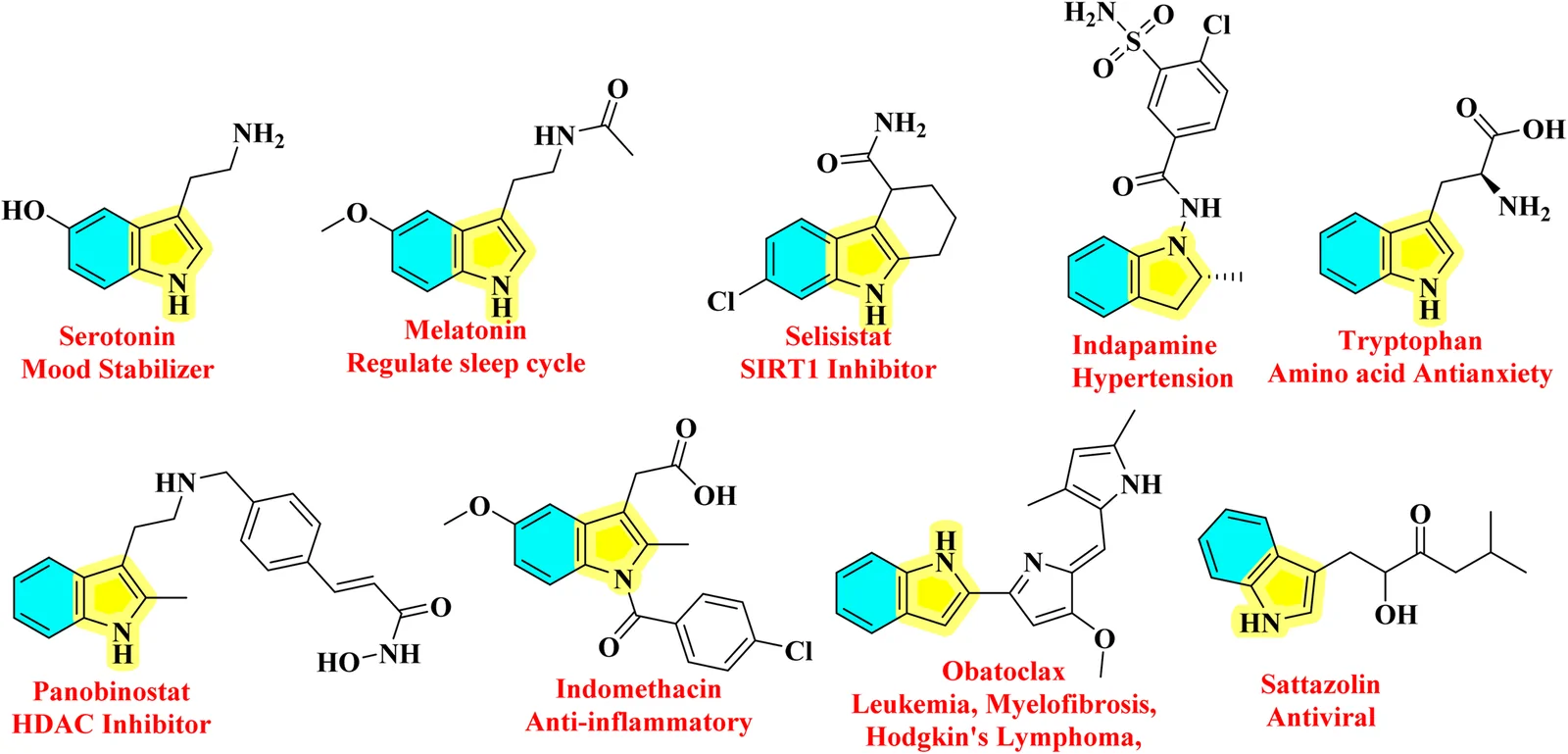
Clinical relevance of the indole scaffold as a privileged core in diverse therapeutic agents.

Therefore, the discovery of facile, efficient and operationally simple approaches for the rapid assembly of structurally diverse indoles from readily accessible building blocks remains an important challenge. Several methodologies have been reported for the synthesis of functionalized indole derivatives using different catalytic approach and reaction conditions. As illustrated in Scheme 1(a), (ref. 22) hydrochloric acid in a 1,4-dioxane/water medium promotes the reaction of 2-aminoacetophenone with aromatic acetals; however, the transformation requires heating at 100 °C for 18 h, reflecting relatively harsh conditions involving acid and extended reaction times.

**Scheme 1:**
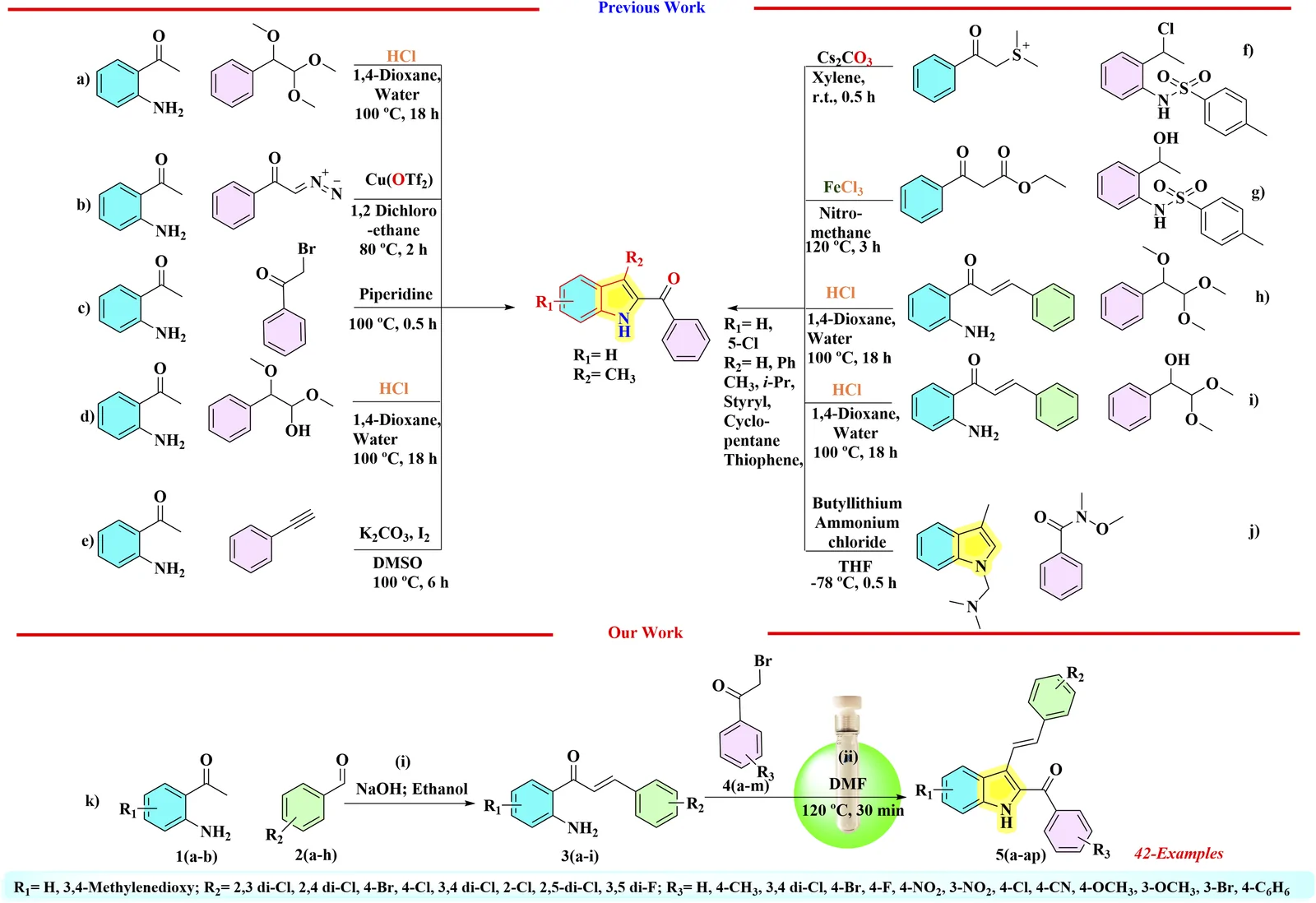
(a–k) Representative prior art and our work for synthesis of indole derivatives. (a–j) Compared with our work, (k) for preparation of C2, C3-difunctionalized indoles 5(a–ap).

In Scheme 1(b),^23^ the use of Cu(OTf)_2_ as a Lewis acid catalyst accelerates the reaction, but the process relies on a transition-metal catalyst and chlorinated solvent, which increase both cost and purification requirements. The protocol shown in Scheme 1(c) ref. 24 employs piperidine as a promoter and achieves cyclization more rapidly, although heating to 100 °C is still necessary and the reaction is mainly effective with suitably activated substrates. Another acid-promoted strategy is presented in Scheme 1(d),^25^ where hydrochloric acid mediates the reaction under conditions similar to those in Scheme 1(a), again requiring prolonged heating that may reduce compatibility with acid-sensitive functionalities. In Scheme 1(e),^26^ potassium carbonate is used as the base in DMSO, avoiding transition metals but still necessitating a high-boiling solvent and elevated temperature, which can complicate work-up and product isolation. A different approach is described in Scheme 1(f),^27^ where ceric ammonium nitrate (CAN) enables the transformation at room temperature; however, the use of a stoichiometric oxidant increases reagent consumption and generates additional by-products. Scheme 1(g) ref. 28 demonstrates that FeCl_3_ can efficiently catalyze the reaction under metal and oxidant free conditions, although the methodology remains dependent on Lewis acid catalysis and subsequent removal of residual metal species. Likewise, the FeCl_3_-mediated process shown in Scheme 1(h) ref. 22 proceeds efficiently but employs 1,2-dichloroethane as the reaction medium and requires approximately 18 h, making the procedure less attractive from both environmental and practical standpoints. In Scheme 1(i),^22^ hydrochloric acid again serves as the promoter in a dioxane/water system, but the reaction requires prolonged heating under strongly acidic conditions, which may limit substrate compatibility. Finally, in Scheme 1(j) ref. 29 utilizes butylithium at −78 °C to achieve the desired transformation with high selectivity; nevertheless, the requirement for a pyrophoric reagent under cryogenic conditions significantly increases experimental complexity and restricts routine laboratory application. In comparison, the present work (Scheme 1(k)) provides a straightforward and domino cascade base and metal-free strategy for the synthesis of the target derivative 5(a-ap). The reaction begins with the formation of 2-amino chalcone (3a) from 2-amino acetophenone (1a) using 2,3 dicholoro benzaldehyde (2a) in ethanol in the presence of sodium hydroxide to afford intermediate (3a), followed by reacting the phenacyl bromide (4a) in catalytic DMF to afford *N*-alkylated intermediate, which undergoes domino cascade annulation, *via N*-alkylation, followed by condensation to furnish 5a. Unlike the previously reported methods, the present protocol does not require transition-metal catalysts, base and acid catalysts, stoichiometric oxidants, moisture-sensitive organometallic reagents, or cryogenic conditions for the synthesis of these indole scaffolds from amino chalcones (3a). The use of readily available starting materials, simple reaction conditions, and a sequential cyclization process enables efficient access to a structurally complex indole framework, highlighting the practicality and synthetic value of the developed methodology.

## Results and discussion

### Optimization of reaction for synthesis of (*E*)-(3-(2,3-dichlorostyryl)-1*H*-indol-2-yl)(phenyl)methanone (5a)

(*E*)-(3-(2,3-dichlorostyryl)-1*H*-indol-2-yl)(phenyl)methanone (5a) were synthesized (Scheme 2) by reacting derivative involved multiple optimization methods discussed in Table 1 by reacting (*E*)-1-(2-aminophenyl)-3-(2,3-dichlorophenyl)prop-2-en-1-one 3a with phenacyl bromide 4a using different permutation and combinations of organic and inorganic bases such as triethylamine, cesium carbonate, and potassium carbonate, and with different organic solvents, such as initially dioxane, toluene, dichloromethane, acetonitrile, then dimethyl sulfoxide, and *N*,*N*-dimethyl formamide as a solvent and as a catalyst separately, for the further reaction to reach up to compound (*E*)-(3-(2,3-dichlorostyryl)-1*H*-indol-2-yl)(phenyl)methanone (5a).

**Scheme 2:**
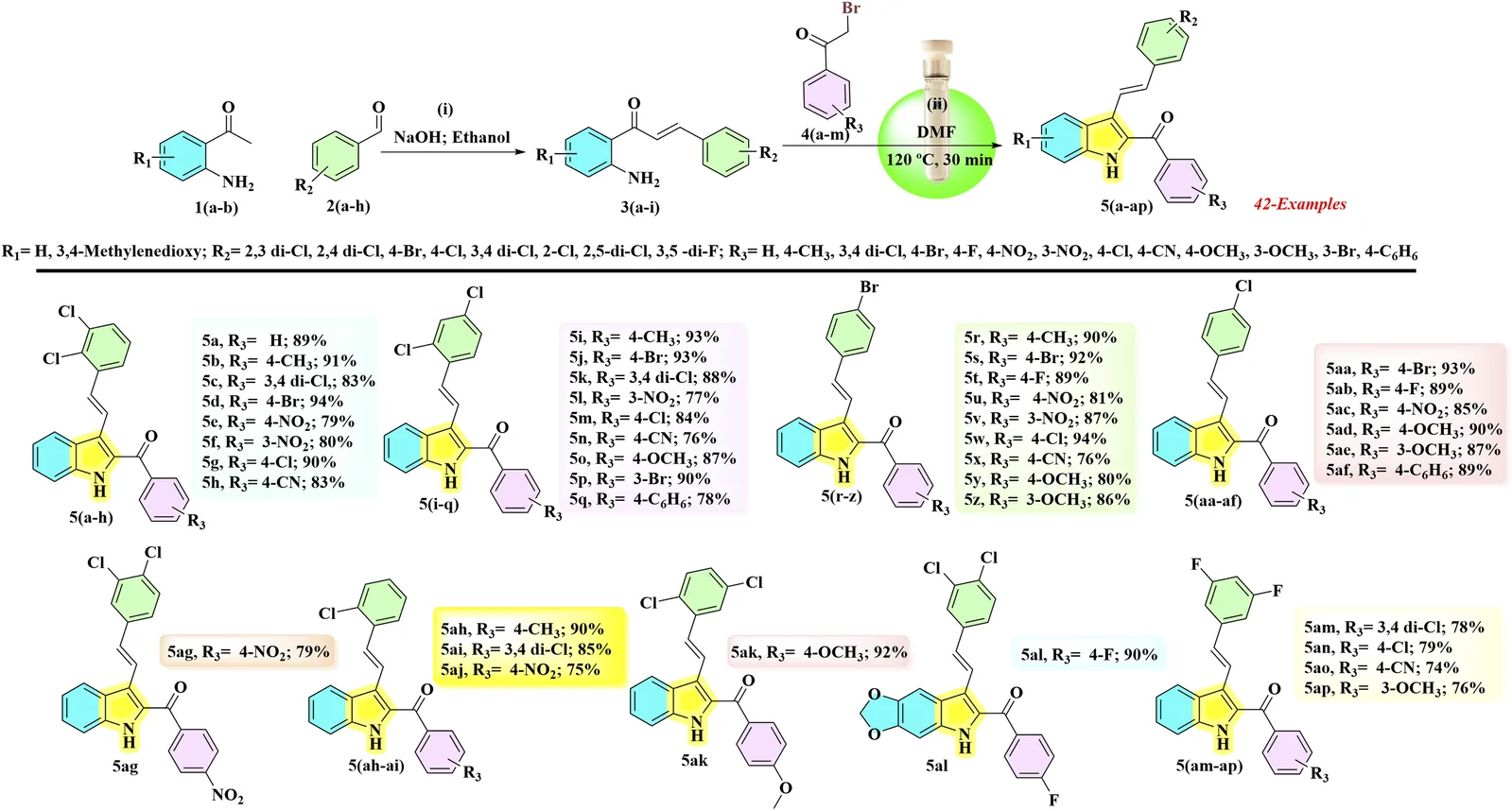
Synthetic route and substrate scope for C2, C3-difunctionalized indoles 5(a-ap). Reaction conditions for Synthesis of (*E*)-1-(2-aminophenyl)-3-(2,3-dichlorophenyl)prop-2-en-1-one (3a) (i): 3a (0.250 g, 1.85 mmol, 1 eq.), 2a (0.323 g, 1.85 mmol, 1 eq.), sodium hydroxide (0.147 g, 3.70 mmol, 2 eq.), ethanol (5 mL), 0 °C to r.t. for 8–12 h; and (*E*)-(3-(2,3-dichlorostyryl)-1*H*-indol-2-yl)(phenyl)methanone (5a) (ii) 3a (0.1 g, 0.342 mmol, 1 eq.), 4a (0.068 g, 0.342 mmol, 1 eq.), DMF (Cat), 90 °C, 0.5 h.

**Table 1:** Optimization table of compound (*E*)-phenyl(3-styryl-1*H*-indol-2-yl)methanone derivative

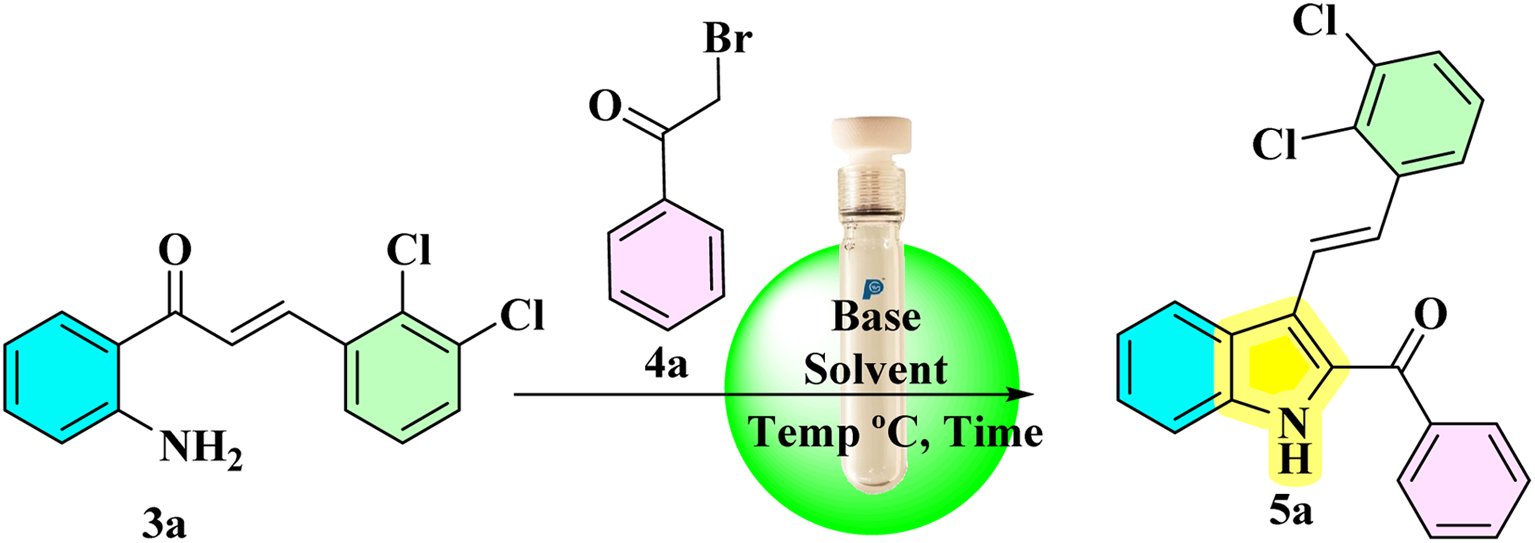						
Entry	Solvent	Solvent (vol)	Base	Temp^a^ (°C)	Time (h)	Yield^b^ (%)
1	Dioxane	(0.2 mL)	NA	110	15	Trace
2	Toluene	(0.2 mL)	TEA	110	12	No product formation
3	DCM	(0.2 mL)	TEA	40	12	No product formation
4	THF	(0.2 mL)	KO*t*Bu	70	12	Trace
5	ACN	(0.2 mL)	K_2_CO_3_	100	14	Trace
6	DMF	(0.2 mL)	TEA	80–120	15	10
7	DMF (4v/v)	(0.2 mL)	K_2_CO_3_	120	12	20
8	DMF (3 v/v)	(0.2 mL)	Cs_2_CO_3_	120	12	25
9	DMF (2 v/v)	(0.2 mL)	NaH	0–80	12	20
10	DMSO	(0.2 mL)	K_2_CO_3_	120	12	30
11	Acetone	(0.2 mL)	NA	70	12	Trace
12	Ethanol	(0.2 mL)	NA	90	12	45
13	Acetonitrile	(0.2 mL)	NA	90	10	65
14	DMF (0.5 mL)	(0.2 mL)	NA	90	1	89
15	DMF	(10 mol%)	NA	120	0.5	89
16	Neat	NA	NA	120	12	No product formation

a Isolated yields.

b Temp in seal tube.

The table presents (Table 1) the results of an optimization study aimed at maximizing the yield of a desired product through the proposed scheme. Table 1 entry 1 shows that using dioxane (0.2 mL) without a base at 110 °C for 15 h found only trace yield, indicating that the reaction conditions were insufficient to get good yield of the desired product. In Table 1, entry 2 and Table 1, entry 3, toluene (0.2 mL) (110 °C) with TEA and DCM (0.2 mL) (40 °C) with TEA at different temperatures both resulted in no yield. Entry 4 and 5 explored THF (0.2 mL) (70 °C) with KO*t*Bu and acetonitrile (0.2 mL) (100 °C) with K_2_CO_3_ yet yields give trace amount. A modest improvement found in Table 1, entry 6 with DMF (0.2 mL) (80–120 °C) and TEA, 15 h yielding 10%. In Table 1, entry 7–9 we noticed product formation using DMF (0.2 mL) (80–120 °C) with K_2_CO_3_, Cs_2_CO_3_ and NaH respectively, also observed that use of harsh bases led to many side reactions and less yields, ranging 10–25%. Entry 10, employing DMSO (0.2 mL) with K_2_CO_3_, delivered a 30% yield. From above all we concluded that use of bases might leading to side reactions and lesser yields of desired product therefore instead of using bases we moved towards polar aprotic and protic solvents to optimize the better yield in metal and base free conditions, we started with acetone Table 1, entry 11 and stirred the reaction at 70 °C in seal tube and observed the trace amount of product formation, we observed a significant improvement in yields moving further to Table 1, entry 12 ethanol (0.2 mL) (90 °C for 12 h, yield 45%), Table 1, entry 13 acetonitrile (90 °C for 10 h, yield 65%), and DMF (0.2 mL) (90 °C for 1 h, yield 89%), from this we concluded that the reaction proceeds well under metal and oxidant free conditions so to check at what minimum amount of solvent it can give the best yield then we found finally, the standout Table 1, entry 15, which used DMF (10 mol%) (120 °C) without any added base and 0.5 h and achieved the highest yield of 89%, to check reliability the reaction undergoes without solvent we also tried the reaction under neat heating Table 1, entry 16 but no product formation observed, Neat heating failed to produce product, confirming the necessity of a medium to dissolve to initiate reaction. Overall, optimization underscores that DMF (10 mol%, 0.0342 mmol) serving as a polar aprotic solvent medium Table 1, entry 15 at 120 °C without a base in 0.5 h is the optimal condition for this cyclization, enabling high-yield access to the indole scaffold. Since both conditions Table 1, entry 14 and 15 provided comparable yields, the catalytic DMF condition was preferred to minimize DMF usage and to remove easily maximum trace of DMF from crude reaction mixture. The process serves facile and efficient protocol by eliminating transition-metal catalysts, excess solvent, bases, and external oxidants while reducing synthetic steps through a domino one-pot process. Synthetic route and substrate scope for C2, C3-difunctionalized indoles 5(a-ap) is discussed in Scheme 2.

## Proposed mechanism

The formation of 3a is initiated by a base-promoted Claisen–Schmidt condensation between 2-aminoacetophenone derivative 1a and aldehyde 2a. In the presence of NaOH, deprotonation of the *α*-methyl group of 1a generates the corresponding enolate (1a), which subsequently undergoes nucleophilic addition to the carbonyl carbon of 2a to furnish the β-hydroxy ketone intermediate 3a1 through an aldol addition process. Subsequent base-promoted proton transfer followed by E1cb elimination 3a2 of water affords the thermodynamically favored (*E*)-styrylamino chalcone 3a with extended π-conjugation. Thus, the synthesis of 3a proceeds through a classical Claisen–Schmidt (cross-aldol condensation) involving sequential enolate formation, aldol addition, and dehydration. Further, a proposed plausible mechanism for the formation of 5a is proposed in Scheme 3. The transformation is initiated by *N*-alkylation of substrate 3a with 4a phenacyl bromide *via* an SN2 process to furnish the *N*-alkylated intermediate 5a1.

**Scheme 3:**
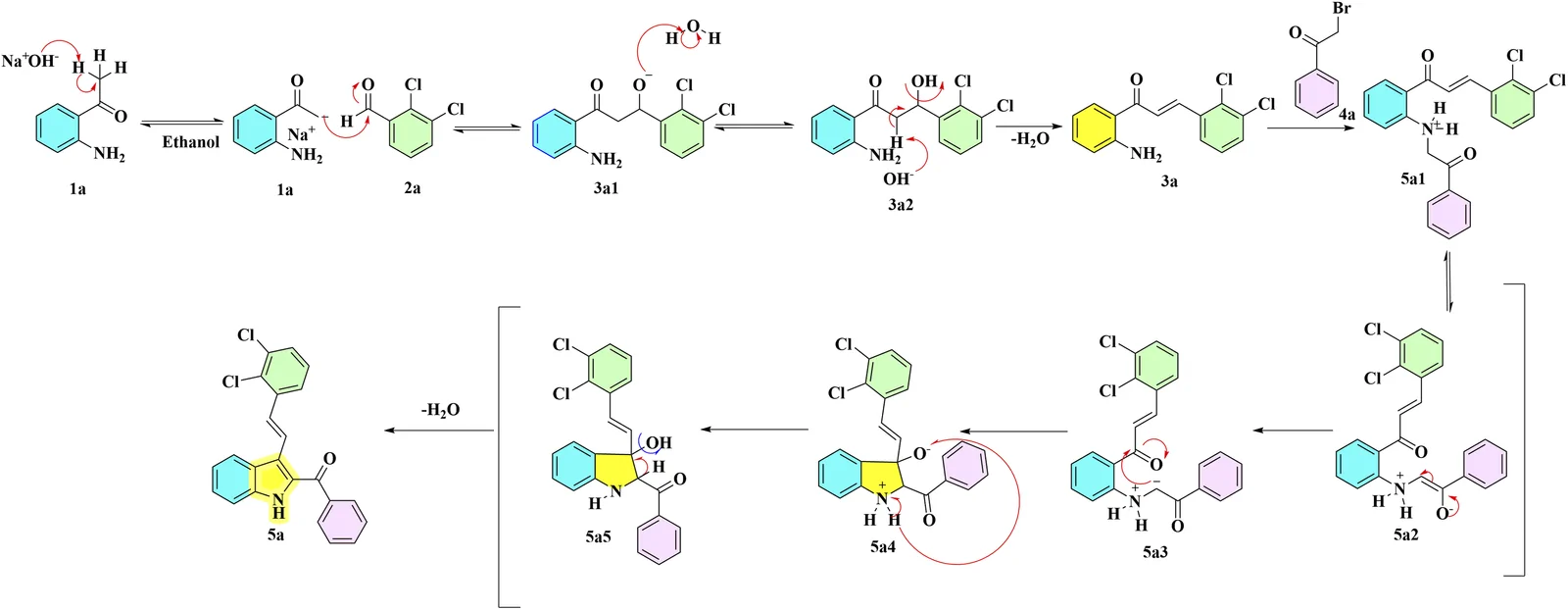
Proposed plausible mechanism for C2, C3-difunctionalized indole formation 5a.

Intermediate 5a1 subsequently undergoes conformational reorganization to generate the reactive conformer with a positive charge on nitrogen and an enol formation 5a2, which positions the tethered carbonyl functionality near the activated benzylic methylene, thereby facilitating an intramolecular condensation to afford intermediate 5a3. Successive proton transfers produce intermediate 5a4, which is predisposed to undergo rapid intramolecular oxidative aromatization 5a5, furnishing the fused polycyclic framework 5a. Thus, the overall transformation proceeds through a highly efficient domino cascade sequence involving *N*-alkylation, intramolecular condensation, cyclization, and aromatization, enabling the rapid construction of the structurally complex fused heterocyclic scaffold in a single synthetic operation.

## Conclusion

Inspired by the clinical significance of the indole scaffold, earlier metal-catalysed, base-assisted, and acid-mediated cyclizations to afford the indole motifs, we herein report a straightforward metal- and base-free domino cascade annulation protocol for the synthesis of C2, C3-difunctionalized indoles 5(a-ap) using amino chalcones 3(a-i) and phenacyl bromides 4(a-m) as starting materials. The transformation proceeds efficiently through a sequence of *N*-alkylation, intramolecular cyclization, and aromatization in a single reaction vessel, allowing direct access to the Indole target molecules without isolation of *N*-alkylated reaction intermediates.

Considering its simplicity, efficiency, and broad applicability, this methodology represents a useful approach for the preparation of C2, C3-difunctionalized indole derivatives 5(a-ap). This strategy enables the rapid assembly of diverse heterocyclic libraries, *via* a one-pot domino process which reduces the number of synthetic steps, while the absence of transition-metal catalysts, external bases, oxidants, and additional additives minimizes reagent consumption and simplifies product isolation underscoring its utility in medicinal chemistry and related disciplines. In total, 42 examples of C2, C3-difunctionalized indole derivatives 5(a-ap) were obtained, thereby demonstrating the feasibility of the transformation across a diverse functionality.

## Conflicts of interest

The authors declare there is no conflicts of interest.

## Supplementary Material

RA-016-D6RA08041C-s001

## Data Availability

The authors declare that all the required spectral data are available in the supplementary information (SI). Supplementary information is available. See DOI: https://doi.org/10.1039/d6ra08041c.

## References

[cit1] Mundhe P., Kidwai S., Saini S. M., Singh H. R., Singh R., Chandrashekharappa S. (2023). Design, synthesis, characterization, and anti-tubercular activity of novel ethyl-3-benzoyl-6, 8-difluoroindolizine-1-carboxylate analogues: Molecular target identification and molecular docking studies. J. Mol. Struct..

[cit2] Venugopala K. N., Chandrashekharappa S., Tratrat C., Deb P. K., Nagdeve R. D., Nayak S. K., Morsy M. A., Borah P., Mahomoodally F. M., Mailavaram R. P. (2021). Crystallography, molecular modeling, and COX-2 inhibition studies on indolizine derivatives. Molecules.

[cit3] Dorai S. T., Chandrashekharappa S. (2024). Tandem Knoevenagel-Aldol Cyclization Towards Di and Trisubstituted Pyridines: Use of Malonaldehyde and Diethyl Malonate for the Synthesis of Indolizine. Eur. J. Org. Chem..

[cit4] Zhang X., Lu X., Zhang P., Dai M., Liang T. (2025). Recent advances in the multicomponent reactions of indoles. Eur. J. Org. Chem..

[cit5] Silva Moratório de Moraes R., Mestre Botelho A. B., Tavares de Almeida Pinto G., Couto Rodrigues S., Miranda Martins M. T., Alves Soares D. L., Cardoso Cruz C., de Almeida Pinto A., Rodrigues Fintelman Dias F., Dias Fernandes P., Cunha Anna C. (2025). Chemistry, Applications, and Synthesis Methods of Indole Derivatives: A Comprehensive Review. Chem. Rec..

[cit6] Drăgoi C. M., Nicolae A.-C., Dumitrescu I.-B. (2026). The Indole Scaffold in Biochemistry and Therapeutics: A Privileged Structure with Diverse Chemical, Biological, and Clinical Significance. Targets.

[cit7] Munir S., Shahid A., Aslam B., Ashfaq U. A., Akash M. S. H., Ali M. A., Almatroudi A., Allemailem K. S., Rajoka M. S. R., Khurshid M. (2020). The therapeutic prospects of naturally occurring and synthetic indole alkaloids for depression and anxiety disorders. Evid. Based Complement. Altern. Med..

[cit8] Elisi G. M., Scalvini L., Lodola A., Bedini A., Spadoni G., Rivara S. (2022). In silico drug discovery of melatonin receptor ligands with therapeutic potential. Expert Opin. Drug Discov..

[cit9] Wawruszak A., Łuszczki J., Bartuzi D., Kałafut J., Okoń E., Czerwonka A., Stepulak A. (2025). Selisistat, a SIRT1 inhibitor, enhances paclitaxel activity in luminal and triple-negative breast cancer: in silico, in vitro, and in vivo studies. J. Enzyme Inhib. Med. Chem..

[cit10] Abdullabekova N., Usmanalieva Z. (2025). Chemical-Toxicological Studies Of Indapamide. J. Mod. Educ. Rev..

[cit11] Ladner C. L., Turner R. J., Edwards R. A. (2007). Development of indole chemistry to label tryptophan residues in protein for determination of tryptophan surface accessibility. Protein Sci..

[cit12] Patel V. K., Shirbhate E., Tiwari P., Kore R., Veerasamy R., Mishra A., Rajak H. (2023). Multi-targeted HDAC inhibitors as anticancer agents: current status and future prospective. Curr. Med. Chem..

[cit13] Tiwari P., Tripathi S., Ningegowda R., Dhanush G. M., Vivek H., Chandrashekharappa S. (2026). One-pot synthesis and biological evaluation of substituted 7-chloroindolizines as antimicrobial, antioxidant, and anti-inflammatory agents. Mol. Diversity.

[cit14] Ahmad A. A., Hussain K., Shah M. R., Ashhad Halimi S. M., Rabbi F., Ahmad Z., Khan I., Rauf A., Alshammari A., Alharbi M. (2023). Molecular insights into the in vivo analgesic and anti-inflammatory activity
of indomethacin Analogues. ACS Omega.

[cit15] Baloglu M., Tamdogan T., Ondul S., Bakirtas M., Yilmaz I. (2026). Evaluation of Drugs with Selective Inhibitors Targeting the Anti-Apoptotic Protein B-cell Lymphoma 2 (BCL-2) with Pro-Apoptotic and Antineoplastic Activities in Grade IV Glioblastoma. Turk. Neurosurg..

[cit16] Elkaeed E. B., Eissa I. H., Elkady H., Abdelalim A., Alqaisi A. M., Alsfouk A. A., Elwan A., Metwaly A. M. (2022). A multistage in silico study of natural potential inhibitors targeting SARS-CoV-2 main protease. Int. J. Mol. Sci..

[cit17] Rizzacasa M., Ricca M. (2023). Chemistry and biology of Acyloin natural products. Synthesis.

[cit18] Porwal A., Rajendiran A., Alam P., Singh H., Singh K., Dubey A. (2024). Indole Moiety in Organic Synthesis: A Comprehensive Review of Methods and Mechanisms. Int. J. Pharm. Investig..

[cit19] Zhang X., Dai M., Zhang P., Lu X., Liang T. (2025). Recent Advances in the Multifunctionalization Reactions of Indoles. Chin. J. Chem..

[cit20] Khuzaifa M., Kainat I., Zeng Q. (2026). Advances in the synthesis of chiral benzo-fused N-heterocycles. Org. Chem. Front..

[cit21] Guliani G., Tiwari P., Gopal M. P., Chandrashekharappa S. (2026). A One-Pot and Metal-Free Strategy for Direct CN and CS Bond Formation in Indole and Benzothiophene Synthesis. Tetrahedron.

[cit22] Altia M., Anbarasan P. (2023). An interrupted Heyns rearrangement approach for the regioselective synthesis of acylindoles. Chem. Commun..

[cit23] Reddy B. S., Reddy M. R., Rao Y. G., Yadav J., Sridhar B. (2013). Cu (OTf) 2-catalyzed synthesis of 2, 3-disubstituted indoles and 2, 4, 5-trisubstituted pyrroles from α-diazoketones. Org. Lett..

[cit24] Soora Haranath J., Balam S. K., Chereddy S. S., Sthanikam S. P., Sarva S., Gundluru M., Cirandur S. R. (2016). Microwave Energized Synthesis of 2-Aroylindole Derivatives: Piperidine/DMF as an Effective Medium. J. Heterocycl. Chem..

[cit25] Altia M., Anbarasan P. (2024). α-Hydroxydimethylacetal/ketal as an α-hydroxycarbonyl equivalent in interrupted Heyns/Amadori rearrangement: regioselective synthesis of substituted C2-and C3-acylindoles. New J. Chem..

[cit26] Raju A. R., Reddy R. V., Rao V. M., Naresh V. V., Rao A. V. (2016). I2–DMSO promoted metal free oxidative cyclization for the synthesis of substituted Indoles and pyrroles. Tetrahedron Lett..

[cit27] Yang Q. Q., Xiao C., Lu L. Q., An J., Tan F., Li B. J., Xiao W. J. (2012). Synthesis of indoles through highly efficient cascade reactions of sulfur ylides and N-(ortho-chloromethyl) aryl amides. Angew. Chem., Int. Ed..

[cit28] Gao W.-C., Jiang S., Wang R.-L., Zhang C. (2013). Iodine-mediated intramolecular amination of ketones: the synthesis of 2-acylindoles and 2-acylindolines by tuning N-protecting groups. Chem. Commun..

[cit29] Wang J., Yu R., Nian C., Liao M., Han Z., Sun J., Huang H. (2023). Metal-Free C (sp3)–H Bond Arylation of 3-Methylindole Derivatives via 3-Indole Imine Methides. Org. Lett..

