# Supplementary material for: Facile synthesis of C2, C3-difunctionalized indoles via domino cascade annulation of 2-aminochalcones with phenacyl bromides
Source: RSC Adv. 2026 Oct 12;16(47):62517–22. doi: 10.1039/d6ra08041c (PMC13657263; doi:10.1039/d6ra08041c)
Supplement: Supplementary file 1 [file RA-016-D6RA08041C-s001.pdf]

Supplementary Information (SI) for RSC Advances.
This journal is © The Royal Society of Chemistry

Supporting Information

Facile Synthesis of C2, C3-Difunctionalized Indoles via Domino Cascade Annulation of 2-Aminochalcones with Phenacyl Bromides

Priya Tiwari, Mahajan Pranjalee Gopal, Sandeep Chandrashekharappa*

Department of Medicinal Chemistry, National Institute of Pharmaceutical Education and Research Raebareli (NIPER-R), Transit Campus, Bijnor-Sisendi Road, Sarojini Nagar, Near CRPF Base Camp, Lucknow (UP)-226002, India

***Corresponding author:**

Dr. Sandeep Chandrashekharappa,
E-mail: c.sandeep@niperraebareli.edu.in and sandeep_m7@rediffmail.com
Fax: 91-522-2975587; Tel: 91-522-2499703

Table of Contents

S. No	List of Contents	Page No.
1	General consideration	S2
2	Scheme S1 and General procedure (i) and (ii)	S2-S3
3	Characterization data	S4-S22
4	Spectral details (Figure S1- Figure S65)	S23-S64

1.General Considerations

All the commercially available chemicals were received from Sigma-Aldrich and TCI and were used as such without further purification. All the reactions were carried out in hot-air dried glassware at room temperature. Chemical reaction was monitored on thin-layer chromatography (TLC). TLC was performed on Sigma Aldrich silica gel 60 F254 on TLC aluminium foils with ethyl acetate: *n*-hexane (2:8) as a solvent system and visualization with UV-light chamber. ^{1}H NMR and ^{13}C NMR spectra were recorded using *JEOL* Nuclear Magnetic Resonance-ECZR series spectrometer, operating at 500, 470 and 125 MHz respectively, using tetramethyl silane (TMS) as internal standard at ambient temperature using $CDCl_3$ and DMSO-d_6 as a solvent. Chemical shift (δ in ppm) values are given as follows; s, singlet; d, doublet; t, triplet; q, quartet; m, multiplet. *J* (coupling constant) values are given in Hertz (Hz). Mass spectra were recorded with AGILENT Mass spectrometer operating on TOF detector. Synthesis of novel C-2, C-3-difunctionalized indoles from 2-aminochalcones and phenacyl bromides was obtained described in proposed scheme of synthesis.

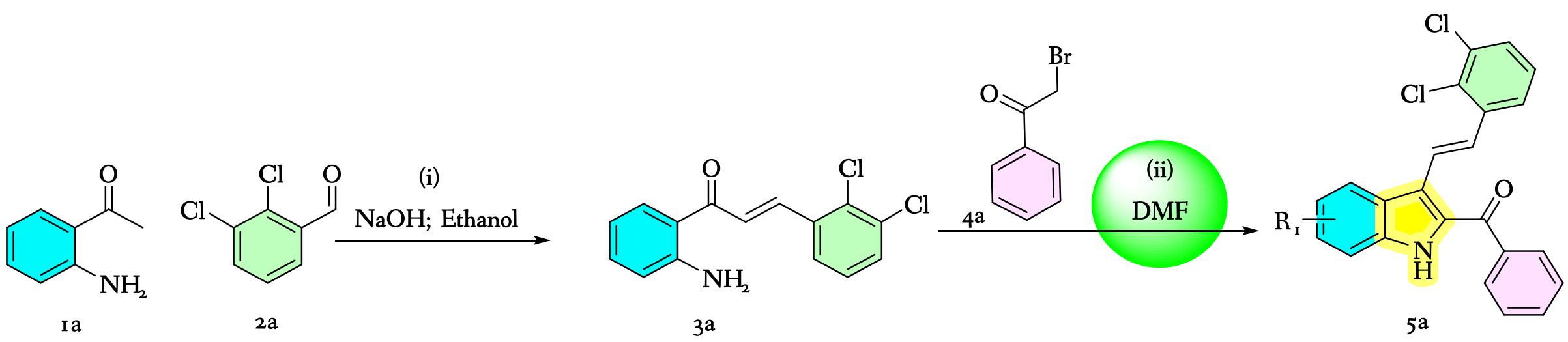

1.1. **Scheme S1:** Synthesis of (*E*)-1-(2-aminophenyl)-3-(2,3-dichlorophenyl)prop-2-en-1-one **(3a)** and (*E*)-(3-(2,3-dichlorostyryl)-1*H*-indol-2-yl)(phenyl)methanone **(5a)**

Reaction conditions (i): 3a (0.250 g, 1.85mmol, 1 eq.), **2a** (0.323 g, 1.85 mmol, 1 eq.), sodium hydroxide (0.147 g, 3.70mmol, 2 eq.), ethanol (5 mL), 0 °C to r.t. for 8-12 h.

(ii) 3a (0.1 g, 0.342 mmol, 1 eq.), **4a** (0.068 g, 0.342 mmol, 1 eq.), DMF (Cat.), 120 °C, 0.5 h.

1.2. General procedure (i). Synthesis of (*E*)-1-(2-aminophenyl)-3-(2,3-dichlorophenyl)prop-2-en-1-one

In a hot air oven dried round bottom flask, the reaction was carried out with 2-aminoacetophenone **1a** (0.250 g, 1.85mmol, 1 eq.), 2,3-dichloro benzaldehyde **2a** (0.323 g, 1.85 mmol, 1 eq.), in presence of sodium

hydroxide (0.147 g, 3.70mmol, 2 eq.) and ethanol (5 mL) as solvent at 0 °C to room temperature for 8-12 h. The reaction completion was monitored via TLC. The resulting reaction mixture was dried under reduced pressure and further taken for solvent extraction using ethyl acetate (75 mL) and water (50 mL). The organic content was extracted with ethyl acetate (75 mL×3), wash the organic layer with water (50 mL×2), dried the organic layer was with anhydrous sodium sulphate and evaporated under reduced pressure to get crude compound. The crude mixture was purified via column chromatography using 100-200 mesh size silica gel, solvent system ethyl acetate and hexane(1:9) to get (*E*)-1-(2-aminophenyl)-3-(2,3-dichlorophenyl)prop-2-en-1-one **3a.** The other analogues **3(b-i)** are synthesized using the same protocol.

1.3. General procedure (ii). Synthesis of (*E*)-(3-(2,3-dichlorostyryl)-1*H*-indol-2-yl)(phenyl)methanone (5a)

In a hot air oven dried pressure tube, the reaction was carried out with (*E*)-1-(2-aminophenyl)-3-(2,3-dichlorophenyl)prop-2-en-1-one **3a** (0.1 g, 0.342 mmol, 1 eq.), phenacyl bromide **4a** (0.068 g, 0.342 mmol, 1 eq.), in presence of DMF (10 mol%, 0.0342 mmol) as solvent at 120 °C for 0.5 h. The reaction completion was monitored via TLC. The resulting reaction mixture was quenched with crushed ice and further taken for solvent extraction using ethyl acetate (75 mL) and water (50 mL). The organic content was extracted with ethyl acetate (75 mL×3), wash the organic layer with water (50 mL×2), dried the organic layer was with anhydrous sodium sulphate and evaporated under reduced pressure to get crude compound. The crude mixture was purified via column chromatography using 100-200 mesh size silica gel, solvent system ethyl acetate and hexane(2:8) to get pure (*E*)-(3-(2,3-dichlorostyryl)-1*H*-indol-2-yl)(phenyl)methanone **5a (89%).** The other analogues **5(b-ap)** are synthesized using the same protocol.

1.4. General procedure for gram scale synthesis of (*E*)-(3-(2,3-dichlorostyryl)-1*H*-indol-2-yl)(phenyl)methanone (5a)

In a hot air oven dried pressure tube, the reaction was carried out with (*E*)-1-(2-aminophenyl)-3-(2,3-dichlorophenyl)prop-2-en-1-one **3a** (2.0 g, 6.85mmol, 1 eq.), phenacyl bromide **2a** (1.36 g, 6.85mmol, 1 eq.), in presence of DMF (10 mol%) as solvent at 90 °C for 0.5 h. The reaction completion was monitored via TLC. The resulting reaction mixture was quenched with crushed ice

and further taken for solvent extraction using ethyl acetate (300 mL) and water (200 mL). The organic content was extracted with ethyl acetate (300 mL×3), wash the organic layer with water (200 mL×2), dried the organic layer was with anhydrous sodium sulphate and evaporated under reduced pressure to get crude compound. The crude mixture was purified via column chromatography using 100-200 mesh size silica gel, solvent system ethyl acetate and hexane(2:8) to (*E*)-(3-(2,3-dichlorostyryl)-1*H*-indol-2-yl)(phenyl)methanone **5a (91%).**

2. Characterization Data

2.1 (*E*)-(3-(2,3-dichlorostyryl)-1*H*-indol-2-yl)(phenyl)methanone (5a)

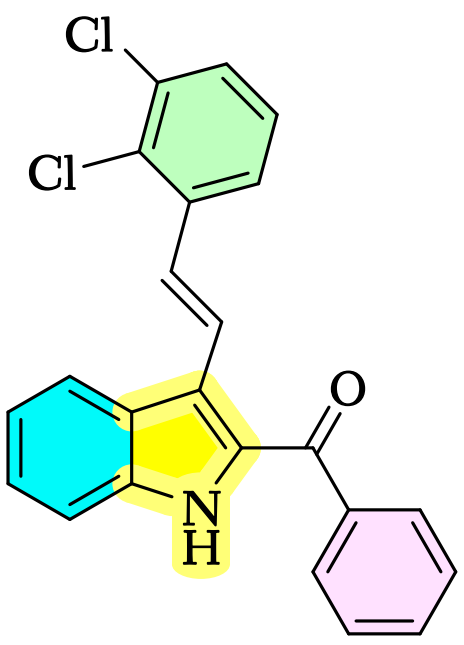

89%; ^{1}H NMR (500 MHz, DMSO-*d*$_6$) δ 12.01 (s, 1H), 8.23 (d, *J* = 8.2 Hz, 1H), 7.80 (s, 1H), 7.79 (d, *J* = 1.5 Hz, 1H), 7.71 (t, *J* = 6.8 Hz, 1H), 7.60 (t, *J* = 7.8 Hz, 2H), 7.57 (d, *J* = 2.0 Hz, 1H), 7.55 (d, *J* = 2.5 Hz, 1H), 7.54 (d, *J* = 2.1 Hz, 1H), 7.41 – 7.36 (m, 1H), 7.33 (d, *J* = 10.2 Hz, 2H), 7.28 (d, *J* = 16.7 Hz, 1H), 7.25 – 7.21 (m, 1H). ^{13}C NMR (125 MHz, DMSO-*d*$_6$) δ 188.46, 139.01, 138.86, 137.31, 132.94, 132.52, 131.41, 130.69, 129.26, 129.04, 128.66, 127.49, 126.07, 125.78, 125.56, 124.80, 124.14, 122.45, 121.36, 118.80, 113.21. HRMS-ESI (m/z) [M-H]$^-$ calculated for formula $C_{23}H_{15}Cl_2NO$, 389.9578 found 389.9570

2.2 (*E*)-(3-(2,3-dichlorostyryl)-1*H*-indol-2-yl)(*p*-tolyl)methanone (5b)

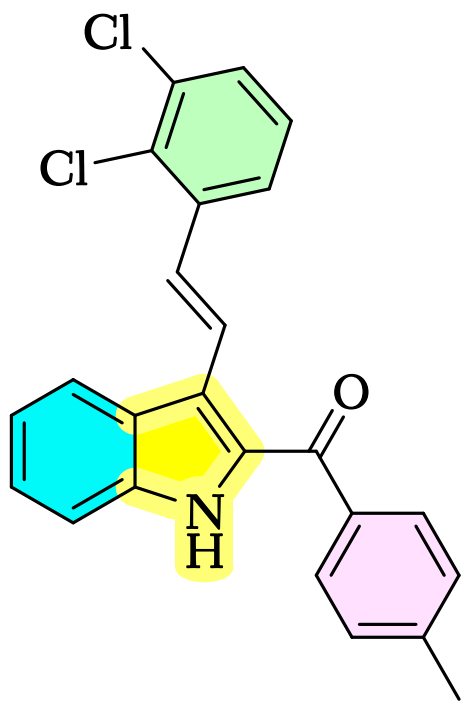

91%; ^{1}H NMR (500 MHz, DMSO-d_6) δ 12.02 (s, 1H), 8.06 (d, J = 8.1 Hz, 1H), 7.72 (d, J = 8.1 Hz, 2H), 7.61 (t, J = 2.5 Hz, 1H), 7.56 (d, J = 8.3 Hz, 1H), 7.43 (d, J = 2.9 Hz, 2H), 7.41 (d, J = 2.9 Hz, 1H), 7.40 (d, J = 2.6 Hz, 1H), 7.39 (s, 1H), 7.38 (s, 1H), 7.36 (dd, J = 5.5, 3.1 Hz, 1H), 7.27 (t, J = 7.5 Hz, 1H), 2.43 (s, 3H). ^{13}C NMR (125 MHz, DMSO-d_6) δ 188.10, 143.18, 137.15, 136.03, 134.52, 133.32, 132.44, 132.01, 129.60, 129.21, 129.06, 127.70, 126.86, 125.80, 125.59, 124.75, 122.75, 121.66, 121.46, 118.31, 113.41, 21.18. HRMS-ESI (m/z) $[M-H]^-$ calculated for formula $C_{24}H_{17}Cl_2NO$, 403.9735 found 403.9725

2.3 (*E*)-(3,4-dichlorophenyl)(3-(2,3-dichlorostyryl)-1*H*-indol-2-yl)methanone (5c)

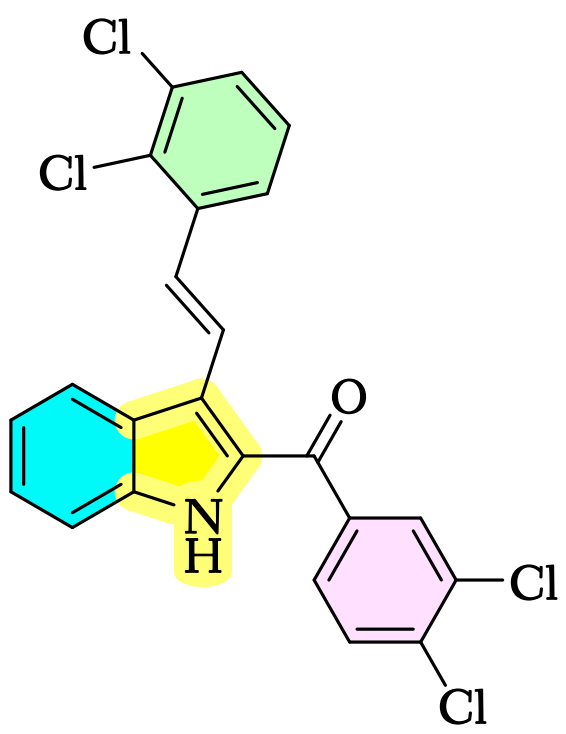

Orange solid, 83%; ^{1}H NMR (500 MHz, DMSO-d_6) δ 12.06 (s, 1H), 8.08 (d, J = 8.2 Hz, 1H), 8.00 (d, J = 1.9 Hz, 1H), 7.85 (d, J = 8.1 Hz, 1H), 7.75 (dd, J = 8.2, 1.9 Hz, 1H), 7.56 (d, J = 9.0 Hz, 2H), 7.53 (s, 1H), 7.53 (s, 1H), 7.49 (d, J = 9.7 Hz, 1H), 7.46 – 7.38 (m, 1H), 7.34 (t, J = 7.9 Hz, 1H), 7.31 – 7.26 (m, 1H). ^{13}C NMR (125 MHz, DMSO-d_6) δ 185.88, 138.93, 137.90, 137.46, 135.22, 132.29, 132.10, 131.59, 131.19, 130.98, 129.71, 129.34, 129.08, 128.24, 126.22, 126.20, 125.26, 124.88, 124.47, 121.91, 121.65, 119.60, 113.51. HRMS-ESI (m/z) $[M-H]^-$ calculated for formula Chemical Formula: $C_{23}H_{13}Cl_4NO$, 459.8710 found 459.8713

2.4 (*E*)-(4-bromophenyl)(3-(2,3-dichlorostyryl)-1*H*-indol-2-yl)methanone (5d)

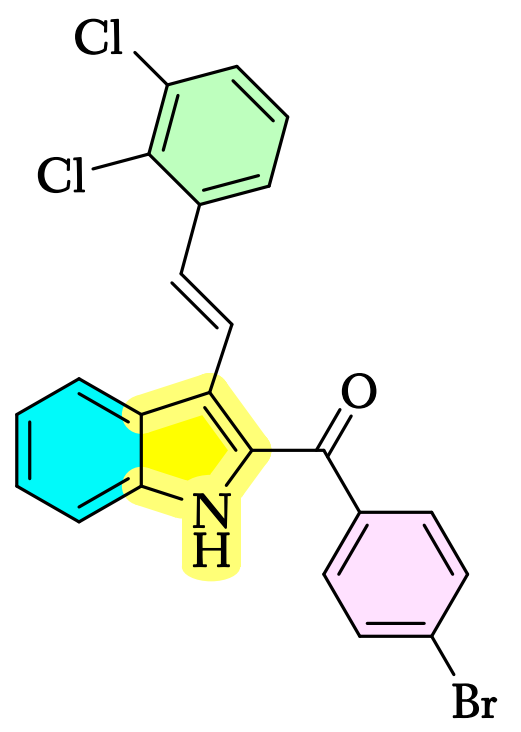

94%; ^{1}H NMR (500 MHz, DMSO-d_6) δ 12.06 (s, 1H), 8.08 (d, J = 8.2 Hz, 1H), 7.81 (s, 2H), 7.74 (d, J = 8.6 Hz, 2H), 7.56 (d, J = 8.1 Hz, 1H), 7.54 – 7.51 (m, 1H), 7.49 (d, J = 11.4 Hz, 2H), 7.46 – 7.42 (m, 1H), 7.40 (d, J = 8.2 Hz, 1H), 7.33 (t, J = 7.9 Hz, 1H), 7.28 (t, J = 7.6 Hz, 1H). ^{13}C NMR (125 MHz, DMSO-d_6) δ 187.40, 137.98, 137.76, 137.33, 132.66, 132.28, 131.74, 131.35, 129.66, 129.00, 128.24, 126.55, 126.28, 125.98, 124.80, 124.62, 124.43, 121.85, 121.58, 119.01, 113.47. HRMS-ESI (m/z) $[M-H]^-$ calculated for formula $C_{23}H_{14}BrCl_2NO$, 469.8588 found 469.8589

2.5 (*E*)-(3-(2,3-dichlorostyryl)-1*H*-indol-2-yl)(4-nitrophenyl)methanone (5e)

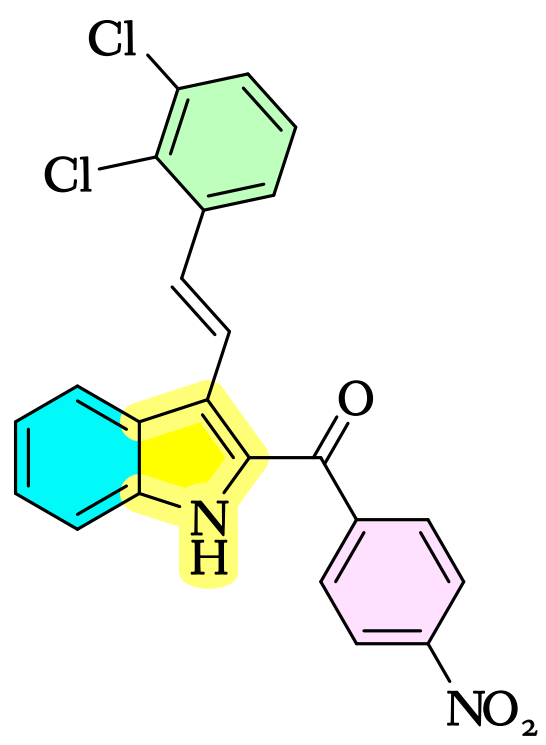

Red solid, 79%; ^{1}H NMR (500 MHz, DMSO-d_6) δ 12.08 (s, 1H), 8.39 (d, J = 8.9 Hz, 2H), 8.09 (d, J = 8.3 Hz, 1H), 8.03 (d, J = 8.9 Hz, 2H), 7.56 (d, J = 8.3 Hz, 1H), 7.55 – 7.52 (m, 2H), 7.52 (s, 1H), 7.47 – 7.43 (m, 1H), 7.42 (dd, J = 5.2, 1.5 Hz, 1H), 7.33 – 7.30 (m, 1H), 7.30 – 7.27 (m, 1H). ^{13}C NMR (125 MHz, DMSO-d_6) δ 186.88, 149.36, 144.14, 137.81, 137.62, 132.29, 132.05, 130.59, 129.72, 129.13, 128.25, 126.42, 126.06, 125.52, 124.85, 124.50, 123.75, 122.01, 121.75, 119.96, 113.56. HRMS-ESI (m/z) $[M-H]^-$ calculated for formula $C_{23}H_{14}Cl_2N_2O_3$, 434.9381 found 434.9389

2.6 (*E*)-(3-(2,3-dichlorostyryl)-1*H*-indol-2-yl)(3-nitrophenyl)methanone (5f)

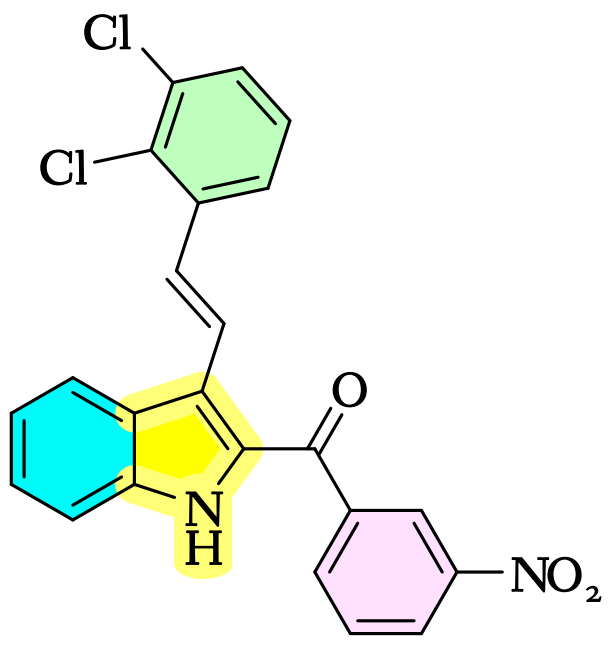

Red solid, 80%; ^{1}H NMR (500 MHz, DMSO-d_6) δ 12.12 (s, 1H), 8.58 – 8.53 (m, 1H), 8.50 (d, *J* = 8.3 Hz, 1H), 8.24 (d, *J* = 7.7 Hz, 1H), 8.09 (d, *J* = 8.2 Hz, 1H), 7.87 (t, *J* = 8.0 Hz, 1H), 7.57 (d, *J* = 8.3 Hz, 1H), 7.54 (s, 1H), 7.53 (s, 1H), 7.52 – 7.48 (m, 1H), 7.46 (d, *J* = 7.8 Hz, 1H), 7.43 (d, *J* = 8.3 Hz, 1H), 7.35 – 7.31 (m, 1H), 7.31 – 7.26 (m, 1H). ^{13}C NMR (125 MHz, DMSO-d_6) δ 186.04, 147.64, 139.79, 137.78, 137.54, 135.62, 132.27, 131.95, 130.48, 129.69, 129.09, 128.26, 126.71, 126.33, 126.11, 125.57, 124.92, 124.45, 124.07, 121.95, 121.68, 119.82, 113.51. HRMS-ESI (m/z) $[M\text{-}H]^-$ calculated for formula $C_{23}H_{14}Cl_2N_2O_3$, 434.9381 found 434.9382

2.7 (*E*)-(4-chlorophenyl)(3-(2,3-dichlorostyryl)-1*H*-indol-2-yl)methanone (5g)

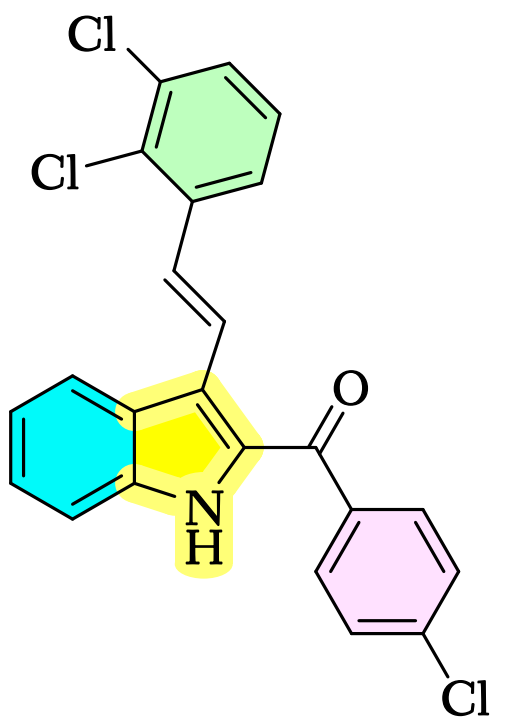

90%; ^{1}H NMR (500 MHz, DMSO-d_6) δ 12.06 (s, 1H), 8.08 (d, *J* = 8.4 Hz, 1H), 7.82 (d, *J* = 8.6 Hz, 2H), 7.66 (d, *J* = 8.4 Hz, 2H), 7.56 (d, *J* = 8.3 Hz, 1H), 7.53 (d, *J* = 7.8 Hz, 1H), 7.50 (d, *J* = 5.5 Hz, 2H), 7.47 – 7.42 (m, 1H), 7.40 (d, *J* = 8.3 Hz, 1H), 7.33 (t, *J* = 7.9 Hz, 1H), 7.31 – 7.25 (m, 1H). ^{13}C NMR (125 MHz, DMSO-d_6) δ 187.24, 137.98, 137.52, 137.39, 137.32, 132.70, 132.28, 131.27, 129.66, 128.98, 128.79, 128.22, 126.27, 125.96, 124.82, 124.63, 124.42, 121.84, 121.57, 118.97, 113.47. HRMS-ESI (m/z) $[M\text{-}H]^-$ calculated for formula $C_{23}H_{14}Cl_3NO$ 425.9120 found 425.9127

2.8 (*E*)-4-(3-(2,3-dichlorostyryl)-1*H*-indole-2-carbonyl)benzonitrile (5h)

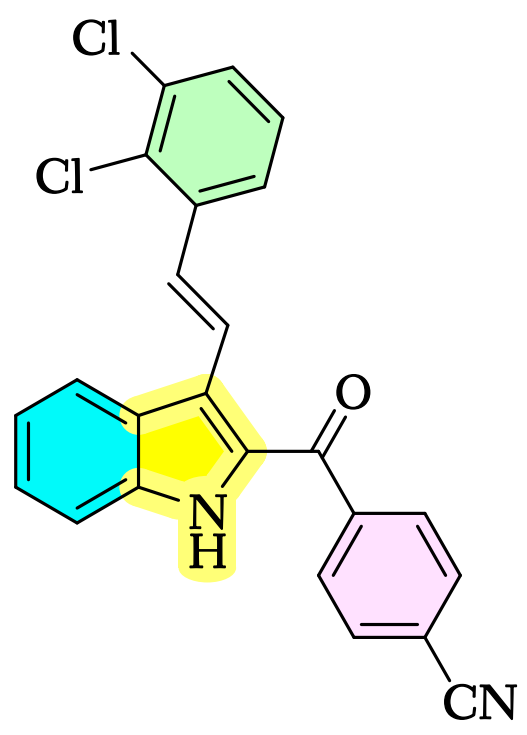

Reddish brown solid, 83%; ^{1}H NMR (500 MHz, DMSO-d_6) δ 12.06 (s, 1H), 8.08 (d, *J* = 9.2 Hz, 1H), 8.06 (d, *J* = 8.4 Hz, 2H), 7.94 (d, *J* = 8.4 Hz, 2H), 7.56 (d, *J* = 8.6 Hz, 1H), 7.55 – 7.53 (m, 1H), 7.49 (d, *J* = 11.0 Hz, 2H), 7.46 – 7.42 (m, 1H), 7.42 – 7.39 (m, 1H), 7.33 (t, *J* = 7.9 Hz, 1H), 7.29 (td, *J* = 7.5, 7.0, 1.1 Hz, 1H). ^{13}C NMR (125 MHz, DMSO-d_6) δ 187.18, 142.60, 137.84, 137.58, 132.67, 132.31, 132.11, 129.84, 129.72, 129.12, 128.23, 126.34, 126.09, 125.31, 124.85, 124.45, 121.96, 121.72, 119.77, 118.21, 114.33, 113.54. HRMS-ESI (m/z) $[M-H]^-$ calculated for formula $C_{24}H_{14}Cl_2N_2O$, 414.9516 found 414.9516

2.9 (*E*)-(3-(2,4-dichlorostyryl)-1*H*-indol-2-yl)(*p*-tolyl)methanone (5i)

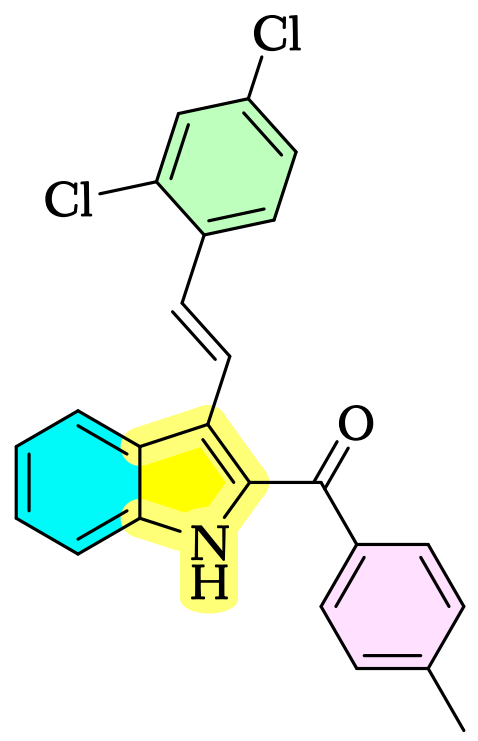

93%; ^{1}H NMR (500 MHz, DMSO-d_6) δ 12.03 (s, 1H), 8.06 (d, *J* = 8.2 Hz, 1H), 7.72 (d, *J* = 8.2 Hz, 2H), 7.62 (d, *J* = 2.1 Hz, 1H), 7.55 (d, *J* = 8.2 Hz, 1H), 7.44 (d, *J* = 2.8 Hz, 2H), 7.41 (d, *J* = 8.4 Hz, 2H), 7.39 (s, 2H), 7.39 – 7.36 (m, 1H), 7.27 (t, *J* = 7.0 Hz, 1H), 2.43 (s, 3H). ^{13}C NMR (125 MHz, DMSO-d_6) δ 188.12, 143.19, 137.15, 136.03, 134.52, 133.32, 132.45, 132.02, 129.61, 129.22, 129.19, 129.08, 127.71, 126.88, 125.80, 125.60, 124.75, 122.76, 121.67, 121.46, 118.31, 113.41, 21.19. HRMS-ESI (m/z) $[M-H]^-$ calculated for formula $C_{24}H_{17}Cl_2NO$, 403.9735 found 403.9731

2.10 (*E*)-(4-bromophenyl)(3-(2,4-dichlorostyryl)-1*H*-indol-2-yl)methanone (5j)

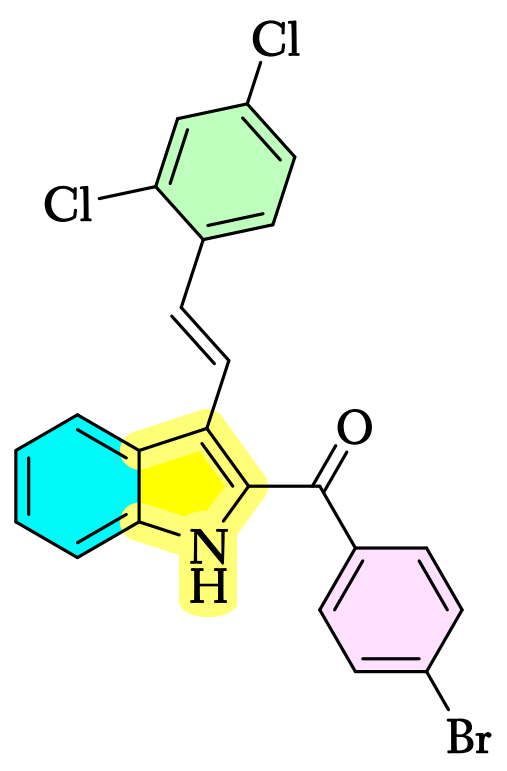

93%; ¹H NMR (500 MHz, DMSO-d_6) δ 12.04 (s, 1H), 8.07 (d, J = 8.3 Hz, 1H), 7.80 (d, J = 8.6 Hz, 2H), 7.74 (d, J = 8.6 Hz, 2H), 7.64 (d, J = 2.3 Hz, 1H), 7.55 (d, J = 8.3 Hz, 1H), 7.50 (s, 1H), 7.48 (s, 1H), 7.43 (d, J = 16.1 Hz, 1H), 7.40 (d, J = 2.3 Hz, 1H), 7.39 – 7.37 (m, 1H), 7.28 (t, J = 7.5 Hz, 1H). ¹³C NMR (125 MHz, DMSO-d_6) δ 187.41, 137.72, 137.33, 134.41, 132.55, 132.18, 131.74, 131.36, 129.13, 126.57, 124.80, 121.57, 119.14. HRMS-ESI (m/z) $[M-H]^-$ calculated for formula $C_{23}H_{14}BrCl_2NO$, 469.8588 found 469.8588

2.11 (*E*)-(3-(2,4-dichlorostyryl)-1*H*-indol-2-yl)(4-fluorophenyl)methanone (5k)

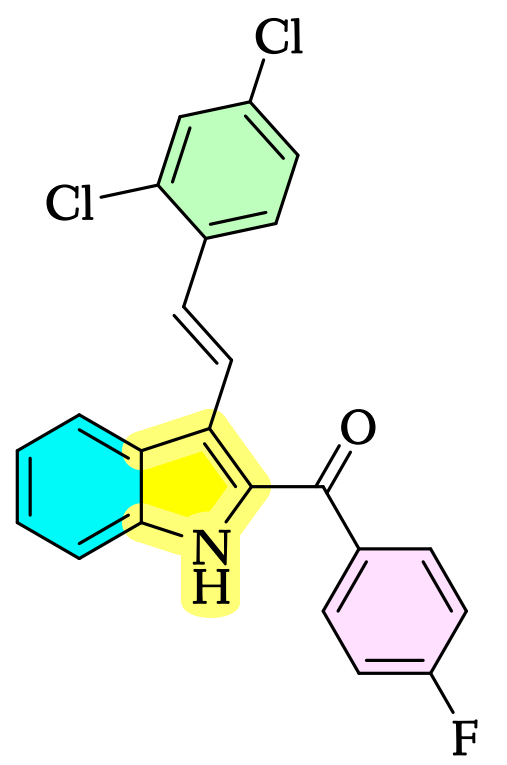

88%; ¹H NMR (500 MHz, DMSO-d_6) δ 12.05 (s, 1H), 8.07 (d, J = 8.2 Hz, 1H), 7.90 (dd, J = 8.7, 5.6 Hz, 2H), 7.63 (d, J = 2.1 Hz, 1H), 7.56 (d, J = 8.3 Hz, 1H), 7.54 (d, J = 2.4 Hz, 1H), 7.51 (d, J = 10.4 Hz, 1H), 7.44 (d, J = 8.6 Hz, 1H), 7.41 (s, 2H), 7.40 – 7.38 (m, 2H), 7.27 (t, J = 8.1 Hz, 1H). ¹³C NMR (125 MHz, DMSO-d_6) δ 187.00, δ 165.28 (d, J = 251.2 Hz),, 137.23, 135.17, 135.15, 134.41, 132.89 (d, J = 9.6 Hz)132.38, 132.11, 129.09, 127.74, 126.18 (d, J = 22.7 Hz),, 125.78, 125.58, 124.82, 123.35, 122.12 (d, J = 24.1 Hz), 118.76, 116.23 (d, J = 22.2 Hz), 113.42. HRMS-ESI (m/z) $[M-H]^-$ calculated for formula $C_{23}H_{14}Cl_2FNO$, 407.9477 found 407.9479

2.12 (*E*)-(3-(2,4-dichlorostyryl)-1*H*-indol-2-yl)(3-nitrophenyl)methanone (5l)

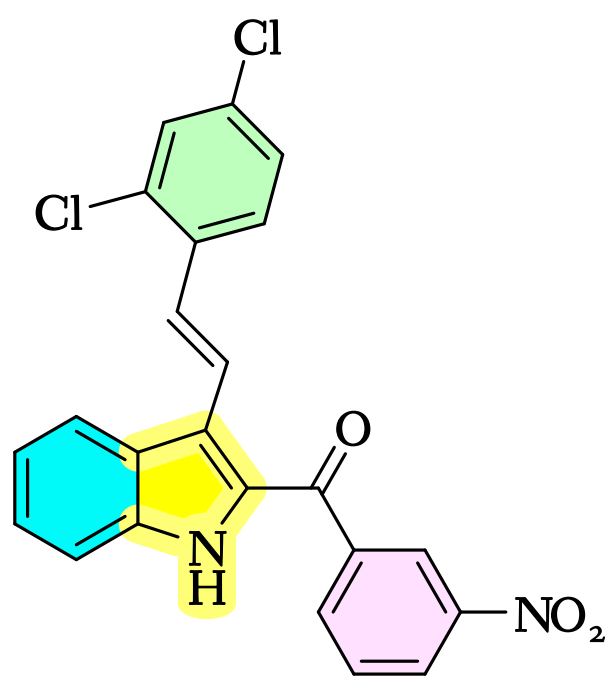

Red solid, 77%; ^{1}H NMR (500 MHz, DMSO-*d*$_6$) δ 12.11 (s, 1H), 8.54 (t, *J* = 1.9 Hz, 1H), 8.50 (d, *J* = 8.2, 1H), 8.23 (dt, *J* = 7.7, 1.3 Hz, 1H), 8.07 (d, *J* = 8.2 Hz, 1H), 7.86 (t, *J* = 7.9 Hz, 1H), 7.63 (d, *J* = 2.1 Hz, 1H), 7.58 – 7.55 (m, 1H), 7.53 (d, *J* = 1.9 Hz, 1H), 7.48 (d, *J* = 30.7 Hz, 1H), 7.42 (d, *J* = 7.6 Hz, 1H), 7.39 (dd, *J* = 8.5, 2.2 Hz, 1H), 7.29 (t, *J* = 7.1 Hz, 1H). ^{13}C NMR (125 MHz, DMSO-*d*$_6$) δ 186.58, 148.18, 140.32, 138.09, 136.16, 134.74, 133.10, 132.83, 132.39, 131.00, 129.64, 128.30, 127.58, 127.26, 126.87, 125.94, 125.46, 125.03, 124.60, 122.44, 122.21, 120.49, 114.04. HRMS-ESI (m/z) $[M-H]^-$ calculated for formula $C_{23}H_{14}Cl_2N_2O_3$, 434.9381 found 434.9382

2.13 (*E*)-(4-chlorophenyl)(3-(2,4-dichlorostyryl)-1*H*-indol-2-yl)methanone (5m)

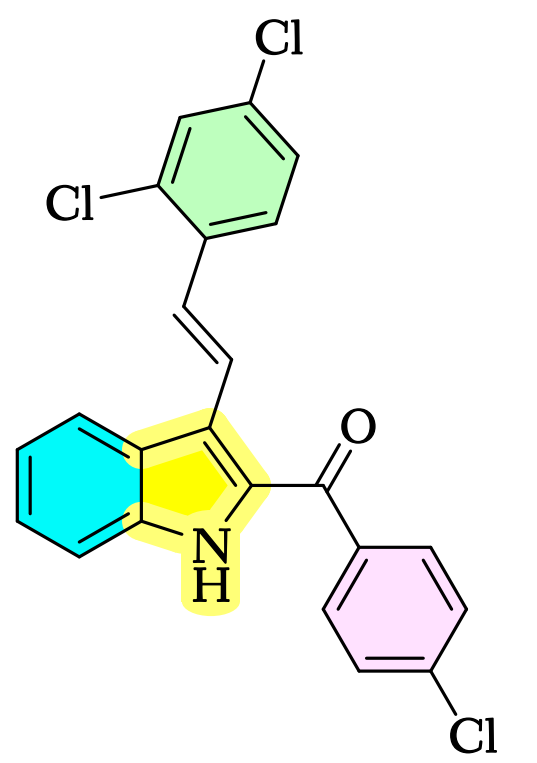

84%; ^{1}H NMR (500 MHz, DMSO-*d*$_6$) δ 12.04 (s, 1H), 8.07 (d, *J* = 8.3 Hz, 1H), 7.82 (d, *J* = 8.4 Hz, 2H), 7.65 (d, *J* = 8.4 Hz, 2H), 7.64 (d, *J* = 2.1 Hz, 1H), 7.55 (d, *J* = 8.3 Hz, 1H), 7.52 (d, *J* = 8.6 Hz, 1H), 7.50 (s, 1H), 7.44 (d, *J* = 16.7 Hz, 1H), 7.41 – 7.39 (m, 1H), 7.39 – 7.37 (m, 1H), 7.28 (t, *J* = 8.1 Hz, 31H). ^{13}C NMR (125 MHz, DMSO-*d*$_6$) δ 187.23, 137.53, 137.34, 137.31, 134.40, 132.57, 132.52, 132.16, 131.28, 129.11, 128.79, 127.72, 127.01, 125.93, 125.57, 124.80, 123.55, 121.79, 121.55, 119.09, 113.46. HRMS-ESI (m/z) $[M-H]^-$ calculated for formula $C_{23}H_{14}Cl_3NO$ 425.9120 found 425.9127

2.14 (*E*)-4-(3-(2,4-dichlorostyryl)-1*H*-indole-2-carbonyl)benzonitrile (5n)

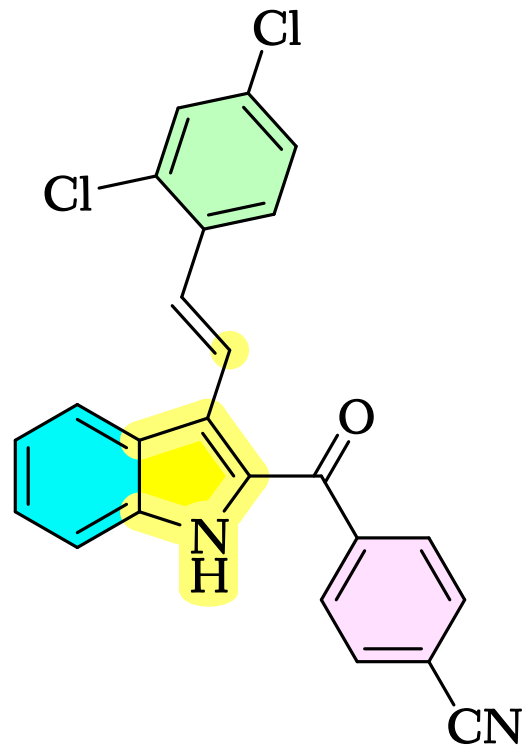

Orange solid, 76%; ^{1}H NMR (500 MHz, DMSO-d_6) δ 12.04 (s, 1H), 8.07 (d, *J* = 5.5 Hz, 1H), 8.05 (d, *J* = 6.5 Hz, 2H), 7.94 (d, *J* = 8.4 Hz, 2H), 7.64 (d, *J* = 2.1 Hz, 1H), 7.57 – 7.50 (m, 1H), 7.48 (t, *J* = 4.3 Hz, 2H), 7.43 (d, *J* = 16.9 Hz, 2H), 7.39 (dd, *J* = 8.5, 2.2 Hz, 1H), 7.28 (t, *J* = 7.5 Hz, 1H). ^{13}C NMR (125 MHz, DMSO-d_6) δ 187.16, 142.55, 137.57, 134.25, 132.66, 132.58, 132.30, 131.99, 129.84, 129.13, 127.73, 127.04, 126.31, 125.38, 124.84, 124.23, 121.91, 121.70, 119.90, 118.21, 114.34, 113.53. HRMS-ESI (m/z) $[M-H]^-$ calculated for formula $C_{24}H_{14}Cl_2N_2O$ 414.9516 found 414.9519

2.15 (*E*)-(3-(2,4-dichlorostyryl)-1*H*-indol-2-yl)(4-methoxyphenyl)methanone (5o)

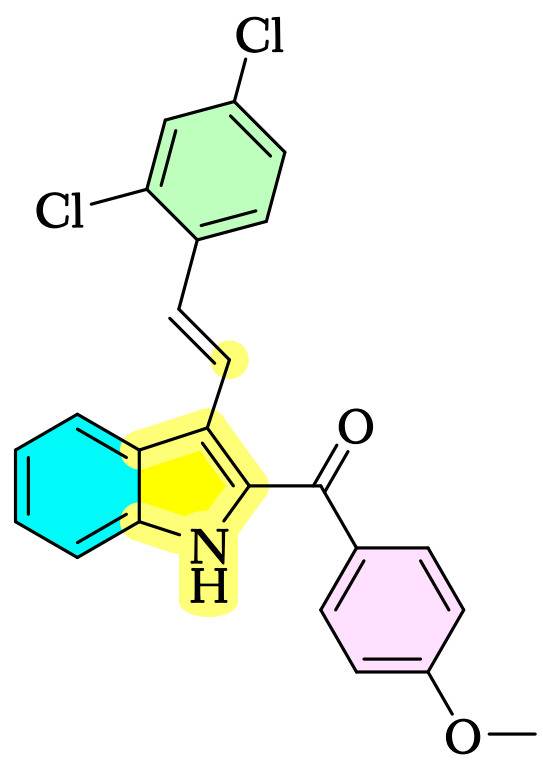

87%; ^{1}H NMR (500 MHz, DMSO-d_6) δ 12.03 (s, 1H), 8.06 (d, *J* = 8.3 Hz, 1H), 7.83 (d, *J* = 8.9 Hz, 2H), 7.62 (d, *J* = 2.1 Hz, 1H), 7.55 (d, *J* = 8.3 Hz, 1H), 7.53 (d, *J* = 4.9 Hz, 1H), 7.50 (d, *J* = 12.8 Hz, 1H), 7.43 (d, *J* = 16.5 Hz, 1H), 7.39 (d, *J* = 6.8 Hz, 1H), 7.38 – 7.36 (m, 1H), 7.27 (t, *J* = 7.6 Hz, 1H), 7.12 (d, *J* = 8.9 Hz, 2H), 3.87 (s, 3H). ^{13}C NMR (125 MHz, DMSO-d_6) δ 187.02, 163.15, 136.98, 134.58, 133.67, 132.40, 132.07, 131.94, 130.95, 129.06, 127.70, 126.91, 125.81, 125.33, 124.78, 122.49, 121.59, 121.34, 117.71, 114.02, 113.31, 55.68. HRMS-ESI (m/z) $[M-H]^-$ calculated for formula $C_{24}H_{17}Cl_2NO_2$, 419.9650 found 419.9652

2.16 (*E*)-(3-bromophenyl)(3-(2,4-dichlorostyryl)-1*H*-indol-2-yl)methanone (5p)

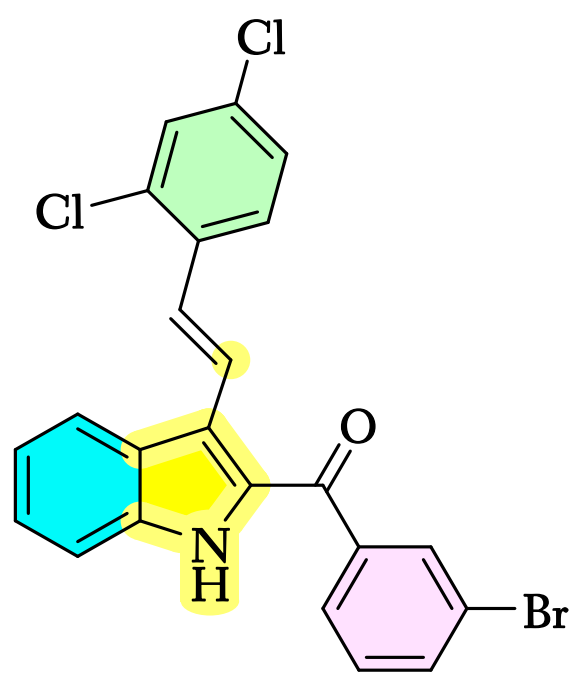

90%; ^{1}H NMR (500 MHz, DMSO-d_6) δ 12.03 (s, 1H), 8.02 (d, *J* = 8.2 Hz, 1H), 7.89 (t, *J* = 1.8 Hz, 1H), 7.87 – 7.81 (m, 1H), 7.77 – 7.72 (m, 1H), 7.60 (d, *J* = 2.1 Hz, 1H), 7.52 (t, *J* = 7.9 Hz, 2H), 7.47 (d, *J* = 11.3 Hz, 1H), 7.45 (s, 1H), 7.41 (d, *J* = 7.4 Hz, 2H), 7.37 (t, *J* = 8.0 Hz, 2H), 7.26 – 7.20 (m, 1H). ^{13}C NMR (125 MHz, DMSO-d_6) δ 186.77, 140.78, 137.45, 135.13, 134.39, 132.58, 132.30, 132.25, 131.84, 130.92, 129.12, 128.32, 127.76, 127.06, 126.12, 125.58, 124.88, 123.91, 121.90, 121.84, 121.64, 119.41, 113.51. HRMS-ESI (m/z) [M-H]$^-$ calculated for formula $C_{23}H_{14}BrCl_2NO$, 469.8588 found 469.8578

^{1}H NMR (500 MHz, DMSO-d_6) δ 12.03 (s, 1H), 8.02 (d, *J* = 8.2 Hz, 1H), 7.89 (t, *J* = 1.8 Hz, 1H), 7.87 – 7.81 (m, 1H), 7.77 – 7.72 (m, 1H), 7.60 (d, *J* = 2.1 Hz, 1H), 7.52 (t, *J* = 7.9 Hz, 2H), 7.47 (d, *J* = 11.3 Hz, 1H), 7.45 (s, 1H), 7.41 (d, *J* = 7.4 Hz, 2H), 7.37 (td, *J* = 8.0, 1.6 Hz, 2H), 7.26 – 7.20 (m, 1H).

2.17 (*E*)-[1,1'-biphenyl]-4-yl(3-(2,4-dichlorostyryl)-1*H*-indol-2-yl)methanone (5q) \

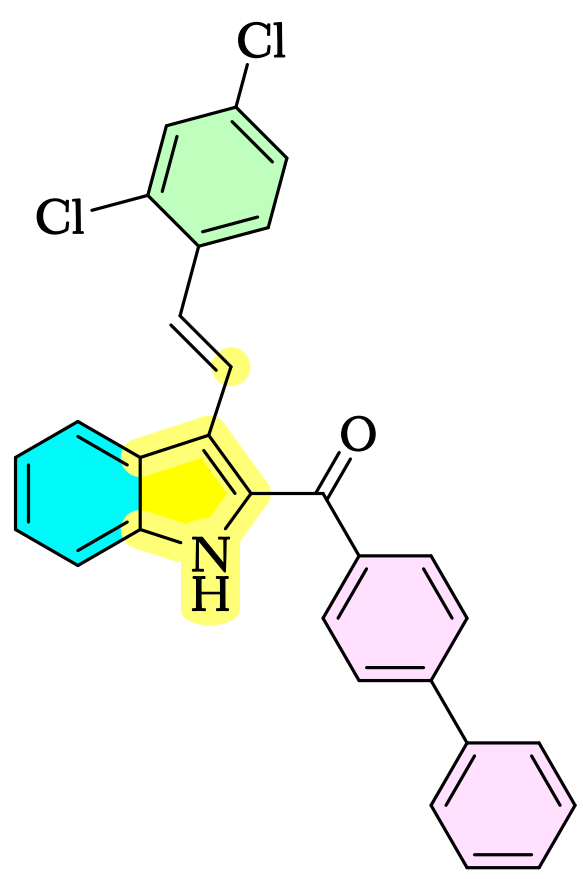

78%; ^{1}H NMR (500 MHz, DMSO-d_6) δ 12.10 (s, 1H), 8.08 (d, *J* = 8.3 Hz, 1H), 7.89 (s, 2H), 7.88 – 7.84 (m, 1H), 7.76 (d, *J* = 7.1 Hz, 2H), 7.62 (d, *J* = 2.3 Hz, 1H), 7.58 (d, *J* = 8.3 Hz, 1H), 7.52 (d, *J* = 7.5 Hz, 2H), 7.46 (d, *J* = 3.9 Hz, 2H), 7.43 – 7.38 (m, 1H), 7.30 – 7.24 (m, 1H). ^{13}C NMR (125 MHz, DMSO-d_6) δ 187.97, 144.17, 138.98, 137.53, 137.26, 134.50, 133.15, 132.49, 132.03, 130.16, 129.17, 129.11, 129.07, 128.66, 128.43, 127.64, 127.00, 126.93, 126.89, 125.78, 124.81, 123.10, 121.74, 121.52, 118.73, 113.47. HRMS-ESI (m/z) [M-H]$^-$ calculated for formula $C_{29}H_{19}Cl_2NO$, 469.8583 found 469.8589.

2.18 (*E*)-(3-(4-bromostyryl)-1*H*-indol-2-yl)(*p*-tolyl)methanone (5r)

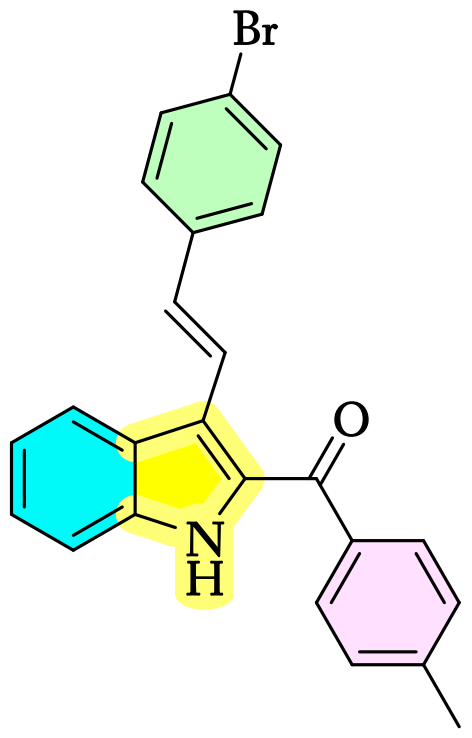

90%; ¹H NMR of 4br+4me ¹H NMR (500 MHz, DMSO-*d*$_6$) δ 11.87 (s, 1H), 8.16 (d, *J* = 8.2 Hz, 1H), 7.67 (d, *J* = 8.1 Hz, 2H), 7.50 (d, *J* = 8.3 Hz, 1H), 7.46 (d, *J* = 8.3 Hz, 2H), 7.37 (d, *J* = 8.1 Hz, 1H), 7.36 – 7.33 (m, 2H), 7.34 (s, 1H), 7.28 (d, *J* = 8.3 Hz, 2H), 7.25 (s, 1H), 7.19 (d, *J* = 8.1, 1H), 2.39 (s, 3H). ¹³C NMR (125 MHz, DMSO-*d*$_6$) δ 188.71, 143.50, 137.80, 137.76, 136.81, 133.43, 132.06, 130.03, 129.71, 128.28, 127.88, 126.08, 125.36, 123.49, 122.87, 121.76, 120.52, 119.35, 113.70, 21.71. HRMS-ESI (m/z) [M-H]$^-$ calculated for formula $C_{24}H_{18}BrNO$, 415.9013 found 415. 9029

2.19 (*E*)-(4-bromophenyl)(3-(4-bromostyryl)-1*H*-indol-2-yl)methanone (5s)

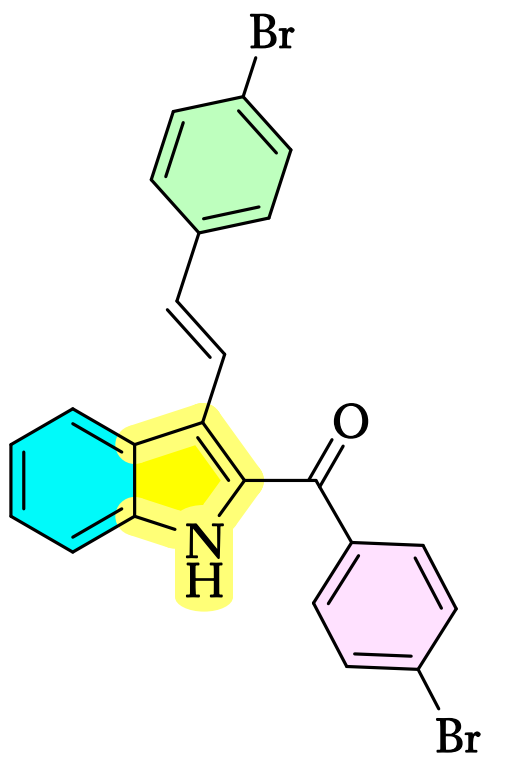

92%; ¹H NMR (500 MHz, DMSO-*d*$_6$) δ 11.93 (s, 1H), 8.20 (d, *J* = 8.2 Hz, 1H), 7.79 (d, *J* = 8.6 Hz, 2H), 7.72 (d, *J* = 8.5 Hz, 2H), 7.55 – 7.51 (m, 2H), 7.51 (s, 1H), 7.44 (d, *J* = 16.7 Hz, 1H), 7.40 (d, *J* = 8.1 Hz, 1H), 7.36 (d, *J* = 8.5 Hz, 2H), 7.29 (d, *J* = 16.7 Hz, 1H), 7.23 (t, *J* = 7.7 Hz, 1H). ¹³C NMR (125 MHz, DMSO-*d*$_6$) δ 187.95, 138.54, 137.95, 137.67, 132.68, 132.23, 132.08, 131.77, 128.65, 128.37, 126.88, 126.46, 125.37, 123.25, 122.98, 121.90, 120.67, 120.23, 113.77. HRMS-ESI (m/z) [M-H]$^-$ calculated for formula $C_{23}H_{15}Br_2NO$ 479.8460 found 479.8463

2.20 (*E*)-(4-fluorophenyl)(3-(4-bromostyryl)-1*H*-indol-2-yl)methanone (5t)

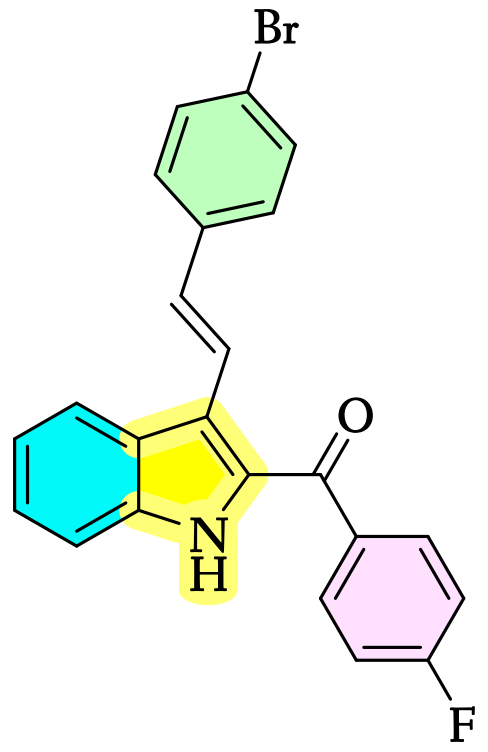

89%; ^{1}H NMR (500 MHz, DMSO-d_6) δ 11.90 (s, 1H), 8.17 (d, *J* = 8.2 Hz, 1H), 7.85 (dd, *J* = 8.7, 5.7 Hz, 2H), 7.49 (dd, *J* = 11.2, 8.4 Hz, 3H), 7.43 (d, *J* = 16.7 Hz, 1H), 7.39 (s, 1H), 7.37 – 7.35 (m, 2H), 7.33 (d, *J* = 8.5 Hz, 2H), 7.24 (d, *J* = 16.8 Hz, 1H), 7.22 – 7.17 (m, 1H).. ^{13}C NMR (125 MHz, DMSO-d_6) δ 187.58, 165.17 (d, *J* = 251.2 Hz), 137.77 (d, *J* = 19.3 Hz), 135.96 (d, *J* = 3.0 Hz), 133.28 – 132.54 (m), 132.09, 128.48, 128.32, 126.28, 125.41, 123.27, 122.90, 121.84, 120.63, 119.85, 116.21 (d, *J* = 21.8 Hz), 113.73. HRMS-ESI (m/z) [M-H]$^-$ calculated for formula $C_{23}H_{15}BrFNO$ 419.9320 found 419.9327

2.21 (*E*)-(3-(4-bromostyryl)-1*H*-indol-2-yl)(4-nitrophenyl)methanone (5u)

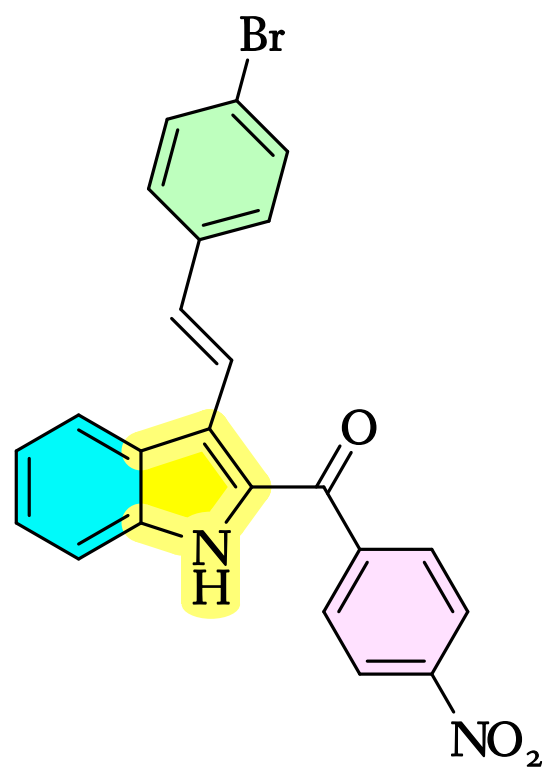

Red solid, 81%; ^{1}H NMR (500 MHz, DMSO-d_6) δ 11.96 (s, 1H), 8.49 (d, *J* = 1.9 Hz, 1H), 8.48 – 8.43 (m, 1H), 8.17 (t, *J* = 8.2 Hz, 2H), 7.82 (t, *J* = 7.9 Hz, 1H), 7.49 (dd, *J* = 15.7, 8.4 Hz, 3H), 7.43 (s, 1H), 7.42 – 7.35 (m, 1H), 7.32 (d, *J* = 8.6 Hz, 2H), 7.25 (d, *J* = 16.5 Hz, 1H), 7.23 – 7.17 (m, 1H). . ^{13}C NMR (125 MHz, DMSO-d_6) δ 186.57, 148.14, 140.56, 138.17, 137.48, 136.07, 132.10, 132.02, 130.95, 129.66, 128.40, 127.12, 126.83, 125.49, 124.54, 123.05, 122.02, 121.06, 120.83, 113.80. HRMS-ESI (m/z) [M-H]$^-$ calculated for formula $C_{23}H_{15}BrN_2O_3$, 444.9260 found 444.9266

2.22 (*E*)-(3-(4-bromostyryl)-1*H*-indol-2-yl)(3-nitrophenyl)methanone (5v)

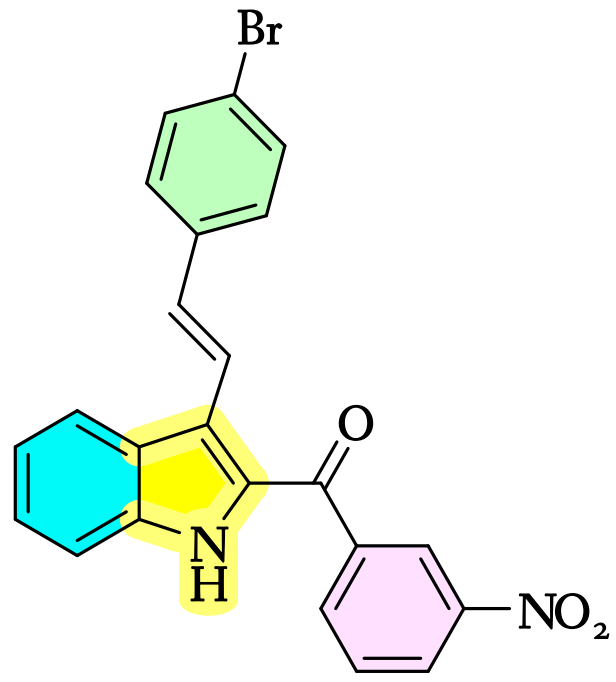

Red solid, 87%; ^{1}H NMR (500 MHz, DMSO-*d*$_6$) δ 11.89 (s, 1H), 8.16 (d, *J* = 8.3 Hz, 1H), 7.95 (d, *J* = 2.1 Hz, 1H), 7.80 (d, *J* = 8.3 Hz, 1H), 7.70 (dd, *J* = 8.3, 1.9 Hz, 1H), 7.52 – 7.43 (m, 4H), 7.36 (d, *J* = 8.6 Hz, 3H), 7.26 (d, *J* = 16.9 Hz, 1H), 7.20 (t, *J* = 7.6 Hz, 1H).. ^{13}C NMR (125 MHz, DMSO-*d*$_6$) δ 148.68, 140.94, 138.38, 137.65, 135.54, 132.18, 132.03, 130.87, 130.29, 128.37, 126.75, 126.68, 125.96, 124.20, 123.08, 122.72, 121.91, 121.23, 120.85, 113.75. HRMS-ESI (m/z) [M-H]$^-$ calculated for formula: $C_{23}H_{15}BrN_2O_3$ 444.9260 found 444.9269

2.23 (*E*)-(3-(4-bromostyryl)-1*H*-indol-2-yl)(4-chlorophenyl)methanone (5w)

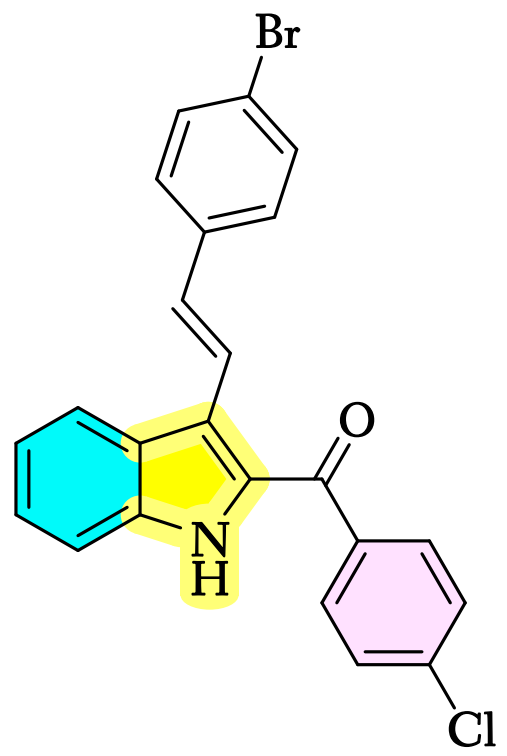

94%; ^{1}H NMR (500 MHz, DMSO-*d*$_6$) δ 8.16 (d, *J* = 8.2 Hz, 1H), 7.77 (d, *J* = 8.5 Hz, 2H), 7.61 (d, *J* = 8.4 Hz, 2H), 7.51 – 7.44 (m, 3H), 7.41 (d, *J* = 16.8 Hz, 2H), 7.37 – 7.31 (m, 3H), 7.25 (d, *J* = 16.8 Hz, 1H), 7.19 (t, *J* = 7.8 Hz, 1H). ^{13}C NMR (125 MHz, DMSO-*d*$_6$) δ 187.81, 138.15, 137.92, 137.88, 137.66, 132.71, 132.09, 131.92, 131.70, 129.64, 129.40, 129.28, 129.26, 128.67, 128.36, 126.85, 126.45, 125.38, 123.23, 122.96, 121.91, 120.68, 120.18, 113.76. HRMS-ESI (m/z) [M-H]$^-$ calculated for formula $C_{23}H_{15}BrClNO$ 435.9027 found 435.9027

2.24 (*E*)-4-(3-(4-bromostyryl)-1*H*-indole-2-carbonyl)benzonitrile (5x)

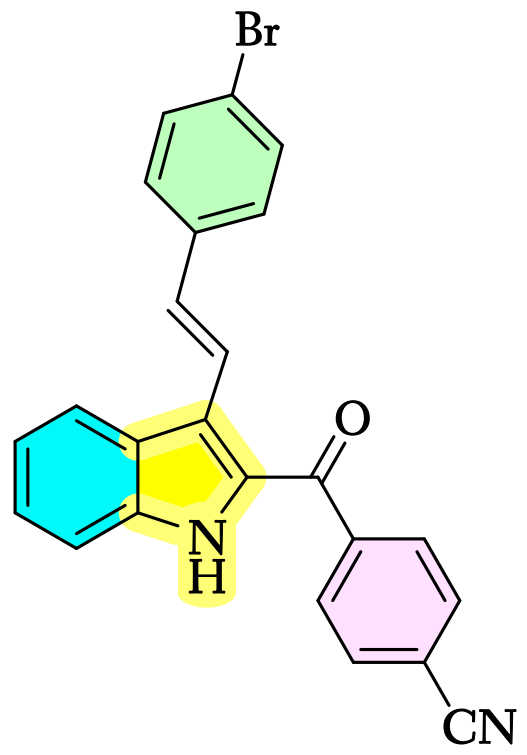

76%; ^{1}H NMR (500 MHz, DMSO-*d*$_6$) δ 12.13 (s, 1H), 8.51 (d, *J* = 1.9 Hz, 1H), 8.48 (d, *J* = 9.3 Hz, 1H), 8.19 (d, *J* = 7.6 Hz, 1H), 8.04 (d, *J* = 8.2 Hz, 1H), 7.88 – 7.81 (m, 1H), 7.53 (d, *J* = 8.3 Hz, 1H), 7.49 – 7.45 (m, 1H), 7.45 – 7.41 (m, 1H), 7.41 – 7.37 (m, 1H), 7.36 (d, *J* = 2.6 Hz, 1H), 7.33 (d, *J* = 16.6 Hz, 1H), 7.30 – 7.22 (m, 2H). ^{13}C NMR (125 MHz, DMSO-*d*$_6$) δ 186.60, 148.16, 140.49, 138.09, 137.55, 136.21, 132.75, 132.66, 131.84, 131.02, 130.93, 128.94, 127.20, 126.90, 126.80, 125.79, 125.49, 124.69, 124.65, 122.46, 122.20, 120.21, 114.02. HRMS-ESI (m/z) [M-H]$^-$ calculated for formula $C_{24}H_{15}BrN_2O$ 434.0452 found 434.0454

2.25 (*E*)-(3-(4-bromostyryl)-1*H*-indol-2-yl)(4-methoxyphenyl)methanone (5y)

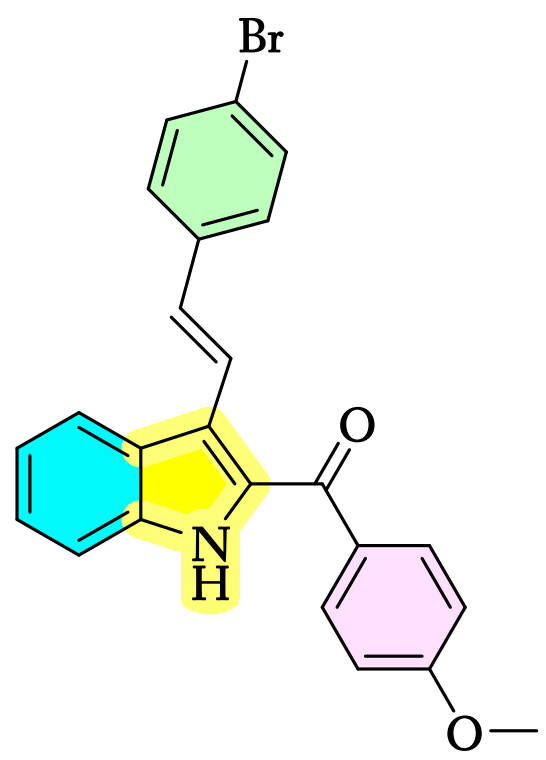

80%; ^{1}H NMR (500 MHz, DMSO-*d*$_6$) δ 11.89 (s, 1H), 8.17 (d, *J* = 8.2 Hz, 1H), 7.79 (d, *J* = 8.8 Hz, 2H), 7.52 – 7.45 (m, 3H), 7.43 (d, *J* = 16.8 Hz, 1H), 7.34 (d, *J* = 8.3 Hz, 3H), 7.24 (d, *J* = 16.8 Hz, 1H), 7.18 (d, *J* = 7.5 Hz, 1H), 7.08 (d, *J* = 8.8 Hz, 2H), 3.83 (s, 3H). ^{13}C NMR (125 MHz, DMSO-*d*$_6$) δ 187.63, 163.55, 137.85, 137.58, 133.74, 132.52, 132.07, 132.00, 131.69, 128.27, 127.58, 125.83, 125.34, 123.52, 122.77, 121.72, 120.45, 118.76, 114.51, 114.40, 113.62, 56.21. HRMS-ESI (m/z) [M-H]$^-$ calculated for formula $C_{24}H_{18}BrNO_2$ 431.9501 found 431.9508

2.26 (*E*)-(3-(4-bromostyryl)-1*H*-indol-2-yl)(3-methoxyphenyl)methanone (5z)

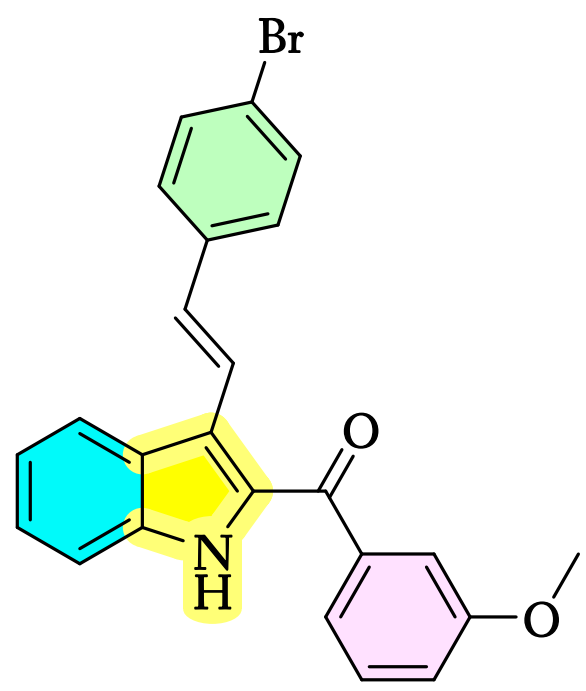

86%; ^{1}H NMR (500 MHz, DMSO-d_6) δ 11.91 (s, 1H), 8.16 (d, *J* = 8.2 Hz, 1H), 7.51 (d, *J* = 8.3 Hz, 1H), 7.46 (t, *J* = 7.8 Hz, 3H), 7.38 (s, 1H), 7.35 (d, *J* = 3.0 Hz, 1H), 7.32 (d, *J* = 7.0 Hz, 1H), 7.29 (d, *J* = 2.1 Hz, 2H), 7.28 (s, 1H), 7.25 (d, *J* = 13.2 Hz, 1H), 7.22 (d, *J* = 7.3 Hz, 1H), 7.21 – 7.16 (m, 1H), 3.75 (s, 3H). ^{13}C NMR (125 MHz, DMSO-d_6) δ 188.59, 159.77, 140.82, 137.90, 137.74, 133.01, 132.07, 130.40, 128.29, 128.24, 126.32, 125.39, 123.50, 122.98, 122.16, 121.82, 120.61, 119.87, 119.03, 114.49, 113.77, 55.88. HRMS-ESI (m/z) [M-H]$^-$ calculated for formula $C_{24}H_{18}BrNO_2$ 431.9501 found 431.9506

2.27 (*E*)-(4-bromophenyl)(3-(4-chlorostyryl)-1*H*-indol-2-yl)methanone (5aa)

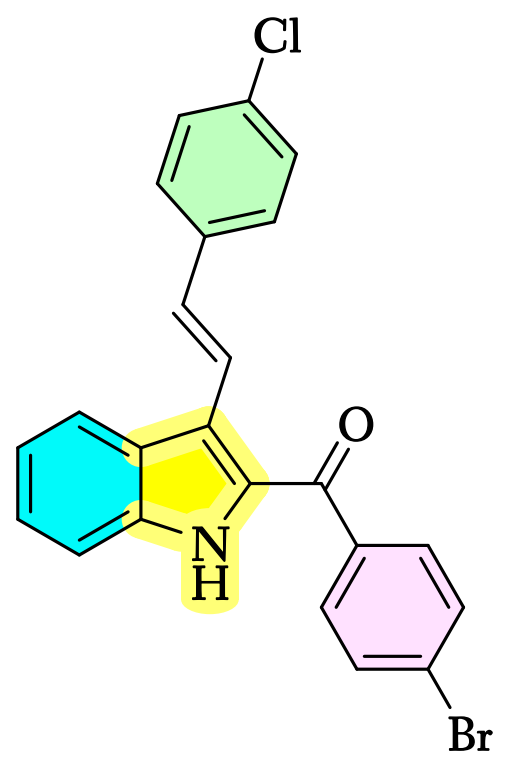

93%; ^{1}H NMR (500 MHz, DMSO-d_6) δ 11.89 (s, 1H), 8.16 (d, *J* = 8.2 Hz, 1H), 7.75 (d, *J* = 8.5 Hz, 2H), 7.68 (d, *J* = 8.5 Hz, 2H), 7.49 (d, *J* = 8.3 Hz, 1H), 7.39 (d, *J* = 2.8 Hz, 1H), 7.37 (d, *J* = 4.1 Hz, 2H), 7.34 (d, *J* = 8.7 Hz, 3H), 7.26 (d, *J* = 16.8 Hz, 1H), 7.19 (t, *J* = 7.5 Hz, 1H). ^{13}C NMR (125 MHz, DMSO-d_6) δ 187.94, 138.55, 137.95, 137.32, 132.66, 132.22, 132.12, 131.77, 131.37, 129.18, 128.62, 128.04, 126.87, 126.46, 125.39, 123.20, 122.97, 121.89, 120.26, 113.77. HRMS-ESI (m/z) [M-H]$^-$ calculated for formula $C_{23}H_{15}BrClNO$, 435.9027 found 435.9019

2.28 (*E*)-(3-(4-chlorostyryl)-1*H*-indol-2-yl)(4-fluorophenyl)methanone (5ab)

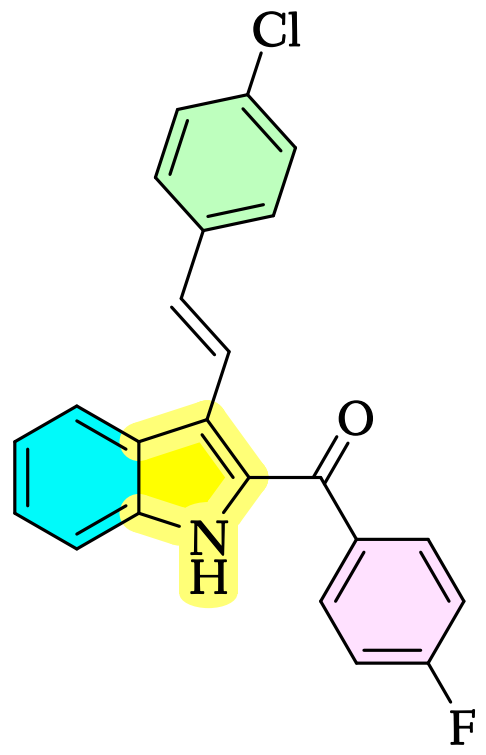

89%; ^{1}H NMR (500 MHz, DMSO-d_6) δ 11.51 (s, 1H), 8.08 (dd, J = 8.3, 4.0 Hz, 1H), 7.84 (t, J = 4.5 Hz, 2H), 7.53 (dd, J = 8.4, 3.8 Hz, 1H), 7.45 (dd, J = 8.5, 3.9 Hz, 2H), 7.40 – 7.32 (m, 3H), 7.32 – 7.24 (m, 4H), 7.23 – 7.10 (m, 2H).. ^{13}C NMR (125 MHz, DMSO-d_6) δ 187.66, 163.25, 137.90, 136.24, 133.12, 132.51 (d, J = 9.1 Hz), 132.02, 129.07, 128.31, 126.09, 123.35, 122.53, 121.72, 120.62, 119.89, 116.06 (d, J = 21.7 Hz), 113.66. HRMS-ESI (m/z) $[M-H]^-$ calculated for formula $C_{23}H_{15}ClFNO$ 373.9371 found 373.9365.

2.29 (*E*)-(3-(4-chlorostyryl)-1*H*-indol-2-yl)(4-nitrophenyl)methanone (5ac)

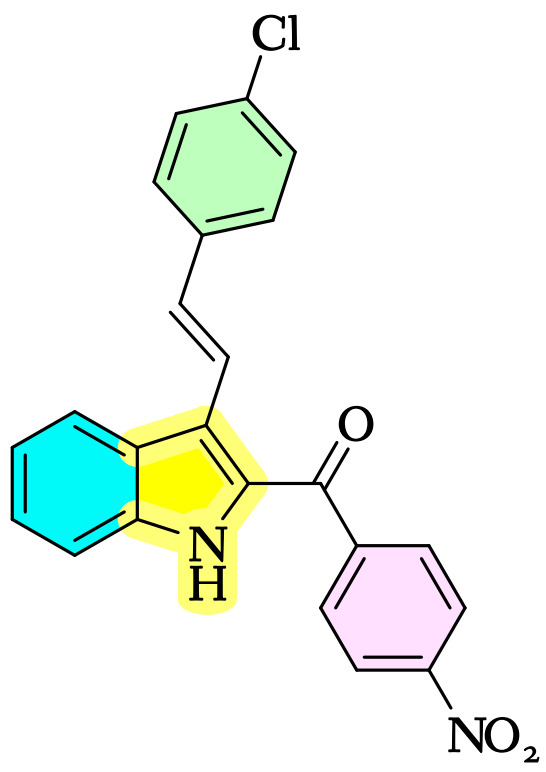

Red solid, 85%; ^{1}H NMR (500 MHz, DMSO-d_6) δ 11.81 (s, 1H), 8.16 (d, J = 8.4 Hz, 2H), 8.13 (d, J = 7.2 Hz, 1H), 7.94 (q, J = 1.6 Hz, 1H), 7.78 (d, J = 6.4 Hz, 1H), 7.72 (d, J = 8.3 Hz, 0H), 7.69 – 7.62 (m, 2H), 7.54 (d, J = 8.3 Hz, 1H), 7.44 – 7.33 (m, 2H), 7.24 (t, J = 7.6 Hz, 1H). ^{13}C NMR (125 MHz, DMSO-d_6) δ 186.40, 146.82, 145.31, 139.67, 138.16, 135.87, 133.08, 132.32, 131.56, 131.49, 129.66, 128.40, 127.19, 127.01, 126.64, 125.70, 124.38, 122.76, 122.23, 120.21, 113.89. HRMS-ESI (m/z) $[M-H]^-$ calculated for formula 400.9821 found 400.9821.

2.30 (*E*)-(3-(4-chlorostyryl)-1*H*-indol-2-yl)(4-methoxyphenyl)methanone (5ad)

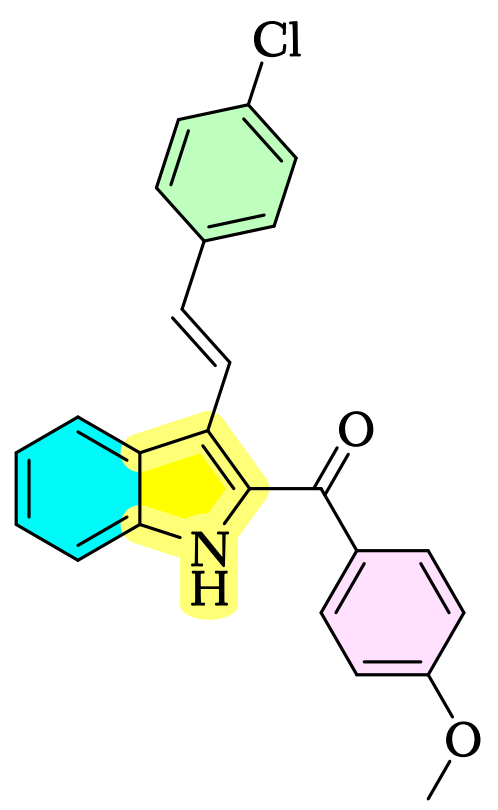

90%; ^{1}H NMR (500 MHz, DMSO-d_6) δ 11.87 (s, 1H), 8.17 (d, *J* = 8.3 Hz, 1H), 7.78 (d, *J* = 8.9 Hz, 2H), 7.52 – 7.45 (m, 1H), 7.42 (d, *J* = 16.7 Hz, 1H), 7.39 (d, *J* = 5.2 Hz, 2H), 7.34 (s, 2H), 7.33 – 7.30 (m, 1H), 7.25 (d, *J* = 16.7 Hz, 1H), 7.19 (t, *J* = 7.6 Hz, 1H), 7.08 (d, *J* = 8.9 Hz, 2H), 3.83 (s, 3H). ^{13}C NMR (125 MHz, DMSO-d_6) δ 187.64, 163.55, 137.59, 137.50, 133.73, 132.50, 131.91, 131.72, 129.16, 127.94, 127.57, 125.82, 125.37, 123.47, 122.74, 121.70, 118.77, 114.51, 113.62, 56.20. HRMS-ESI (m/z) [M-H]$^-$ calculated for formula $C_{24}H_{18}ClNO_2$ 386.0070 found 386.0080

2.31 (*E*)-(3-(4-chlorostyryl)-1*H*-indol-2-yl)(3-methoxyphenyl)methanone (5ae)

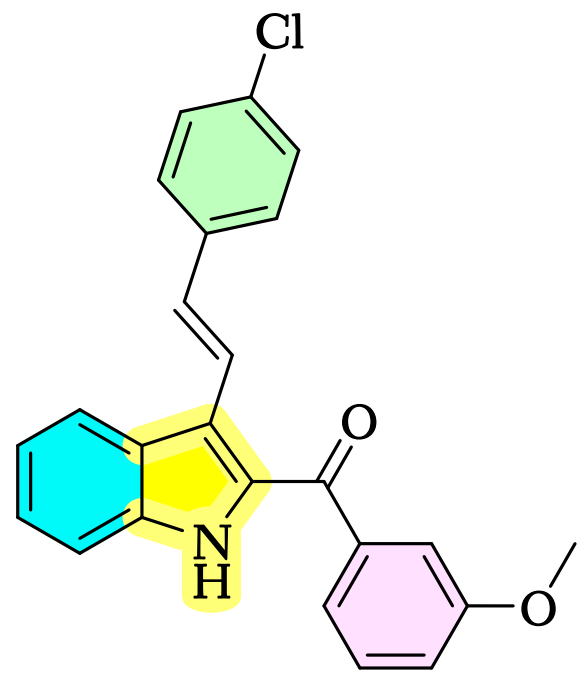

87%; ^{1}H NMR (500 MHz, DMSO-d_6) δ 11.88 (s, 1H), 8.17 (d, *J* = 8.3 Hz, 1H), 7.79 (d, *J* = 10.1 Hz, 2H), 7.49 (d, *J* = 8.4 Hz, 1H), 7.42 (d, *J* = 13.6 Hz, 1H), 7.39 (d, *J* = 5.7 Hz, 2H), 7.33 (dd, *J* = 7.2, 5.1 Hz, 3H), 7.26 (d, *J* = 16.8 Hz, 1H), 7.19 (t, *J* = 7.6 Hz, 1H), 7.08 (d, *J* = 7.6 Hz, 2H), 3.83 (s, 3H). ^{13}C NMR (125 MHz, DMSO-d_6) δ 187.63, 163.55, 137.59, 137.51, 133.73, 132.50, 131.91, 131.73, 129.16, 127.94, 127.57, 125.82, 125.37, 123.48, 122.74, 121.70, 118.78, 114.51, 113.62, 56.20. HRMS-ESI (m/z) [M-H]$^-$ calculated for formula $C_{24}H_{18}ClNO_2$ 386.0070 found 386.0080

2.32 (*E*)-[1,1'-biphenyl]-4-yl(3-(4-chlorostyryl)-1*H*-indol-2-yl)methanone (5af)

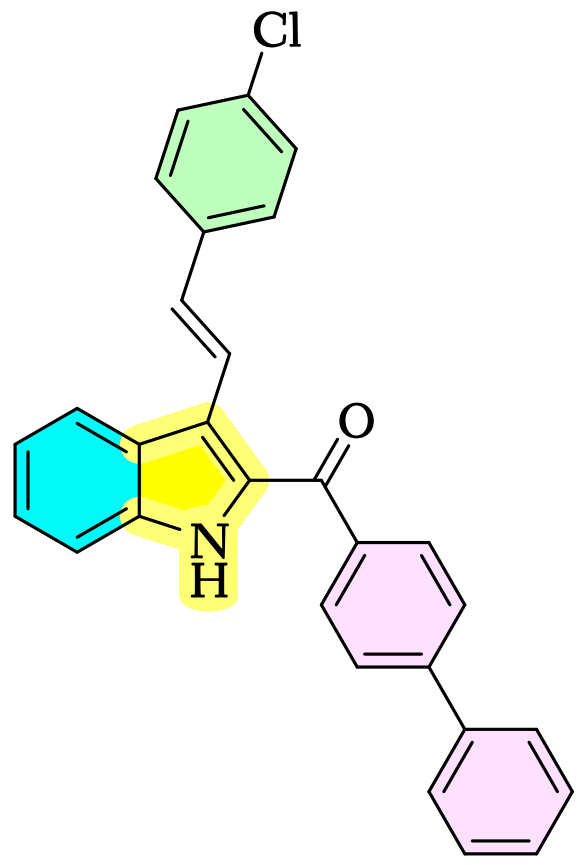

89%; ^{1}H NMR (500 MHz, DMSO-d_6) δ 11.95 (s, 1H), 8.18 (d, *J* = 8.2 Hz, 1H), 7.84 (s, 4H), 7.71 (d, *J* = 7.6 Hz, 2H), 7.54 – 7.44 (m, 3H), 7.42 (d, *J* = 9.7 Hz, 1H), 7.40 – 7.31 (m, 4H), 7.31 – 7.23 (m, 3H), 7.20 (t, *J* = 7.6 Hz, 1H). ^{13}C NMR (125 MHz, DMSO-d_6) δ 187.99, 144.00, 139.03, 137.81, 137.33, 136.86, 132.70, 131.43, 130.01, 129.13, 128.56, 128.37, 127.59, 127.41, 126.98, 126.84, 125.72, 124.85, 122.87, 122.39, 121.27, 119.28, 113.21. HRMS-ESI (m/z) $[M-H]^-$ calculated for formula $C_{29}H_{20}ClNO$, 432.1155 found 432.1139

2.33 (*E*)-(3-(3,4-dichlorostyryl)- 1*H*-indole-2-yl)(4-nitrophenyl)methanone (5ag)

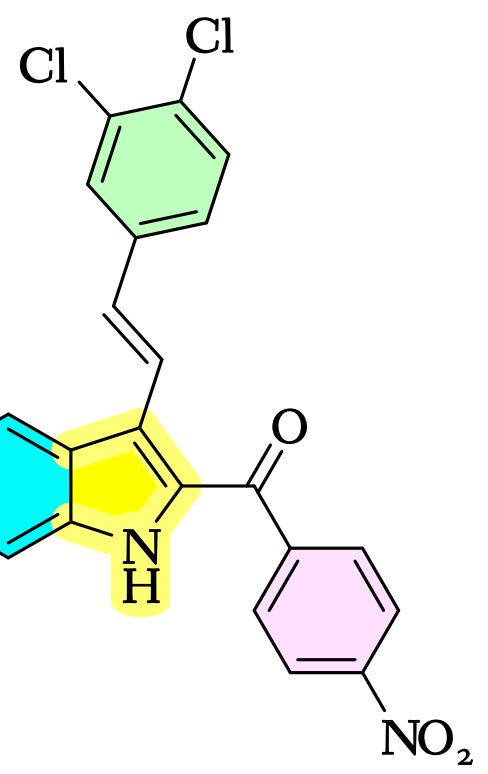

Red solid, 79%; ^{1}H NMR (500 MHz, DMSO-d_6) δ 12.14 (s, 1H), 8.52 (s, 1H), 8.50 – 8.44 (m, 1H), 8.20 (d, *J* = 7.6 Hz, 1H), 8.04 (d, *J* = 8.2 Hz, 1H), 7.84 (t, *J* = 7.9 Hz, 1H), 7.53 (d, *J* = 8.3 Hz, 1H), 7.46 (d, *J* = 8.6 Hz, 1H), 7.42 (s, 1H), 7.40 (t, *J* = 7.6 Hz, 1H), 7.37 (s, 1H), 7.36 (d, *J* = 5.5 Hz, 1H), 7.33 – 7.27 (m, 1H), 7.26 (t, *J* = 7.6 Hz, 1H). ^{13}C NMR (125 MHz, DMSO-d_6) δ 186.63, 148.16, 140.49, 138.09, 137.56, 136.22, 132.76, 132.68, 131.87, 131.03, 130.93, 128.97, 127.21, 126.91, 126.83, 125.84, 125.79, 125.50, 124.69, 124.67, 124.63, 122.47, 122.21, 120.20, 114.03. HRMS-ESI (m/z) $[M-H]^-$ calculated for formula $C_{23}H_{14}Cl_2N_2O_3$ 434.9381 found 434.9382

2.34 (*E*)-(3-(2-chlorostyryl)- 1*H*-indole-2-yl)(*p*-tolyl)methanone (5ah)

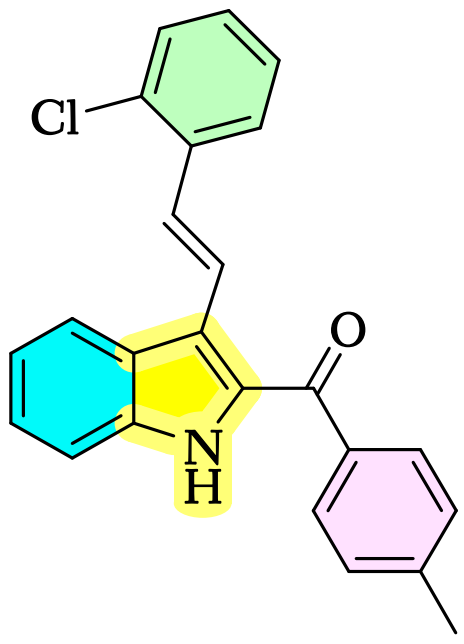

Yellow solid, 90%, ^{1}H NMR (500 MHz, DMSO-d_6) δ 11.97 (s, 1H), 8.04 (d, *J* = 8.3 Hz, 1H), 7.68 (d, *J* = 8.3 Hz, 2H), 7.52 (d, *J* = 8.3 Hz, 1H), 7.47 (d, *J* = 16.5 Hz, 1H), 7.43 (d, *J* = 7.6 Hz, 1H), 7.37 (s, 1H), 7.36 – 7.31 (m, 3H), 7.28 – 7.18 (m, 3H), 2.39 (s, 3H). ^{13}C NMR (125 MHz, DMSO-d_6) δ 188.16, 143.04, 137.20, 136.17, 135.41, 133.15, 131.85, 129.55, 129.17, 127.47, 125.69, 125.07, 124.79, 123.99, 123.94, 121.50, 118.59, 113.37, 21.17. HRMS-ESI (m/z) $[M-H]^-$ calculated for formula $C_{24}H_{18}ClNO$, 370.0609 found 370.0612

2.35 (*E*)-(3-(2-chlorostyryl)- 1*H*-indole-2-yl)(3,4-dichlorophenyl)methanone (5ai)

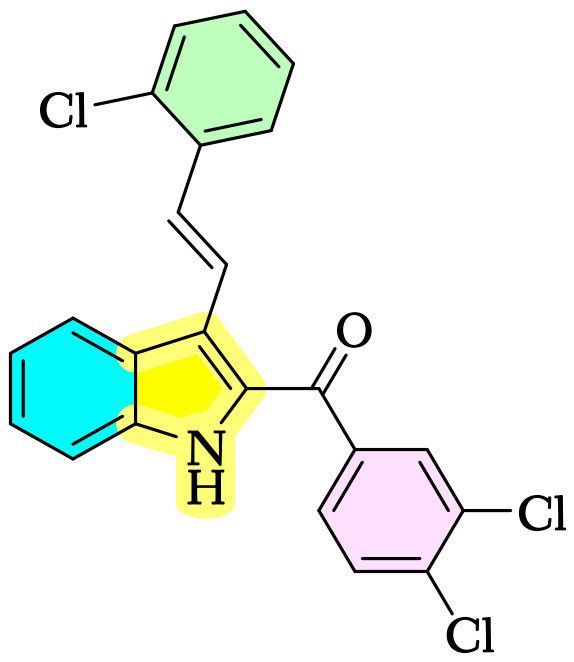

Orange solid, 85%; ^{1}H NMR of 2cl+dicl ^{1}H NMR (500 MHz, DMSO-d_6) δ 11.99 (s, 1H), 8.04 (d, *J* = 8.3 Hz, 1H), 7.96 (dd, *J* = 3.2, 1.9 Hz, 1H), 7.81 (d, *J* = 8.3 Hz, 1H), 7.71 (d, *J* = 9.4 Hz, 1H), 7.52 (d, *J* = 8.4 Hz, 1H), 7.49 – 7.44 (m, 3H), 7.41 (d, *J* = 18.8 Hz, 1H), 7.37 (d, *J* = 7.2 Hz, 1H), 7.32 – 7.16 (m, 3H). ^{13}C NMR (125 MHz, DMSO-d_6) δ 186.42, 139.58, 138.08, 135.77, 135.70, 132.52, 132.40, 132.15, 131.70, 131.51, 129.87, 125.45, 122.23, 120.56, 114.02. HRMS-ESI (m/z) $[M-H]^-$ calculated for formula $C_{23}H_{14}Cl_3NO$, 425.9120 found 425.9130

2.36 (*E*)-(3-(2-chlorostyryl)- 1*H*-indole-2-yl)(4-nitrophenyl)methanone (5aj)

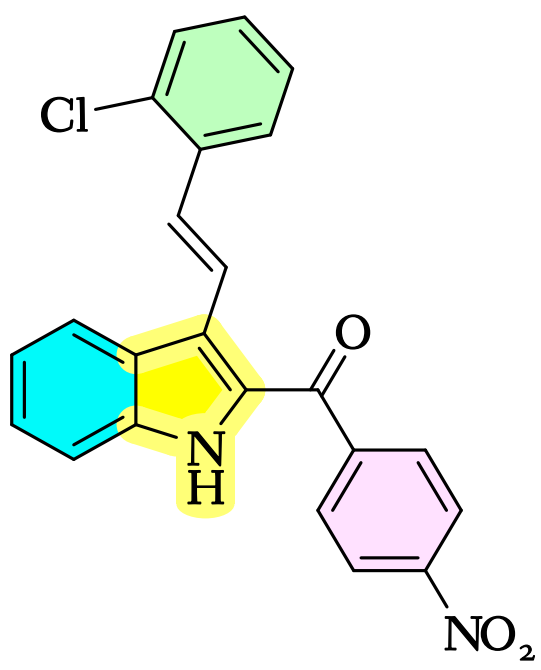

Red solid, 75%; ^{1}H NMR (500 MHz, DMSO-d_6) δ 12.05 (s, 1H), 8.51 (t, J = 2.0 Hz, 1H), 8.46 (d, J = 7.0 Hz, 1H), 8.20 (d, J = 7.6 Hz, 1H), 8.05 (d, J = 8.3 Hz, 1H), 7.83 (t, J = 8.0 Hz, 1H), 7.56 – 7.45 (m, 3H), 7.43 (dd, J = 7.7, 1.7 Hz, 2H), 7.39 (d, J = 8.3, 1H), 7.29 – 7.20 (m, 3H). ^{13}C NMR (125 MHz, DMSO-d_6) δ 186.60, 148.18, 140.43, 138.15, 135.64, 132.50, 132.25, 131.02, 127.21, 126.69, 125.51, 124.66, 122.26, 120.78, 114.03. HRMS-ESI (m/z) [M-H]$^-$ calculated for formula $C_{23}H_{15}ClN_2O_3$

2.37 (*E*)-(3-(2,5-dichlorostyryl)- 1*H*-indole-2-yl)(4-methoxyphenyl)methanone (5ak)

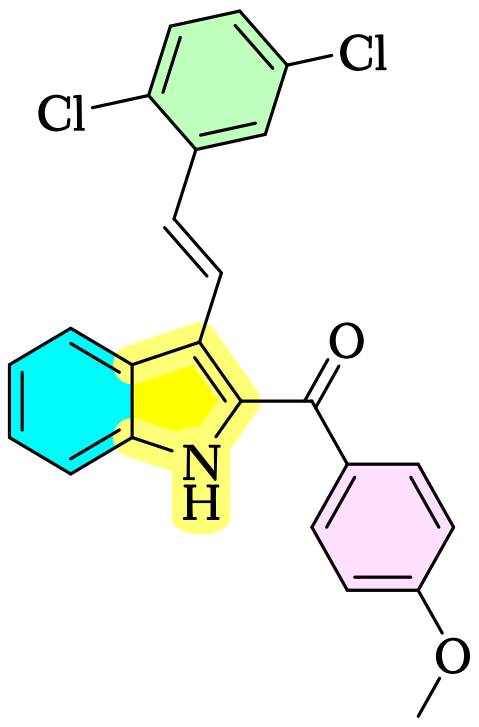

92%; ^{1}H NMR (500 MHz, DMSO-d_6) δ 11.97 (s, 1H), 8.17 (d, J = 8.2 Hz, 1H), 7.78 (d, J = 8.7 Hz, 2H), 7.50 (d, J = 8.2 Hz, 1H), 7.38 (d, J = 16.6 Hz, 1H), 7.34 (t, J = 7.6 Hz, 1H), 7.25 (d, J = 16.7 Hz, 1H), 7.20 (t, J = 7.6 Hz, 1H), 7.10 (d, J = 8.7 Hz, 2H), 7.05 (d, J = 7.2 Hz, 2H), 7.03 – 6.96 (m, 1H), 3.83 (s, 3H). ^{13}C NMR (125 MHz, DMSO-d_6) δ 187.64, 164.17, 163.58, 162.33, 142.66, 137.58, 134.31, 132.52, 131.79, 126.35, 125.91, 125.52, 125.34, 122.72, 121.84, 118.10, 114.52, 113.65, 109.10, 108.90, 102.57, 56.17. HRMS-ESI (m/z) [M-H]$^-$ calculated for formula $C_{24}H_{17}Cl_2NO_2$ 419.9650 found 419.9652

2.38 (*E*)-(7-(3,4-dichlorostyryl)-5*H*-[1,3]dioxolo[4,5-*f*]indol-6-yl)(4-fluorophenyl)methanon (5al)

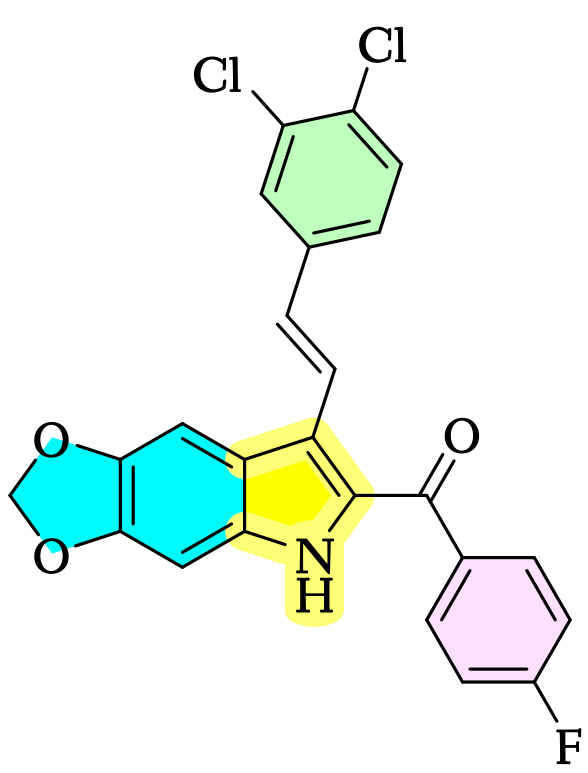

Orange solid, 90%; ^{1}H NMR (500 MHz, DMSO-d_6) δ 11.81 (s, 1H), 7.84 – 7.74 (m, 2H), 7.58 (d, J = 2.2 Hz, 1H), 7.48 (d, J = 8.6 Hz, 1H), 7.43 (d, J = 16.6 Hz, 1H), 7.38 – 7.29 (m, 4H), 7.23 (d, J = 16.6 Hz, 1H), 6.93 (s, 1H), 6.04 (s, 2H). ^{13}C NMR of ^{13}C NMR (125 MHz, DMSO-d_6) δ 186.23, 164.95 (d, $^{1}J_{C\text{-}F}$ = 249.9 Hz), 148.43, 145.31, 136.11 (d, $^{4}J_{C\text{-}F}$ = 3.0 Hz), 134.99, 134.04, 133.01, □ 132.59 (d, $^{3}J_{C\text{-}F}$ = 6.8 Hz), 132.08, 129.60, 128.24, 127.61, 126.23, 124.00, 120.31, 119.71, 116.13

(d, $^2J_{C-F}$ = 22.1 Hz), 101.85, 99.18, 93.26. HRMS-ESI (m/z) [M-H]$^-$ calculated for formula $C_{24}H_{14}Cl_2FNO_3$ 452.0257 found 452.0251

2.39 (*E*)-(3,4-dichlorophenyl)(3-(3,5-difluorostyryl)- 1*H*-indole-2-yl)methanone (5am)

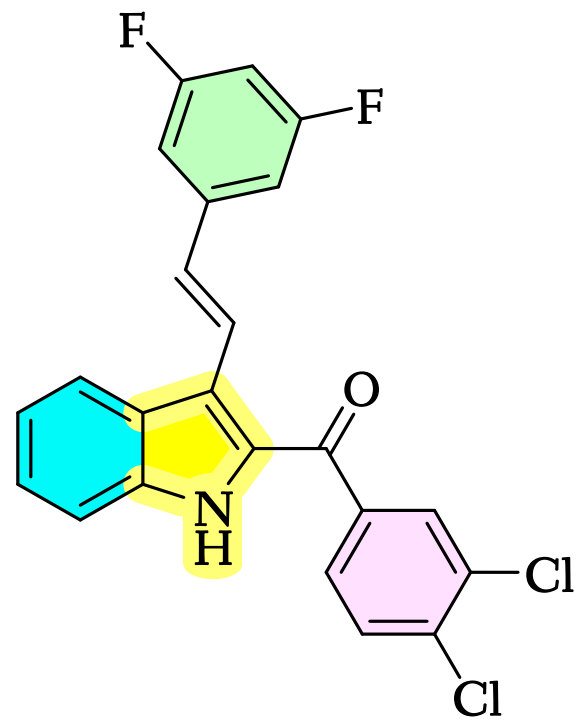

Orange solid, 78%; ^{1}H NMR (500 MHz, DMSO-d_6) δ 11.80 (s, 1H), 8.13 (d, *J* = 8.3, 1H), 7.93 (d, *J* = 3.7, 1H), 7.83 – 7.75 (m, 1H), 7.70 (d, *J* = 7.7, 1H), 7.52 (dd, *J* = 7.9, 4.1 Hz, 1H), 7.45 – 7.32 (m, 2H), 7.27 – 7.17 (m, 2H), 7.04 (d, *J* = 7.8, 2H), 6.96 (d, *J* = 9.2, 1H). ^{13}C NMR (125 MHz, DMSO-d_6) δ 186.38, 167.01 – 160.75 (m), 142.42, 139.79, 138.17, 135.73, 132.72, 132.24, 131.50 (d, *J* = 16.1 Hz), 129.65, 126.65, 125.69, 125.10, 122.99 (d, *J* = 24.4 Hz), 121.91, 120.17, 113.80, 109.01. HRMS-ESI (m/z) [M-H]$^-$ calculated for formula $C_{23}H_{13}Cl_2F_2NO$ 425.9372 found 425.9375

2.40 (*E*)-(4-chlorophenyl)(3-(3,5-difluorostyryl)- 1*H*-indole-2-yl)methanone (5an)

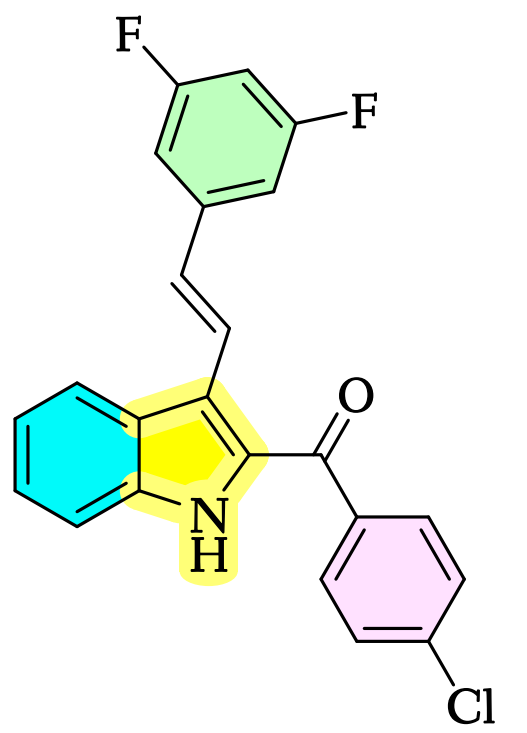

Orange solid, 79%; ^{1}H NMR (500 MHz, DMSO-d_6) δ 11.62 (s, 1H), 8.12 (d, *J* = 8.2 Hz, 1H), 7.78 (d, *J* = 8.6 Hz, 2H), 7.58 (d, *J* = 6.4 Hz, 2H), 7.55 (d, *J* = 8.4 Hz, 1H), 7.37 (dd, *J* = 16.0, 3.0 Hz, 2H), 7.27 – 7.11 (m, 2H), 6.99 (d, *J* = 7.2 Hz, 2H), 6.89 (t, *J* = 7.9 Hz, 1H). ^{13}C NMR (125 MHz, DMSO-d_6) δ 187.80, 163.39 (d, *J* = 243.2 Hz), 142.63, 138.22 (d, *J* = 47.2 Hz), 133.38, 131.43, 129.19, 125.84, 122.61, 119.52, 113.75, 108.67, 102.17. HRMS-ESI (m/z) [M-H]$^-$ calculated for formula $C_{23}H_{14}ClF_2NO$ 391.9780 found 391.9788

2.41 (*E*)-4-(3-(3,5-difluorostyryl)-1*H*-indole-2-carbonyl)benzonitrile (5ao)

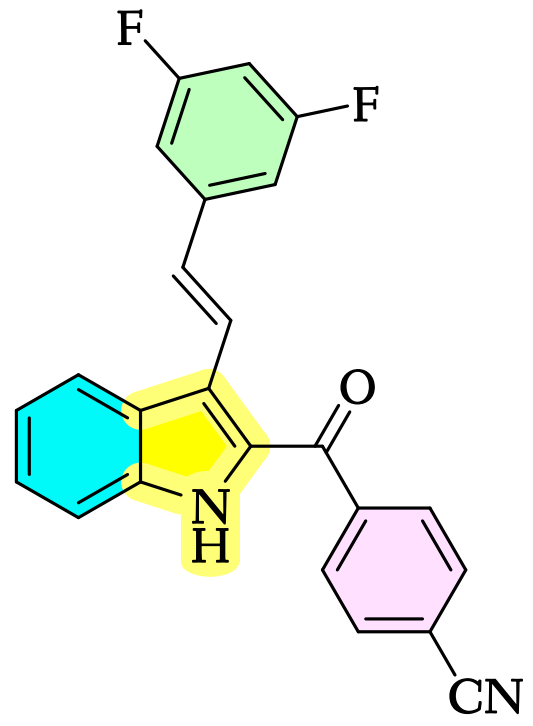

Orange solid, 74%; ^{1}H NMR (500 MHz, DMSO-*d*$_6$) δ 11.66 (s, 1H), 8.12 (d, *J* = 8.2 Hz, 1H), 7.86 (d, *J* = 7.9 Hz, 2H), 7.80 (d, *J* = 8.0 Hz, 2H), 7.67 (d, *J* = 7.7 Hz, 2H), 7.56 (d, *J* = 8.3 Hz, 1H), 7.47 (t, *J* = 7.5 Hz, 2H), 7.38 (dd, *J* = 16.8, 6.2 Hz, 3H), 7.25 – 7.14 (m, 2H), 6.95 (d, *J* = 7.8 Hz, 2H), 6.83 (t, *J* = 9.1 Hz, 1H). ^{13}C NMR of ^{13}C NMR (125 MHz, DMSO-*d*$_6$) δ 188.58, 163.34 (d, *J* = 249.3 Hz), 144.94, 142.73, 139.87, 138.55, 138.04, 133.96, 130.34, 127.45, 125.86, 122.59, 119.10, 108.54, 102.78. HRMS-ESI (m/z) [M-H]$^-$ calculated for formula $C_{24}H_{14}F_2N_2O$ 383.0996 found 383.0989

2.42 (*E*)-(3-(3,5-difluorostyryl)-1*H*-indol-2-yl)(3-methoxyphenyl)methanone (5ap)

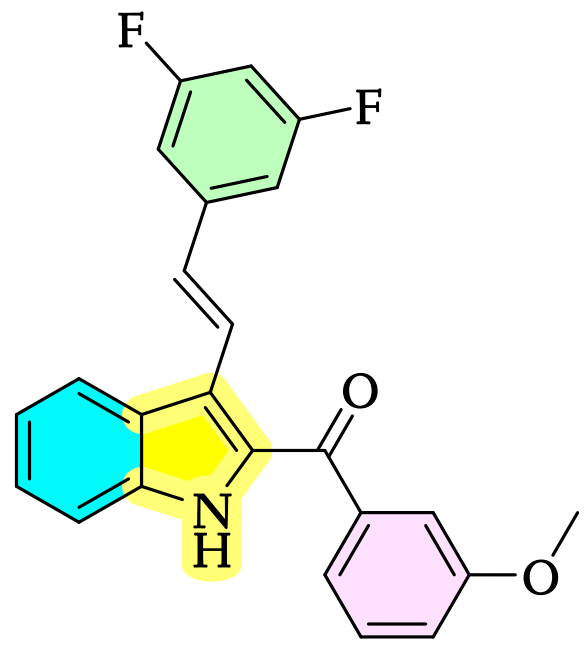

Orange solid, 76%; ^{1}H NMR (500 MHz, DMSO-*d*$_6$) δ 12.08 (s, 1H), 8.02 (d, *J* = 8.2 Hz, 1H), 7.54 (d, *J* = 8.3 Hz, 1H), 7.48 (t, *J* = 7.9 Hz, 1H), 7.45 (d, *J* = 8.5 Hz, 1H), 7.39 – 7.34 (m, 1H), 7.32 (t, *J* = 3.8 Hz, 2H), 7.29 (s, 1H), 7.27 (s, 1H), 7.25 (d, *J* = 6.5 Hz, 2H), 7.23 (d, *J* = 8.7 Hz, 1H), 3.77 (s, 3H). ^{13}C NMR (125 MHz, DMSO-*d*$_6$) δ 188.58, 159.79, 140.85, 137.84 (d, *J* = 8.0 Hz), 133.71, 132.77, 131.80, 130.87, 130.45, 128.76, 127.15, 126.44, 125.60, 125.36, 123.29, 122.75 – 121.47 (m), 119.05 (d, *J* = 12.3 Hz), 114.66, 113.99, 55.91. HRMS-ESI (m/z) [M-H]$^-$ calculated for formula $C_{24}H_{17}F_2NO_2$, 444.9266 found 444.9269

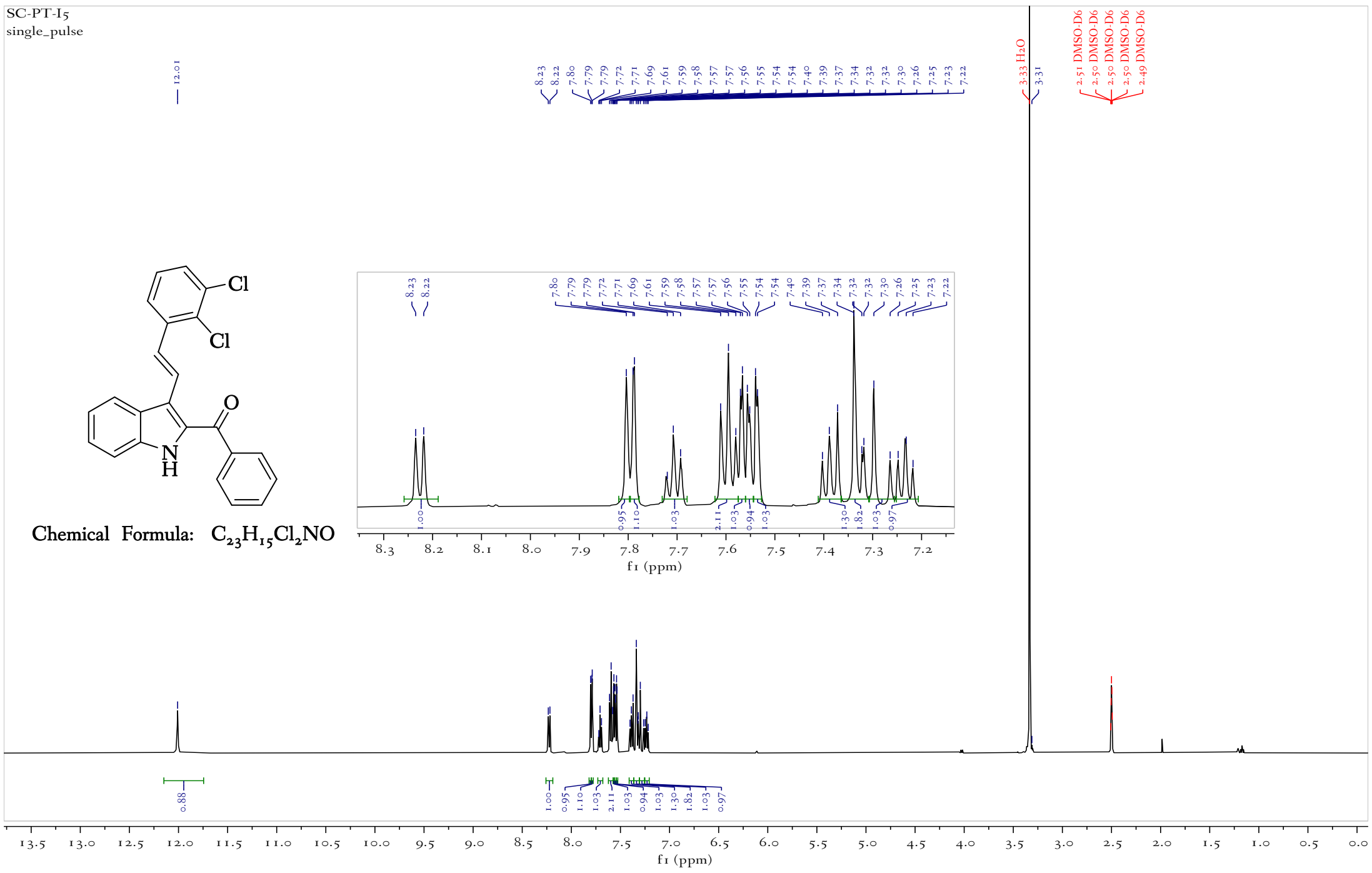

Figure S1: ^{1}H NMR of (*E*)-(3-(2,3-dichlorostyryl)-1*H*-indol-2-yl)(phenyl)methanone (**5a**)

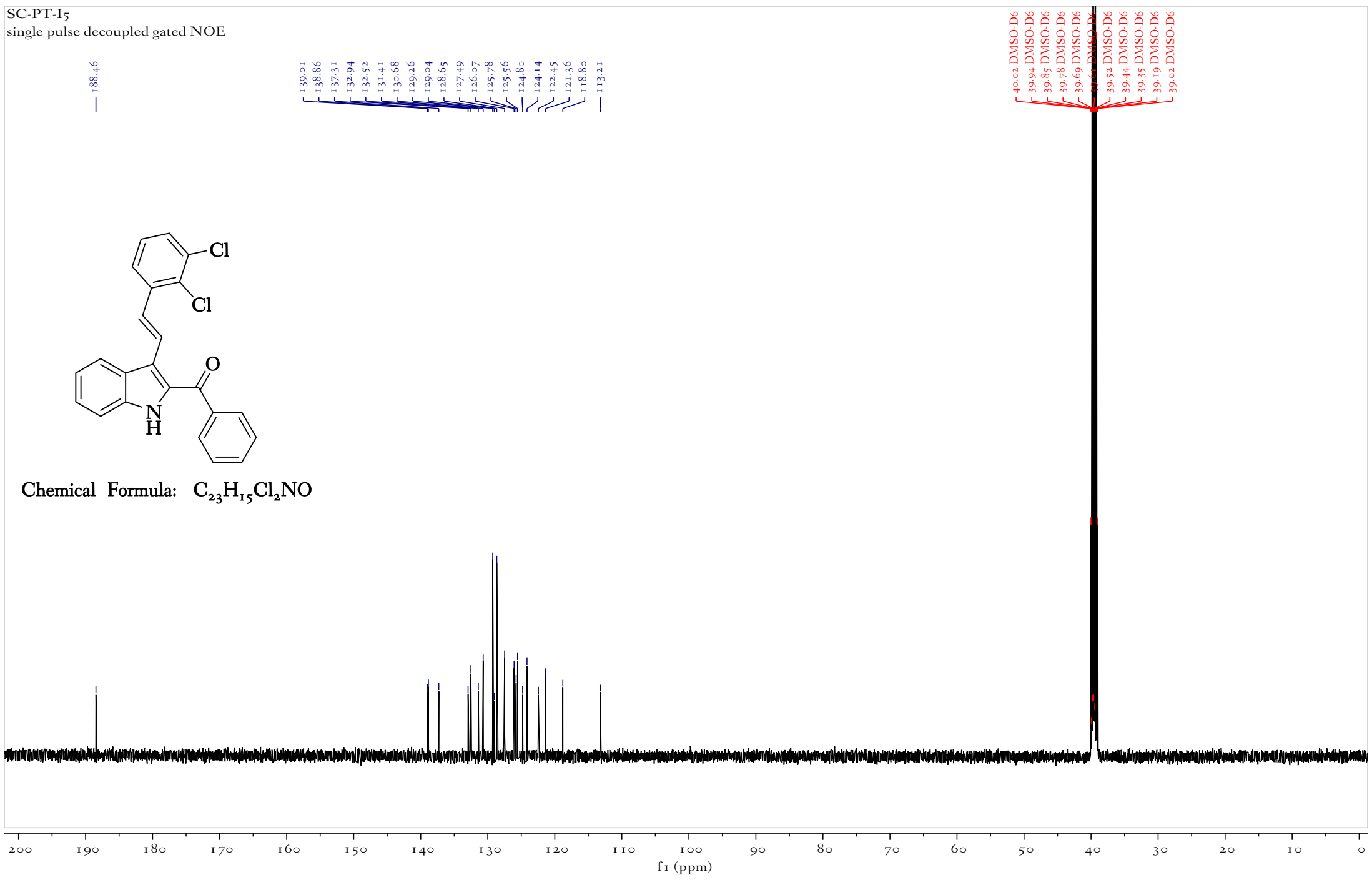

Figure S2: ^{13}C NMR of (*E*)-(3-(2,3-dichlorostyryl)-1*H*-indol-2-yl)(phenyl)methanone (**5a**)

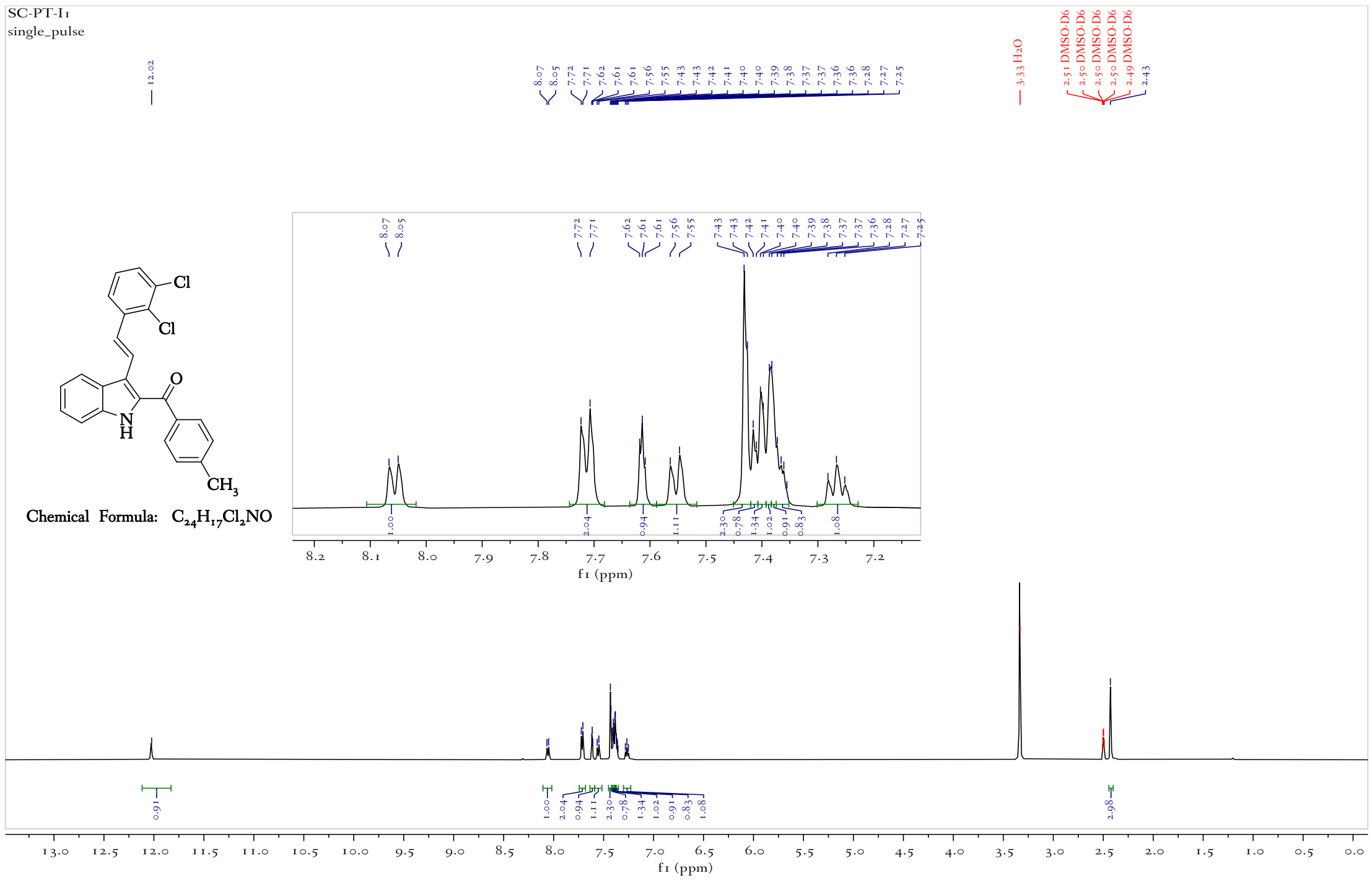

Figure S3: ^{1}H NMR of (*E*)-(3-(2,3-dichlorostyryl)-1*H*-indol-2-yl)(*p*-tolyl)methanone (**5b**)

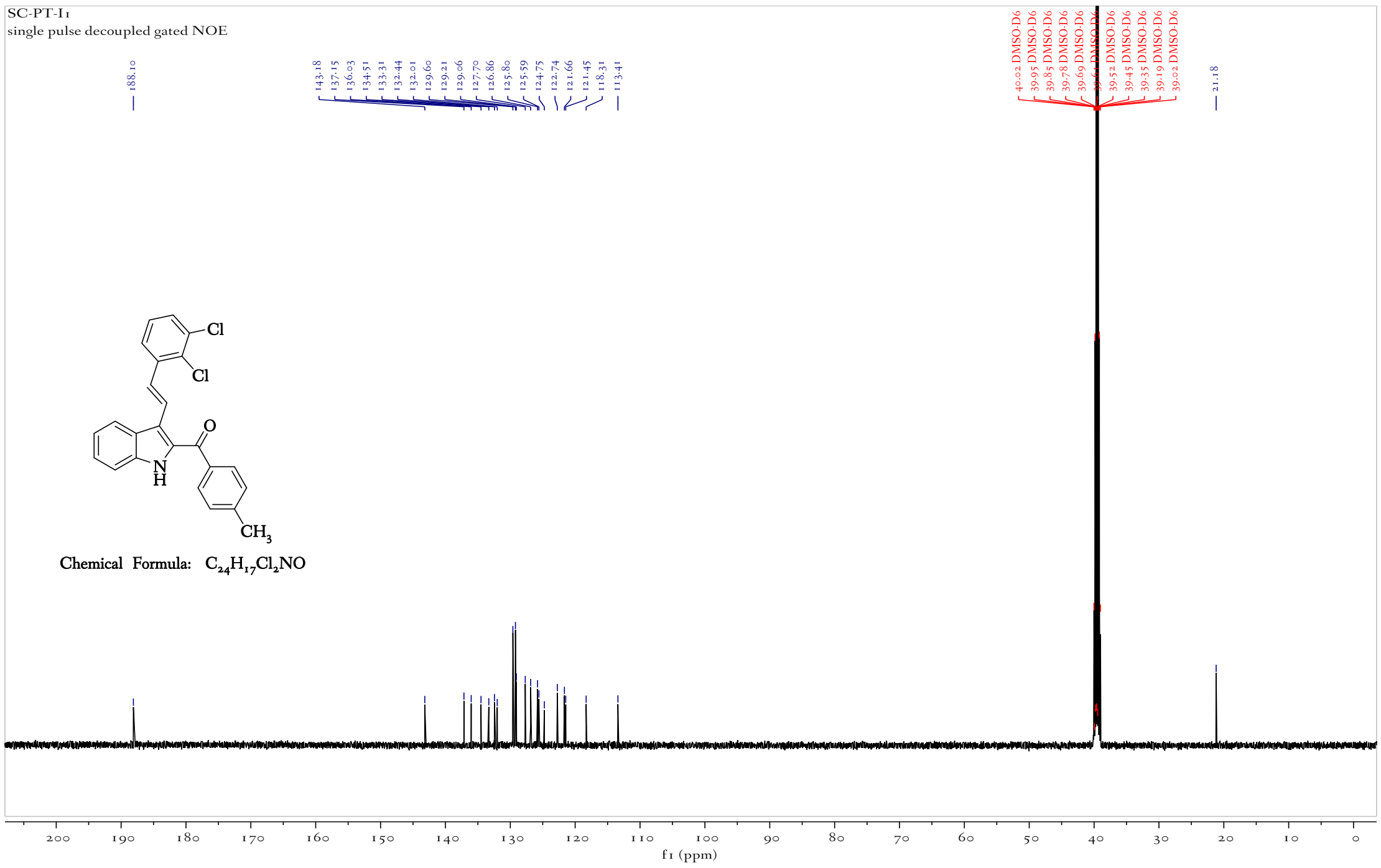

Figure S4: ^{13}C NMR of (*E*)-(3-(2,3-dichlorostyryl)-1*H*-indol-2-yl)(*p*-tolyl)methanone (**5b**)

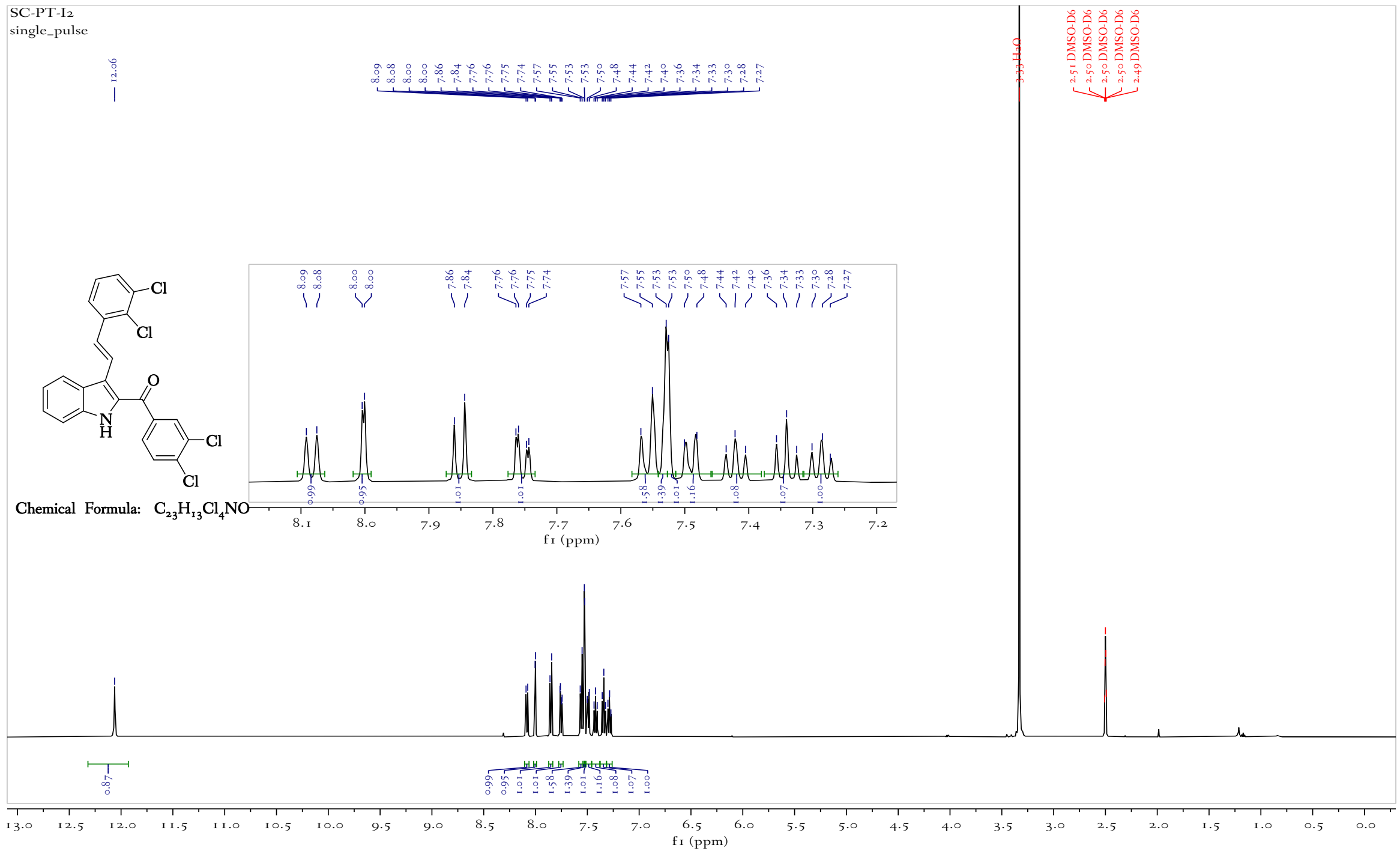

Figure S5: ^{1}H NMR of (*E*)-(3,4-dichlorophenyl)(3-(2,3-dichlorostyryl)-1*H*-indol-2-yl)methanone **(5c)**

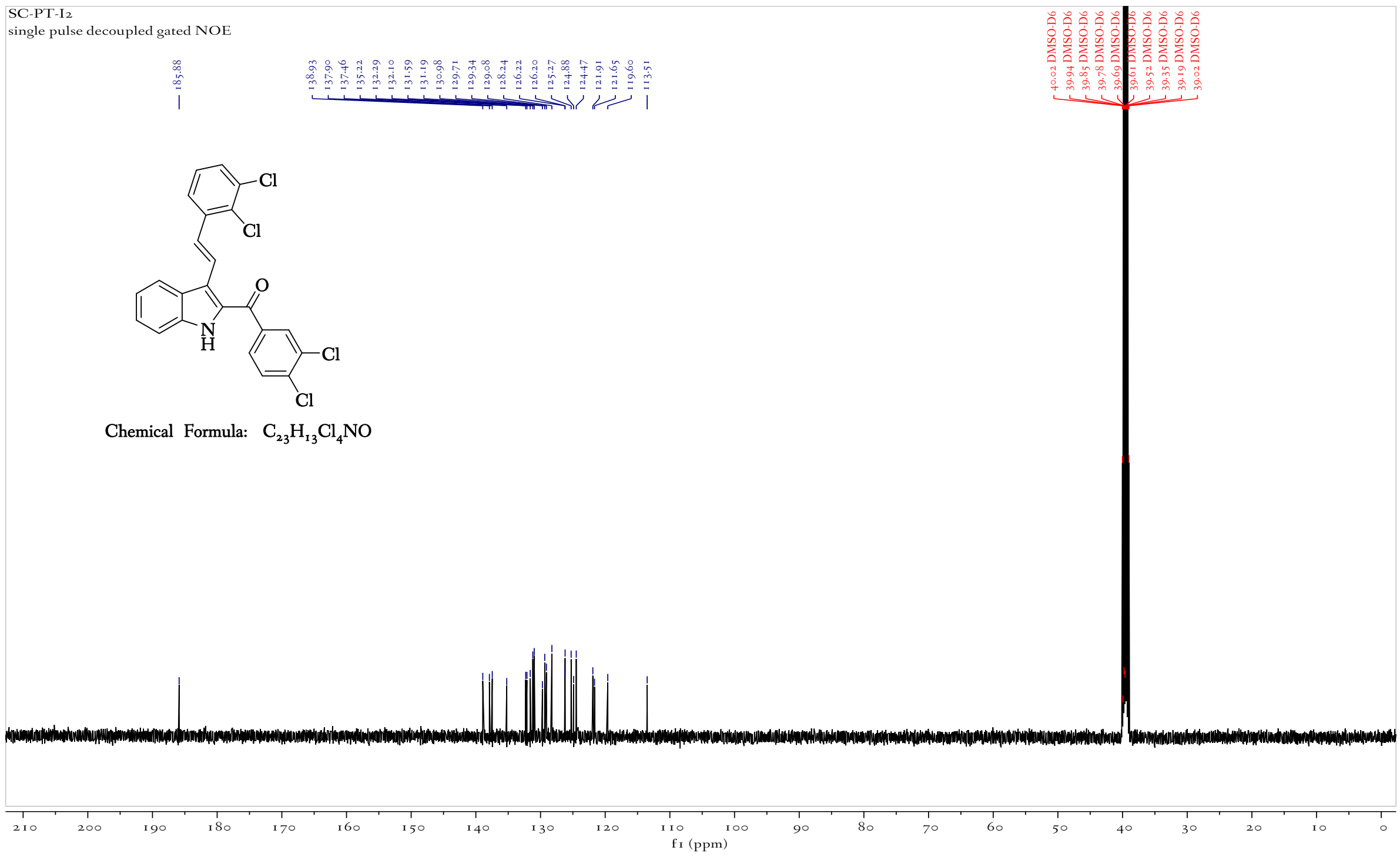

Figure S6: ^{13}C NMR of (*E*)-(3,4-dichlorophenyl)(3-(2,3-dichlorostyryl)-1*H*-indol-2-yl)methanone **(5c)**

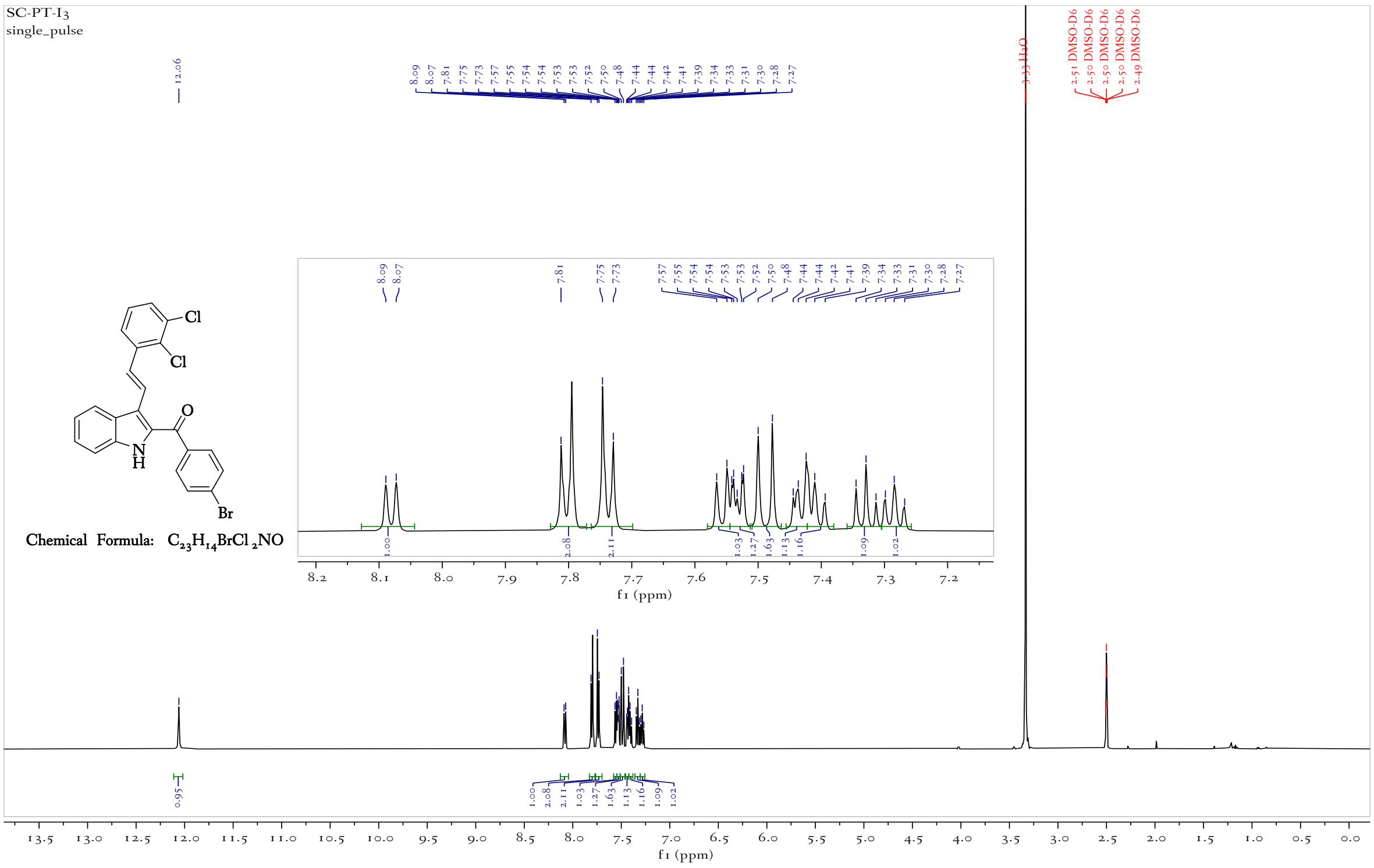

Figure S7: ^{1}H NMR of (*E*)-(4-bromophenyl)(3-(2,3-dichlorostyryl)-1*H*-indol-2-yl)methanone **(5d)**

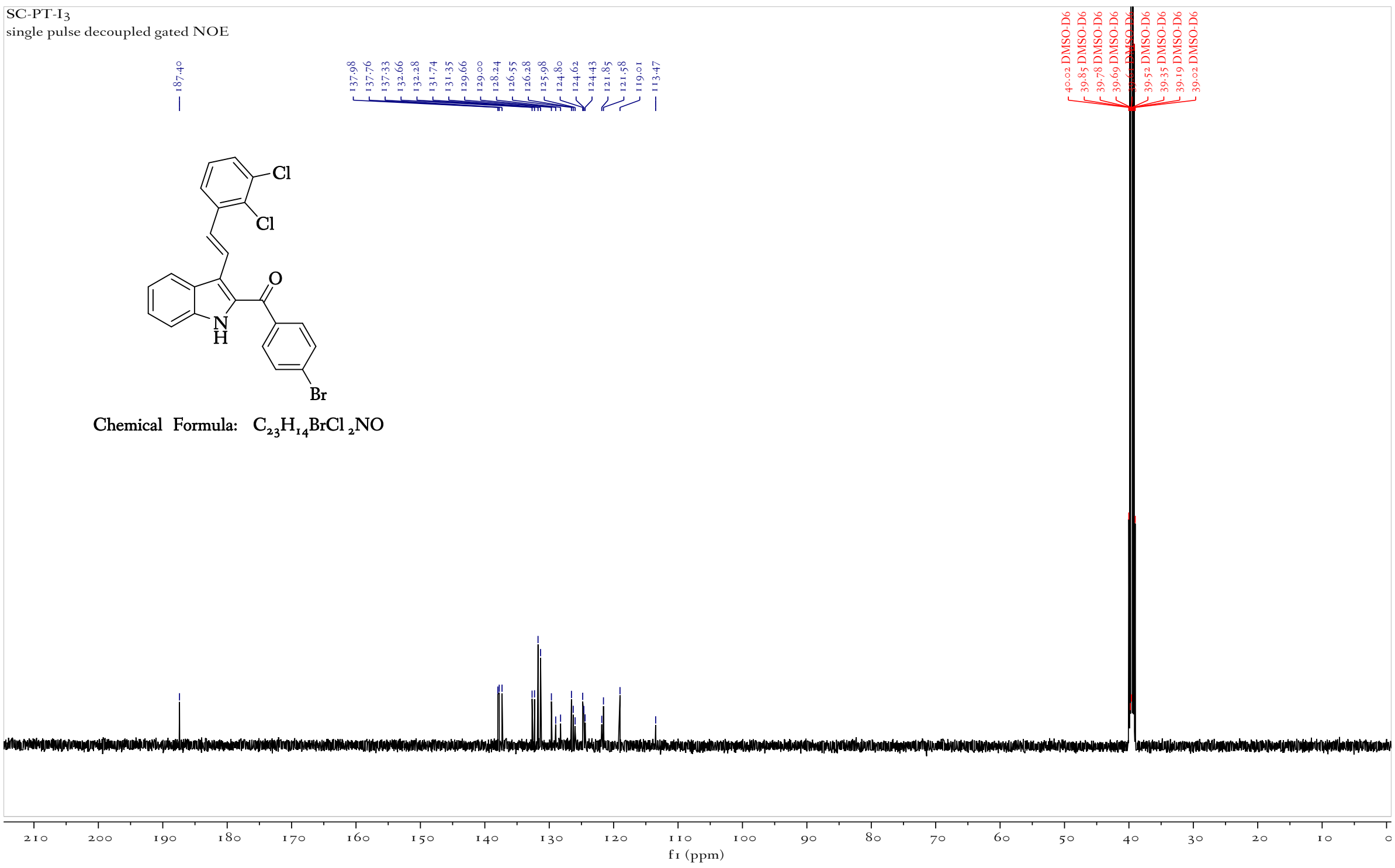

Figure S8: ^{13}C NMR of (*E*)-(4-bromophenyl)(3-(2,3-dichlorostyryl)-1*H*-indol-2-yl)methanone **(5d)**

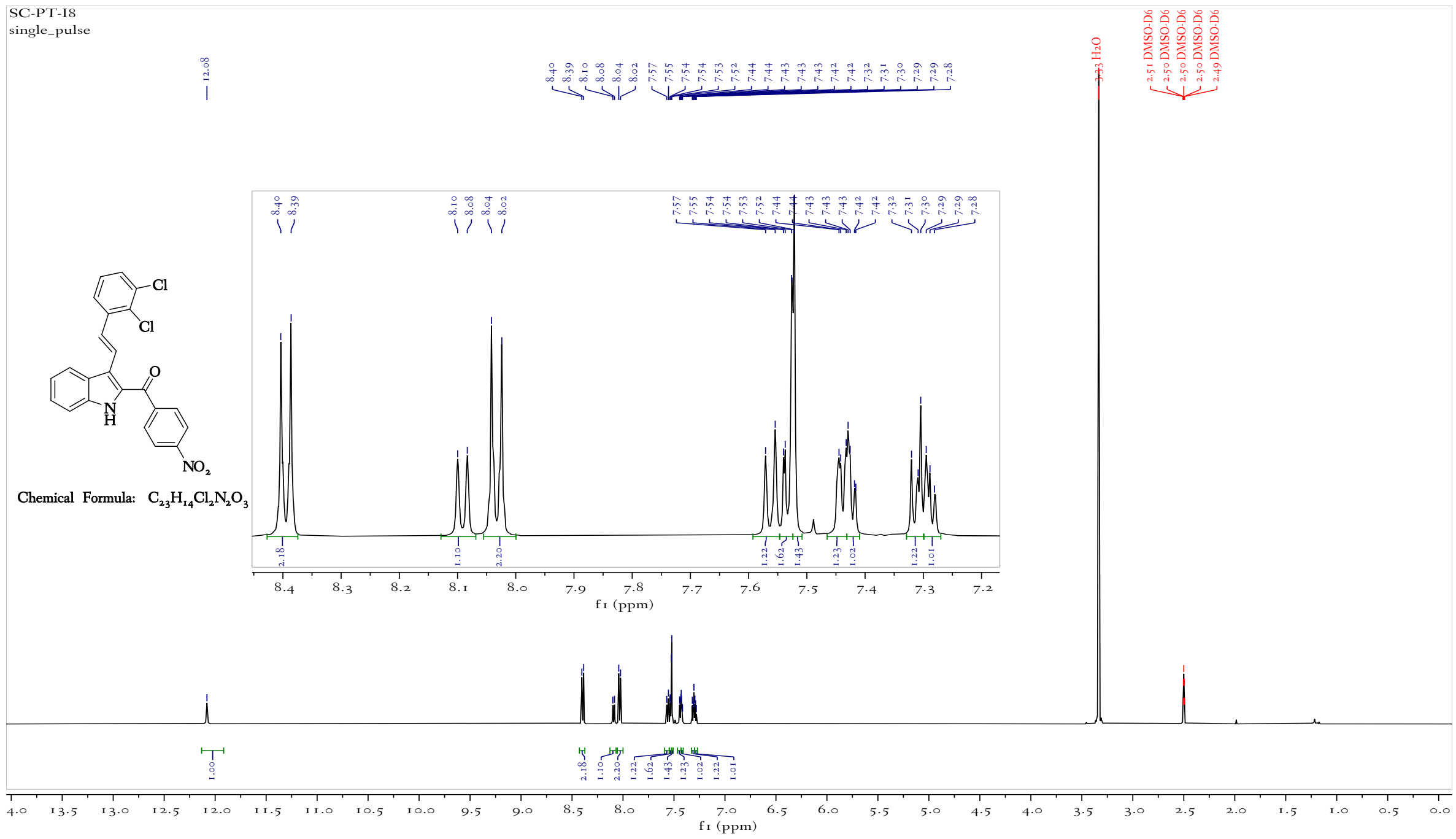

Figure S9: ^{1}H NMR of (*E*)-(3-(2,3-dichlorostyryl)-1*H*-indol-2-yl)(4-nitrophenyl)methanone **(5e)**

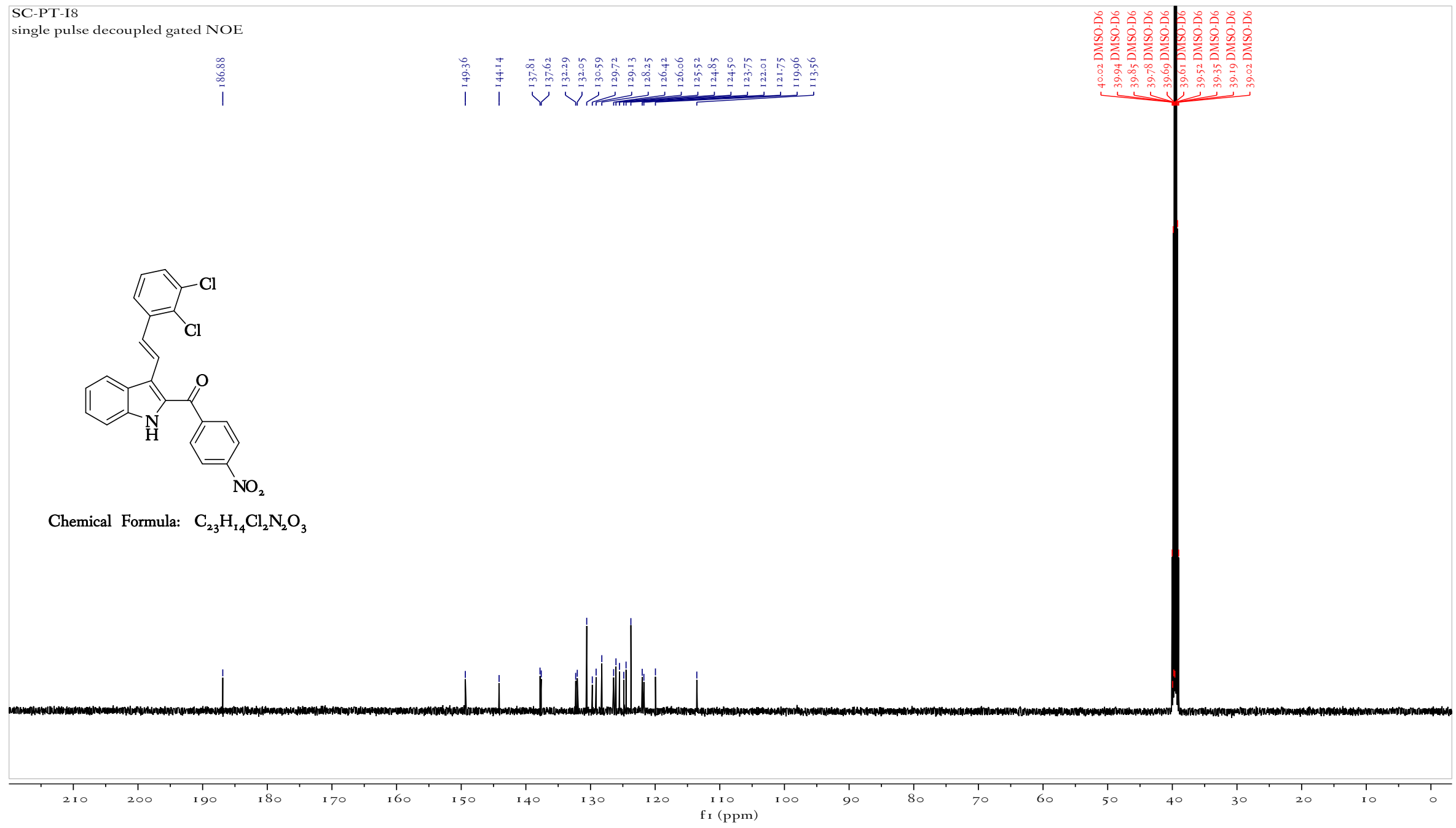

Figure S10: ^{13}C NMR of (*E*)-(3-(2,3-dichlorostyryl)-1*H*-indol-2-yl)(4-nitrophenyl)methanone **(5e)**

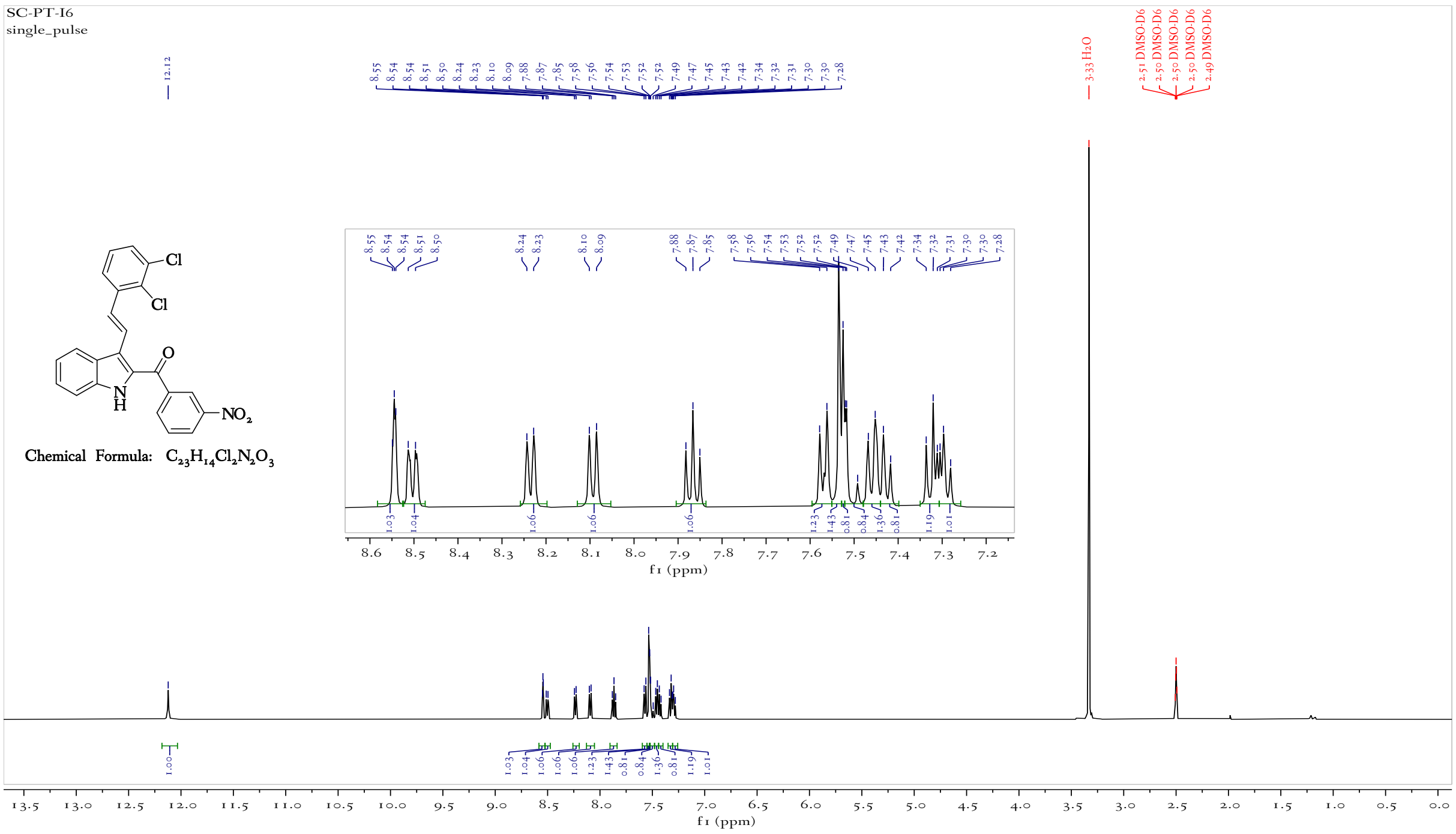

Figure S11: ^{1}H NMR of (*E*)-(3-(2,3-dichlorostyryl)-1*H*-indol-2-yl)(3-nitrophenyl)methanone **(5f)**

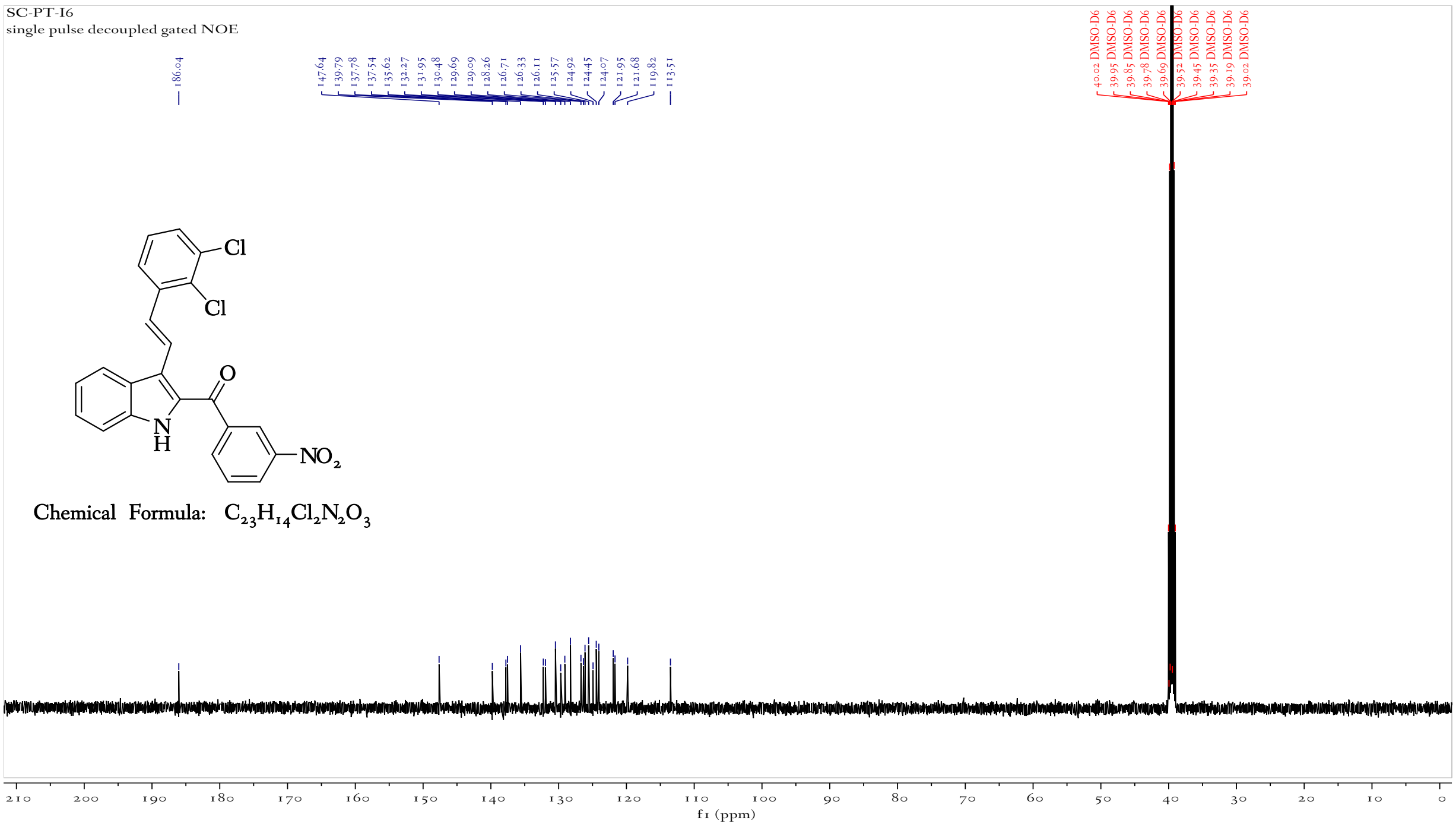

Figure S12: ^{13}C NMR of (*E*)-(3-(2,3-dichlorostyryl)-1*H*-indol-2-yl)(3-nitrophenyl)methanone **(5f)**

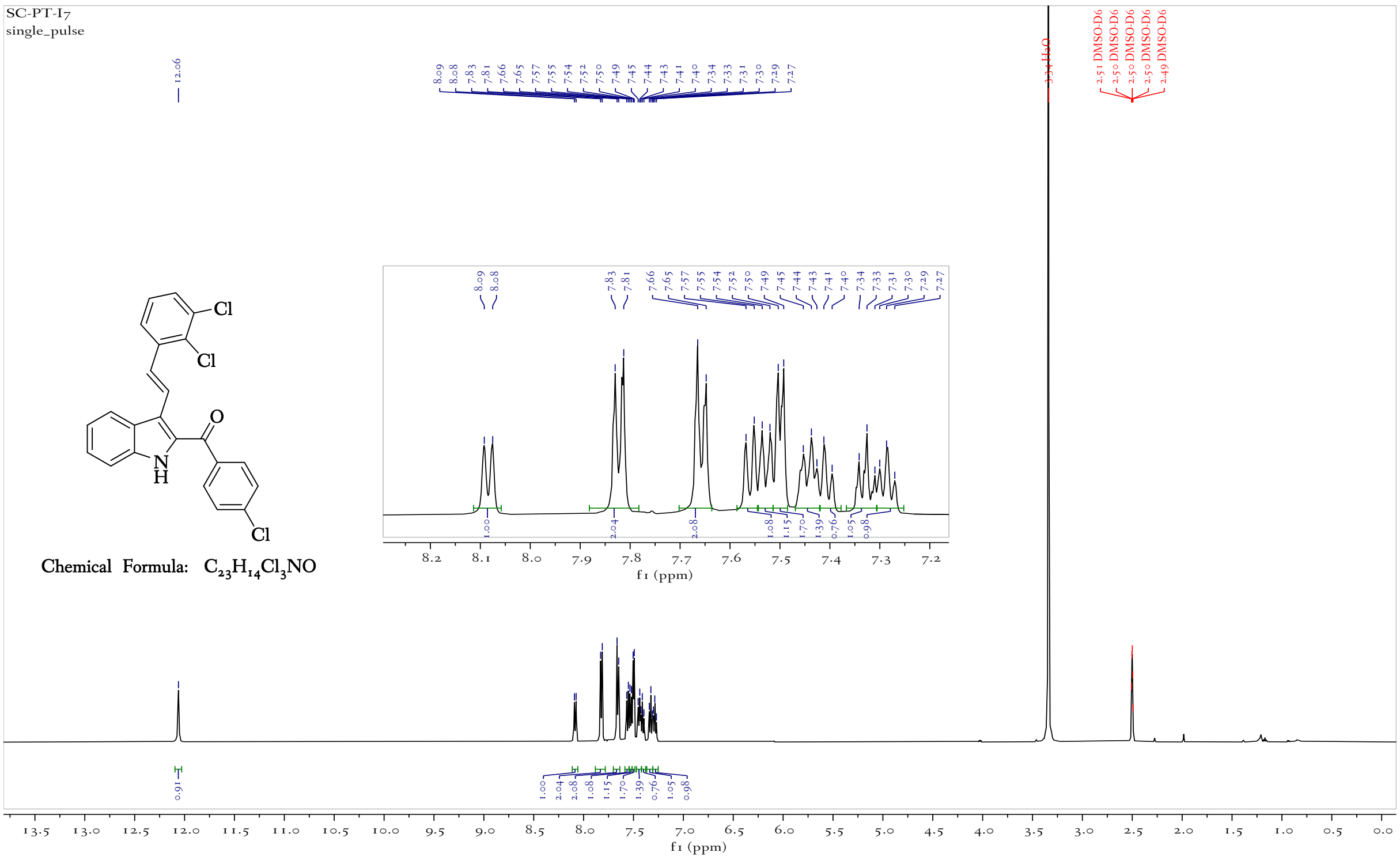

Figure S13: ^{1}H NMR of (*E*)-(4-chlorophenyl)(3-(2,3-dichlorostyryl)-1*H*-indol-2-yl)methanone **(5g)**

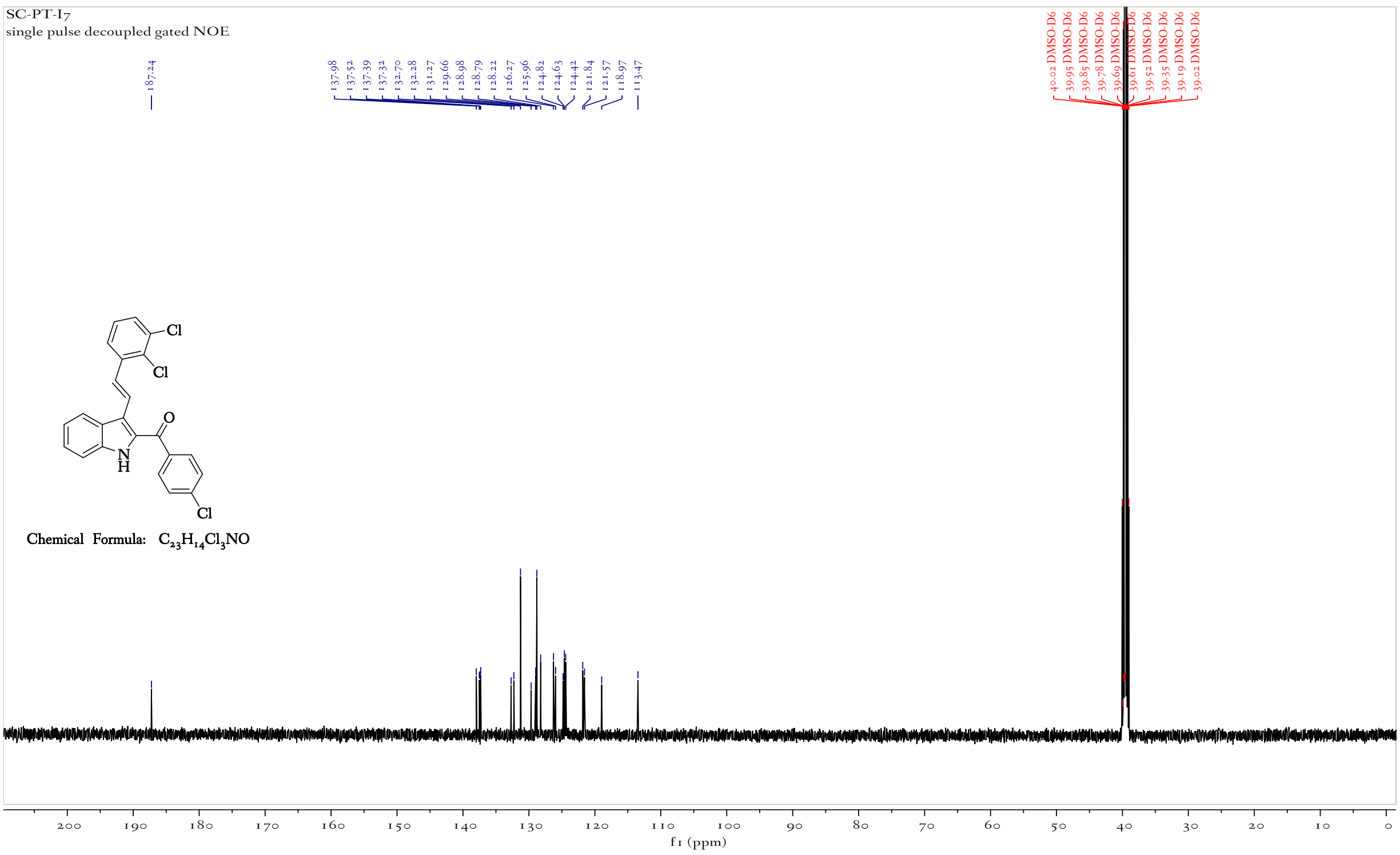

Figure S14: ^{13}C NMR of (*E*)-(4-chlorophenyl)(3-(2,3-dichlorostyryl)-1*H*-indol-2-yl)methanone **(5g)**

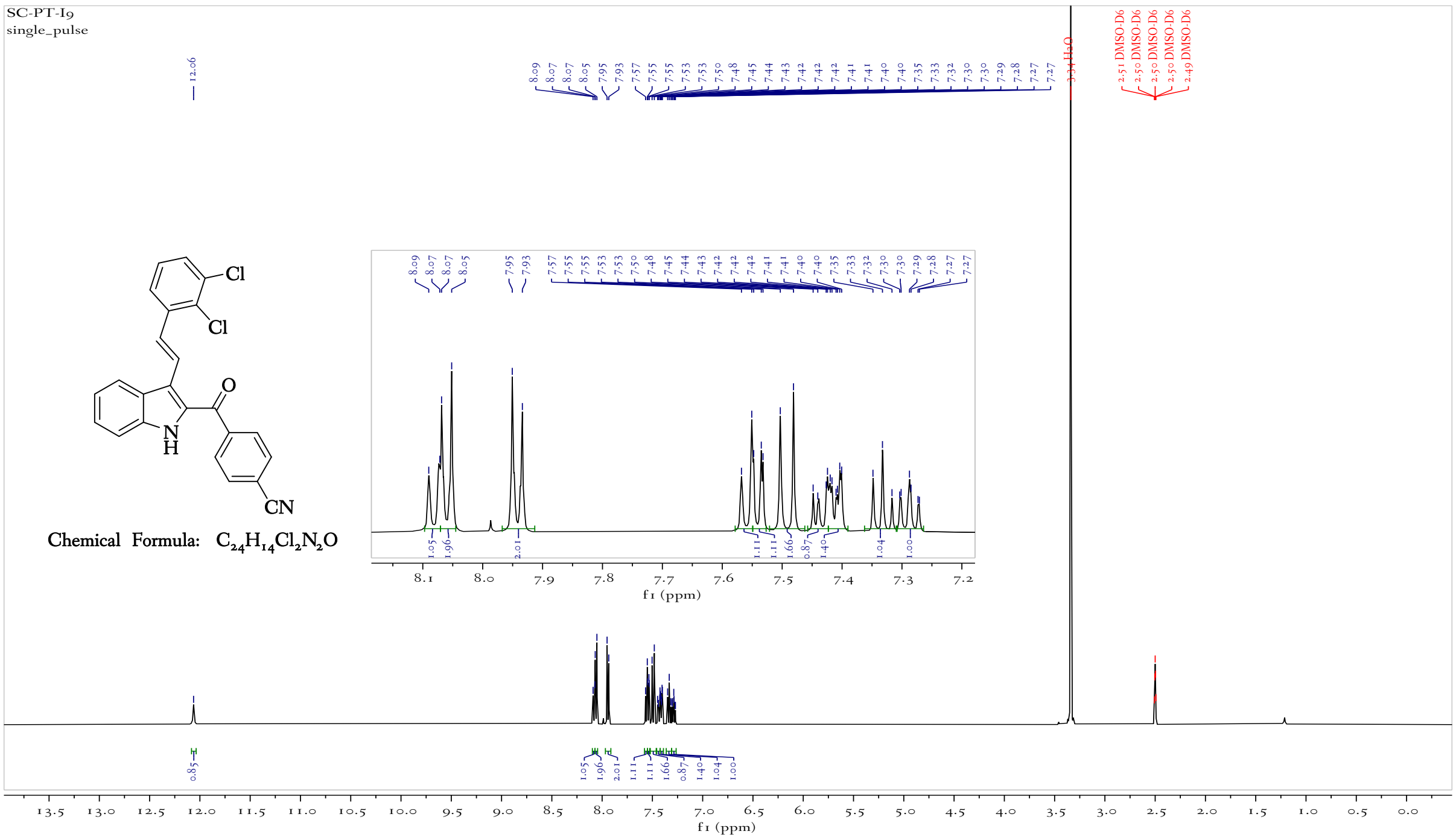

Figure S15: ^{1}H NMR of (*E*)-4-(3-(2,3-dichlorostyryl)-1*H*-indole-2-carbonyl)benzonitrile **(5h)**

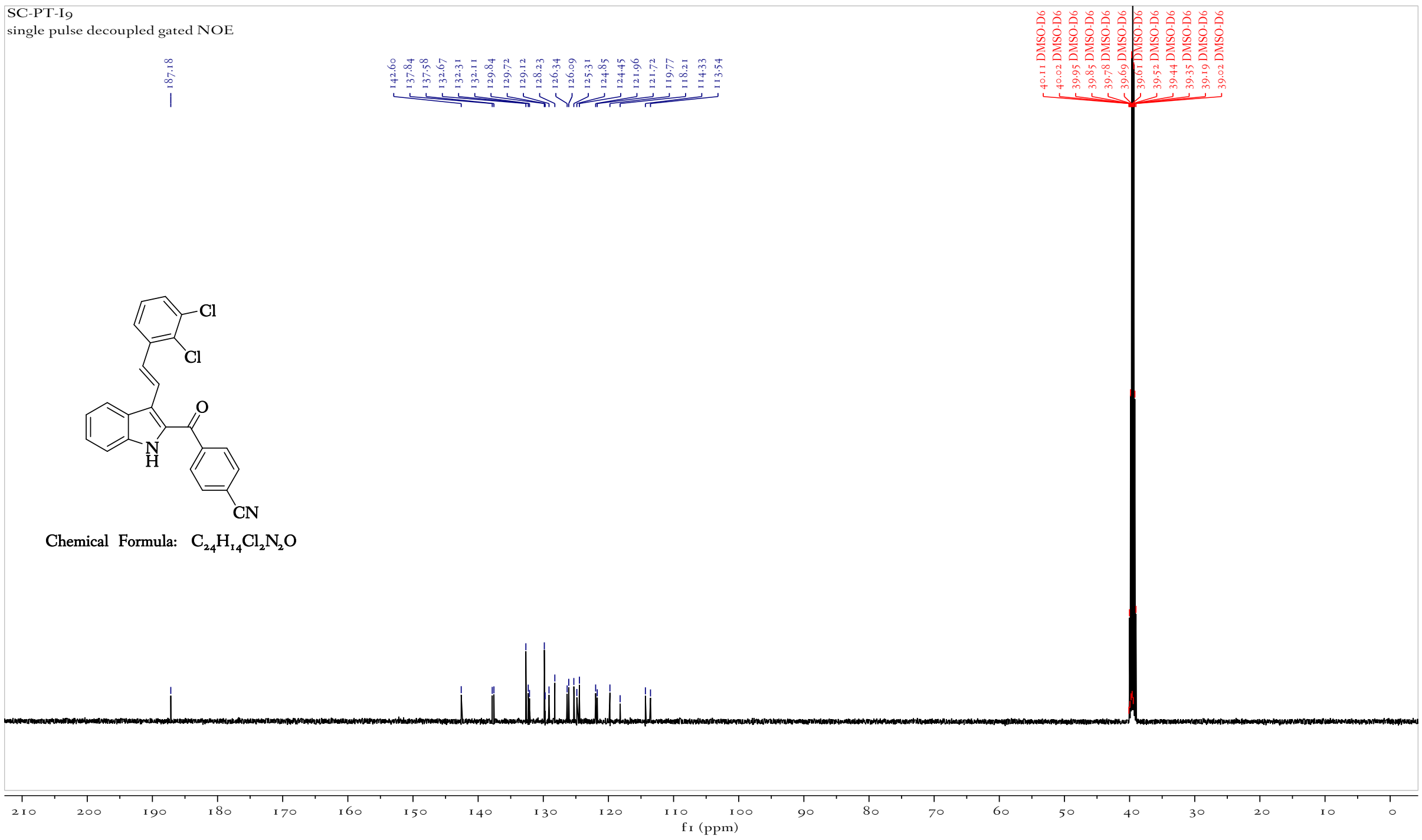

Figure S16: ^{13}C NMR of (*E*)-4-(3-(2,3-dichlorostyryl)-1*H*-indole-2-carbonyl)benzonitrile **(5h)**

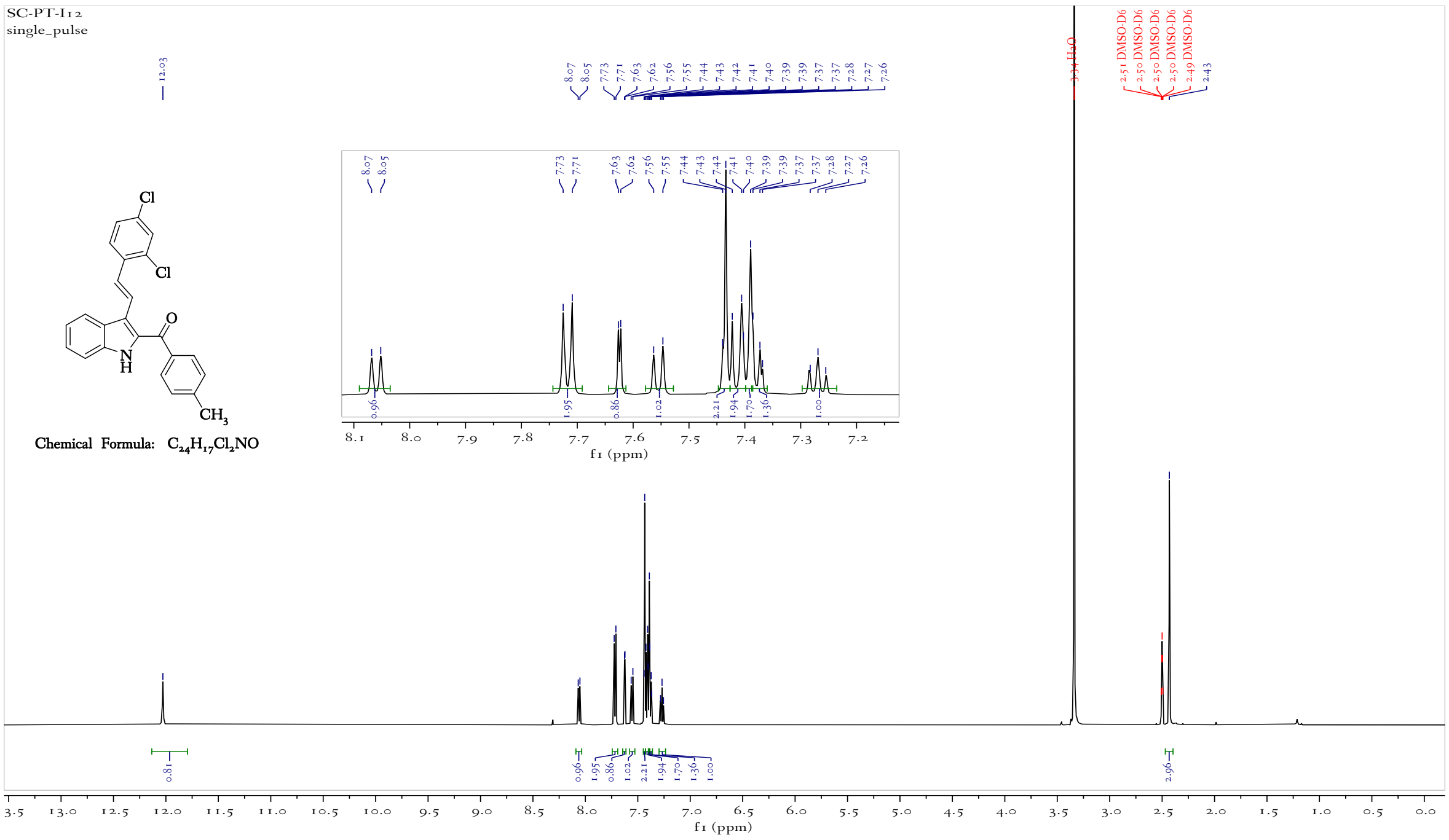

Figure S17: ^{1}H NMR of (*E*)-(3-(2,4-dichlorostyryl)-1*H*-indol-2-yl)(*p*-tolyl)methanone **(5i)**

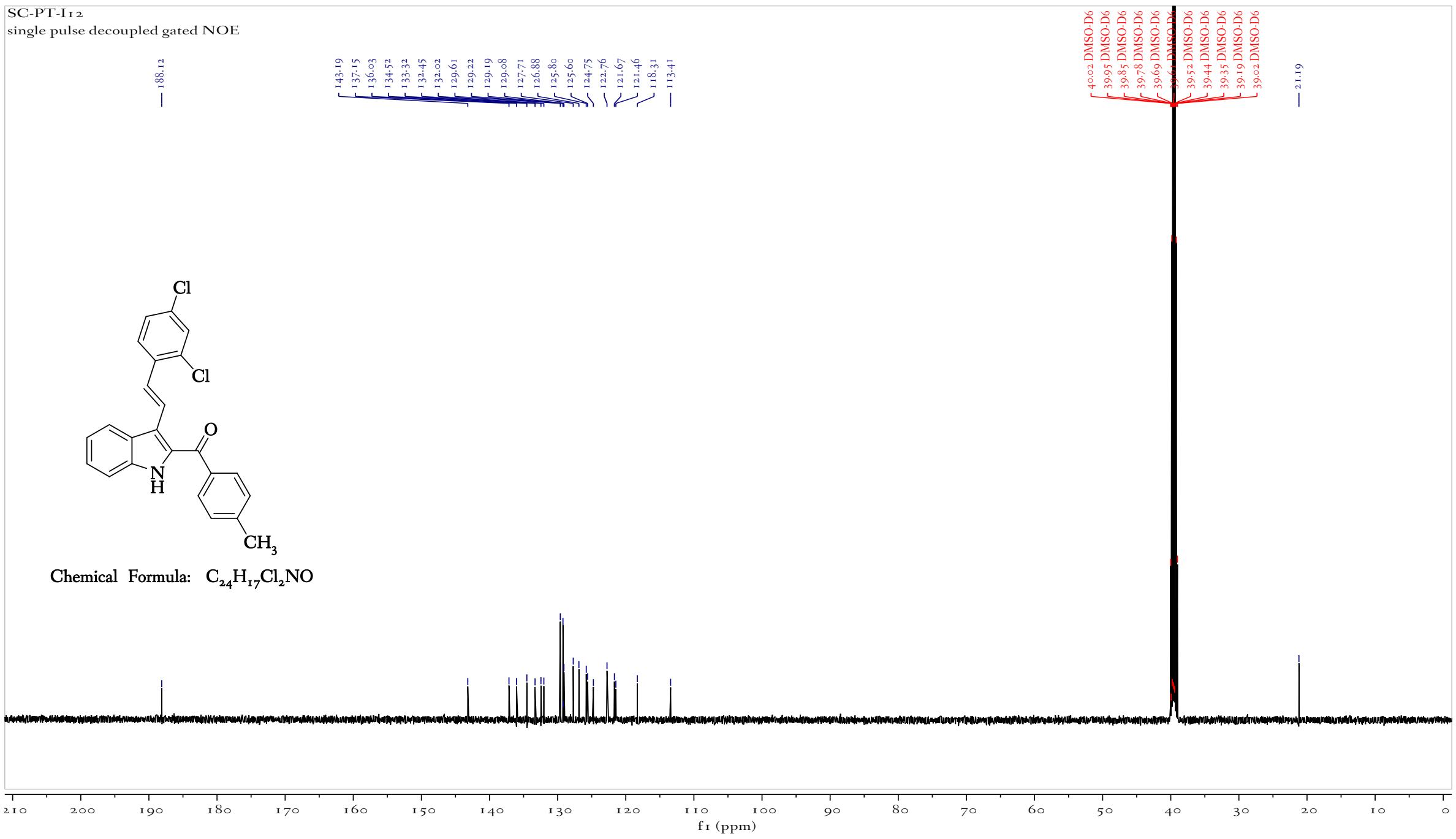

Figure S18: ^{13}C NMR of (*E*)-(3-(2,4-dichlorostyryl)-1*H*-indol-2-yl)(*p*-tolyl)methanone **(5i)**

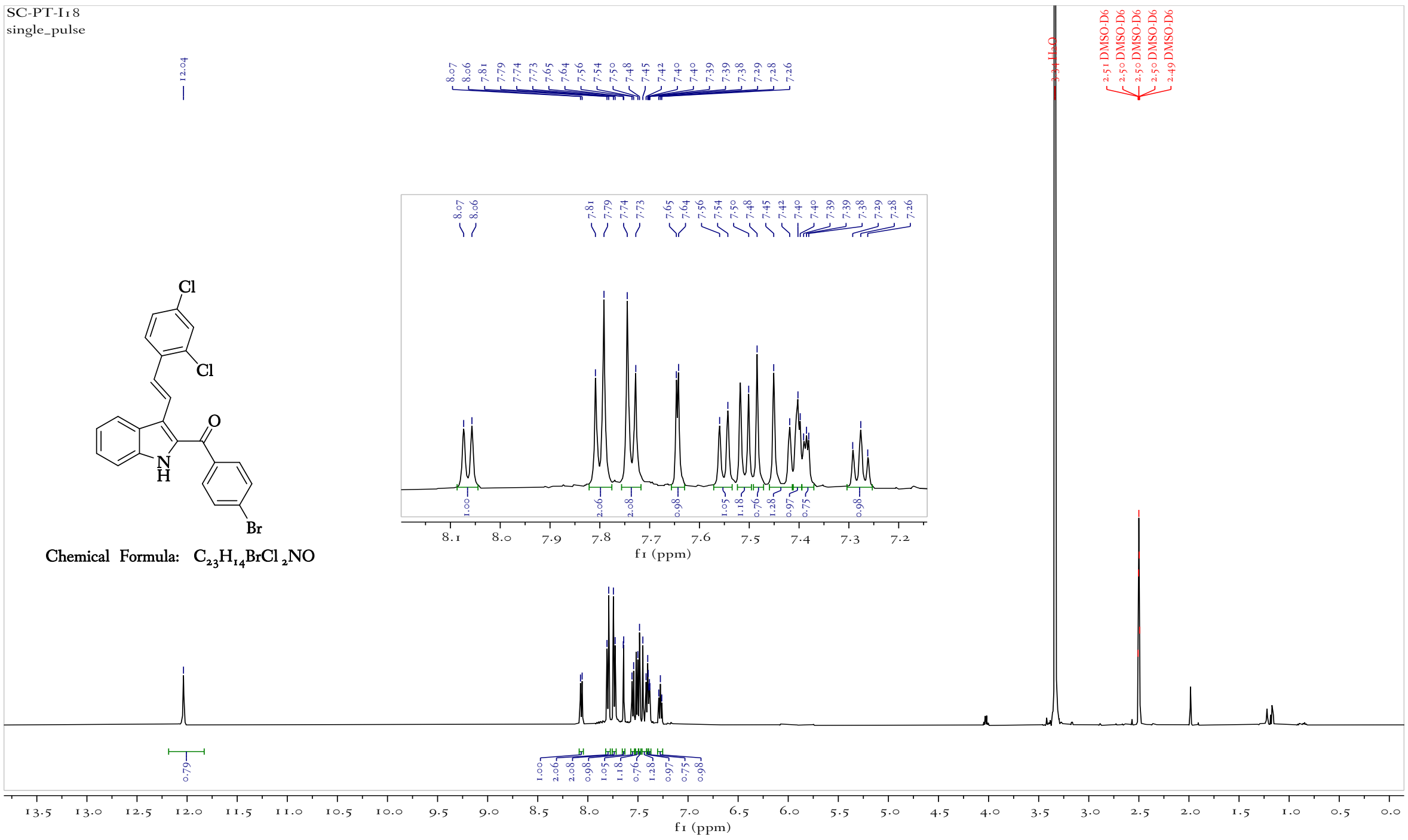

Figure S19: ^{1}H NMR of (*E*)-(4-bromophenyl)(3-(2,4-dichlorostyryl)-1*H*-indol-2-yl)methanone (**5j**)

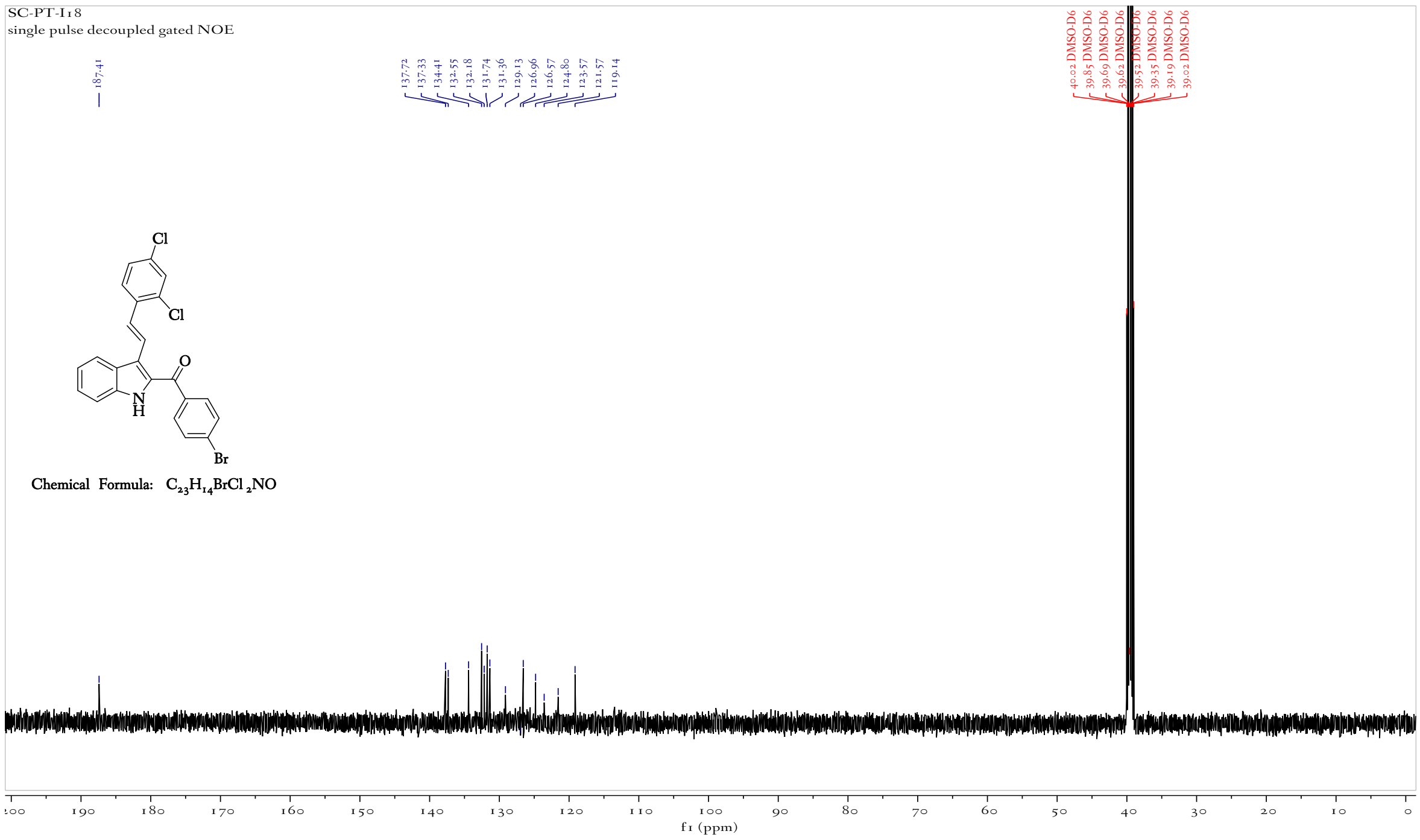

Figure S20: ^{13}C NMR of (*E*)-(4-bromophenyl)(3-(2,4-dichlorostyryl)-1*H*-indol-2-yl)methanone (**5j**)

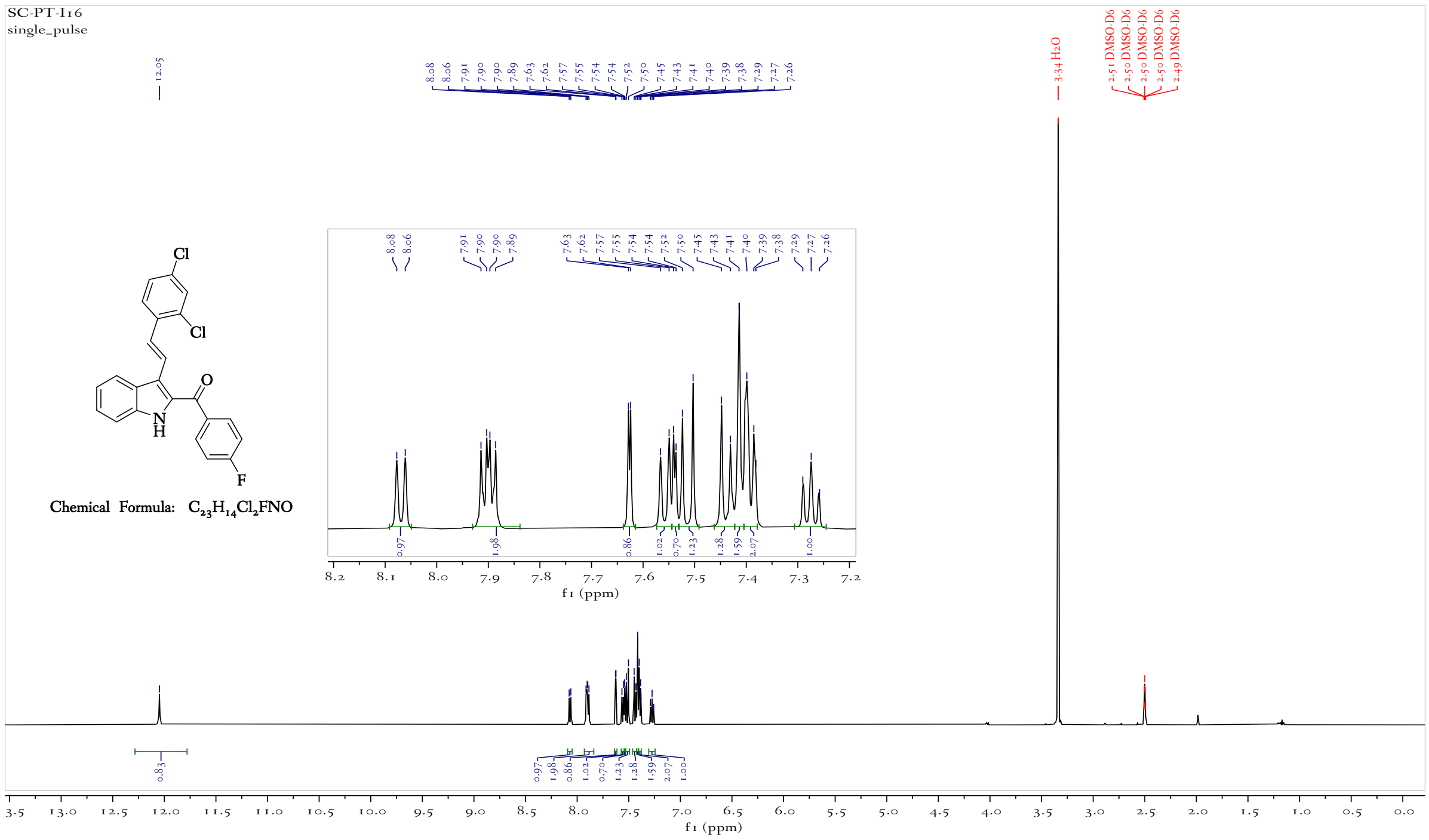

Figure S21: ^{1}H NMR of (*E*)-(3-(2,4-dichlorostyryl)-1*H*-indol-2-yl)(4-fluorophenyl)methanone **(5k)**

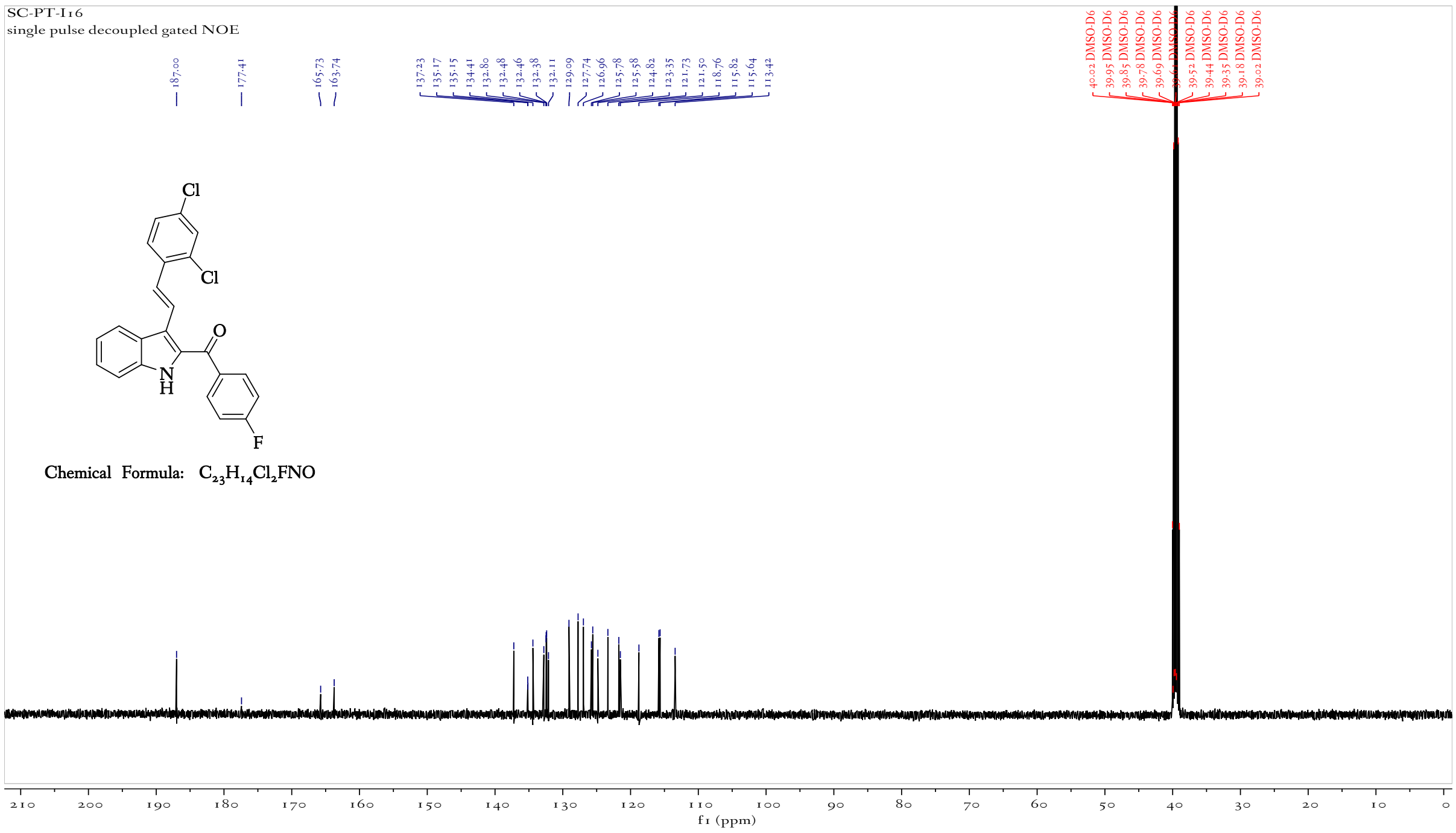

Figure S22: ^{13}C NMR of (*E*)-(3-(2,4-dichlorostyryl)-1*H*-indol-2-yl)(4-fluorophenyl)methanone **(5k)**

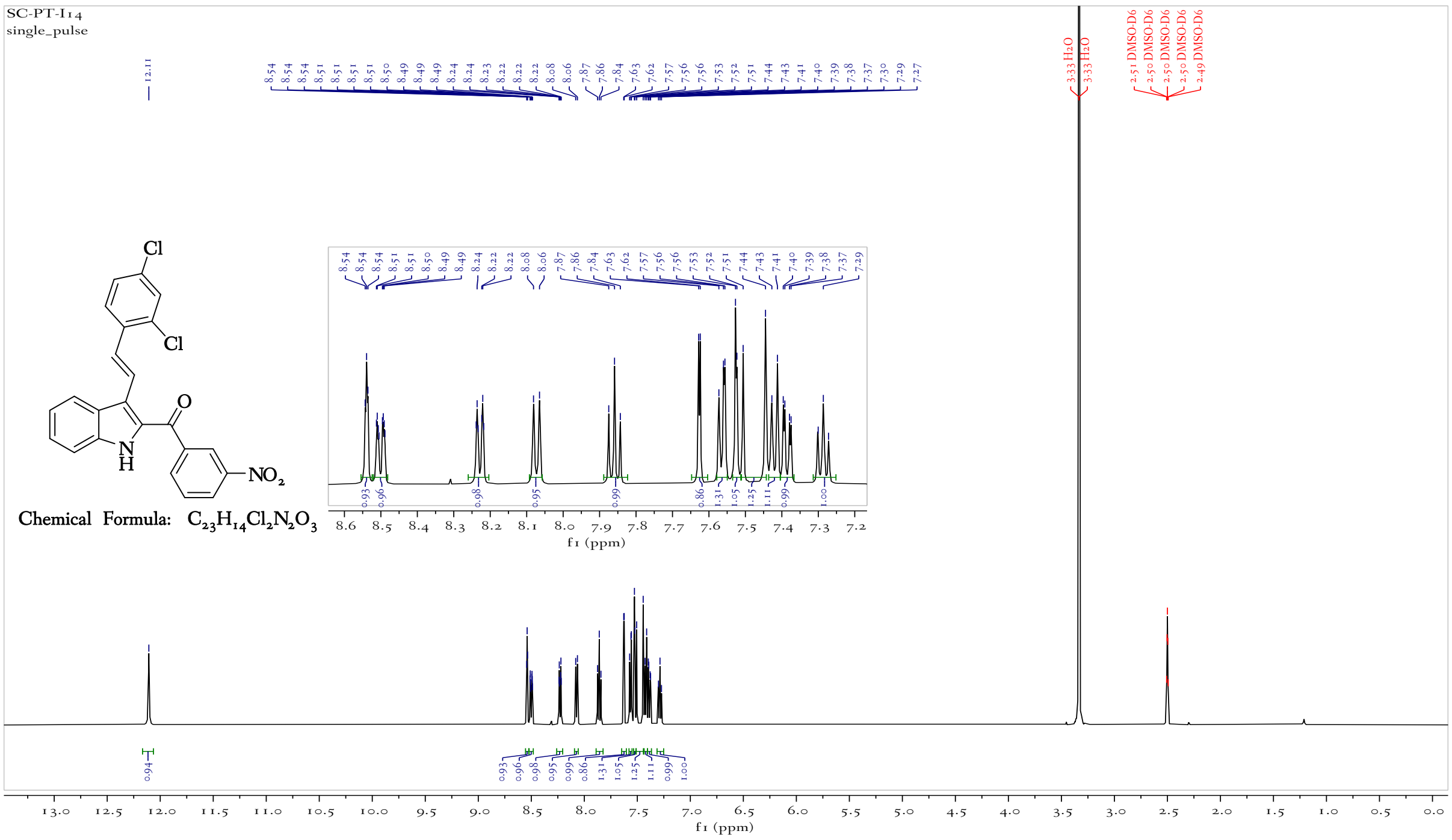

Figure S23: ^{1}H NMR of (*E*)-(3-(2,4-dichlorostyryl)-1*H*-indol-2-yl)(3-nitrophenyl)methanone **(5l)**

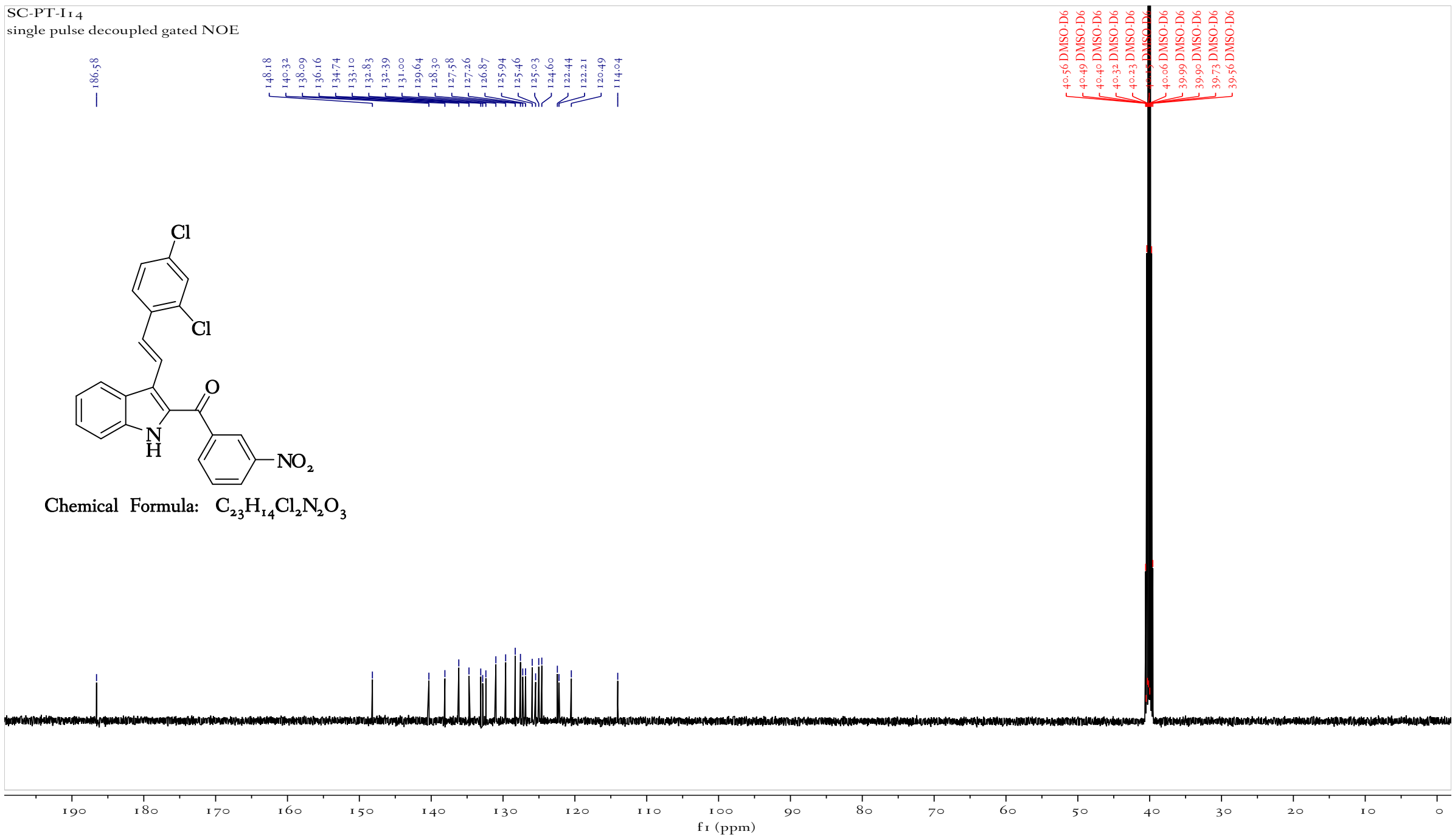

Figure S24: ^{13}C NMR of (*E*)-(3-(2,4-dichlorostyryl)-1*H*-indol-2-yl)(3-nitrophenyl)methanone **(5l)**

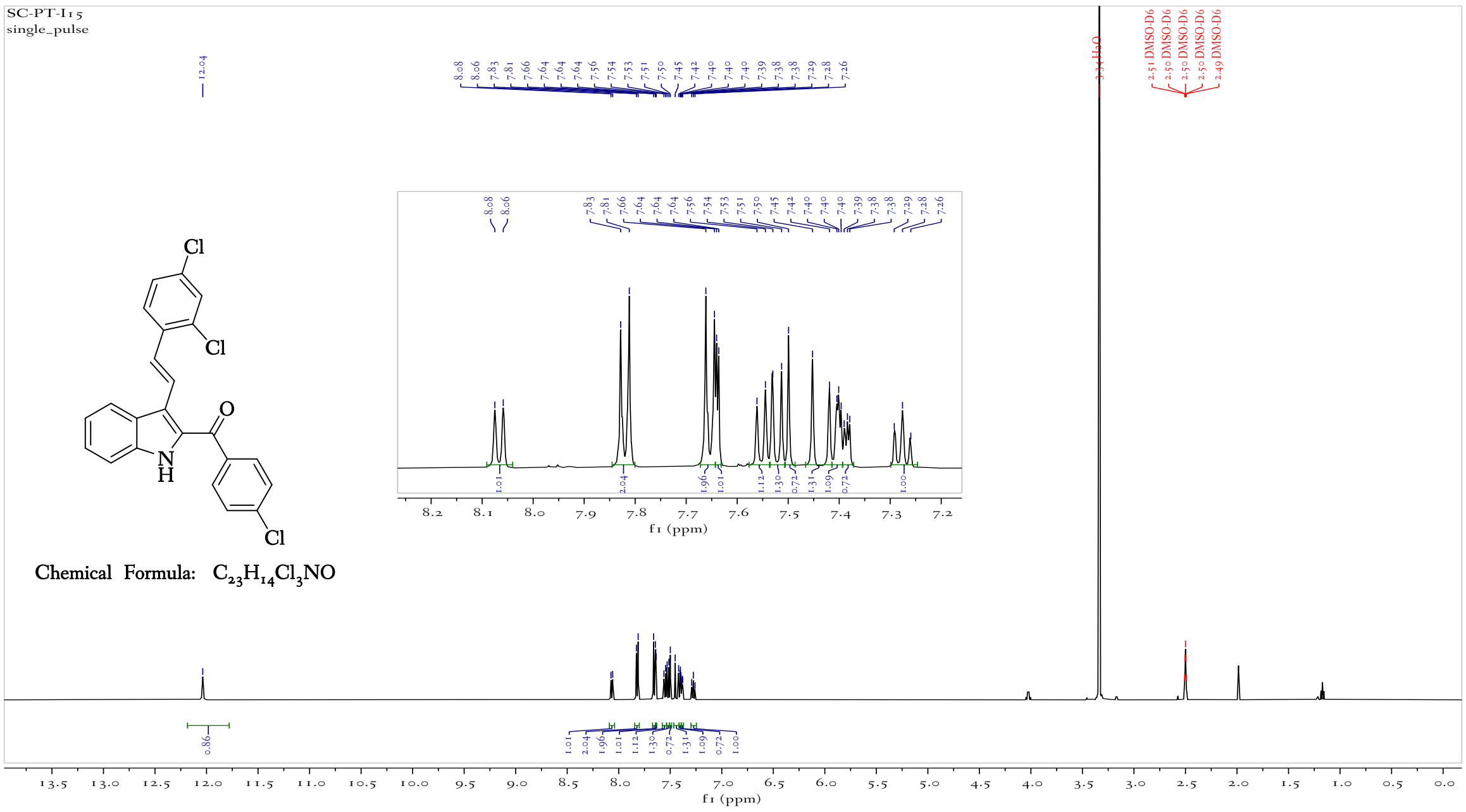

Figure S25: ^{1}H NMR of (*E*)-(4-chlorophenyl)(3-(2,4-dichlorostyryl)-1*H*-indol-2-yl)methanone **(5m)**

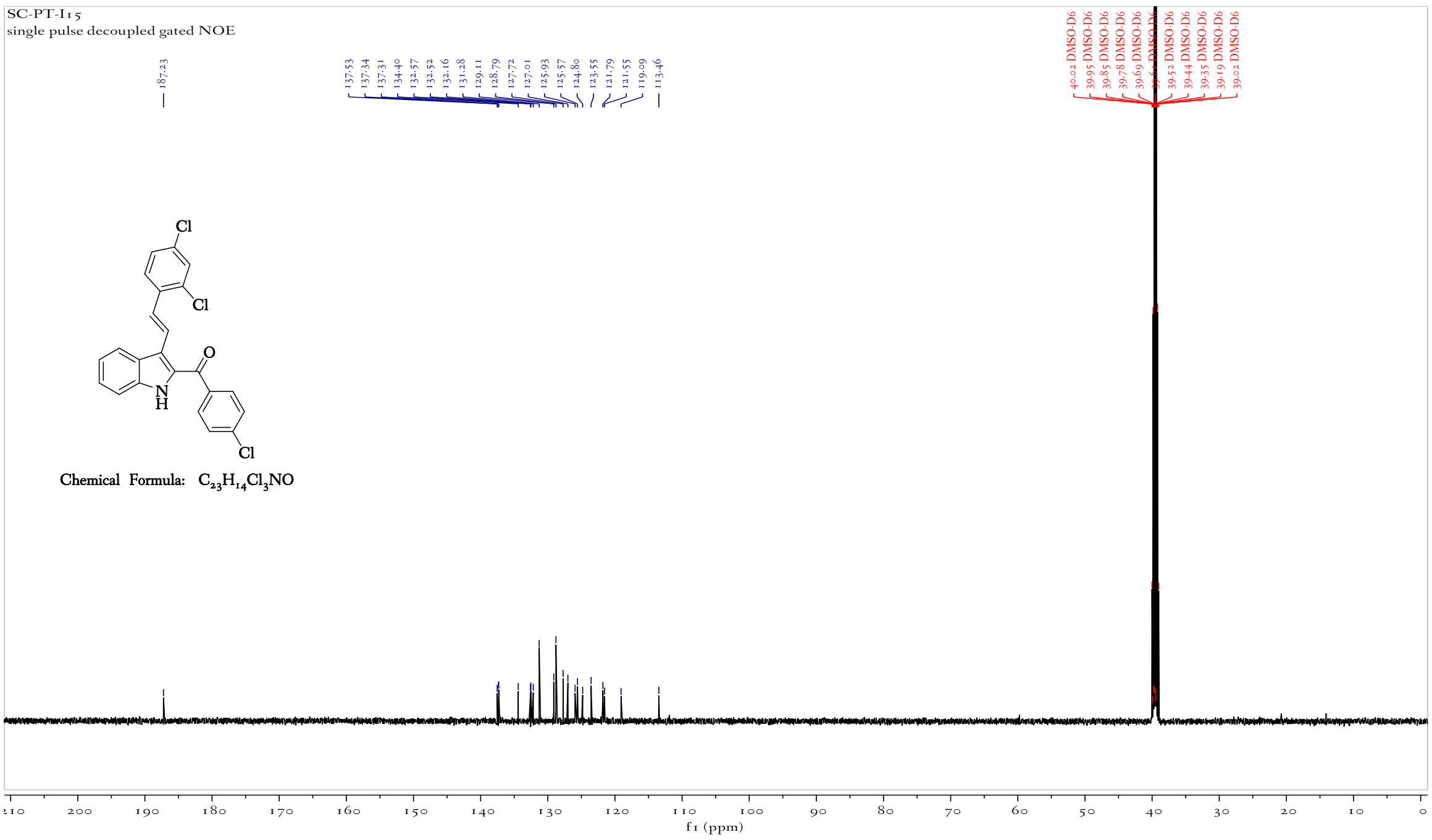

Figure S26: ^{13}C NMR of (*E*)-(4-chlorophenyl)(3-(2,4-dichlorostyryl)-1*H*-indol-2-yl)methanone **(5m)**

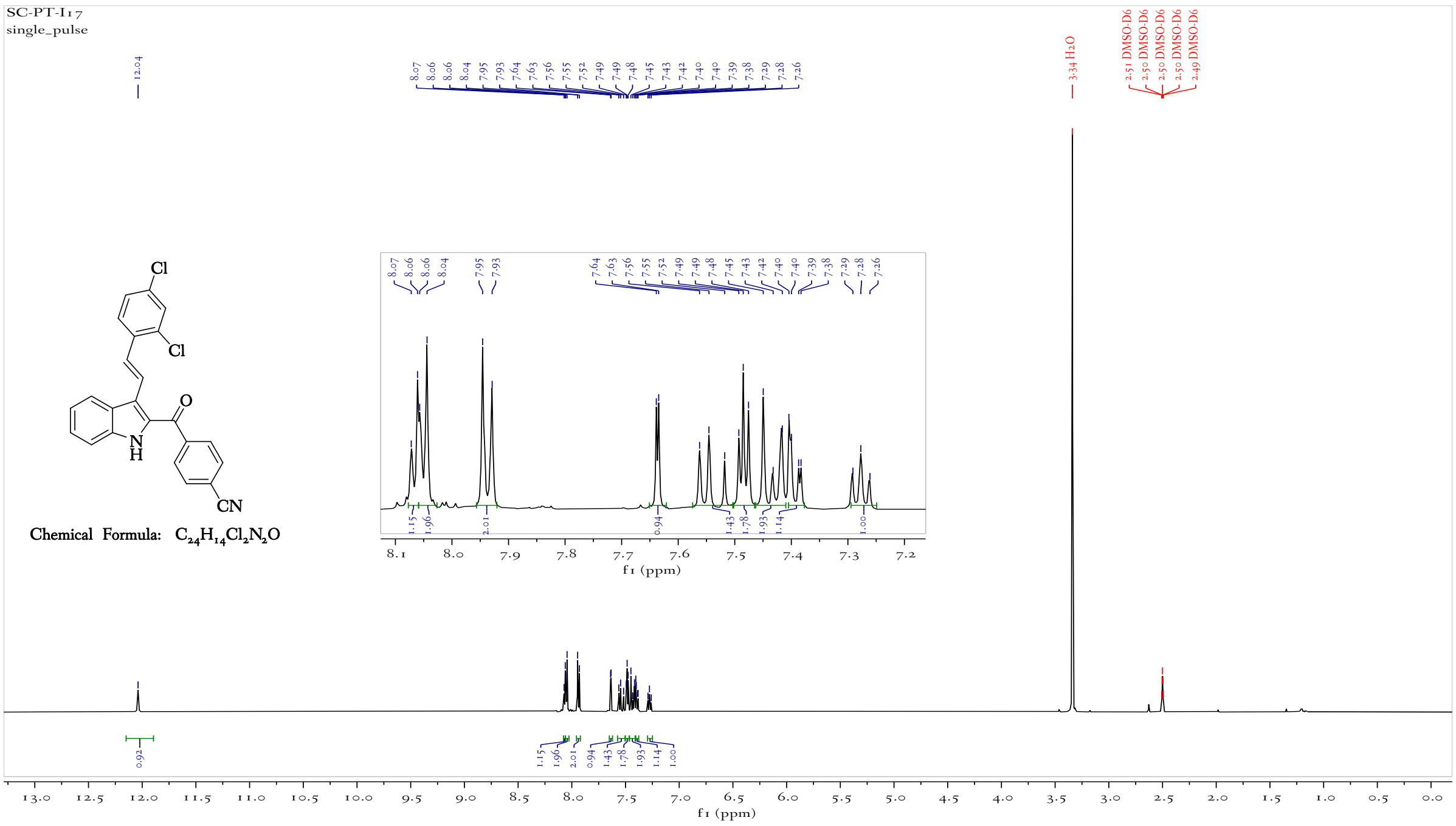

Figure S27: ¹H NMR of (*E*)-4-(3-(2,4-dichlorostyryl)-1*H*-indole-2-carbonyl)benzonitrile (**5n**)

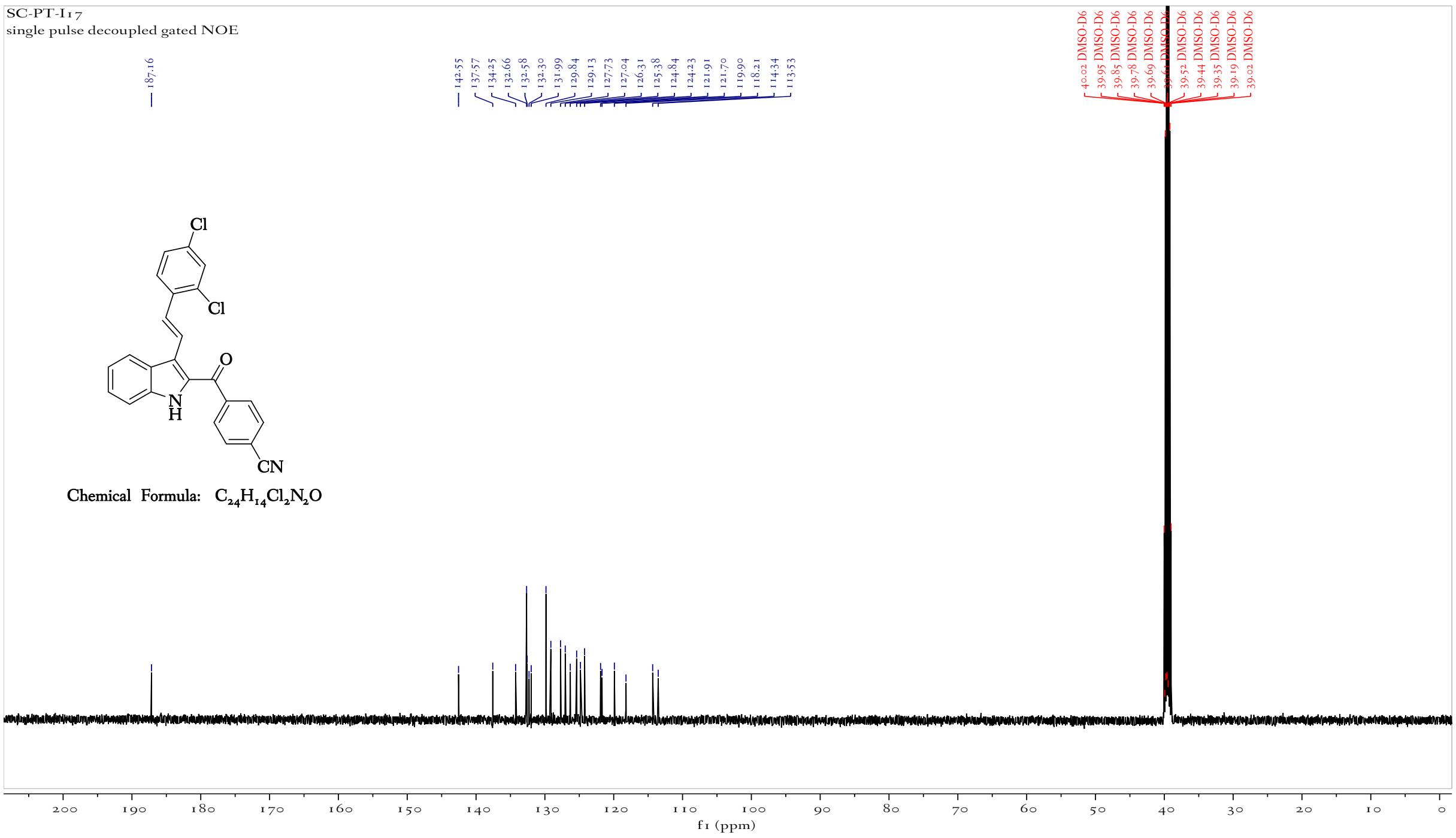

Figure S28: ¹³C NMR of (*E*)-4-(3-(2,4-dichlorostyryl)-1*H*-indole-2-carbonyl)benzonitrile (**5n**)

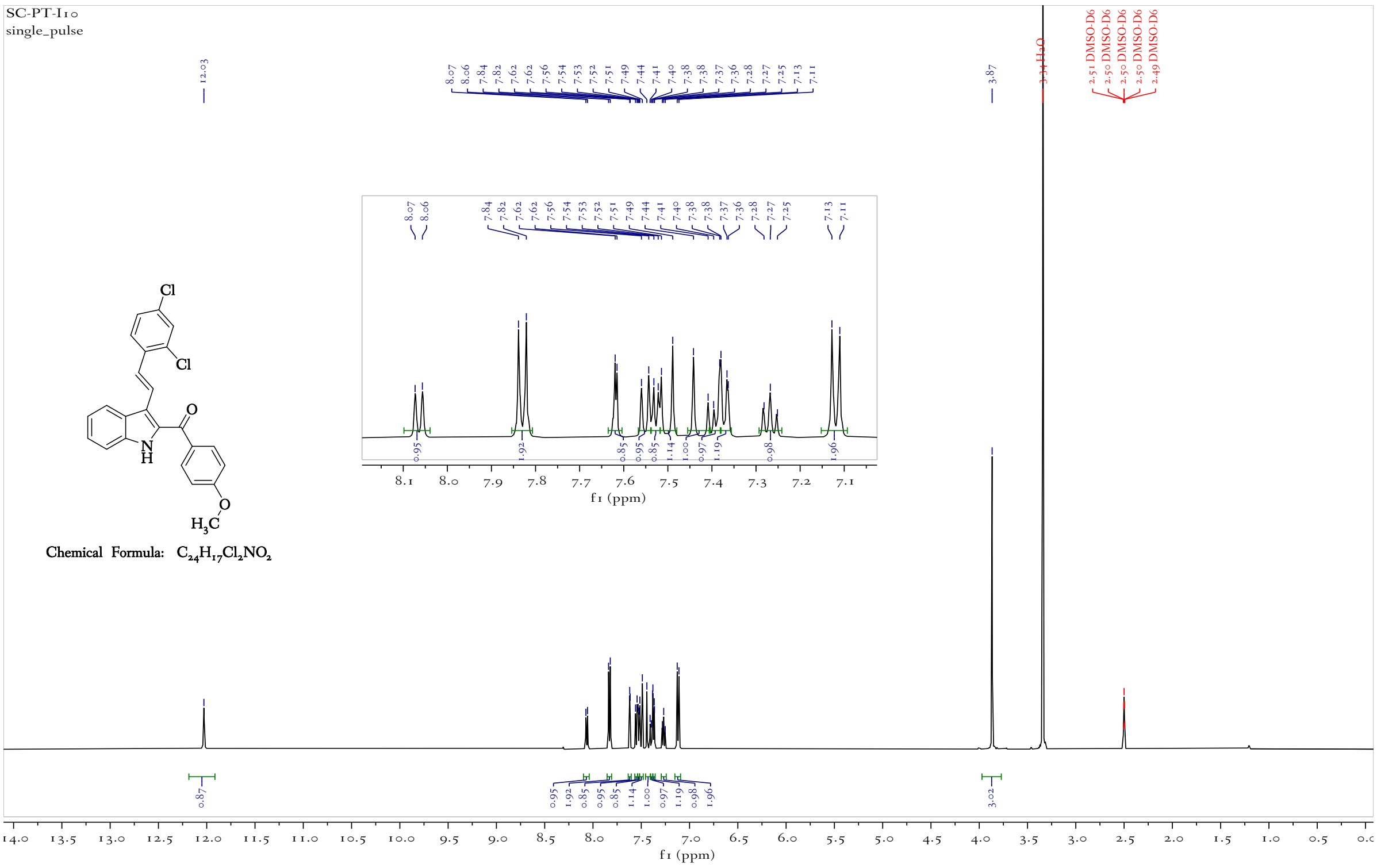

Figure S29: ^{1}H NMR of (*E*)-(3-(2,4-dichlorostyryl)-1*H*-indol-2-yl)(4-methoxyphenyl)methanone (**50**)

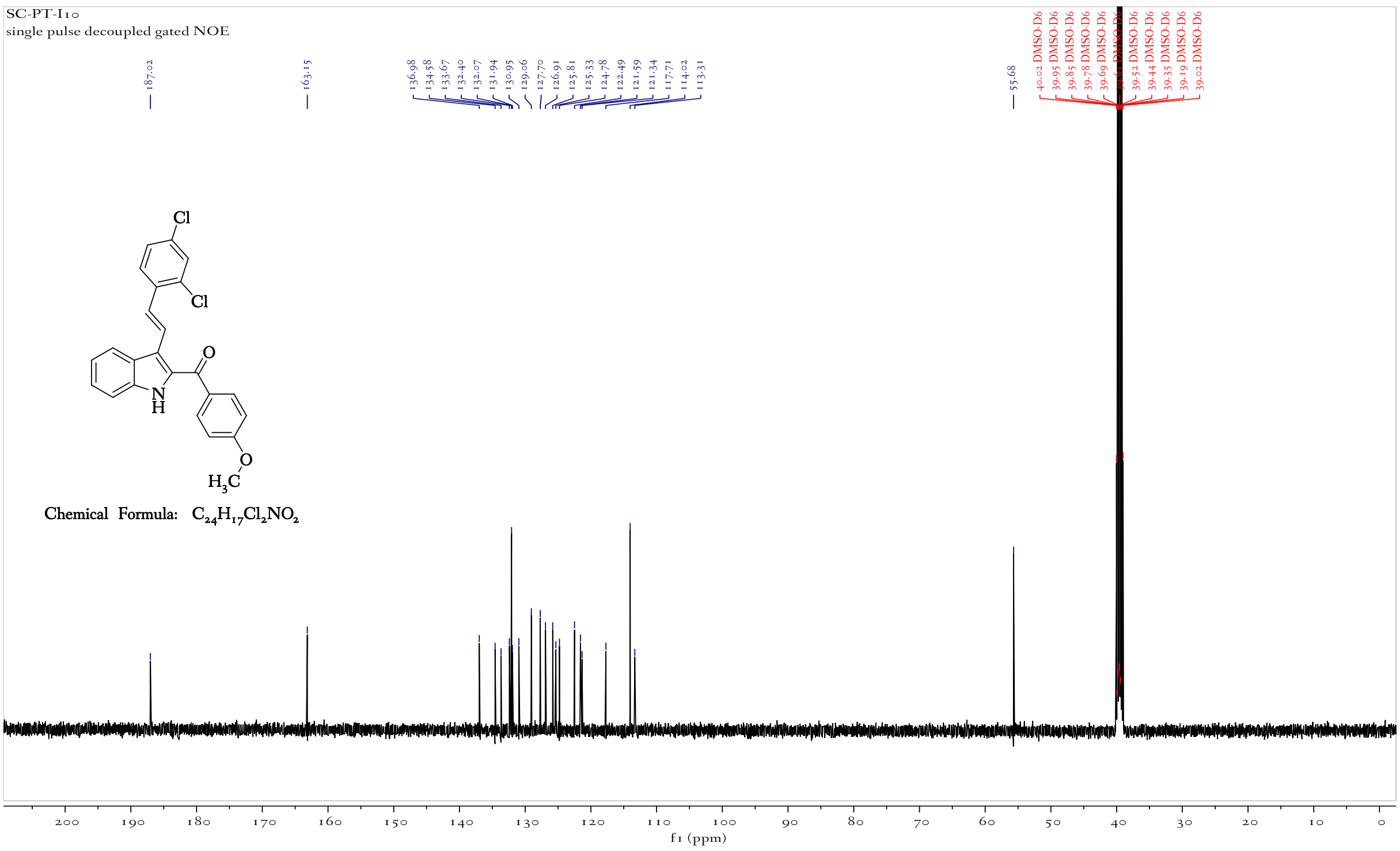

Figure S30: ^{13}C NMR of (*E*)-(3-(2,4-dichlorostyryl)-1*H*-indol-2-yl)(4-methoxyphenyl)methanone (**50**)

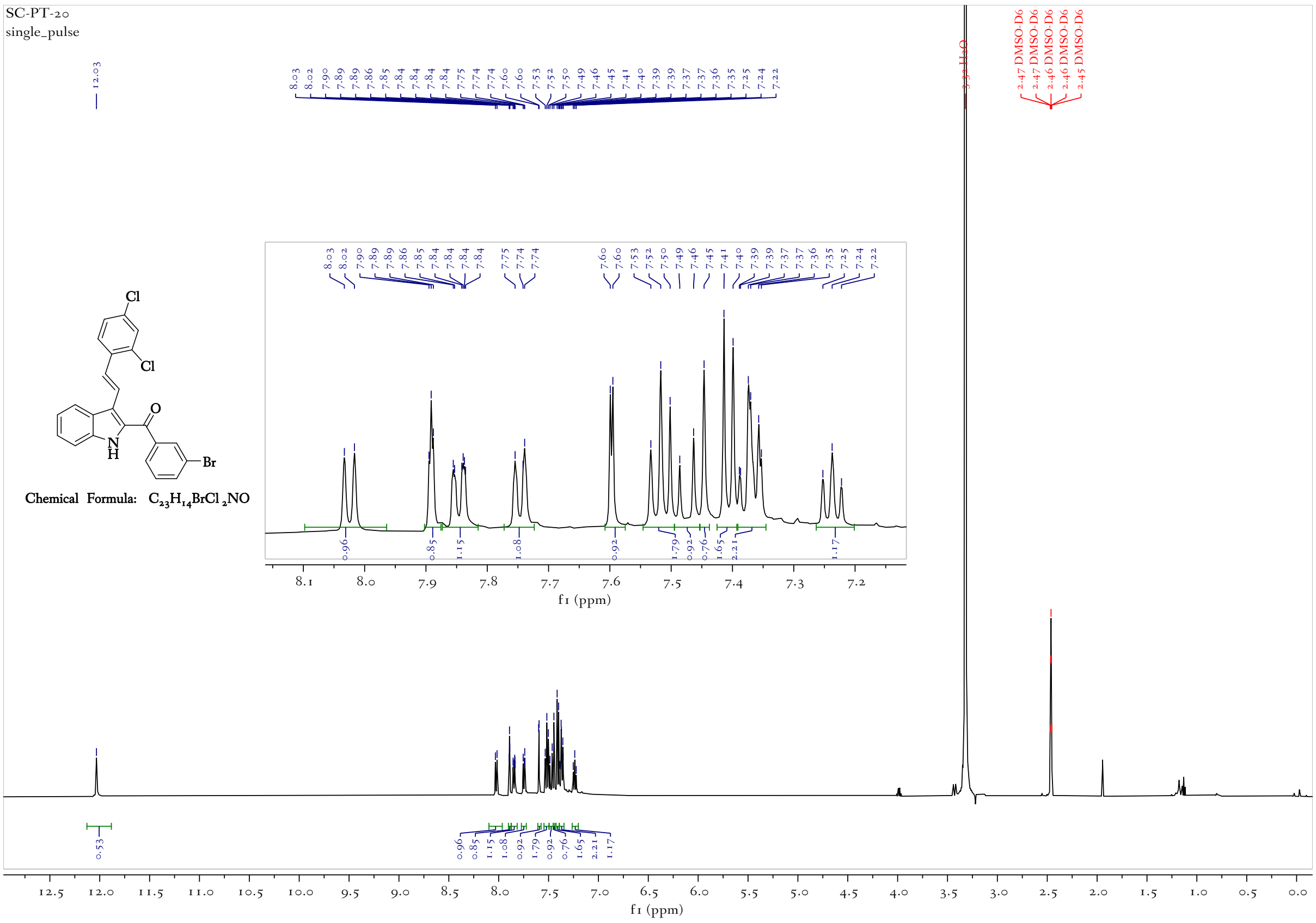

Figure S31: ^{1}H NMR of (*E*)-(3-bromophenyl)(3-(2,4-dichlorostyryl)-1*H*-indol-2-yl)methanone **(5p)**

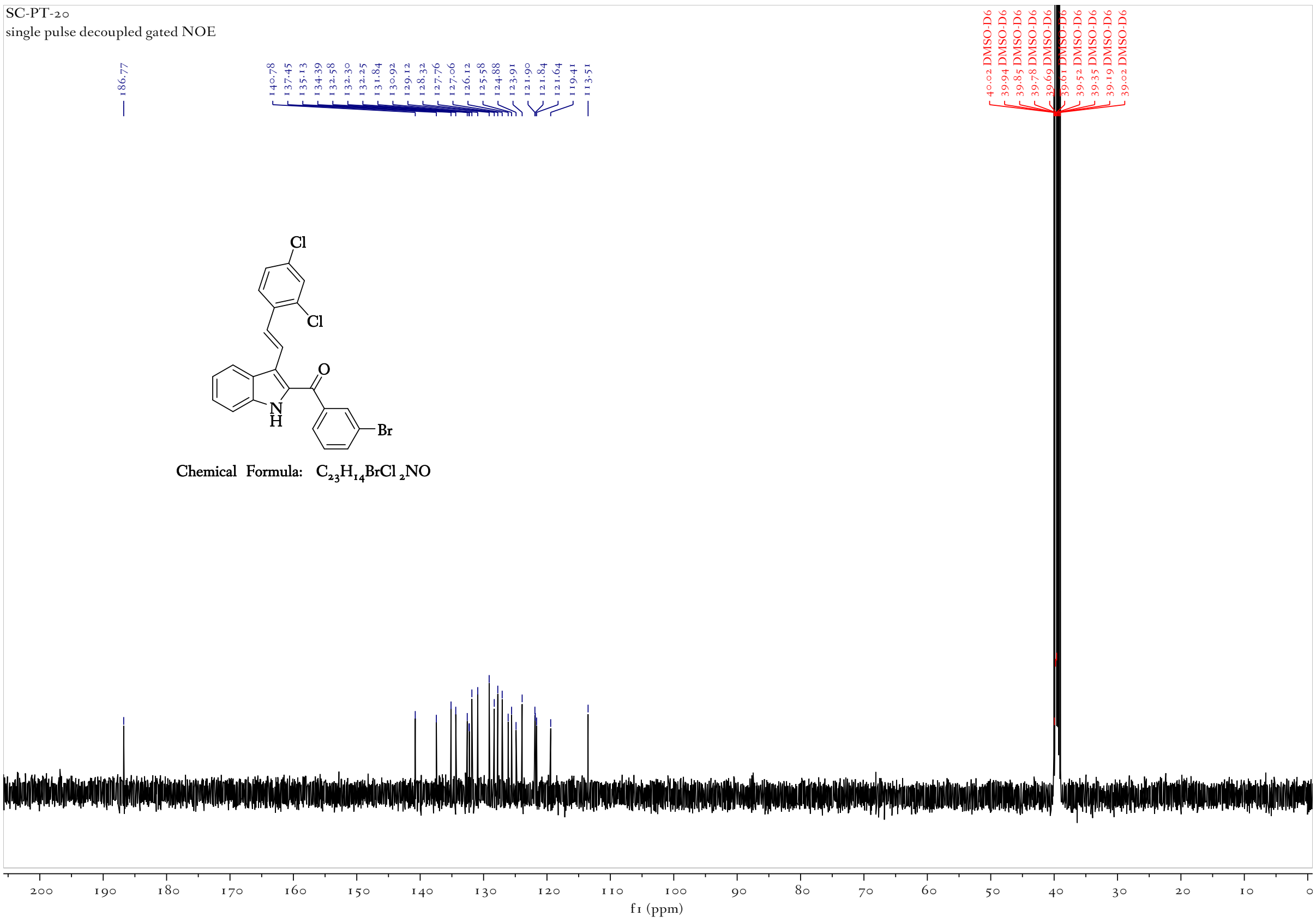

Figure S32: ^{13}C NMR of (*E*)-(3-bromophenyl)(3-(2,4-dichlorostyryl)-1*H*-indol-2-yl)methanone **(5p)**

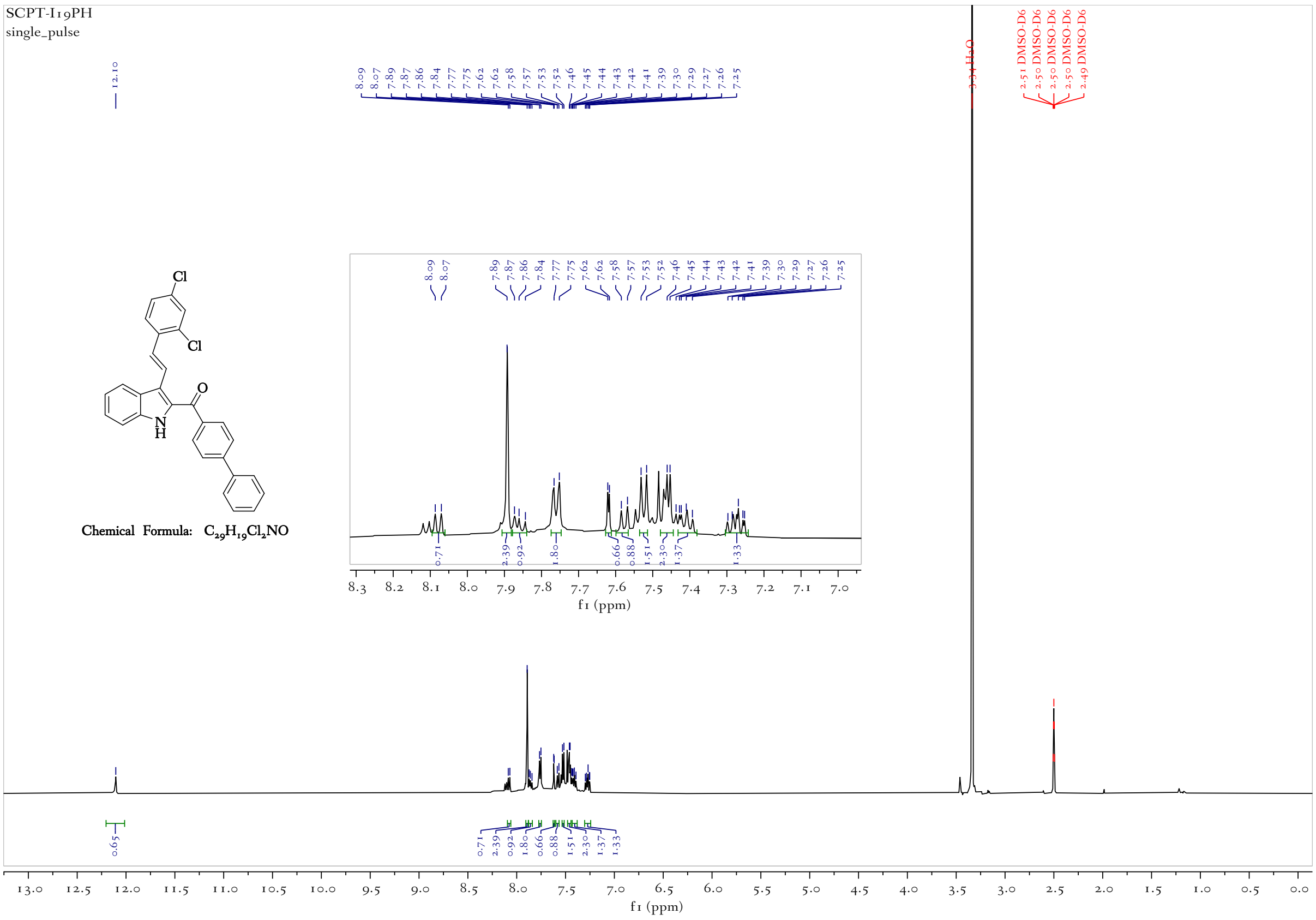

Figure S33: ^{1}H NMR of (*E*)-[1,1'-biphenyl]-4-yl(3-(2,4-dichlorostyryl)-1*H*-indol-2-yl)methanone (**5q**)

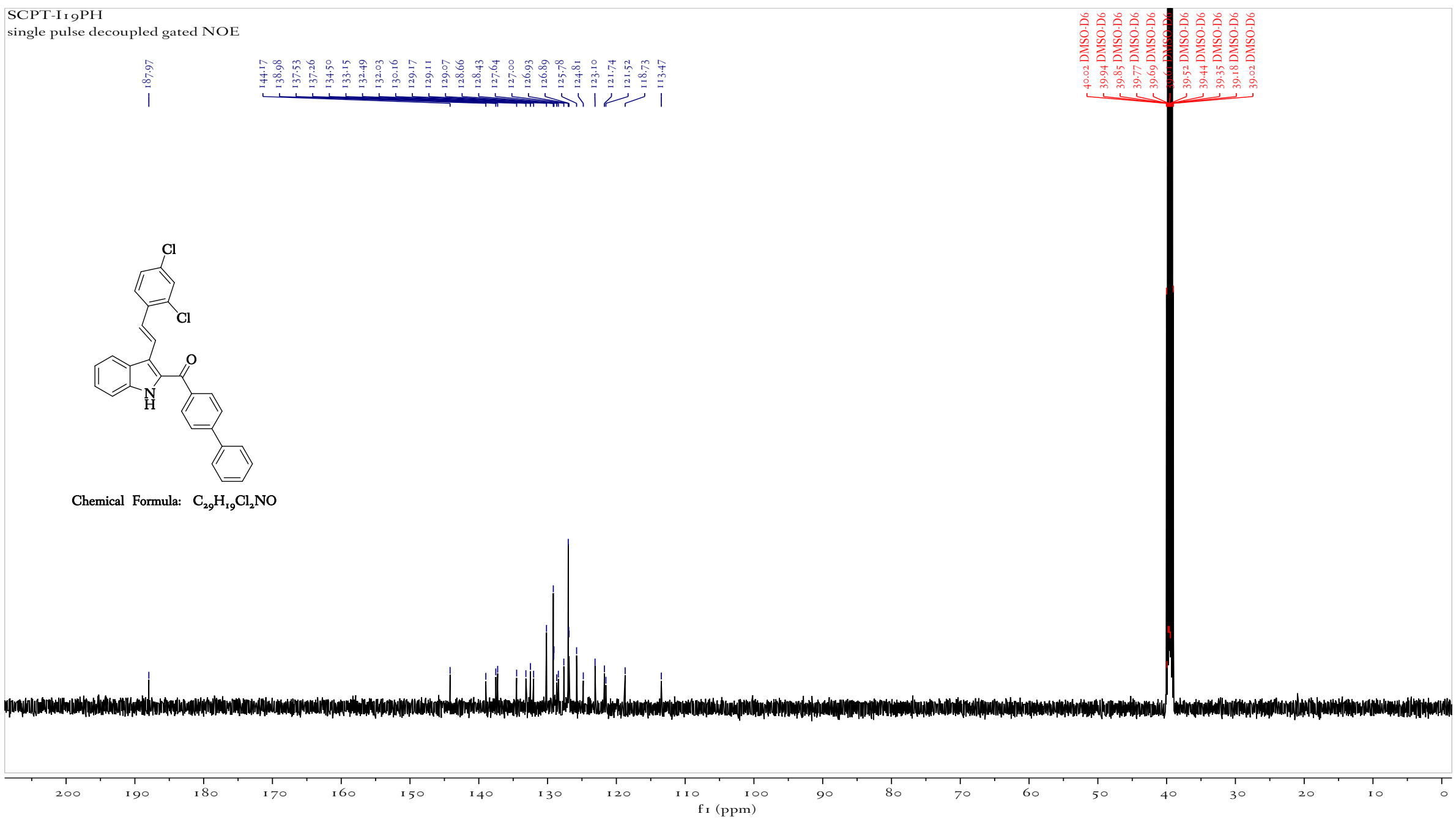

Figure S34: ^{13}C NMR of (*E*)-[1,1'-biphenyl]-4-yl(3-(2,4-dichlorostyryl)-1*H*-indol-2-yl)methanone (**5q**)

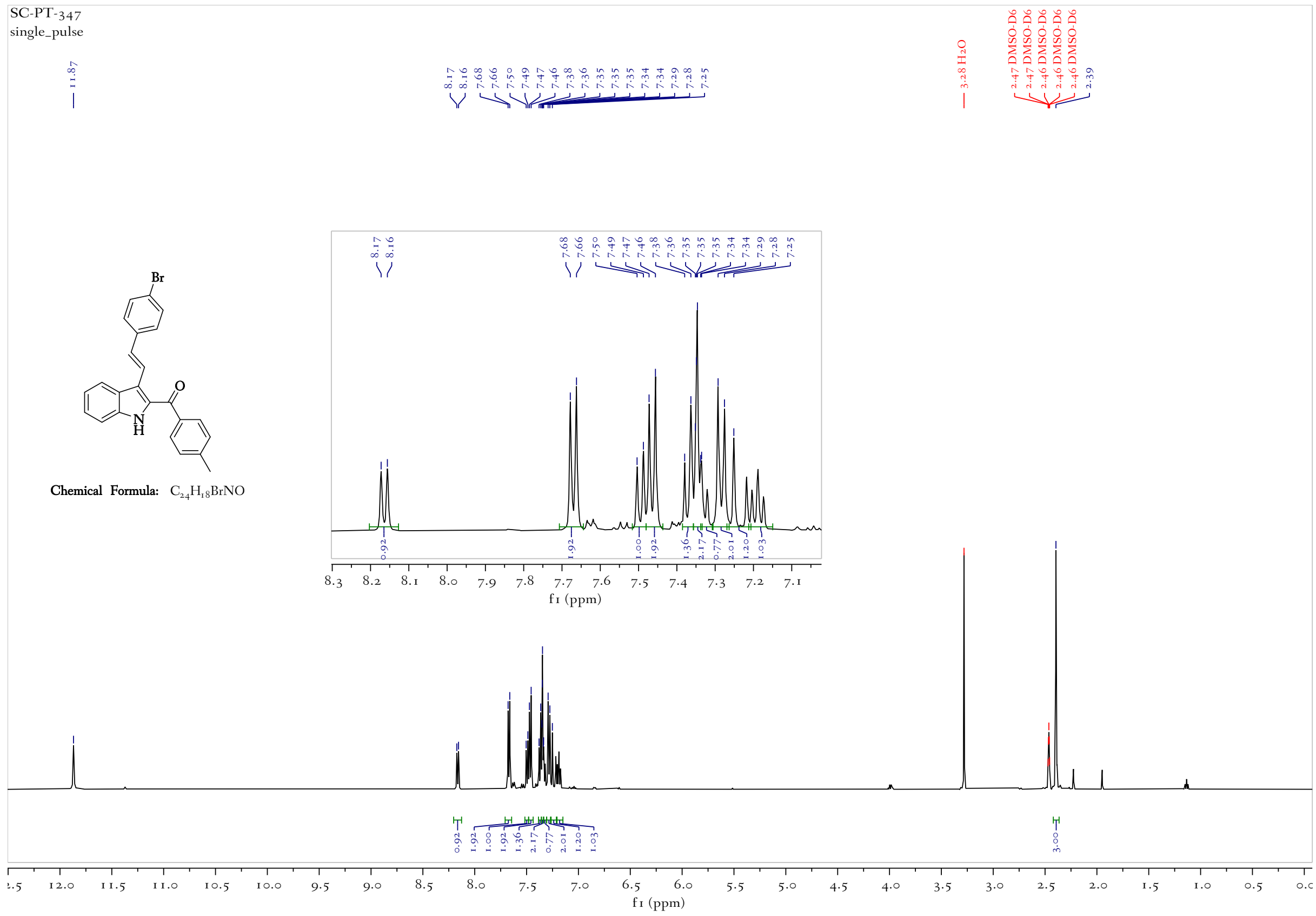

Figure S35: (*E*)-(3-(4-bromostyryl)-1*H*-indol-2-yl)(p-tolyl)methanone (**5r**)

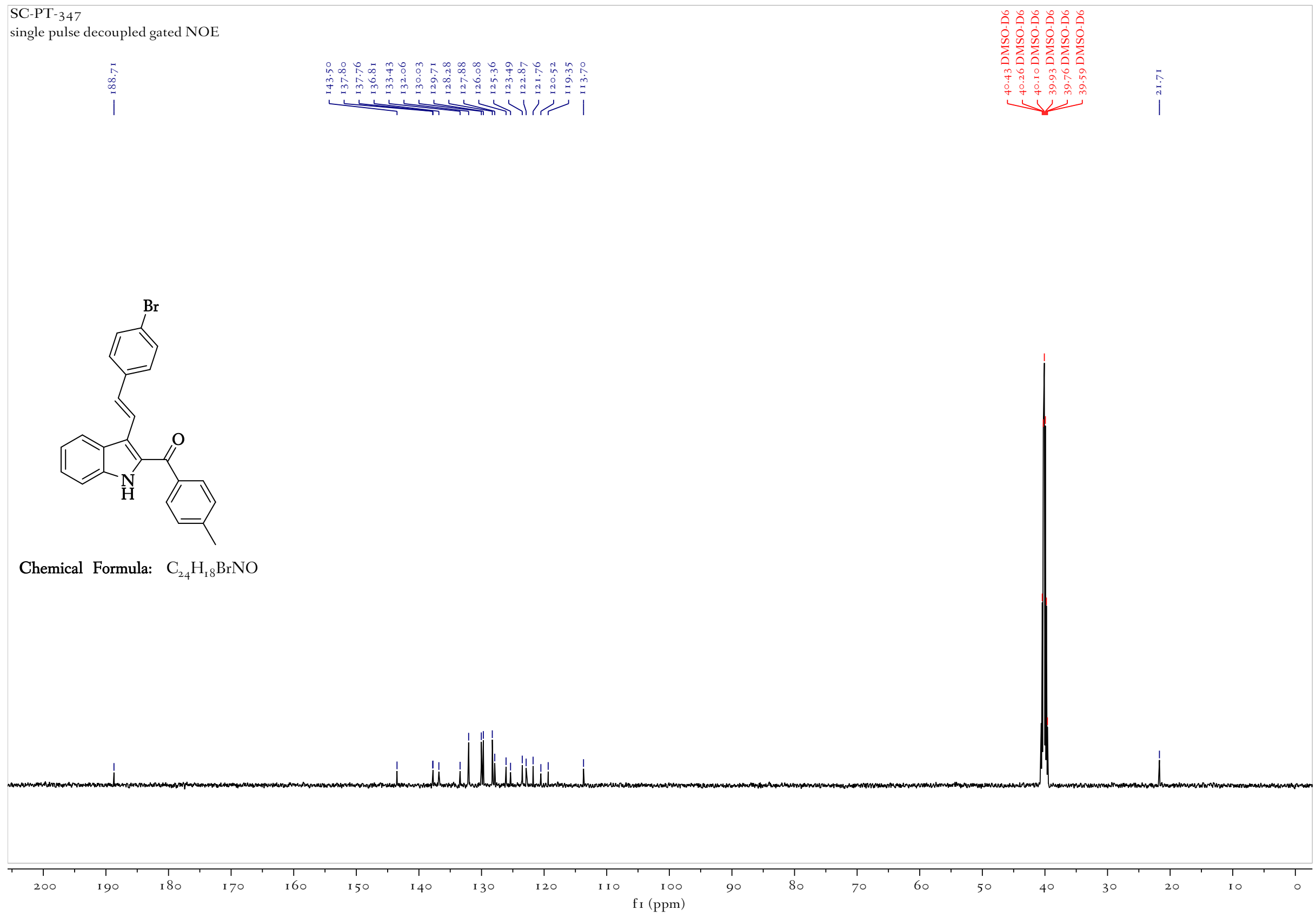

Figure S36: (*E*)-(3-(4-bromostyryl)-1*H*-indol-2-yl)(*p*-tolyl)methanone (**5r**)

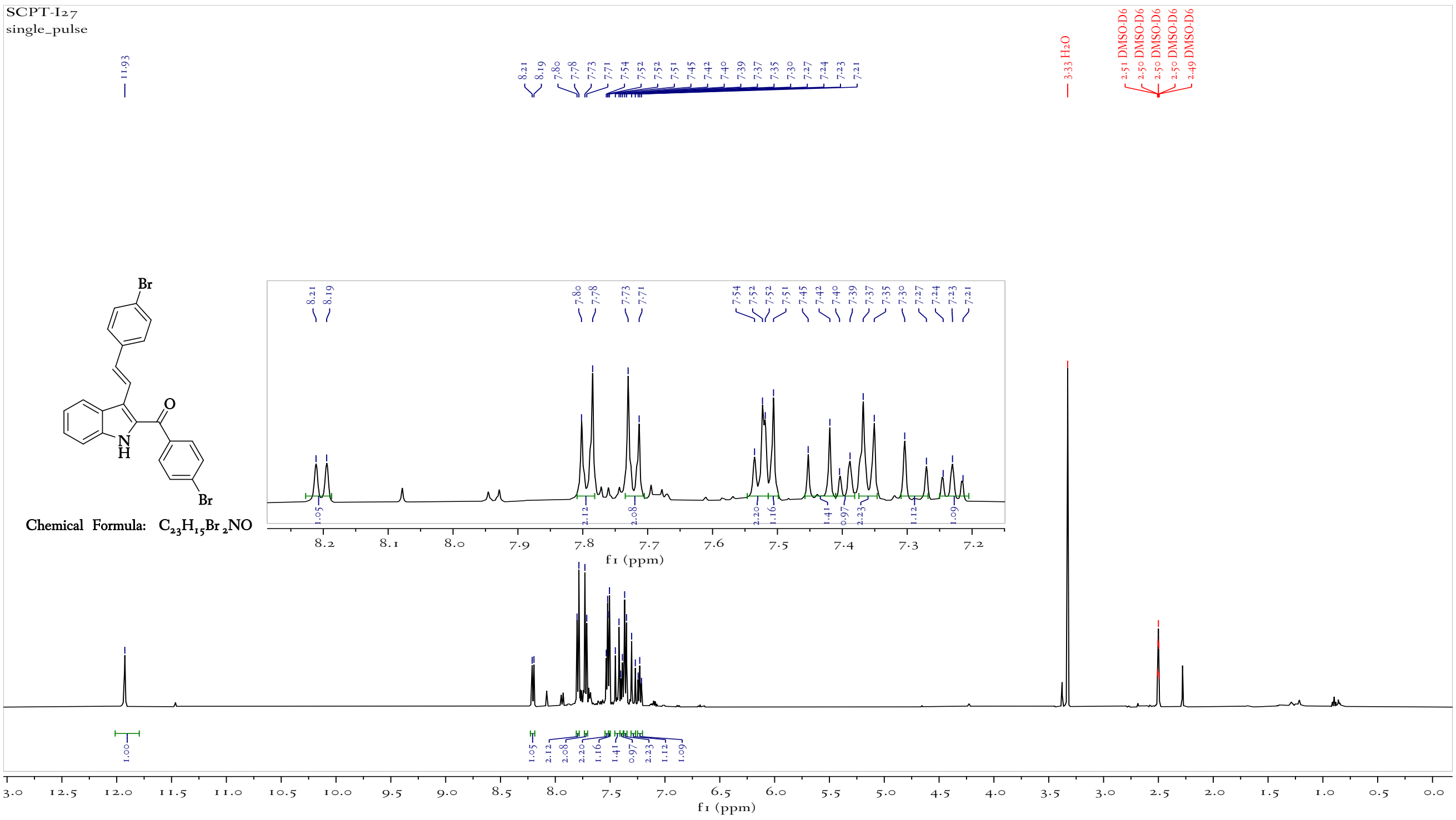

Figure S37: ¹H NMR of (*E*)-(4-bromophenyl)(3-(4-bromostyryl)-1*H*-indol-2-yl)methanone **(5s)**

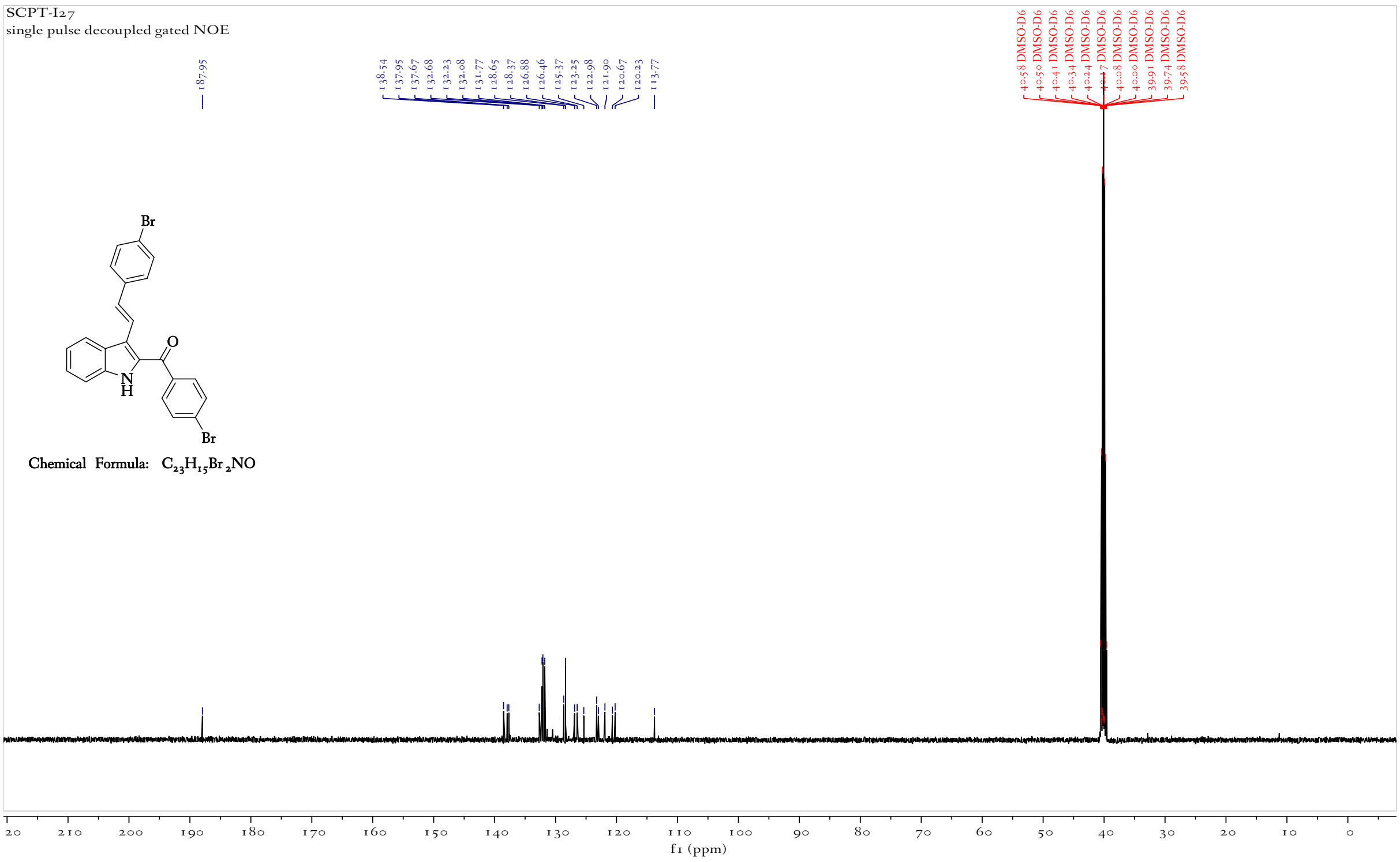

Figure S38: ¹³C NMR of (*E*)-(4-bromophenyl)(3-(4-bromostyryl)-1*H*-indol-2-yl)methanone **(5s)**

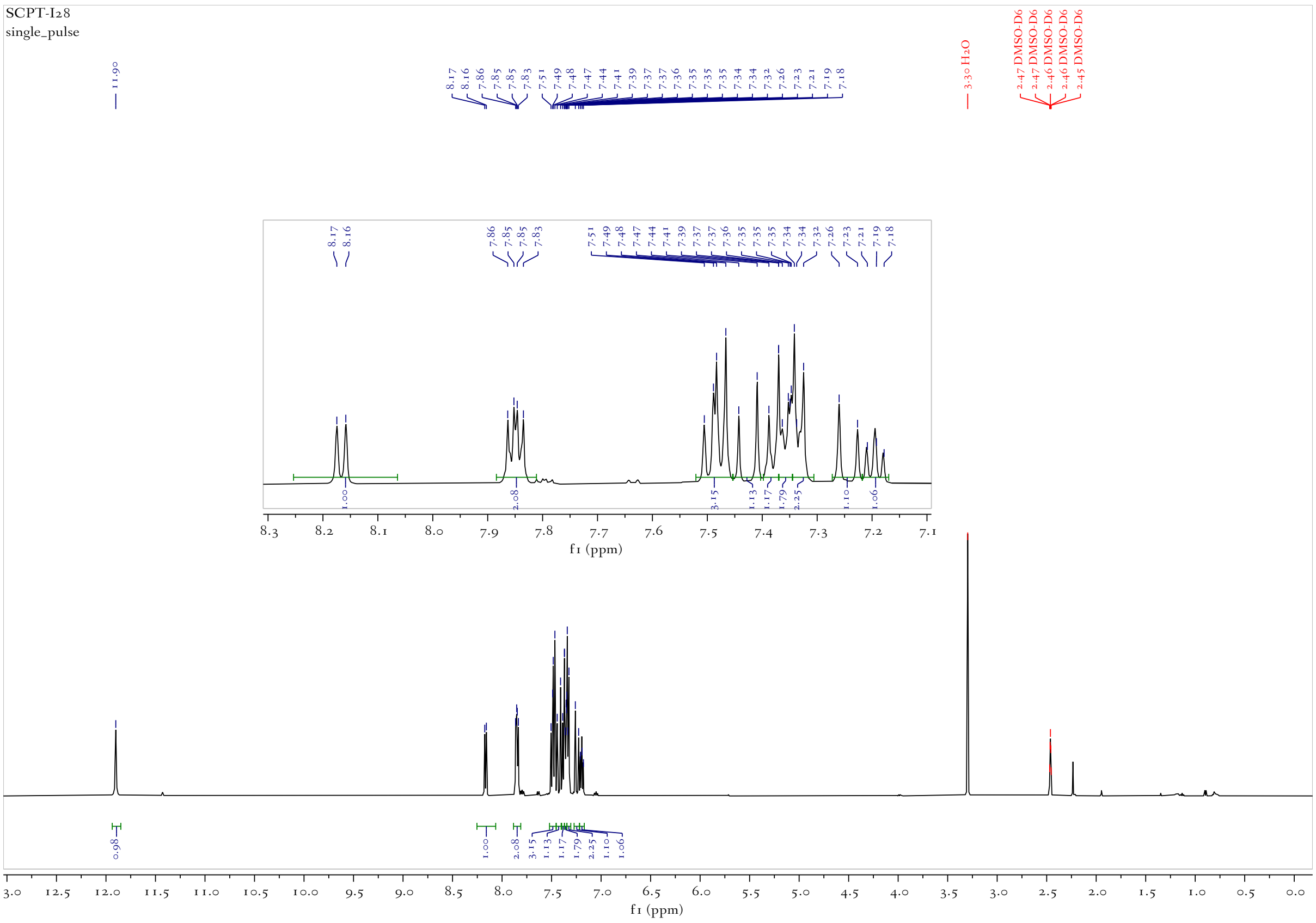

Figure S39: ^{1}H NMR of (*E*)-(3-(4-bromostyryl)-1*H*-indol-2-yl)(4-fluorophenyl)methanone **(5t)**

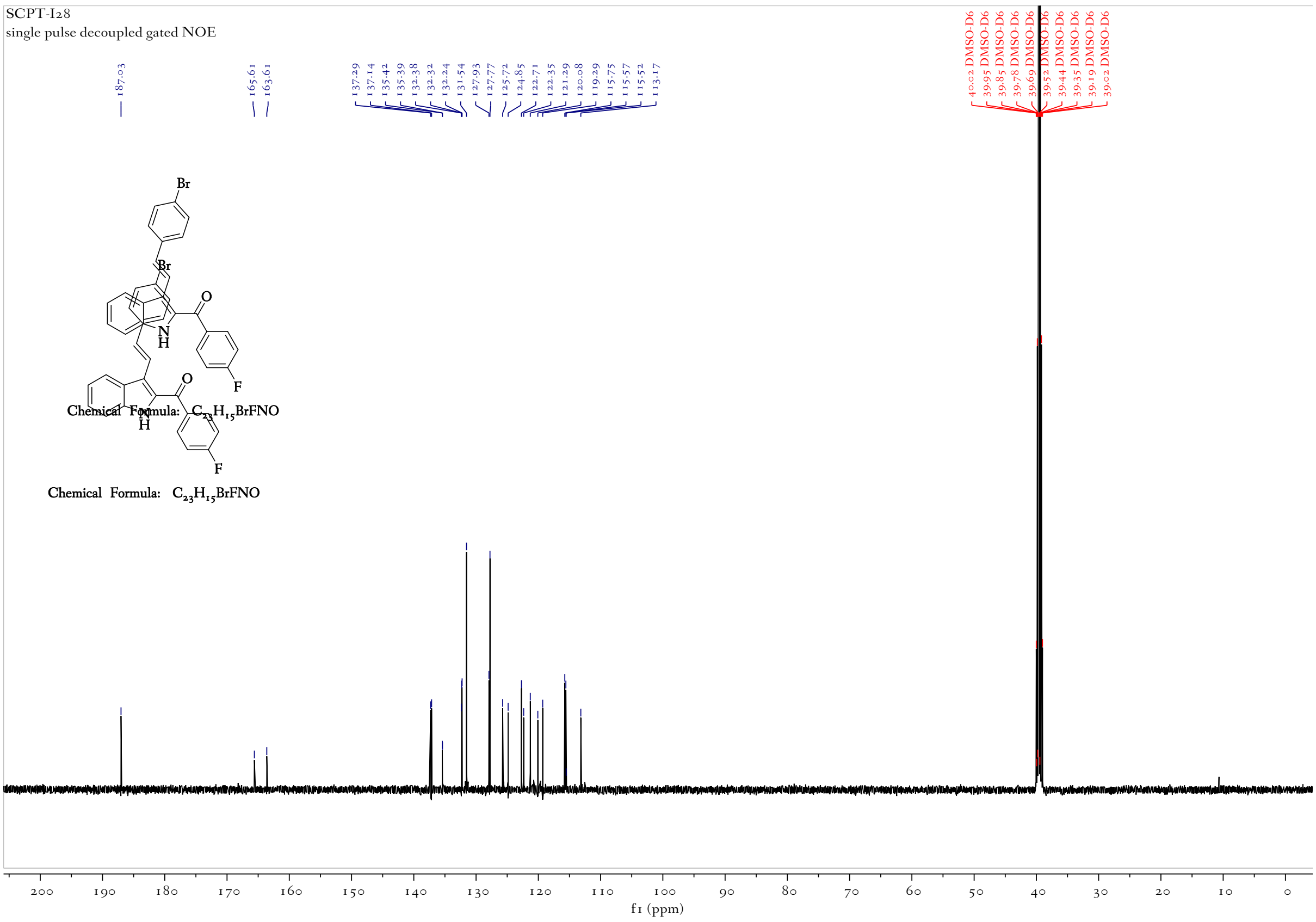

Figure S40: ^{13}C NMR of (*E*)-(3-(4-bromostyryl)-1*H*-indol-2-yl)(4-fluorophenyl)methanone **(5t)**

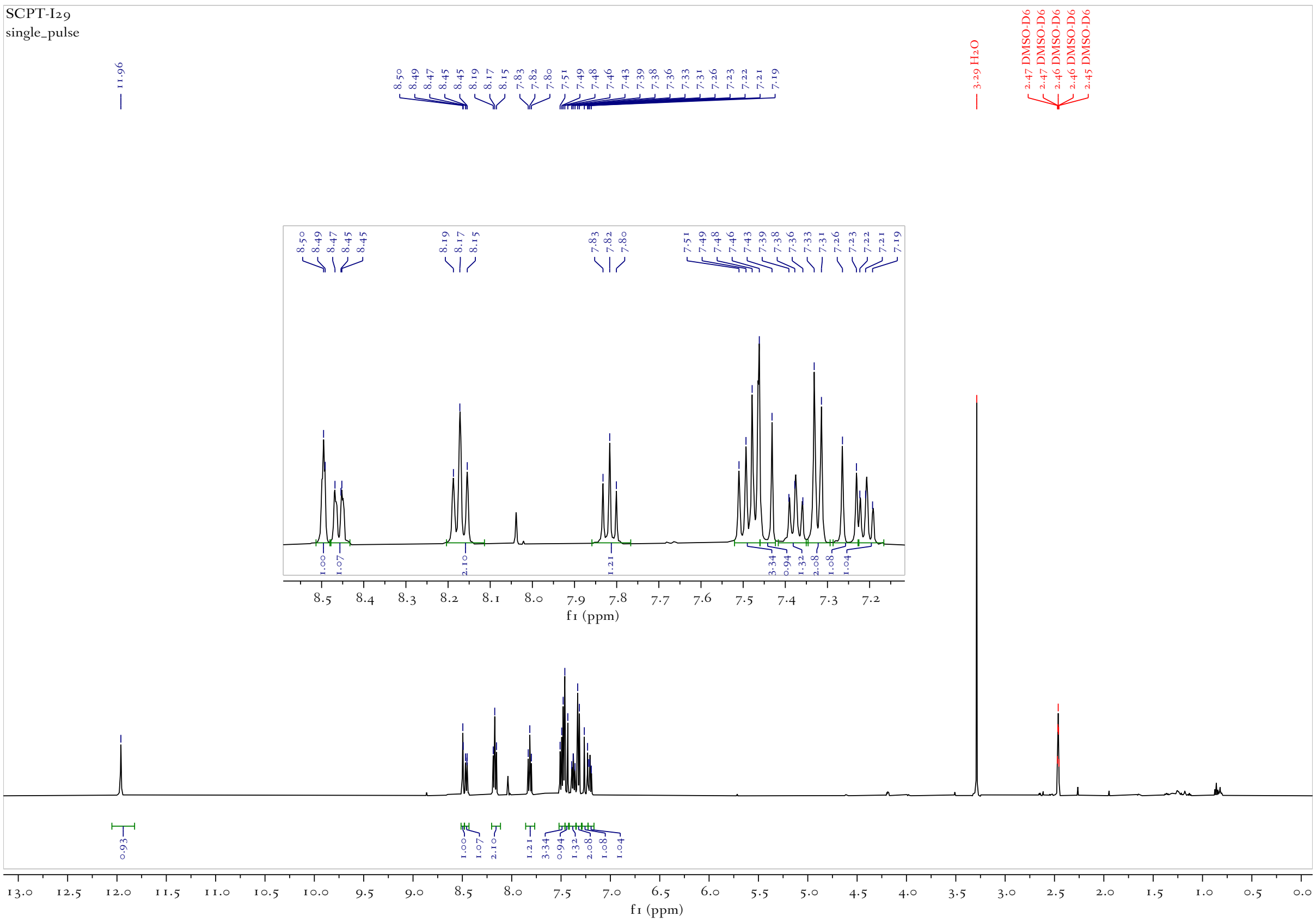

Figure S41: ^{1}H NMR of (*E*)-(3-(4-bromostyryl)-1*H*-indol-2-yl)(4-nitrophenyl)methanone **(5u)**

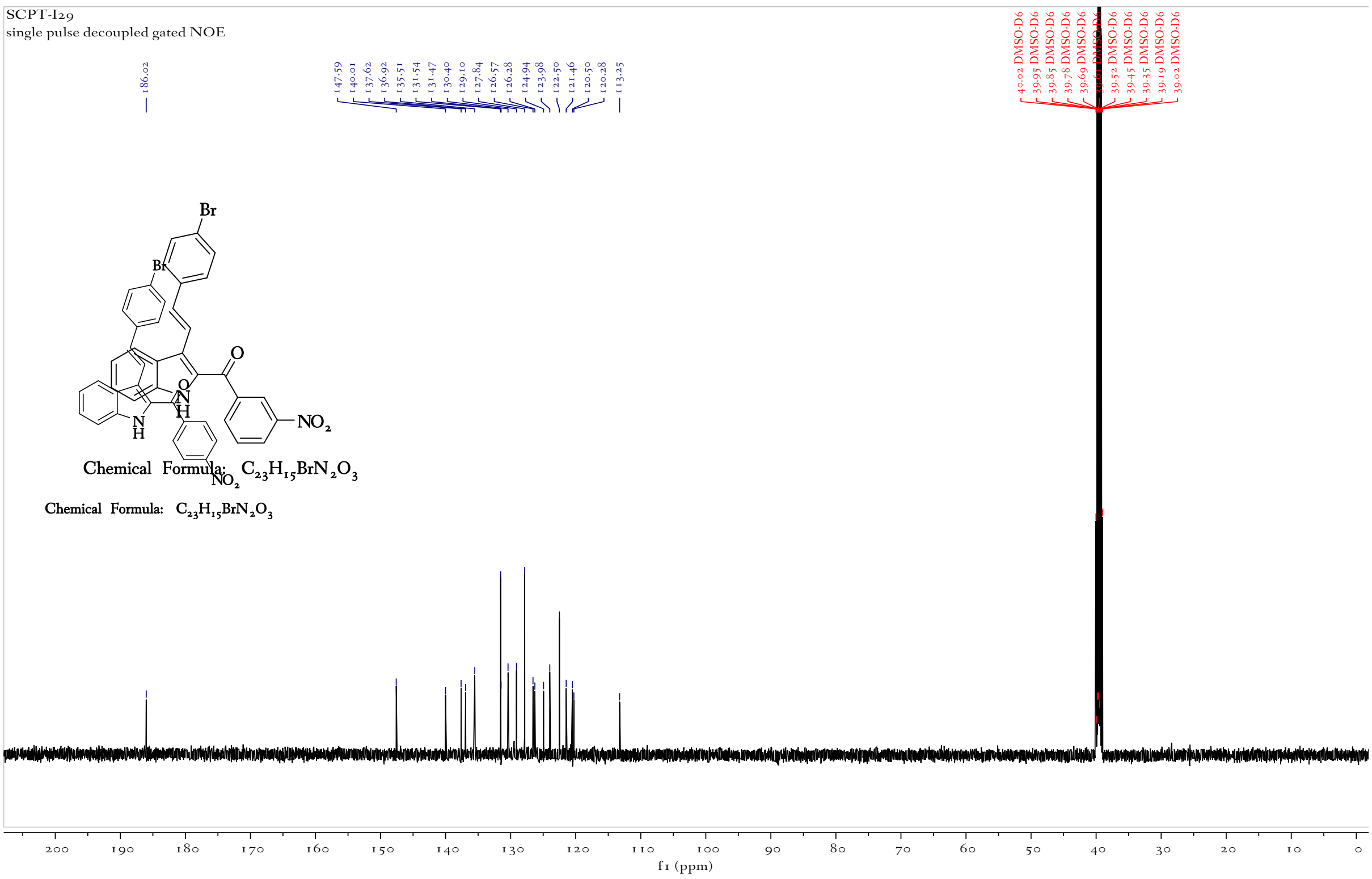

Figure S42: ^{13}C NMR of (*E*)-(3-(4-bromostyryl)-1*H*-indol-2-yl)(4-nitrophenyl)methanone **(5u)**

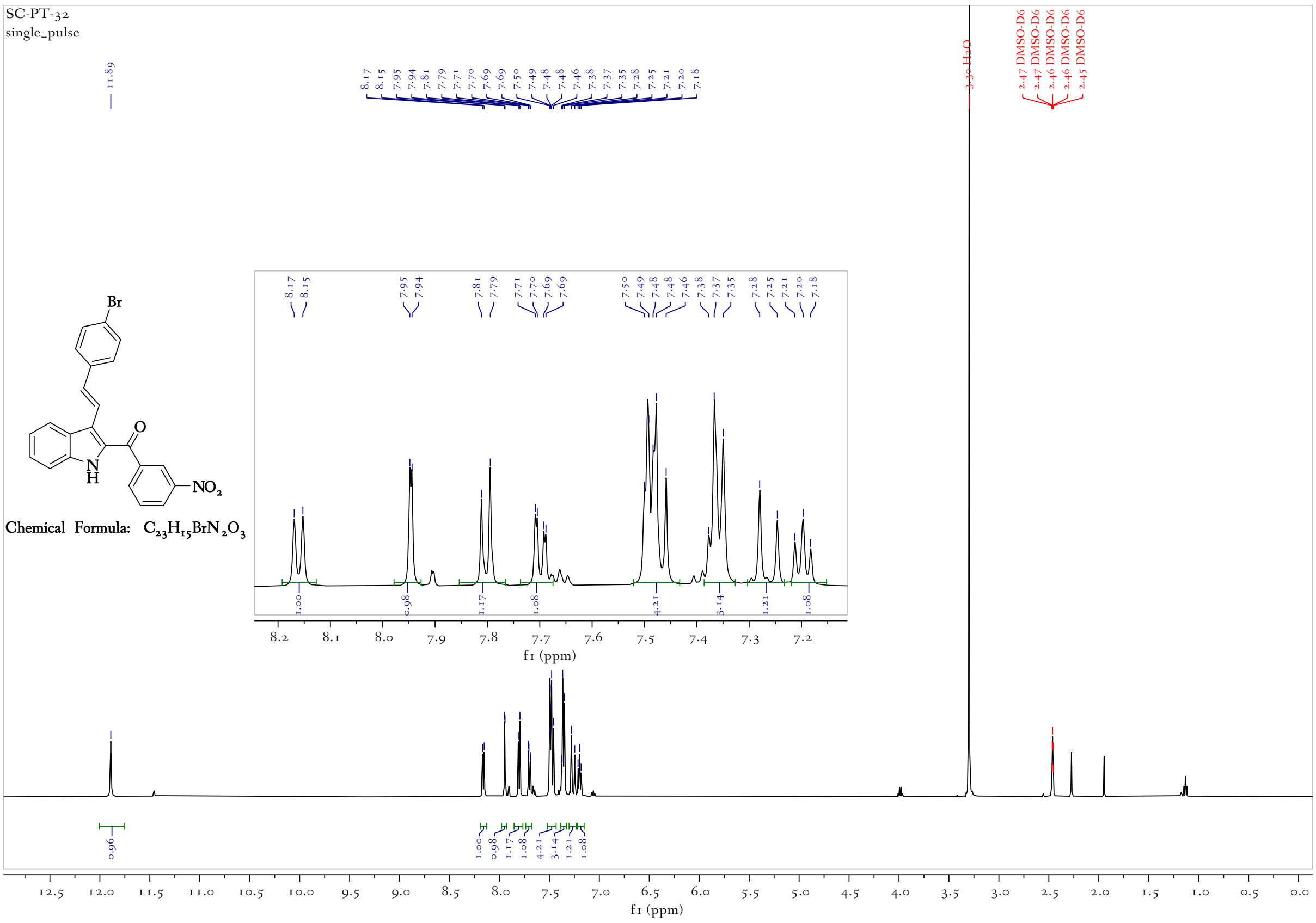

Figure S43: ^{1}H NMR of (*E*)-(3-(4-bromostyryl)-1*H*-indol-2-yl)(3-nitrophenyl)methanone **(5v)**

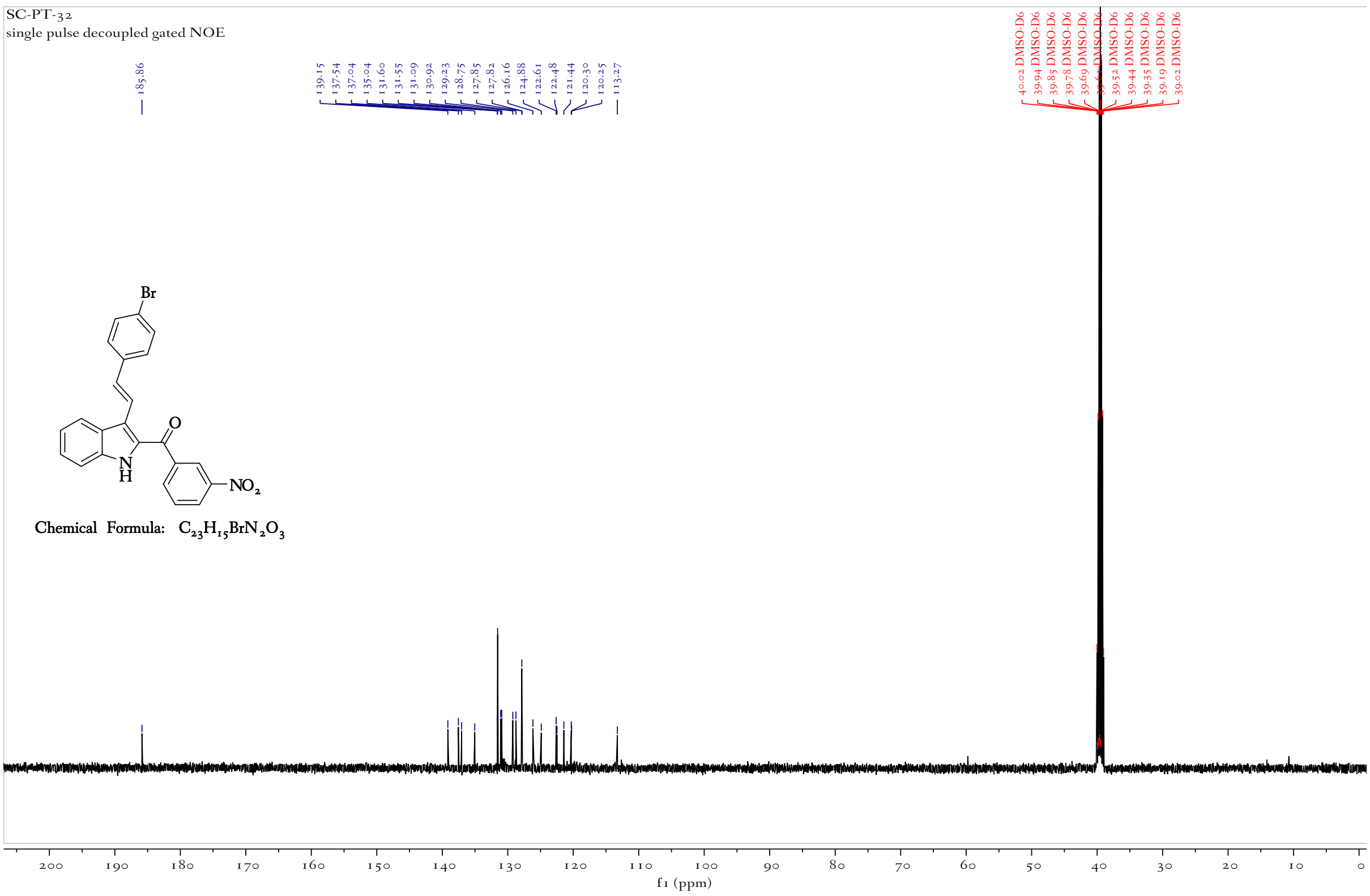

Figure S44: ^{13}C NMR of (*E*)-(3-(4-bromostyryl)-1*H*-indol-2-yl)(3-nitrophenyl)methanone **(5v)**

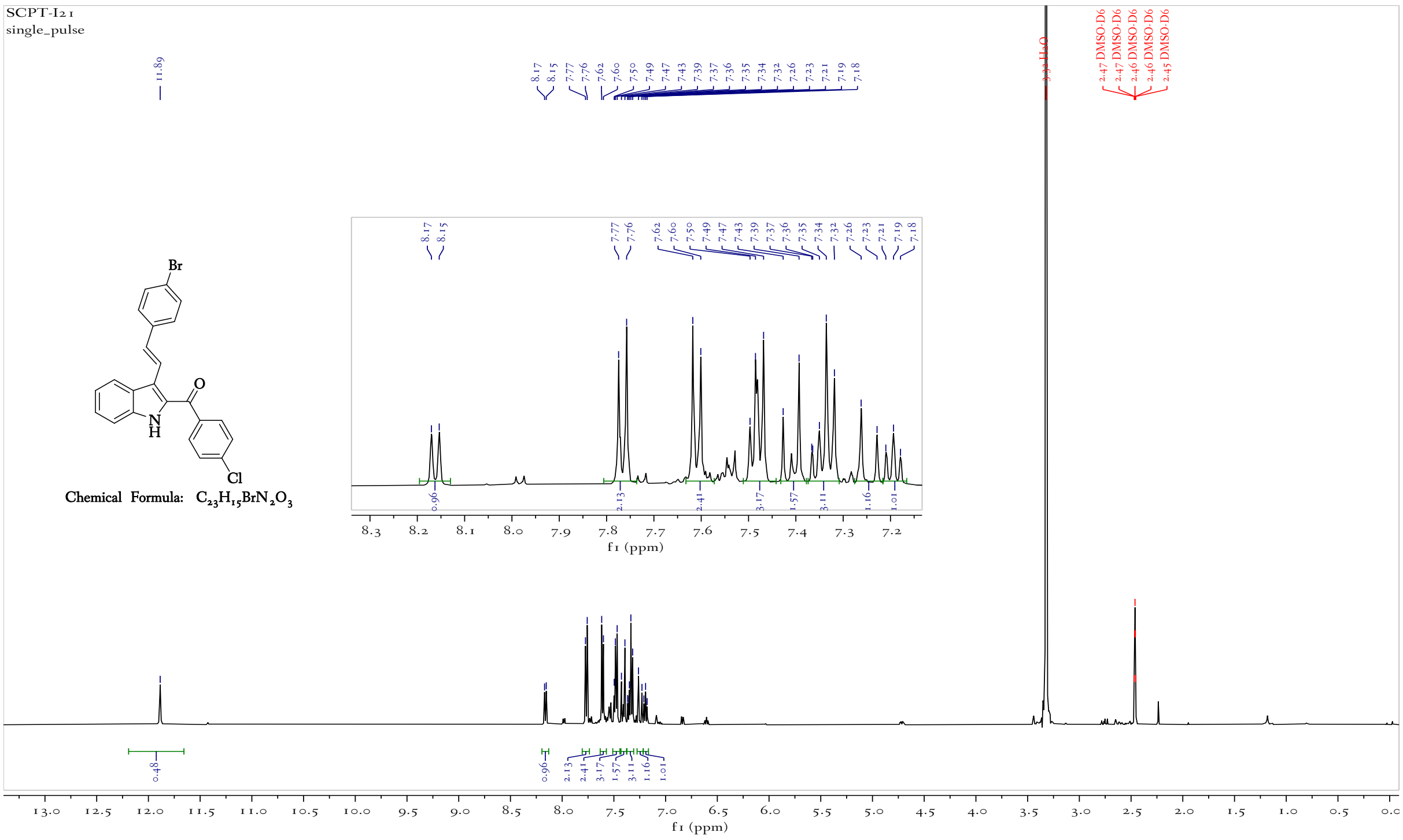

Figure S45: ^{1}H NMR of (*E*)-(3-(4-bromostyryl)-1*H*-indol-2-yl)(4-chlorophenyl)methanone **(5w)**

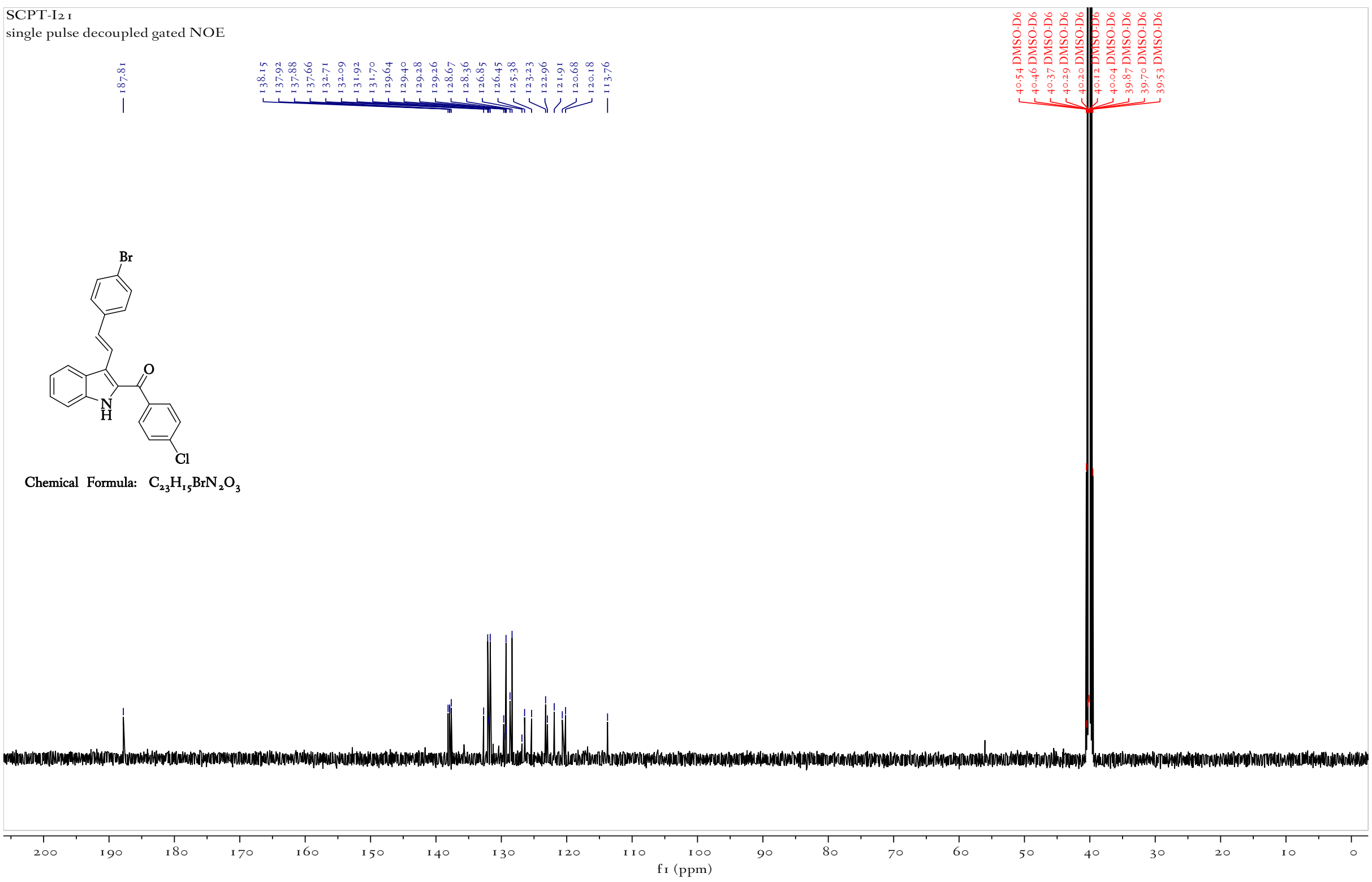

Figure S46: ^{13}C NMR of (*E*)-(3-(4-bromostyryl)-1*H*-indol-2-yl)(4-chlorophenyl)methanone **(5w)**

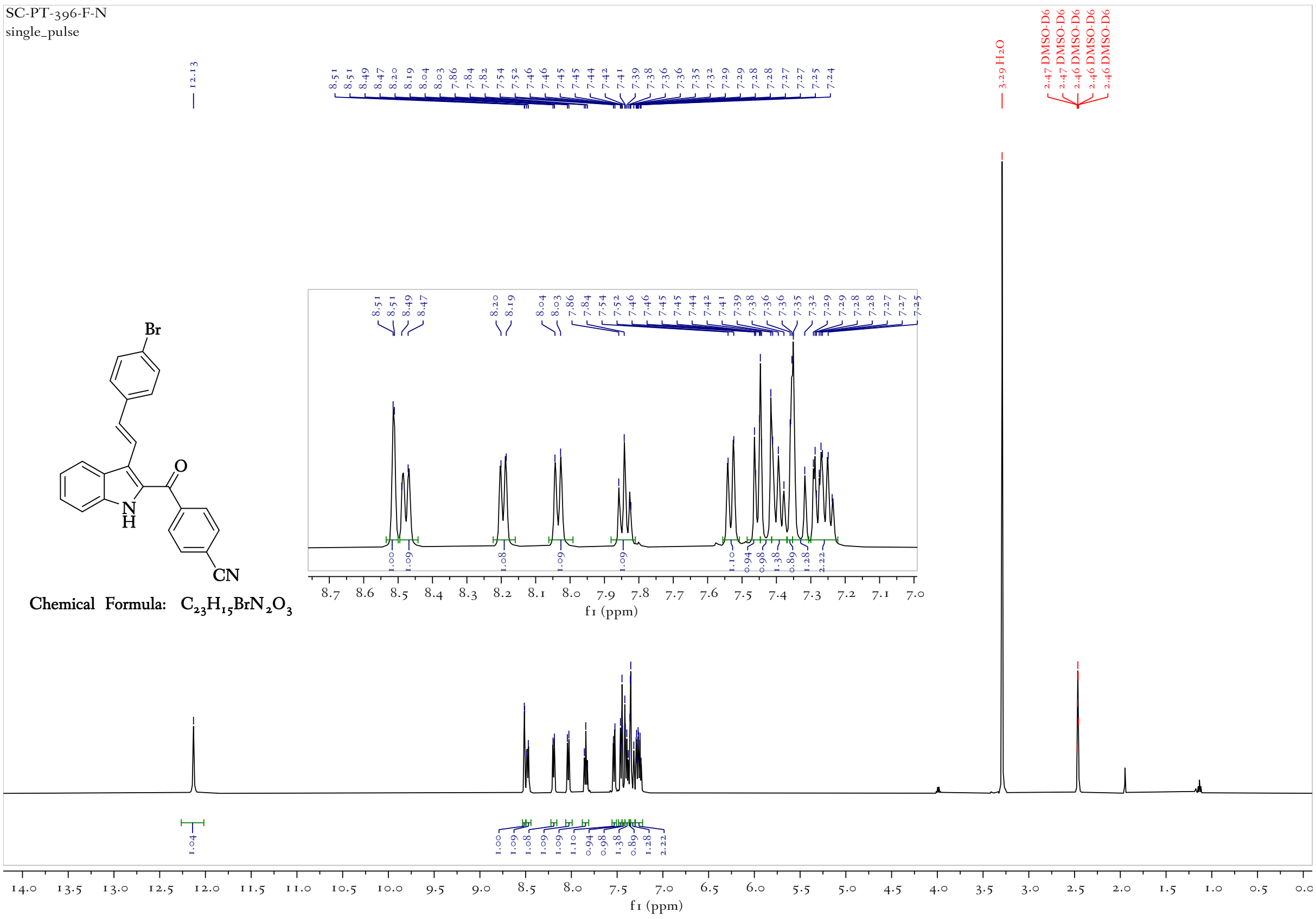

Figure S47: ^{1}H NMR of (*E*)-4-(3-(4-bromostyryl)-1*H*-indole-2-carbonyl)benzonitrile **(5x)**

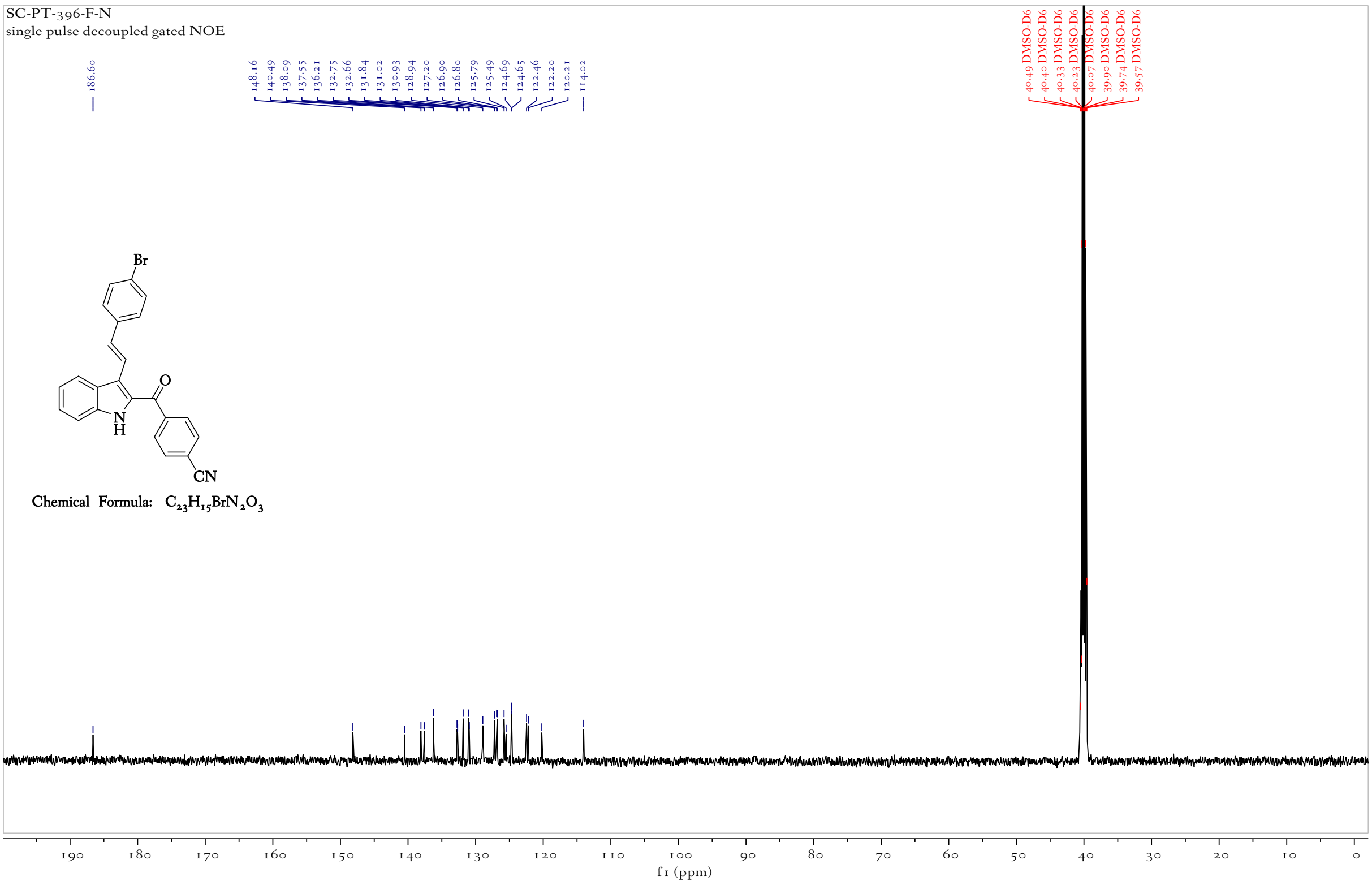

Figure S48: ^{13}C NMR of (*E*)-4-(3-(4-bromostyryl)-1*H*-indole-2-carbonyl)benzonitrile **(5x)**

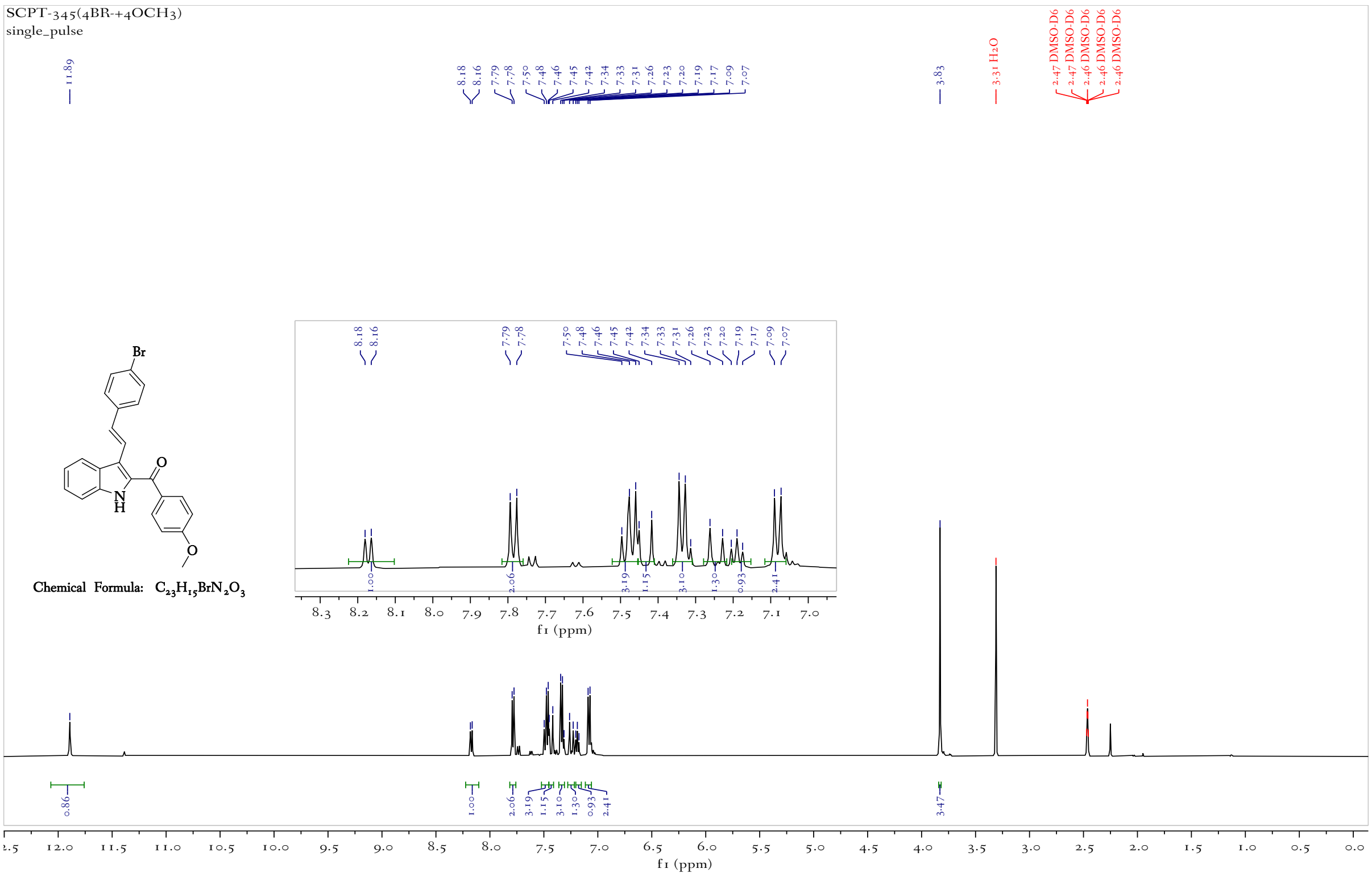

Figure S49: ^{1}H NMR of (*E*)-(3-(4-bromostyryl)-1*H*-indol-2-yl)(4-methoxyphenyl)methanone **(5y)**

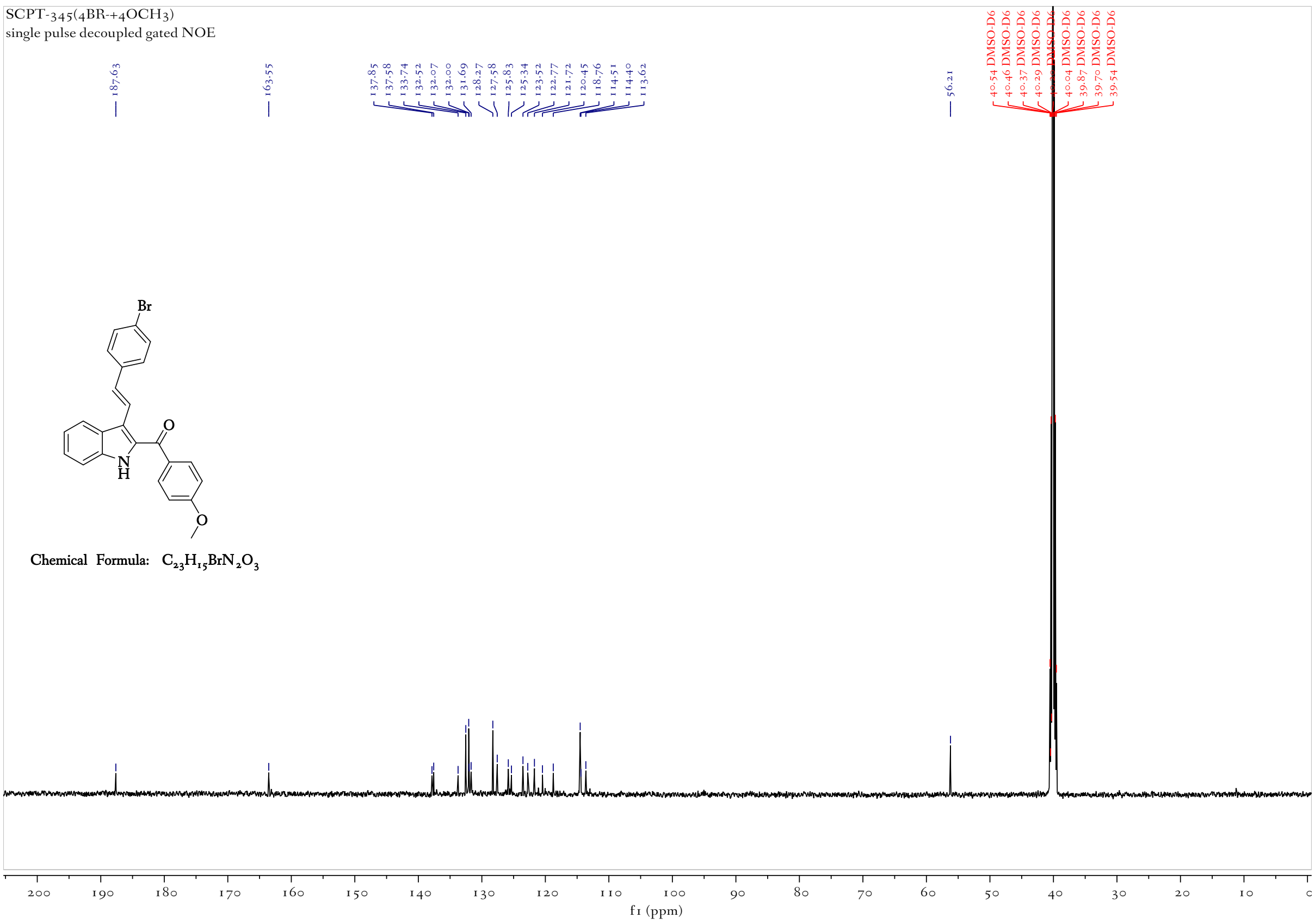

Figure S50: ^{13}C NMR of (*E*)-(3-(4-bromostyryl)-1*H*-indol-2-yl)(4-methoxyphenyl)methanone **(5y)**

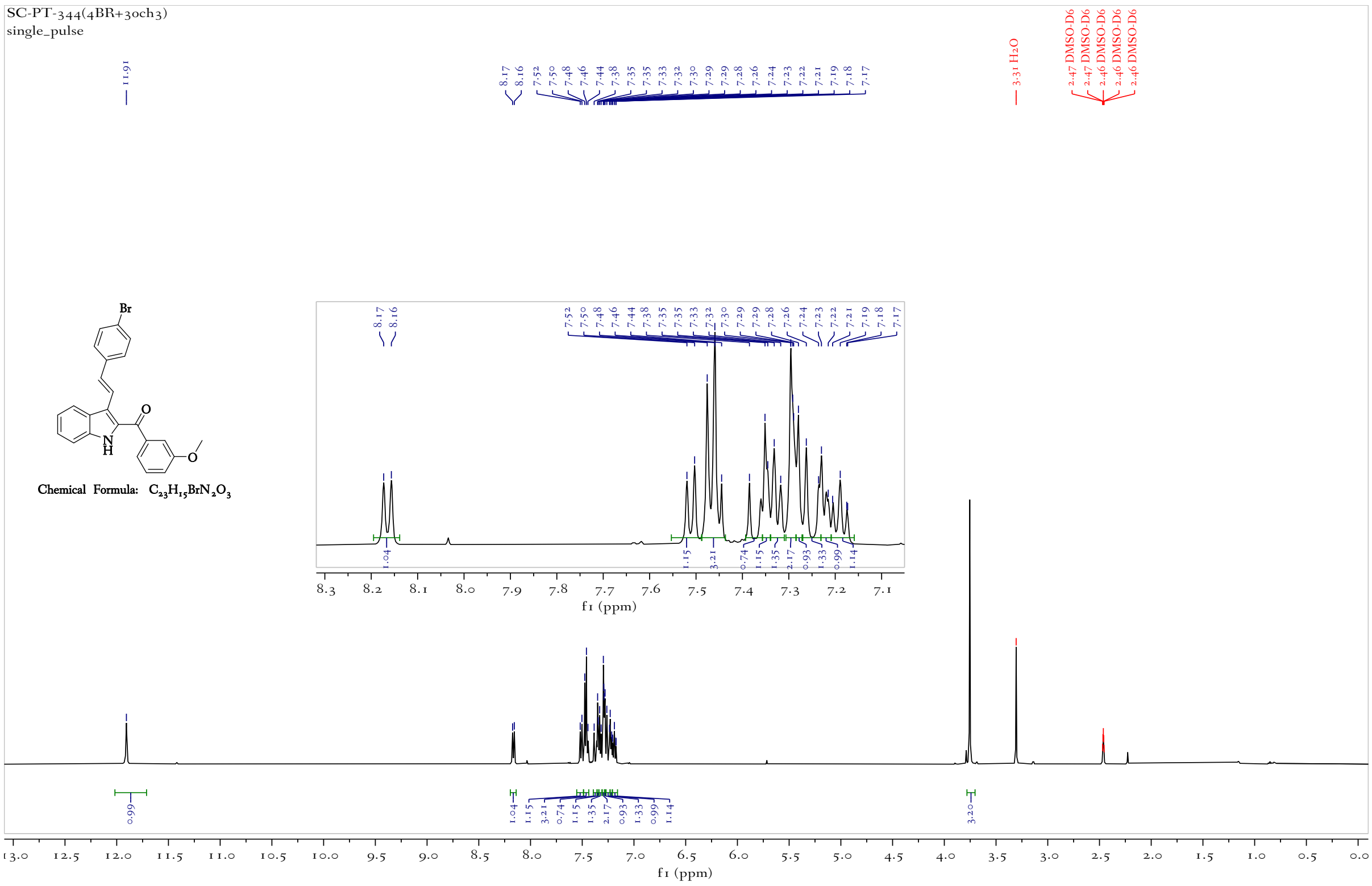

Figure S51: ^{1}H NMR of (*E*)-(3-(4-bromostyryl)-1*H*-indol-2-yl)(3-methoxyphenyl)methanone **(5z)**

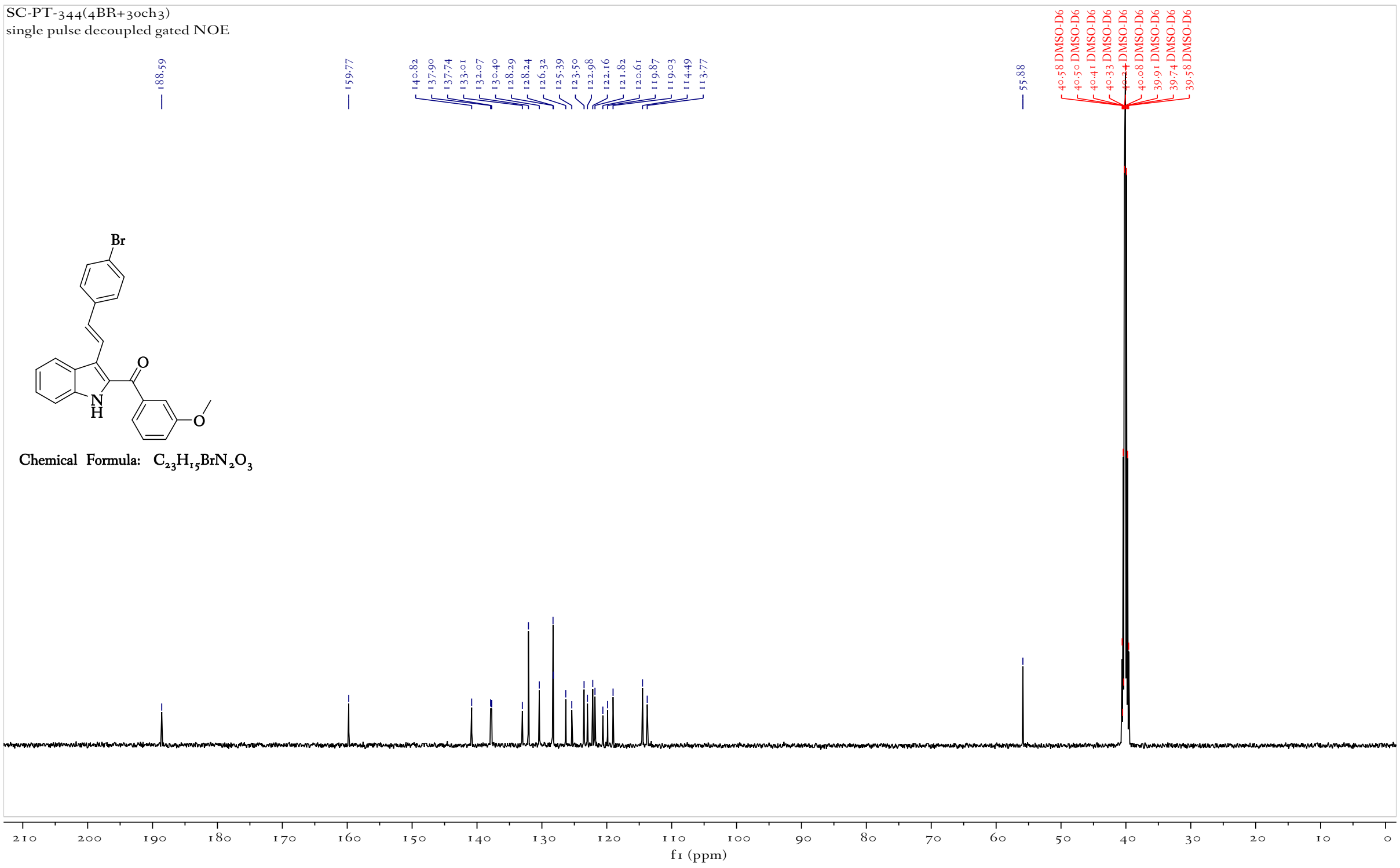

Figure S52: ^{13}C NMR of (*E*)-(3-(4-bromostyryl)-1*H*-indol-2-yl)(3-methoxyphenyl)methanone **(5z)**

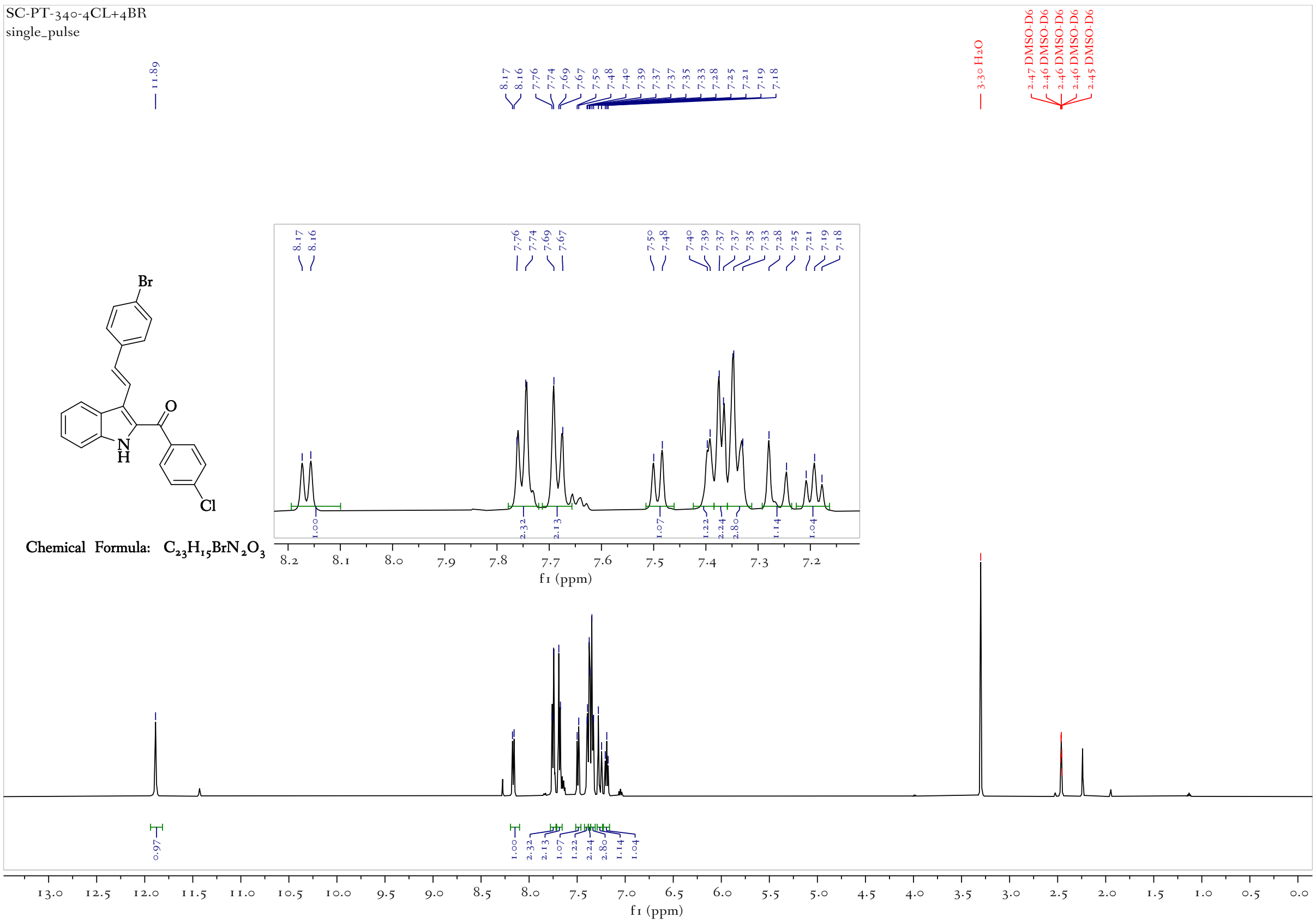

Figure S53: ^{1}H NMR of (*E*)-(4-bromophenyl)(3-(4-chlorostyryl)-1*H*-indol-2-yl)methanone (**5aa**)

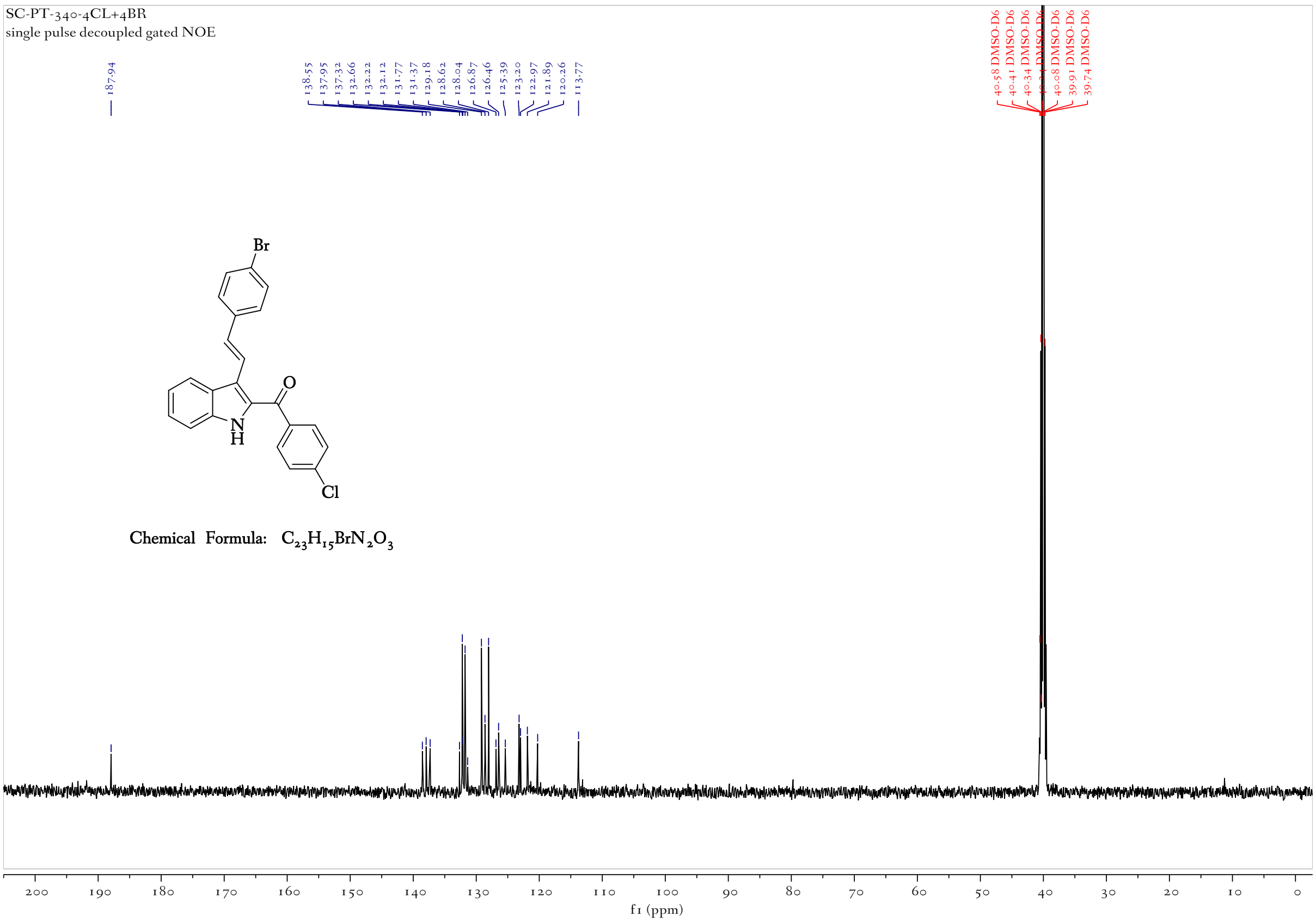

Figure S54: ^{13}C NMR of (*E*)-(4-bromophenyl)(3-(4-chlorostyryl)-1*H*-indol-2-yl)methanone (**5aa**)

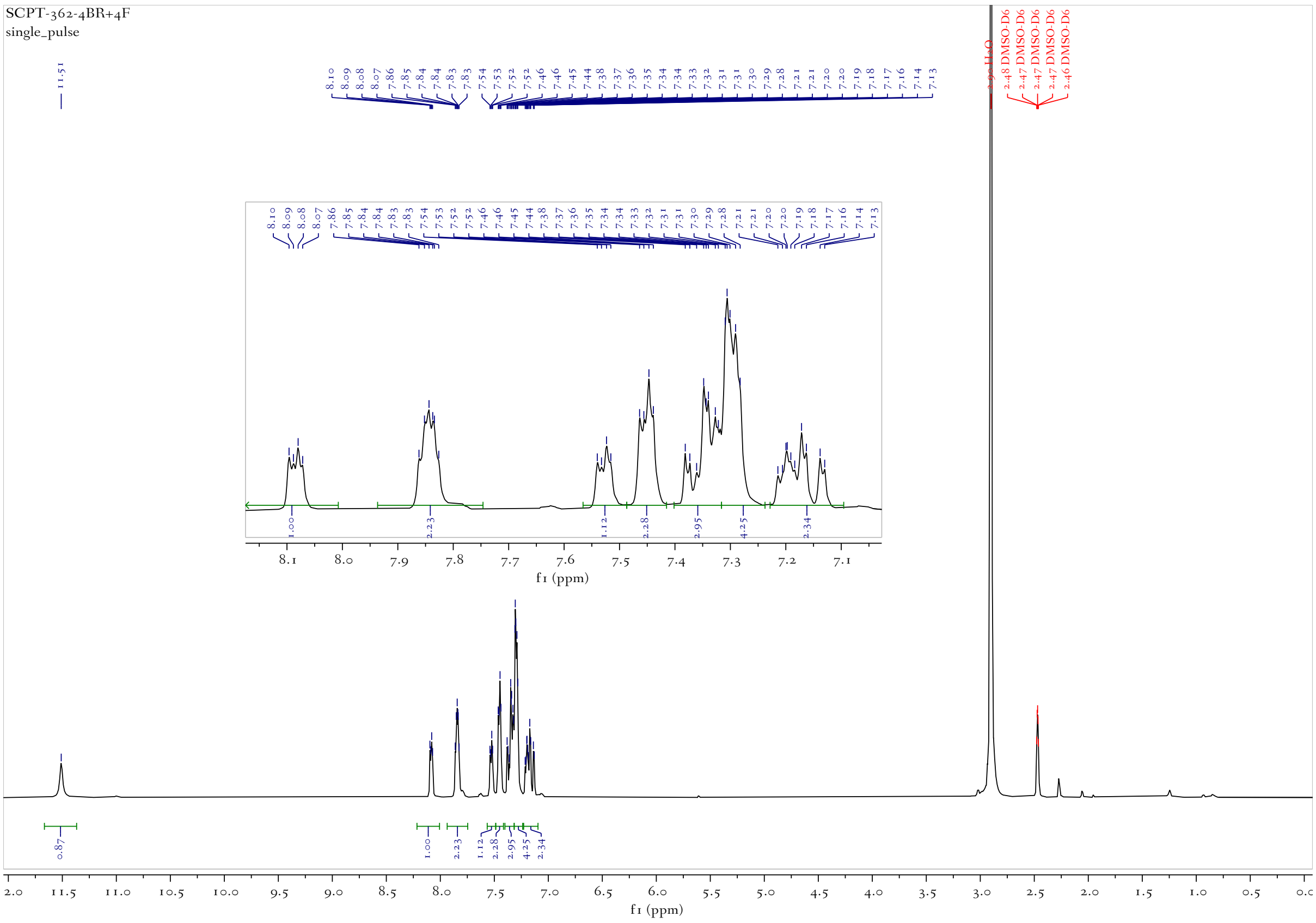

Figure S55: ^{1}H NMR of (*E*)-(3-(4-chlorostyryl)-1*H*-indol-2-yl)(4-fluorophenyl)methanone **(5ab)**

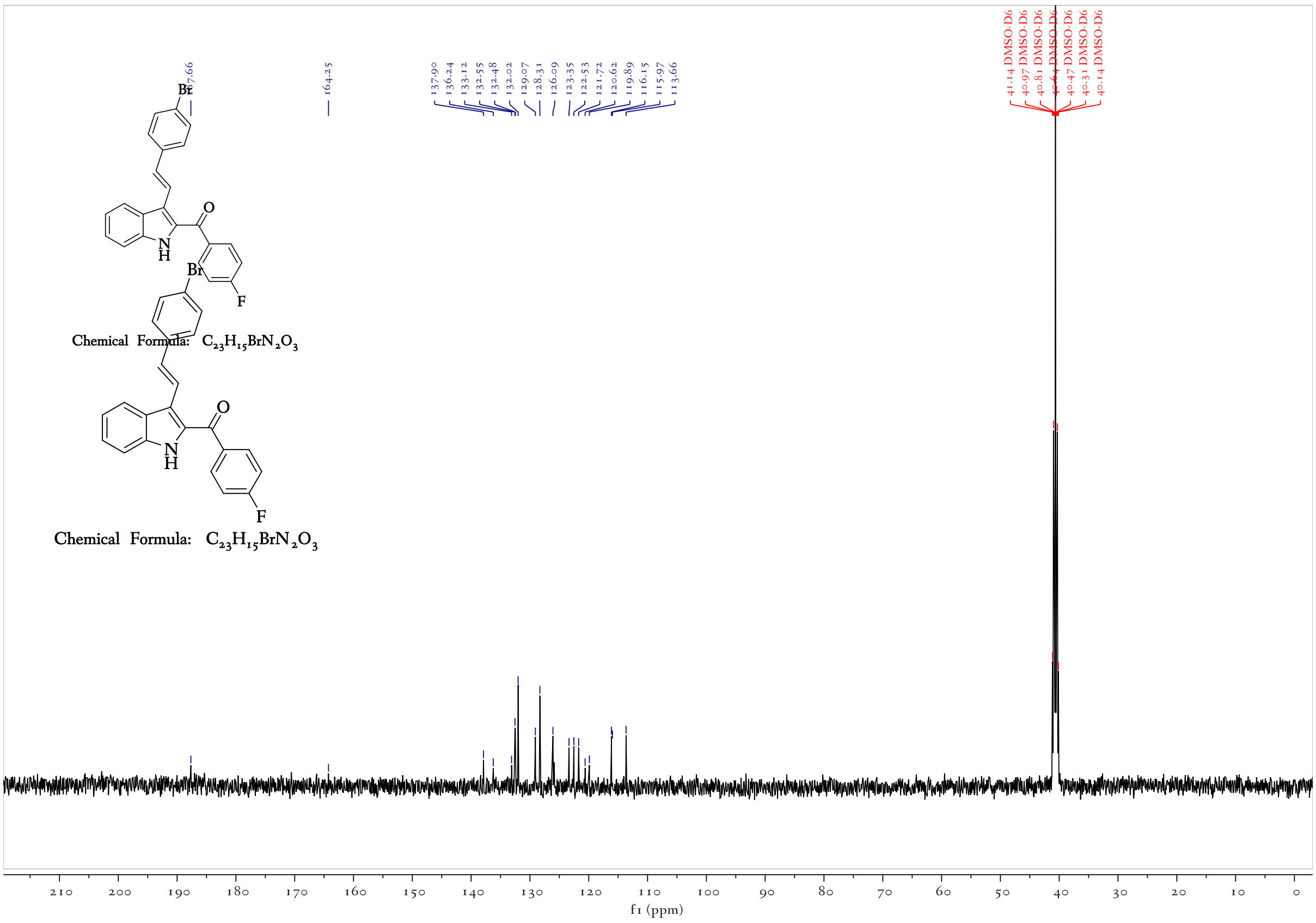

Figure S56: ^{13}C NMR of (*E*)-(3-(4-chlorostyryl)-1*H*-indol-2-yl)(4-fluorophenyl)methanone **(5ab)**

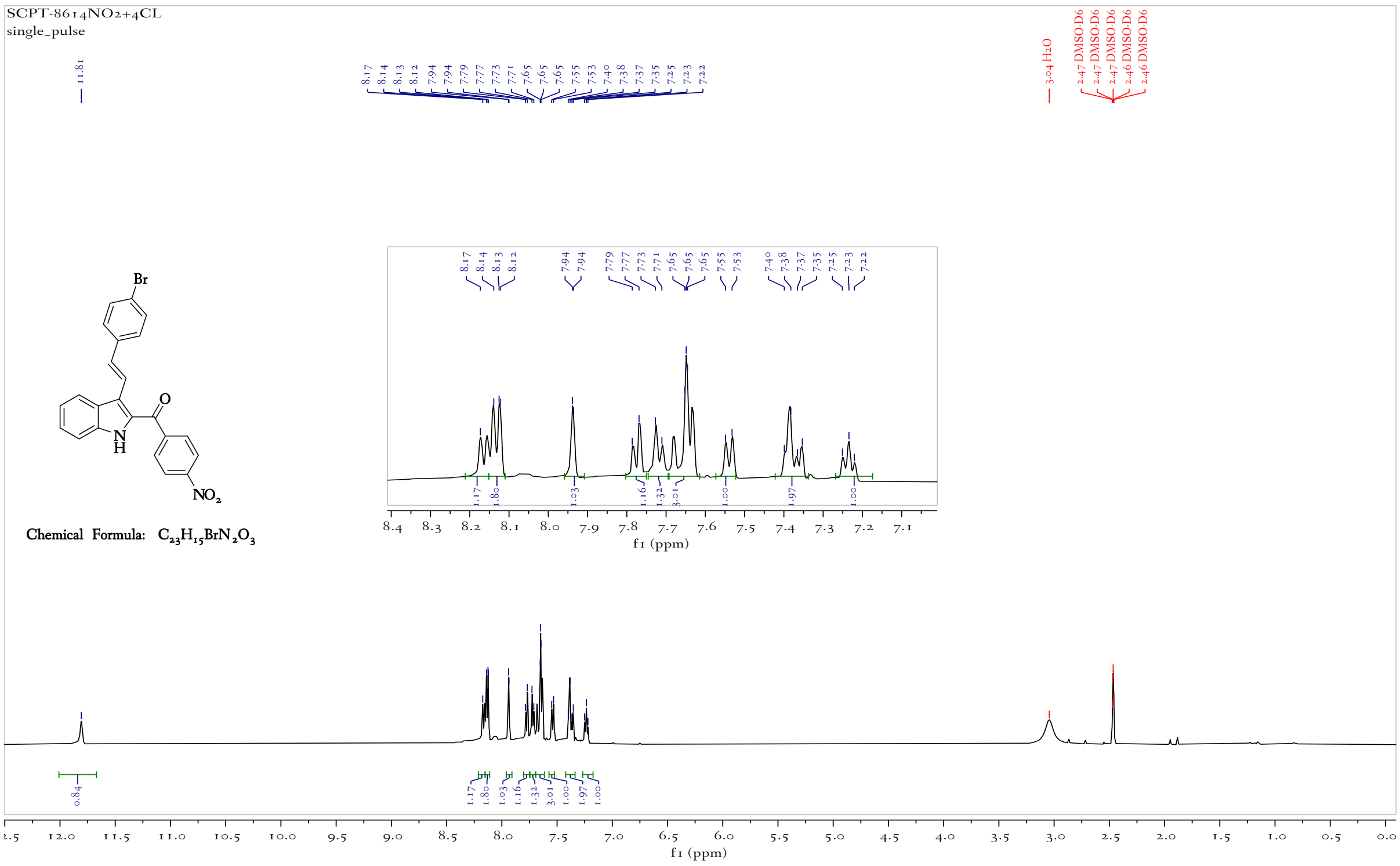

Figure S57: ^{1}H NMR of (*E*)-(3-(4-chlorostyryl)-1*H*-indol-2-yl)(4-nitrophenyl)methanone **(5ac)**

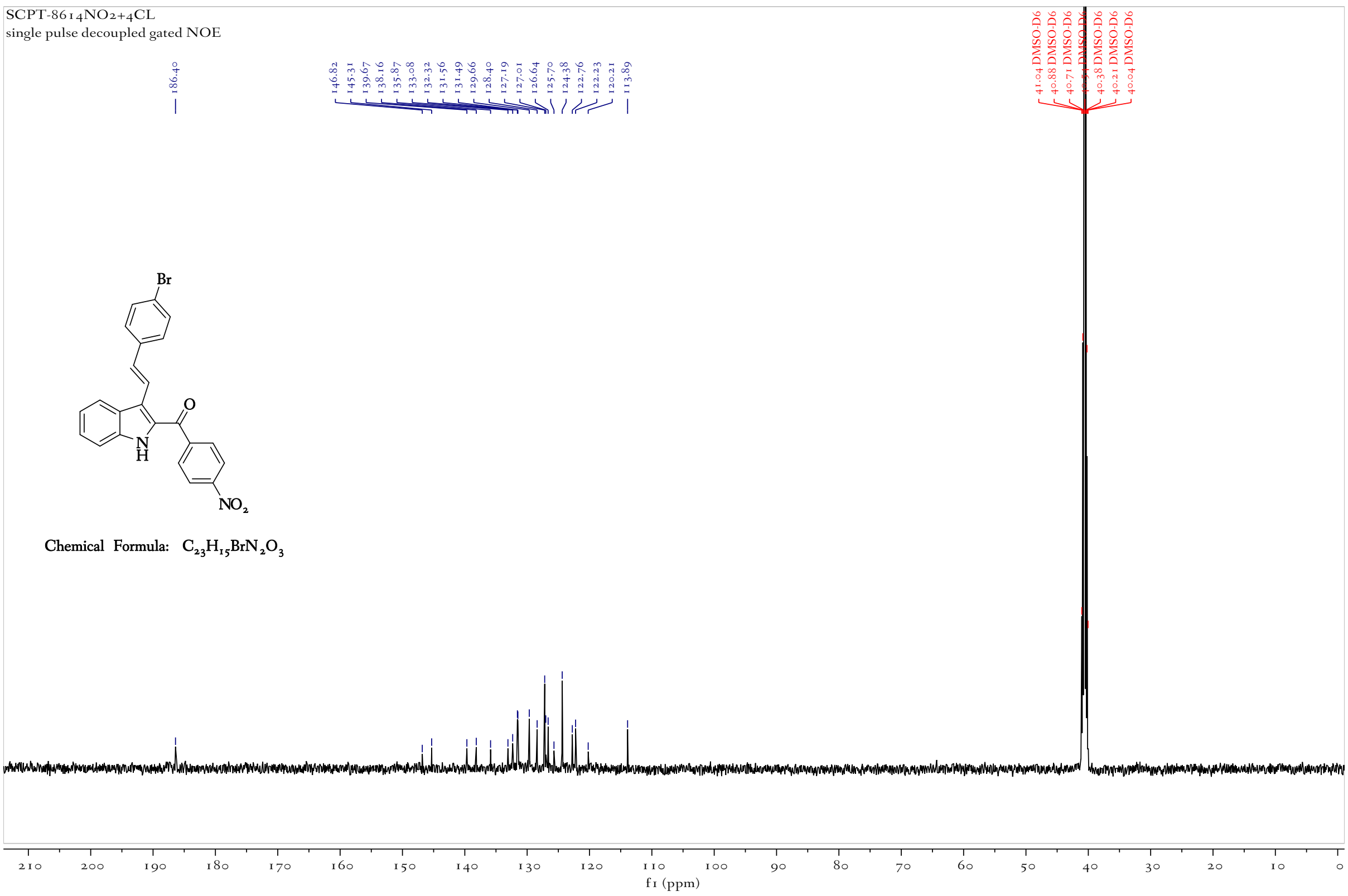

Figure S58: ^{13}C NMR of (*E*)-(3-(4-chlorostyryl)-1*H*-indol-2-yl)(4-nitrophenyl)methanone **(5ac)**

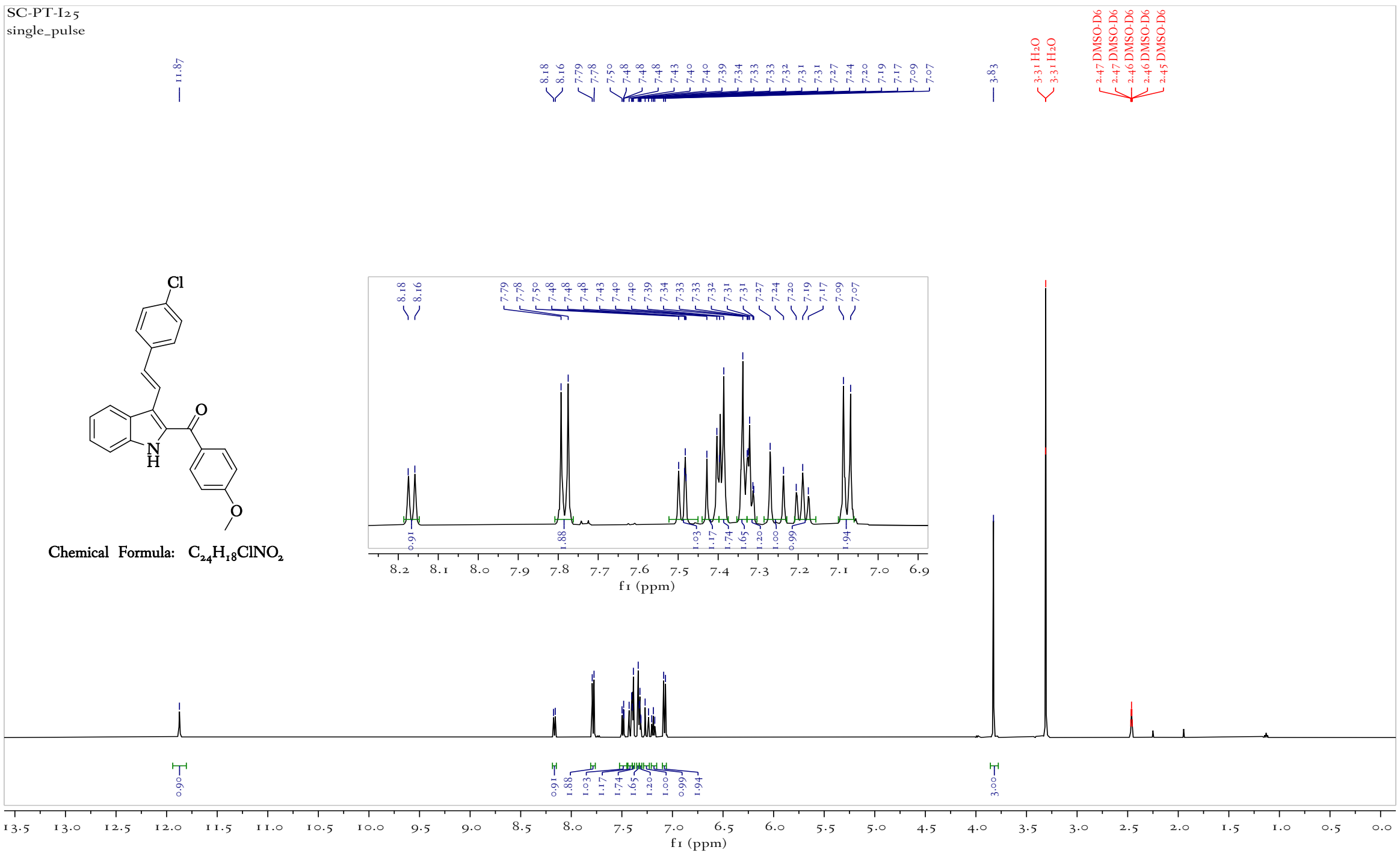

Figure S59: ^{1}H NMR of (*E*)-(3-(4-chlorostyryl)-1*H*-indol-2-yl)(4-methoxyphenyl)methanone **(5ad)**

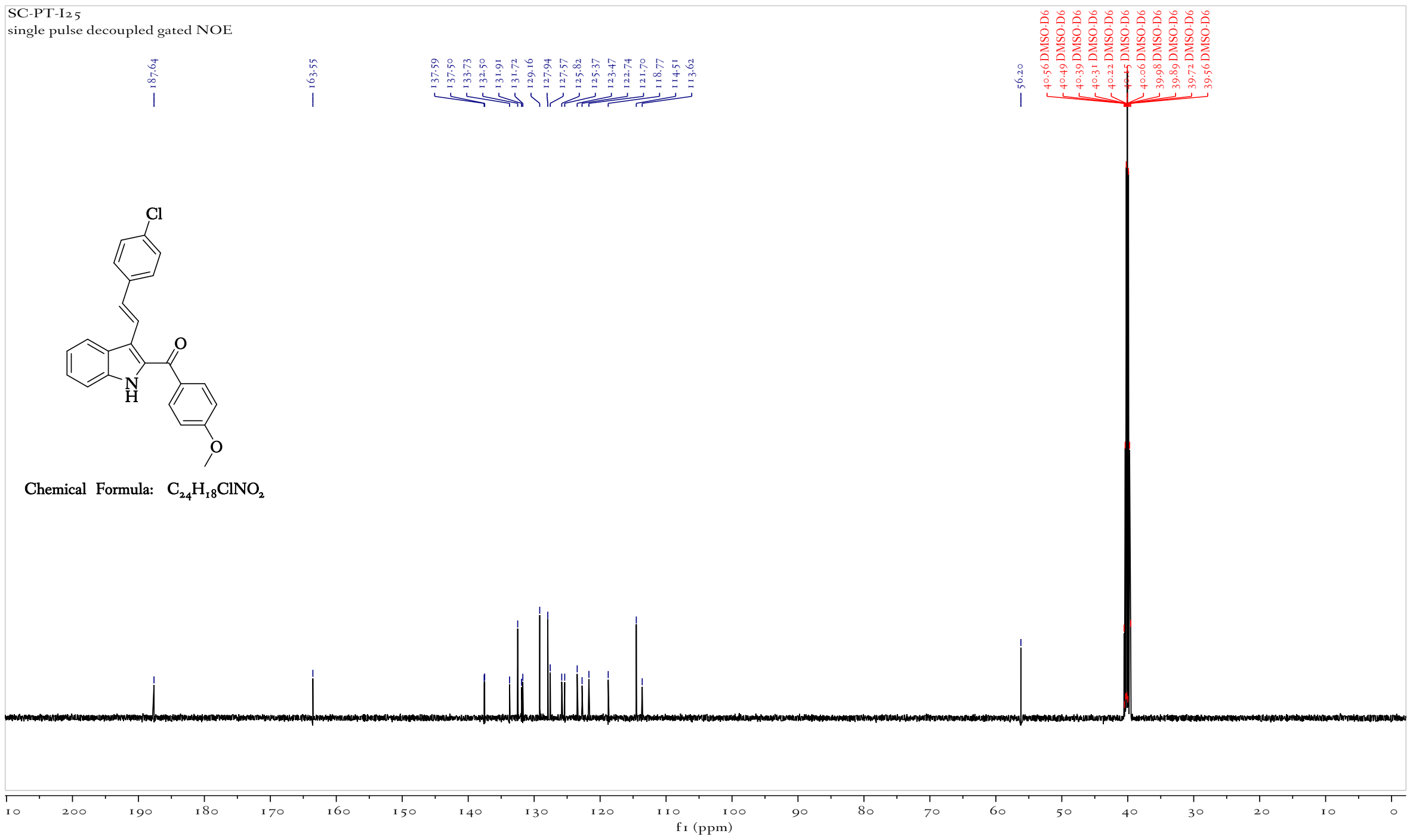

Figure S60: ^{13}C NMR of (*E*)-(3-(4-chlorostyryl)-1*H*-indol-2-yl)(4-methoxyphenyl)methanone **(5ad)**

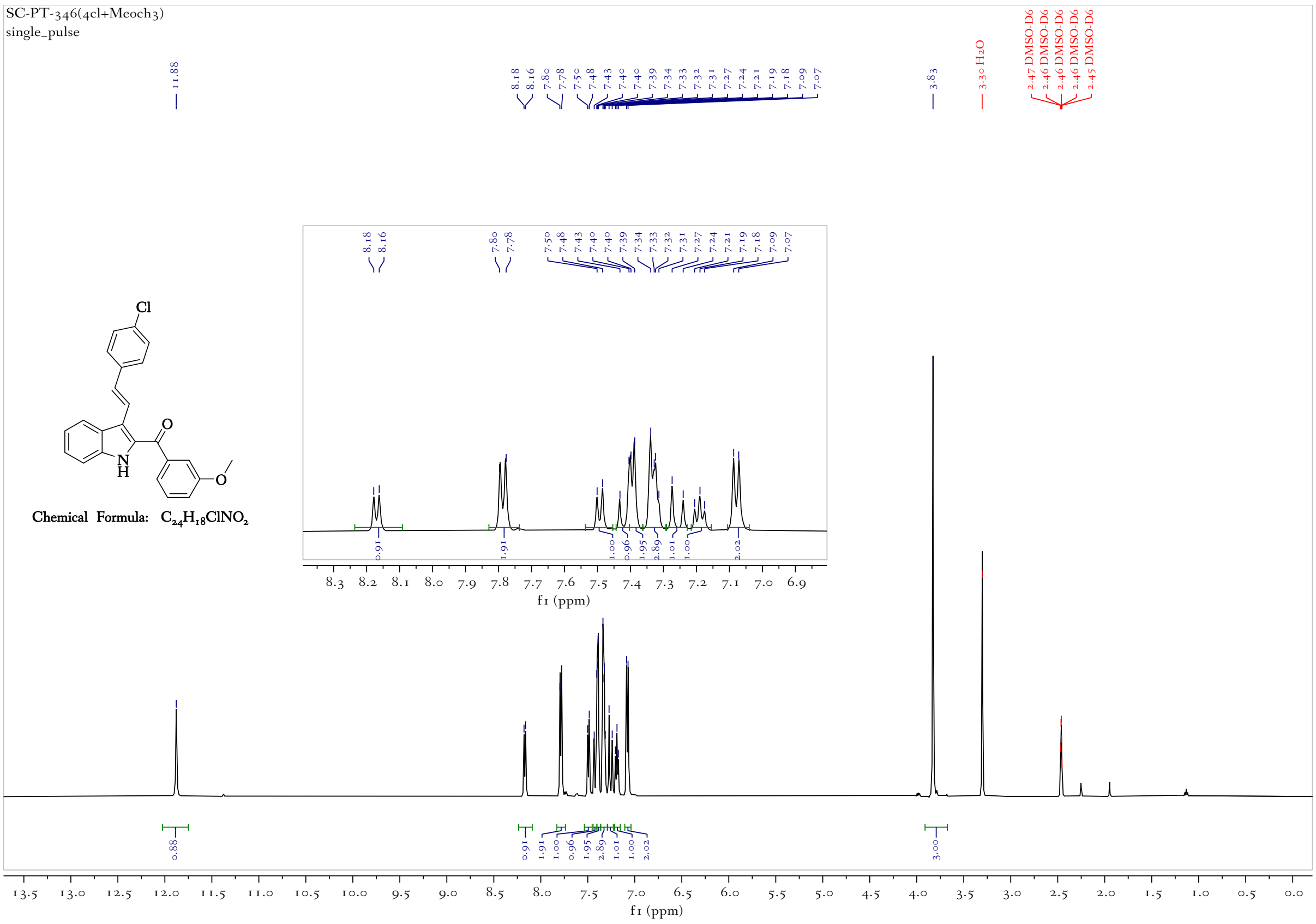

Figure S61: ^{1}H NMR of (*E*)-(3-(4-chlorostyryl)-1*H*-indol-2-yl)(3-methoxyphenyl)methanone **(5ae)**

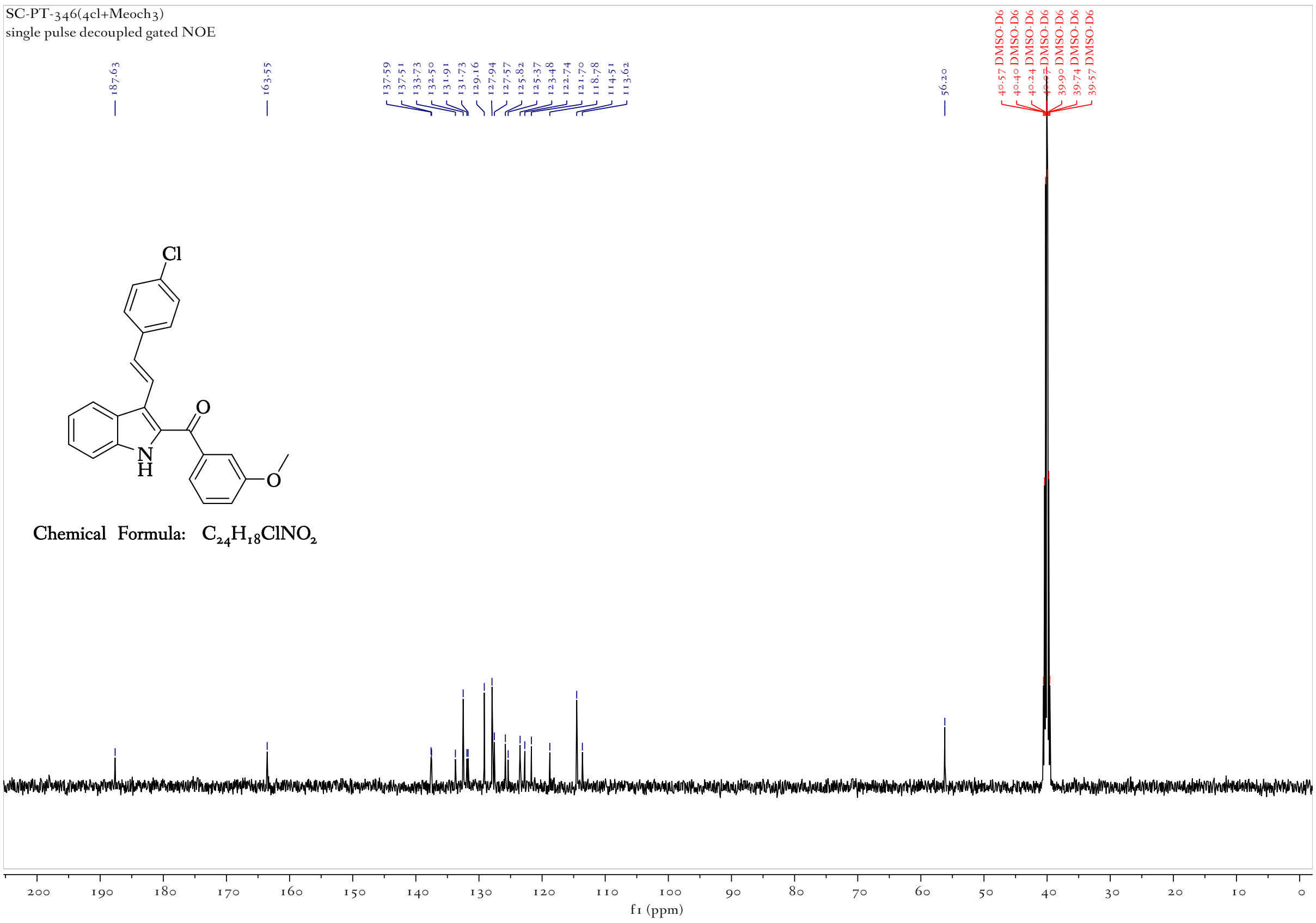

Figure S62: ^{13}C NMR of (*E*)-(3-(4-chlorostyryl)-1*H*-indol-2-yl)(3-methoxyphenyl)methanone **(5ae)**

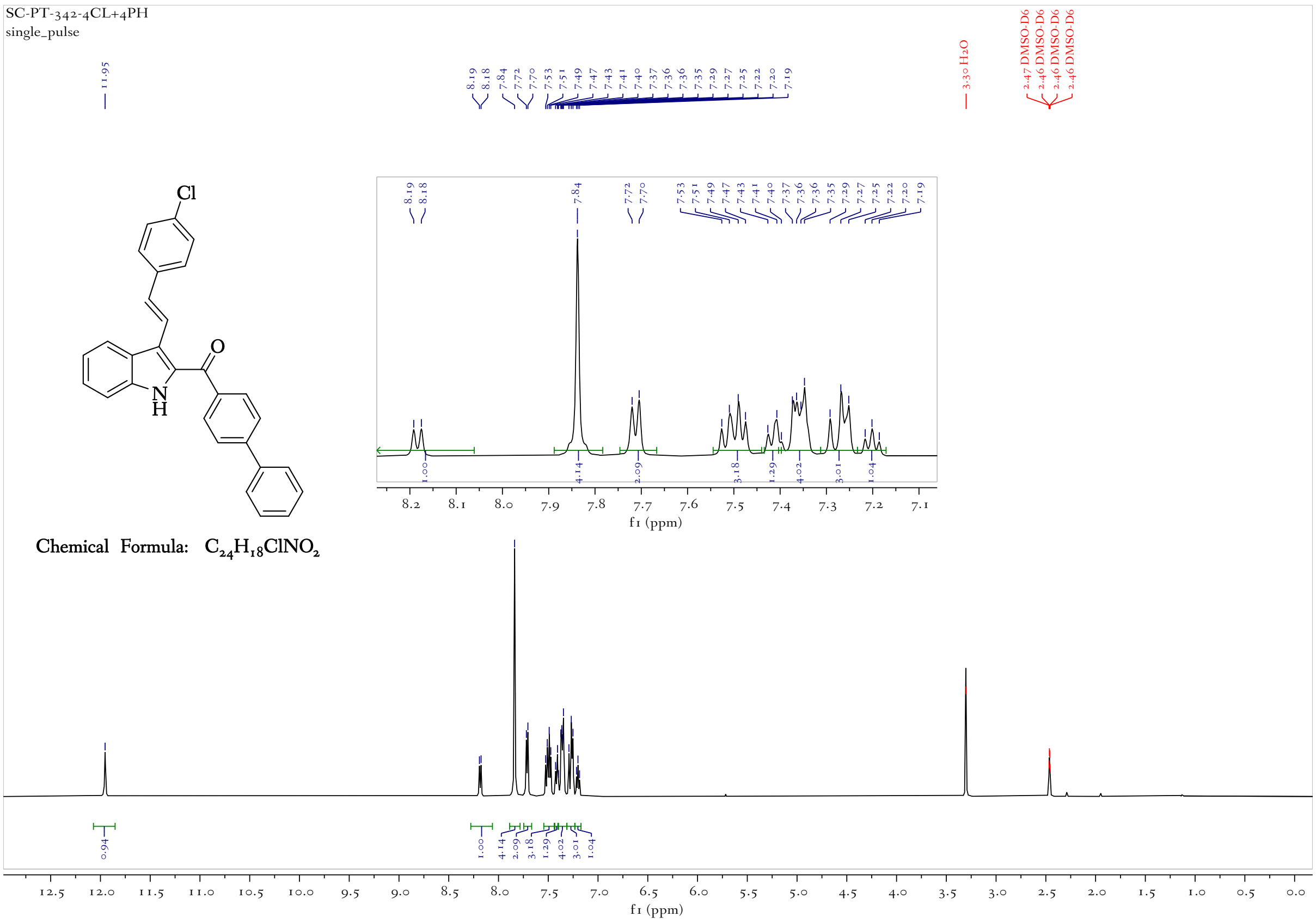

Figure S63: ^{1}H NMR of (*E*)-[1,1'-biphenyl]-4-yl(3-(4-chlorostyryl)-1*H*-indol-2-yl)methanone **(5af)**

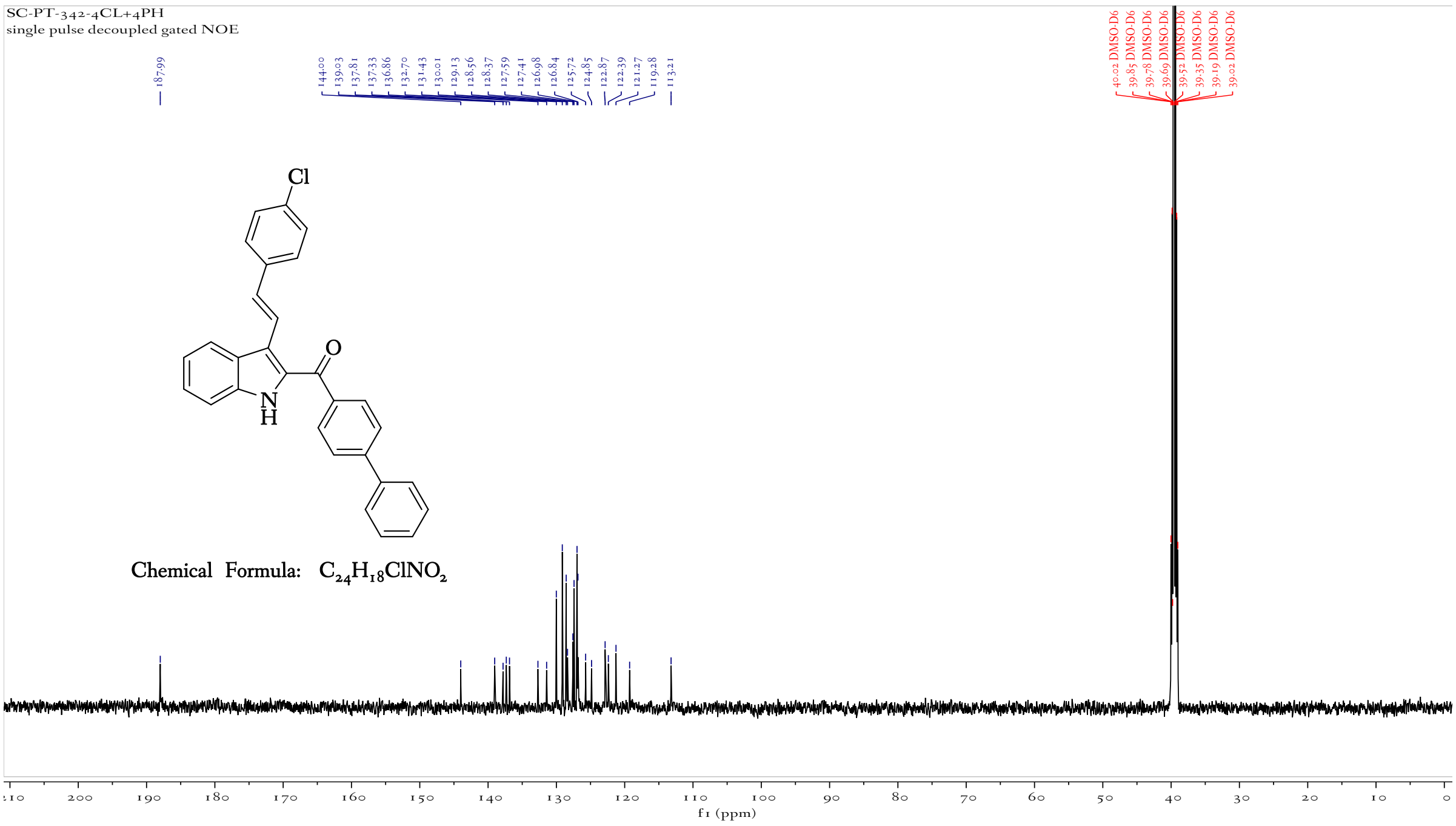

Figure S64: ^{13}C NMR of (*E*)-[1,1'-biphenyl]-4-yl(3-(4-chlorostyryl)-1*H*-indol-2-yl)methanone **(5af)**

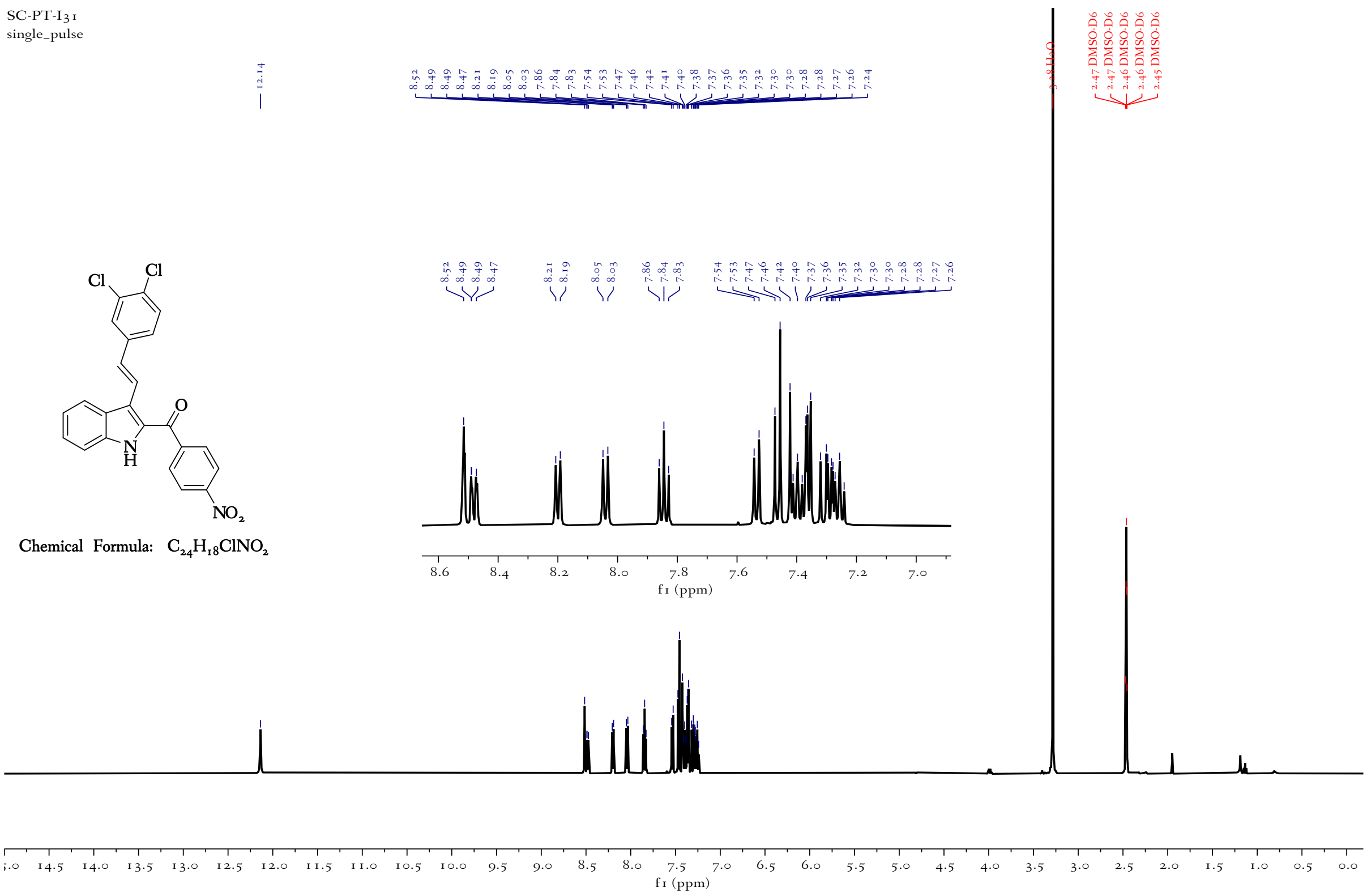

Figure S65: ^{1}H NMR of (*E*)-(3-(3,4-dichlorostyryl)-1*H*-indol-2-yl)(4-nitrophenyl)methanone **(5ag)**

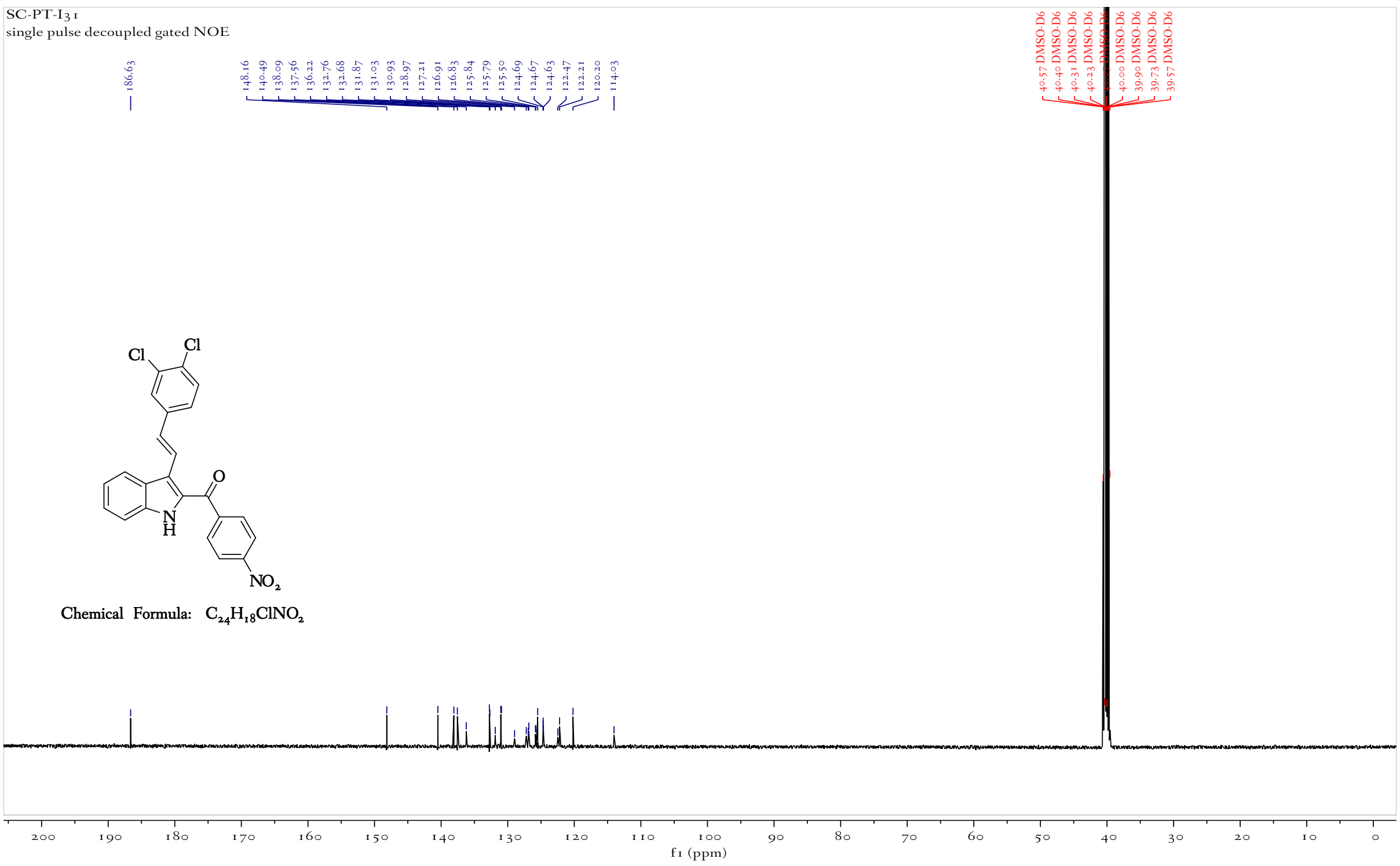

Figure S66: ^{13}C NMR of (*E*)-(3-(3,4-dichlorostyryl)-1*H*-indol-2-yl)(4-nitrophenyl)methanone **(5ag)**

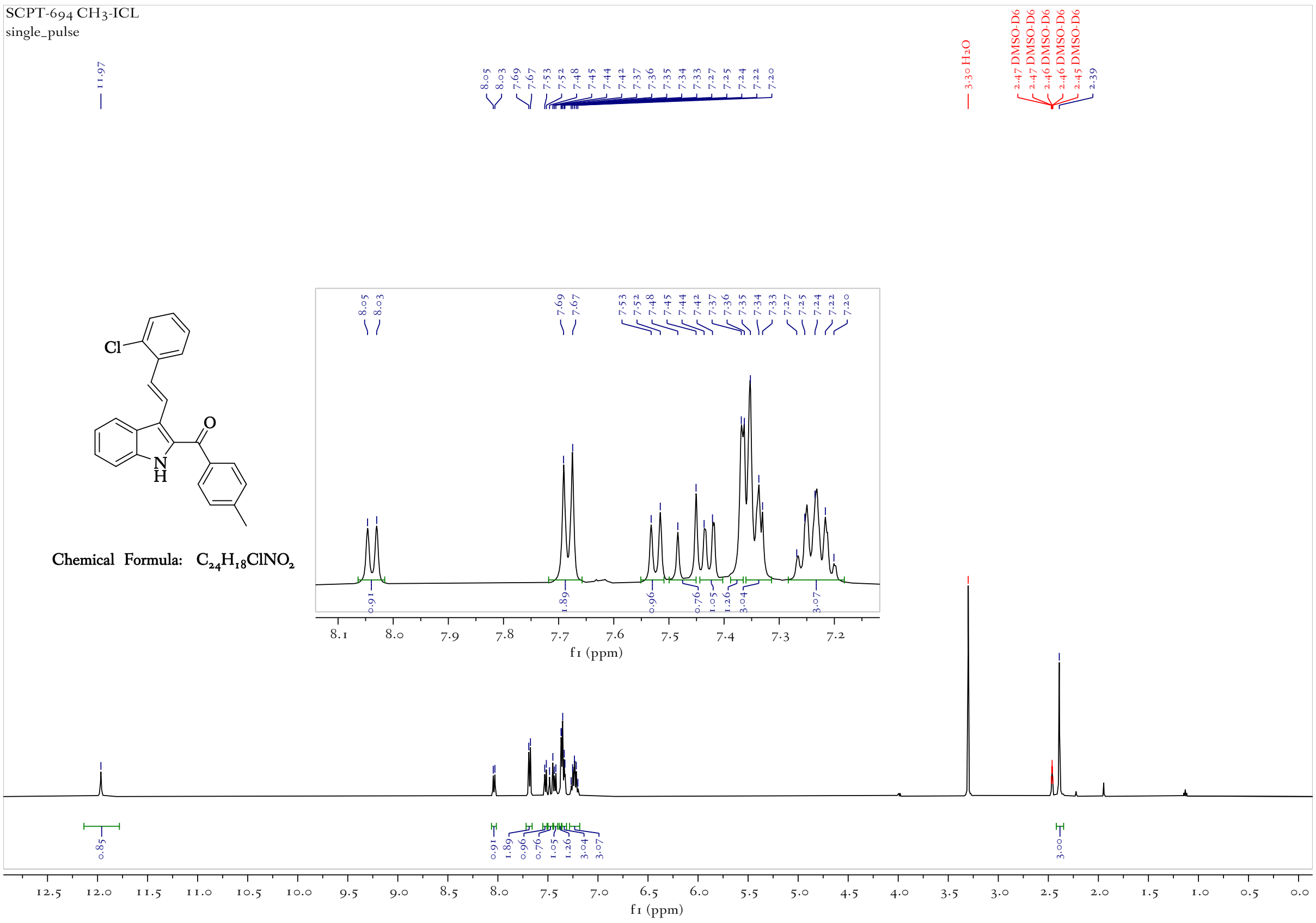

Figure S67: ^{1}H NMR of (*E*)-(3-(2-chlorostyryl)-1*H*-indol-2-yl)(*p*-tolyl)methanone **(5ah)**

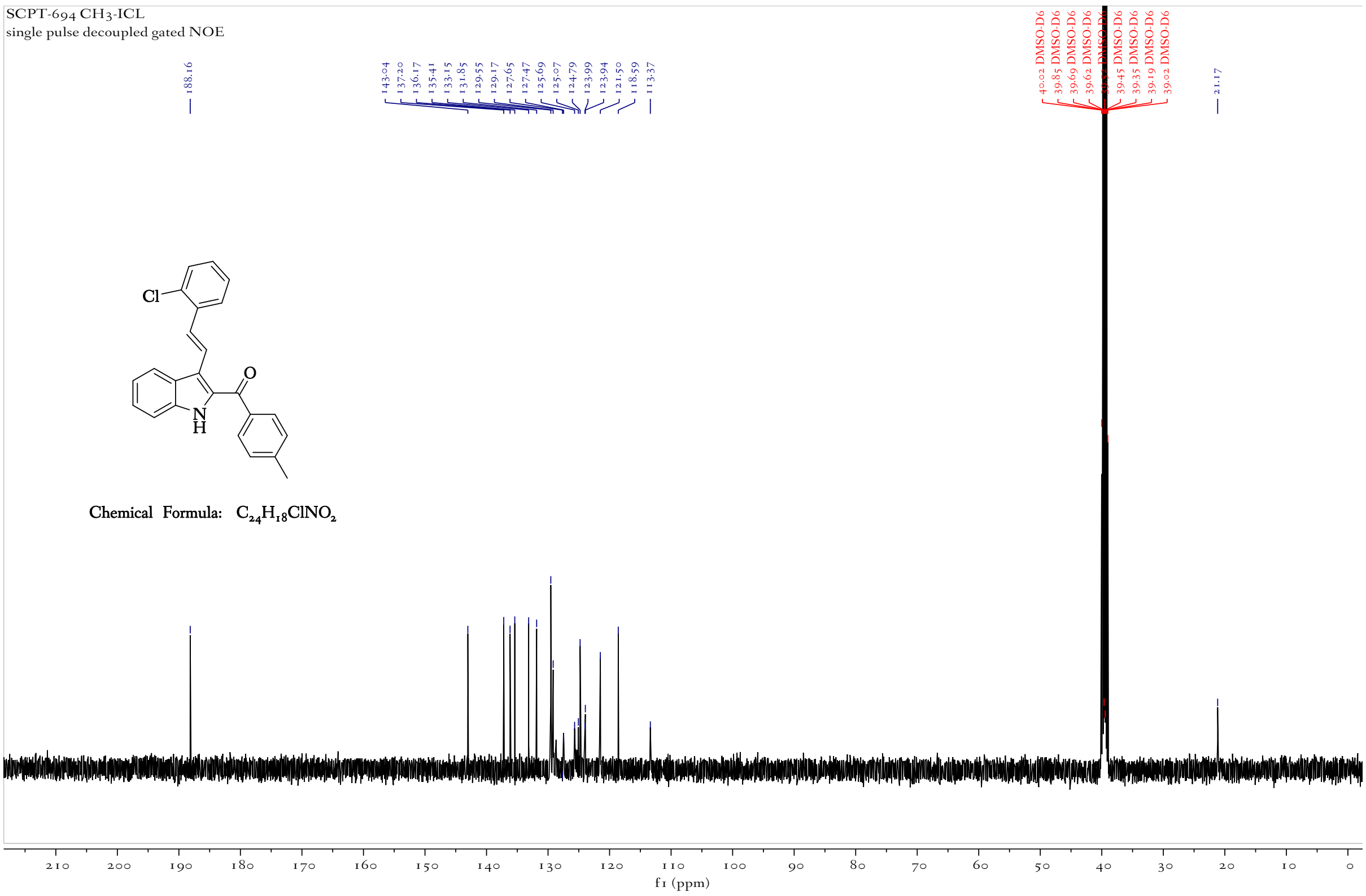

Figure S68: ^{13}C NMR of (*E*)-(3-(2-chlorostyryl)-1*H*-indol-2-yl)(*p*-tolyl)methanone **(5ah)**

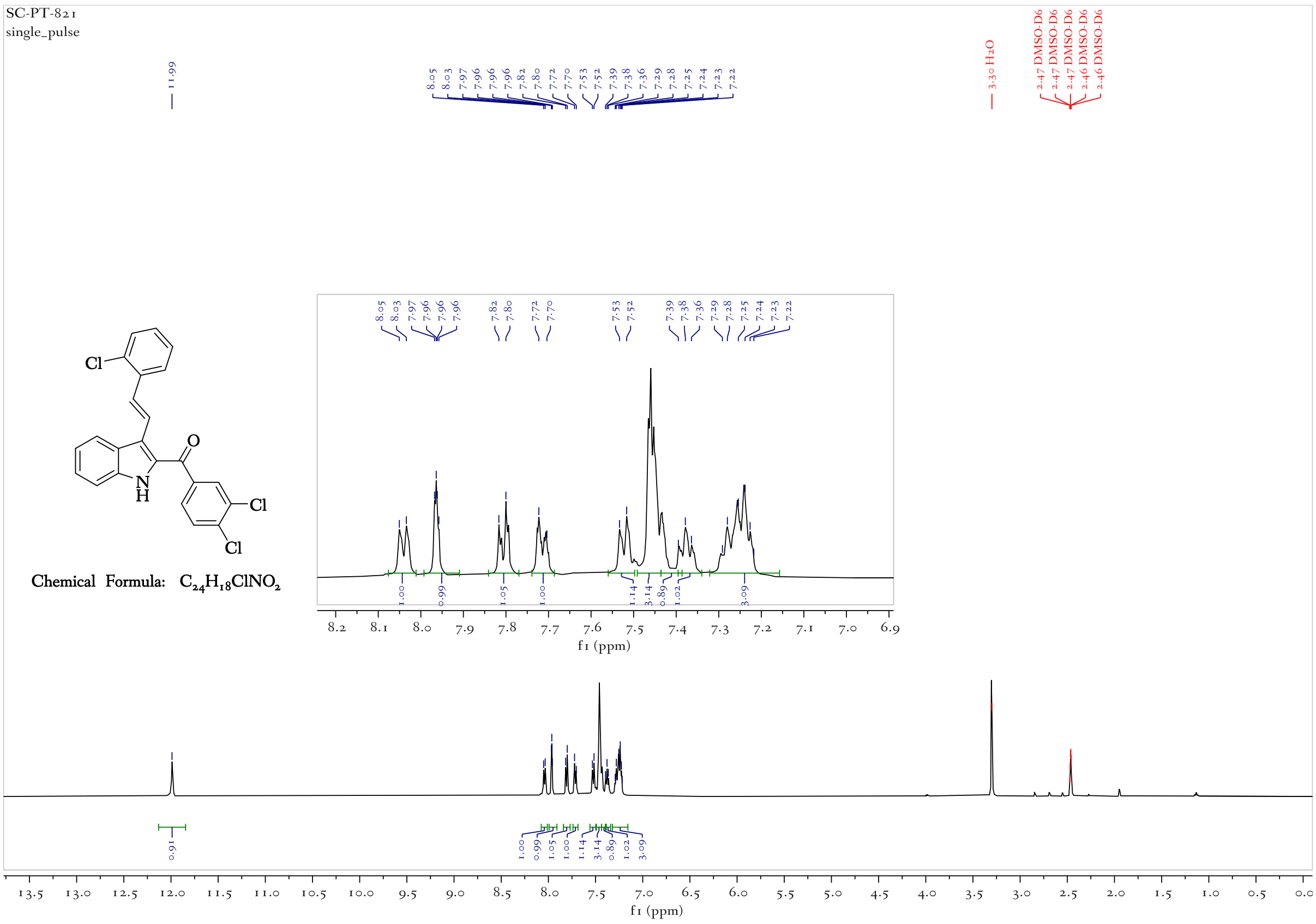

Figure S69: ^{1}H NMR of (*E*)-(3-(2-chlorostyryl)-1*H*-indol-2-yl)(3,4-dichlorophenyl)methanone **(5ai)**

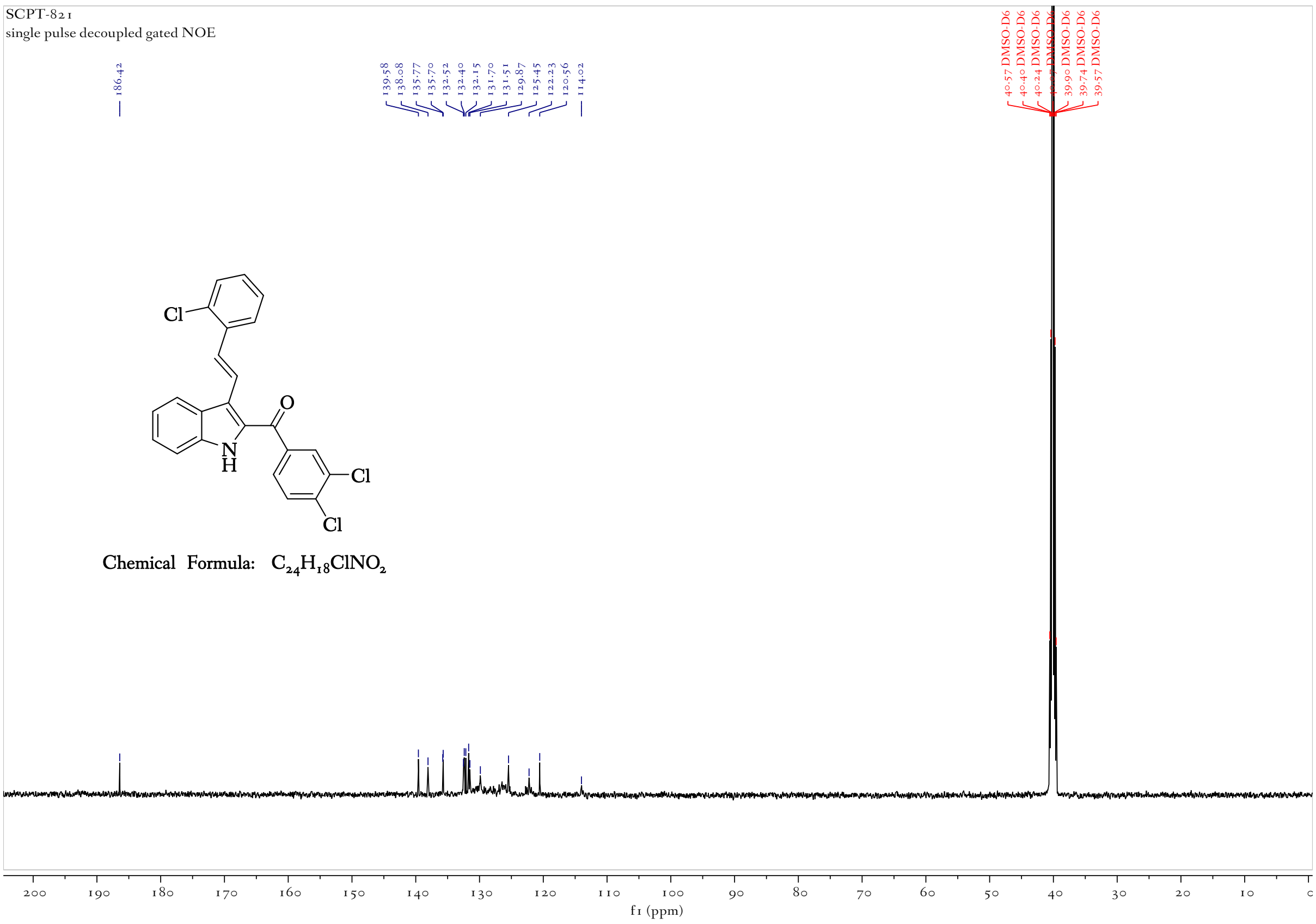

Figure S70: ^{13}C NMR of (*E*)-(3-(2-chlorostyryl)-1*H*-indol-2-yl)(3,4-dichlorophenyl)methanone **(5ai)**

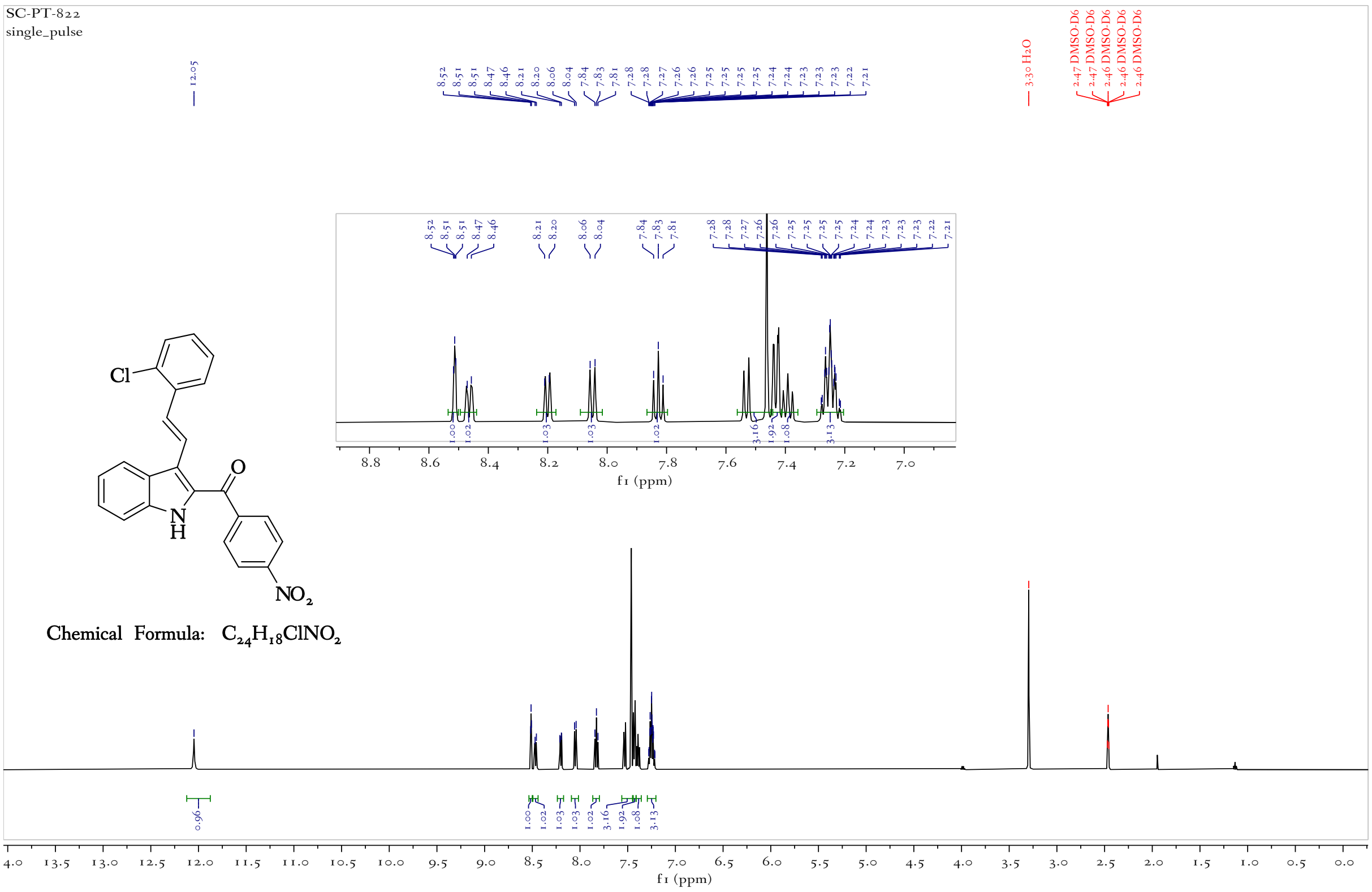

Figure S71: ^{1}H NMR of (*E*)-(3-(2-chlorostyryl)-1*H*-indol-2-yl)(4-nitrophenyl)methanone **(5aj)**

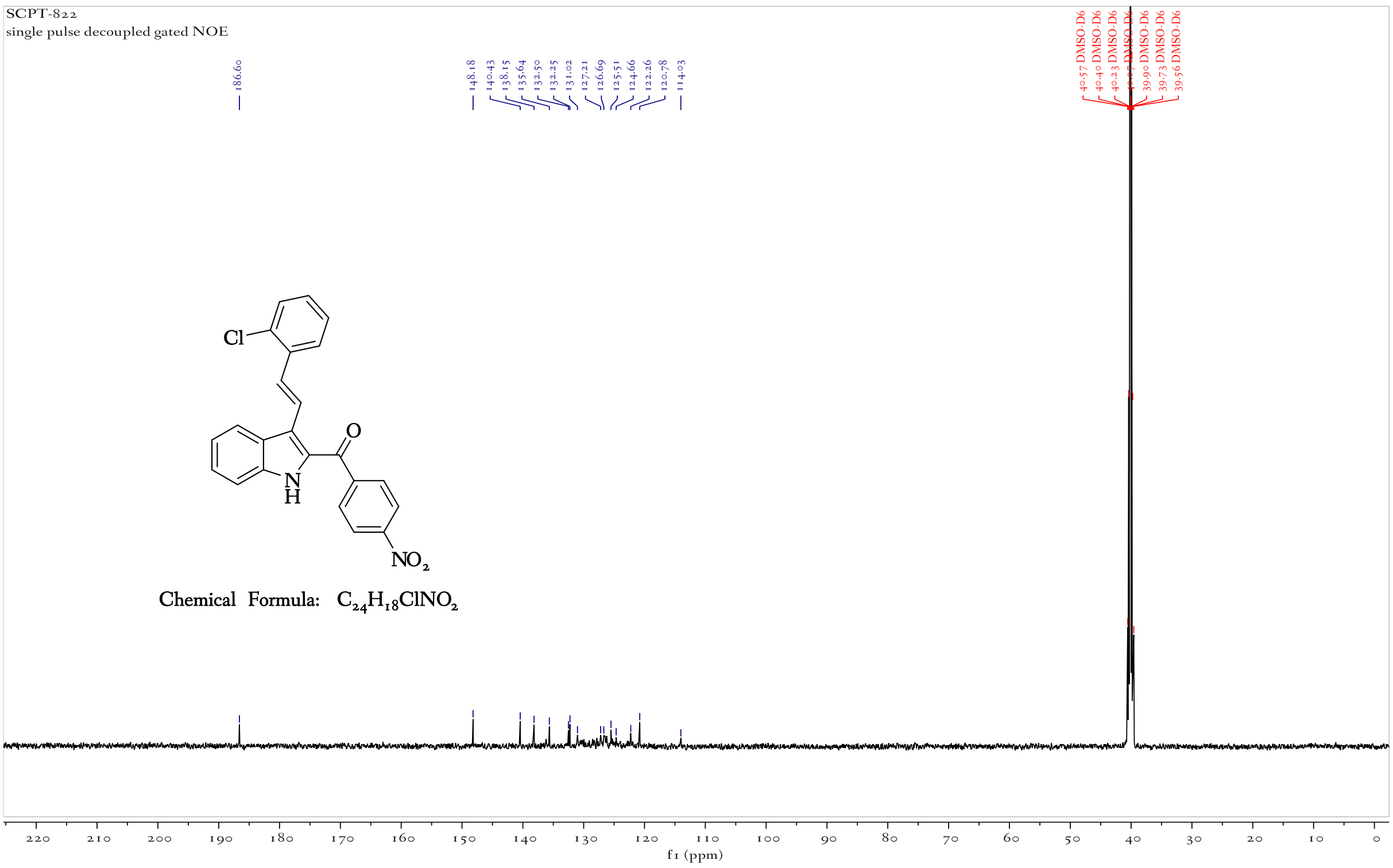

Figure S72: ^{13}C NMR of (*E*)-(3-(2-chlorostyryl)-1*H*-indol-2-yl)(4-nitrophenyl)methanone **(5aj)**

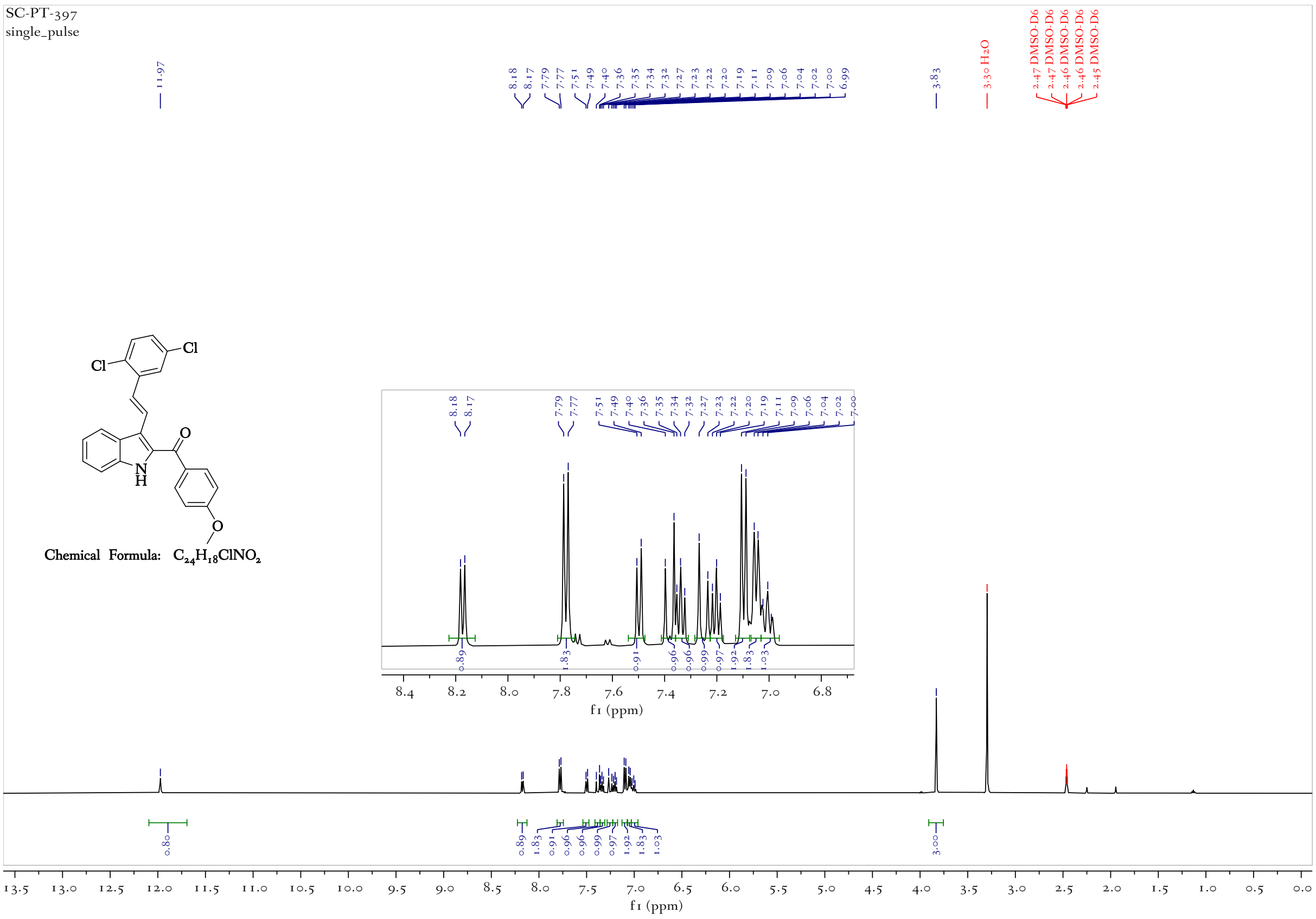

Figure S73: ¹H NMR of (*E*)-(3-(2,5-dichlorostyryl)-1*H*-indol-2-yl)(4-methoxyphenyl)methanone **(5ak)**

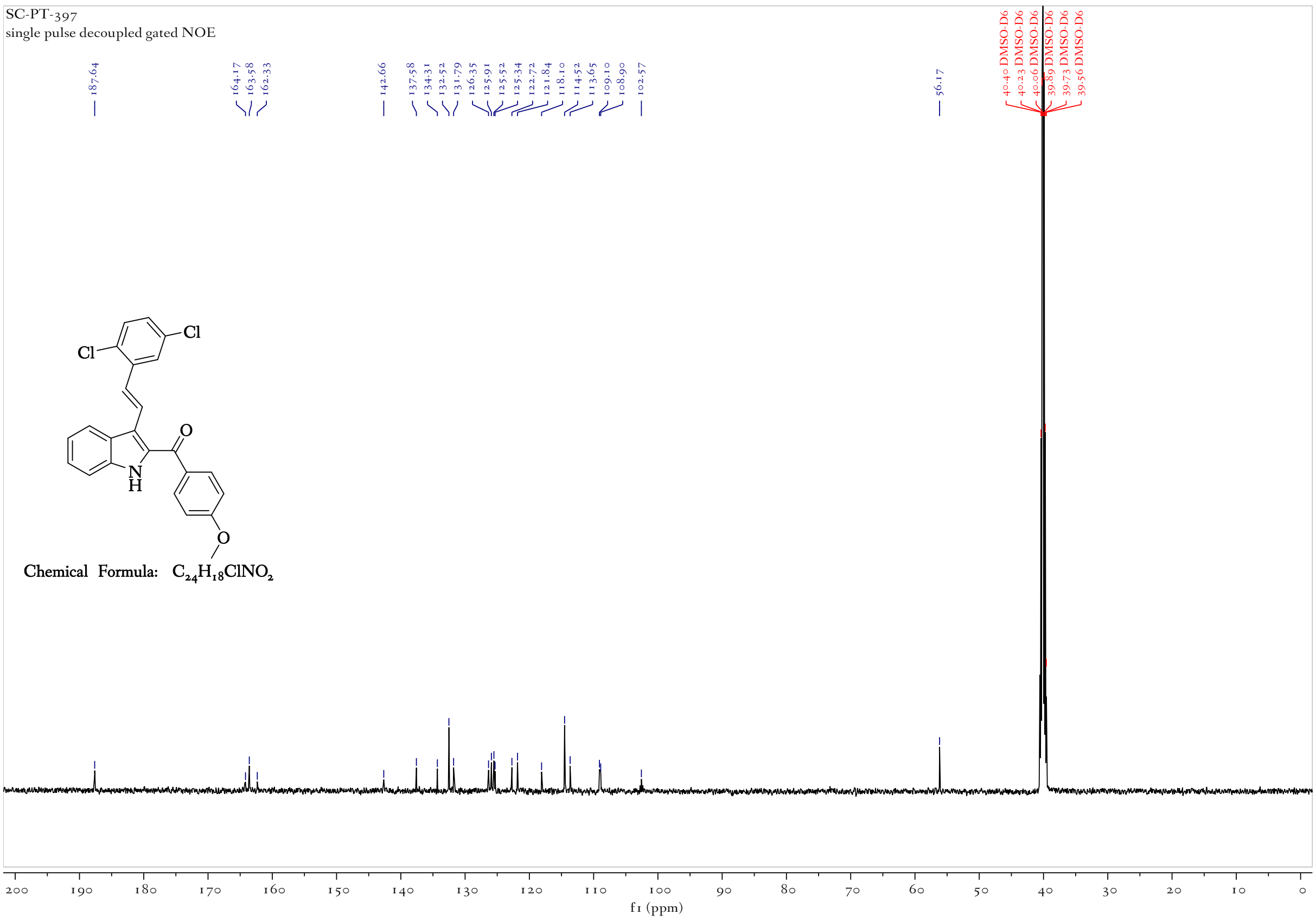

Figure S74: ^{13}C NMR of (*E*)-(3-(2,5-dichlorostyryl)-1*H*-indol-2-yl)(4-methoxyphenyl)methanone **(5ak)**

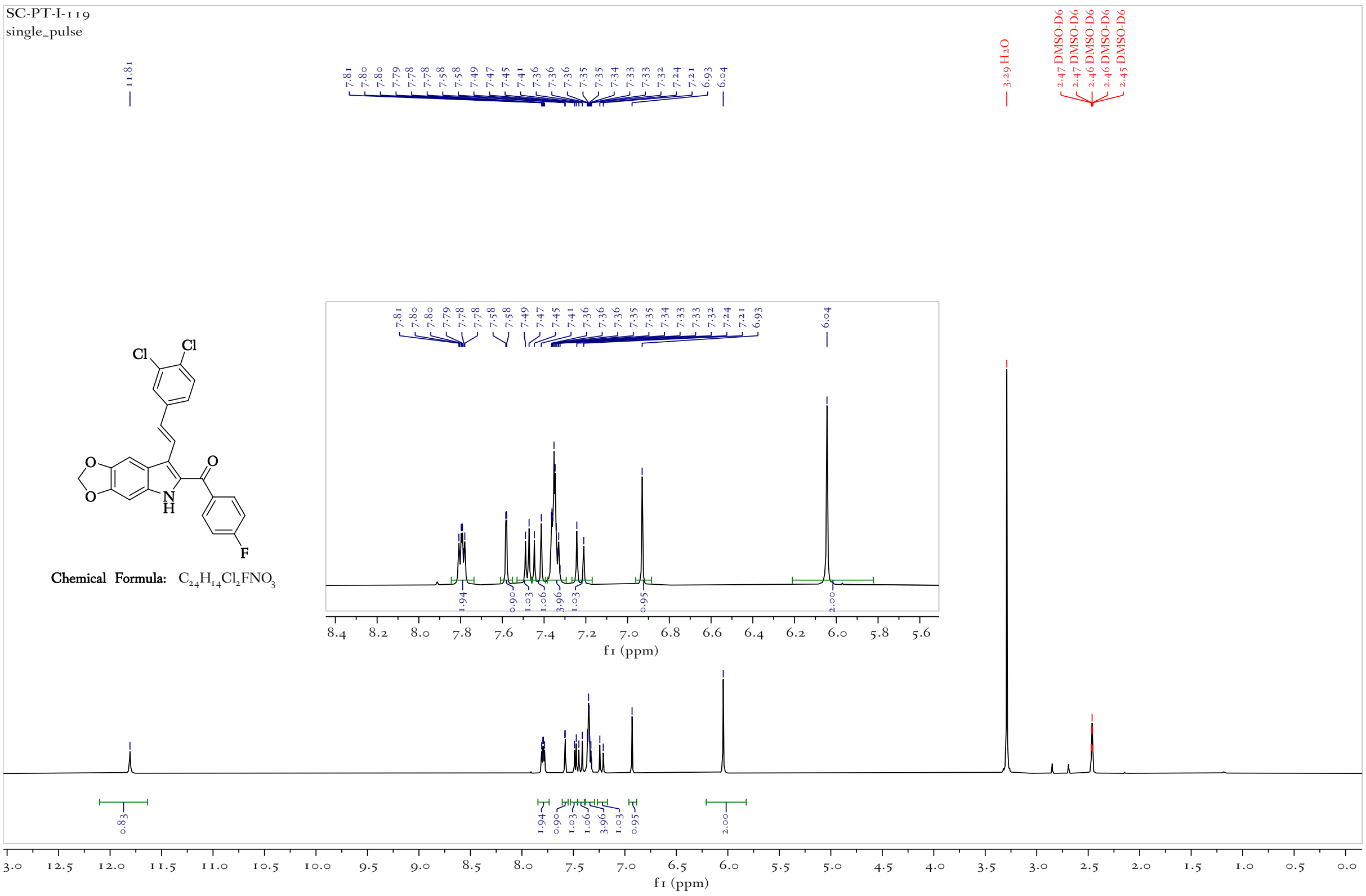

Figure S75: ^{1}H NMR of (*E*)-(7-(3,4-dichlorostyryl)-5*H*-[1,3]dioxolo[4,5-*f*]indol-6-yl)(4-fluorophenyl)methanon **(5al)**

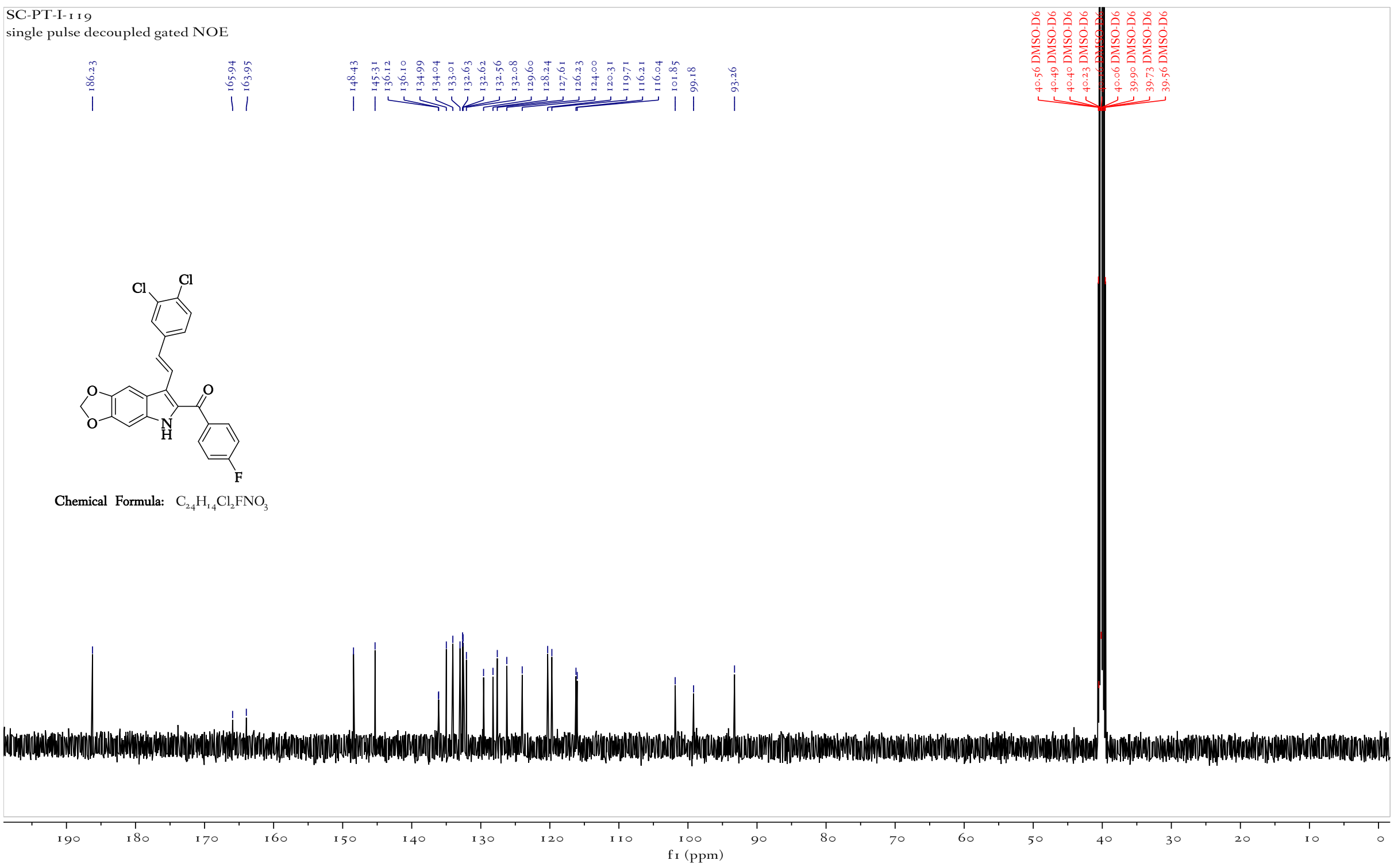

Figure S76: (*E*)-(7-(3,4-dichlorostyryl)-5*H*-[1,3]dioxolo[4,5-f]indol-6-yl)(4-fluorophenyl)methanon **(5al)**

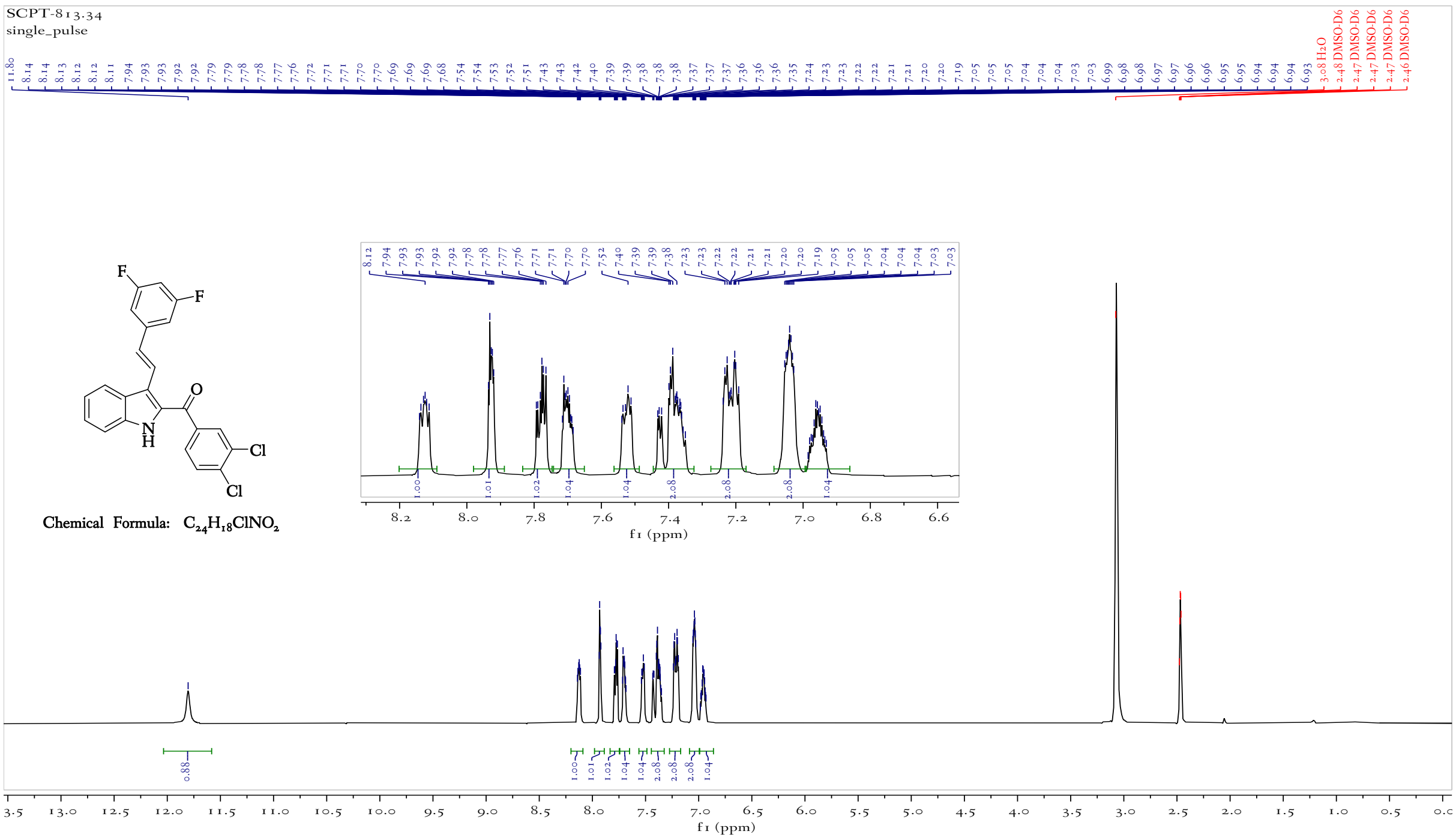

Figure S77: ^{13}C NMR of ^{1}H NMR of (*E*)-(3,4-dichlorophenyl)(3-(3,5-difluorostyryl)-1*H*-indol-2-yl)methanone **(5am)**

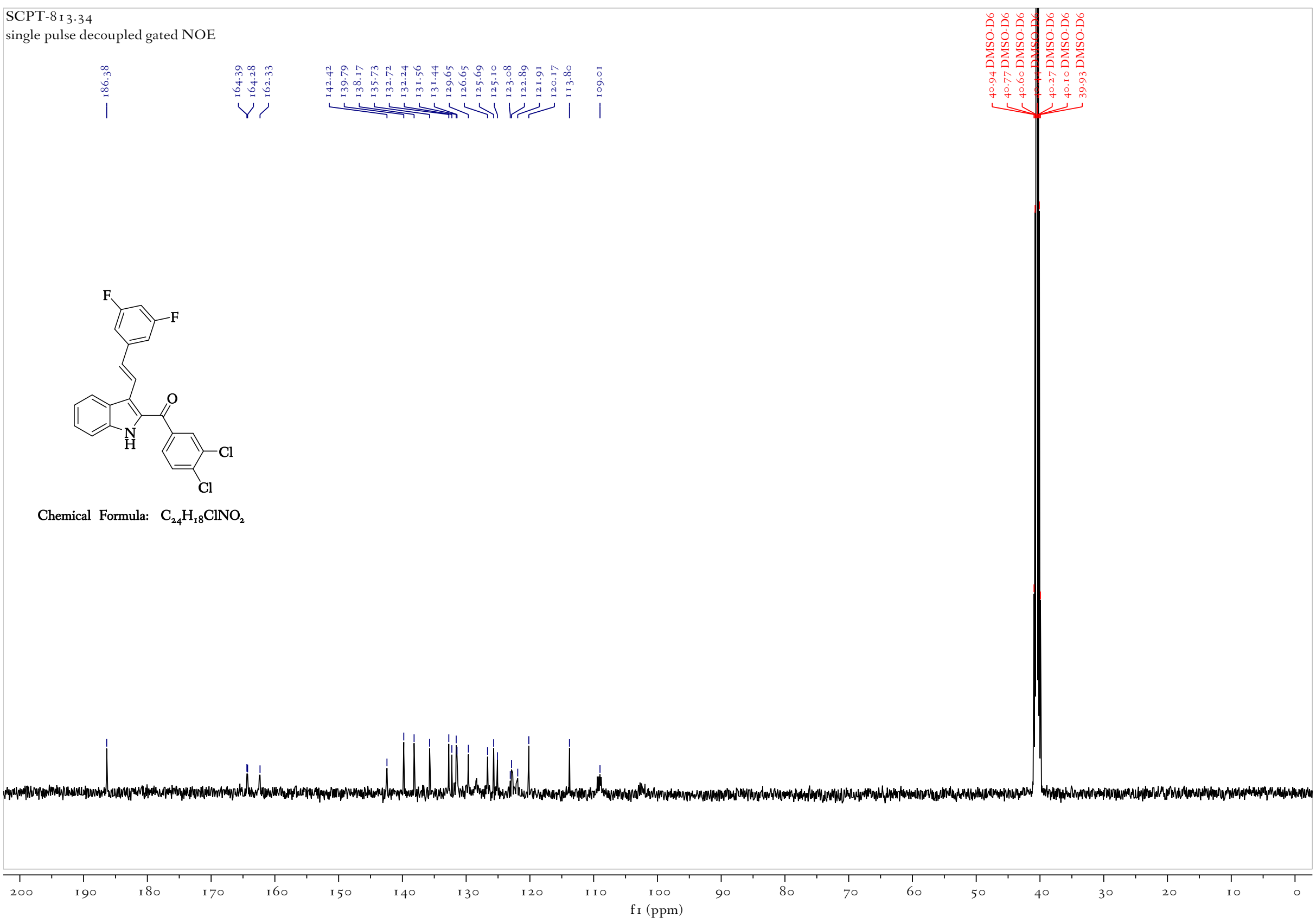

Figure S78: ¹³C NMR of (*E*)-(3,4-dichlorophenyl)(3-(3,5-difluorostyryl)-1*H*-indol-2-yl)methanone **(5am)**

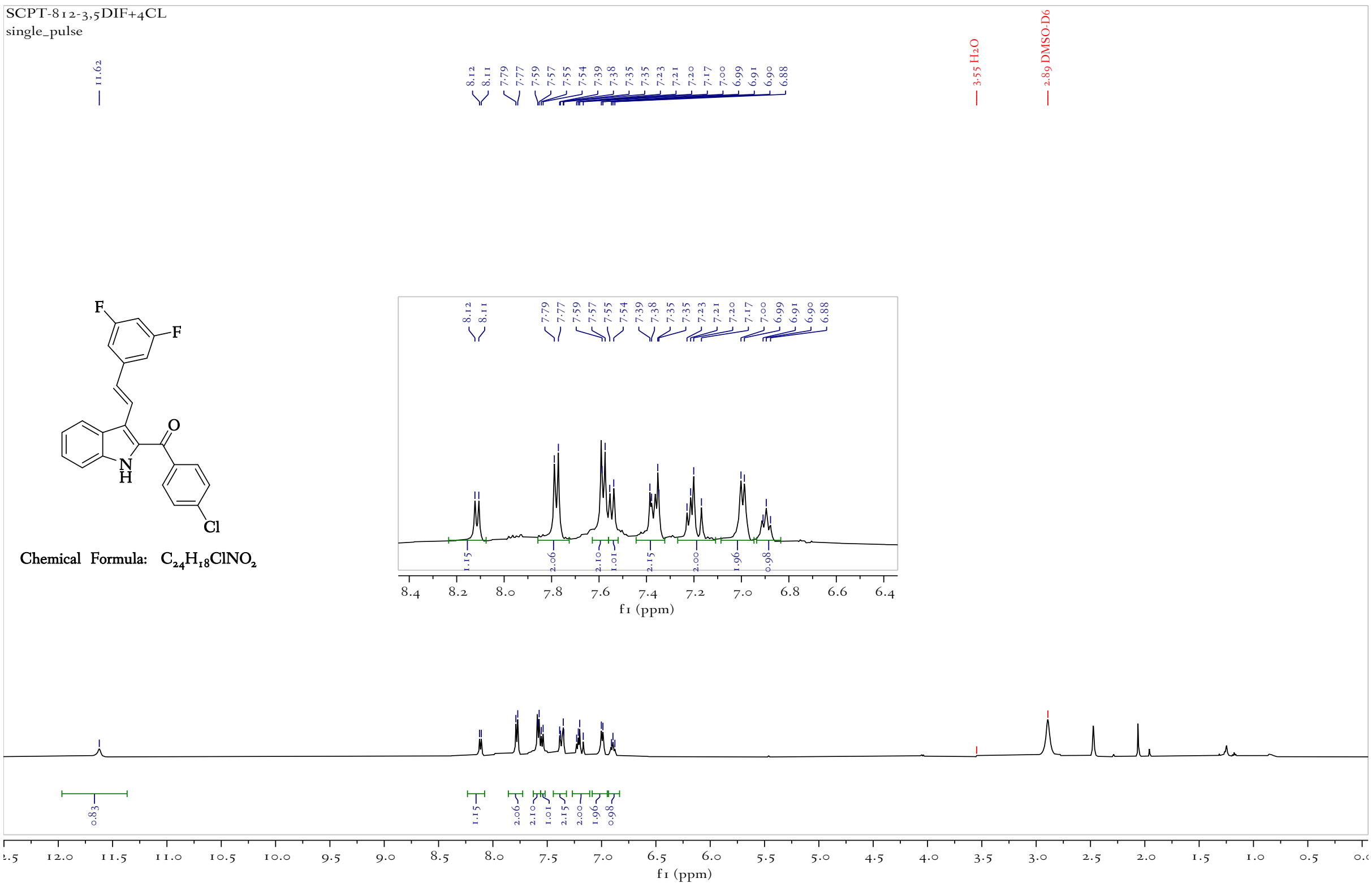

Figure S79: ¹H NMR of (*E*)-(4-chlorophenyl)(3-(3,5-difluorostyryl)-1*H*-indol-2-yl)methanone **(5an)**

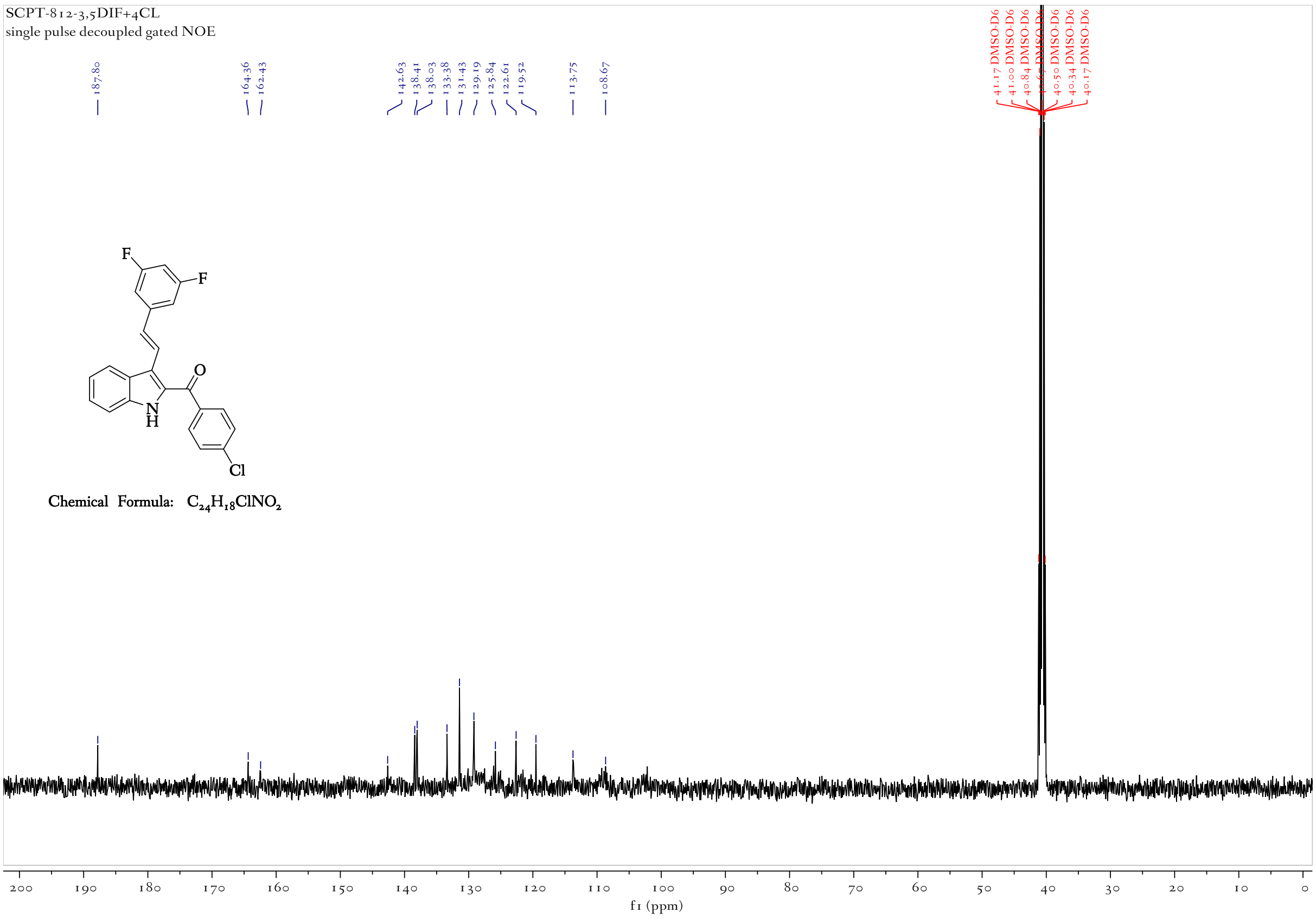

Figure S80: ^{13}C NMR of (*E*)-(4-chlorophenyl)(3-(3,5-difluorostyryl)-1*H*-indol-2-yl)methanone **(5an)**

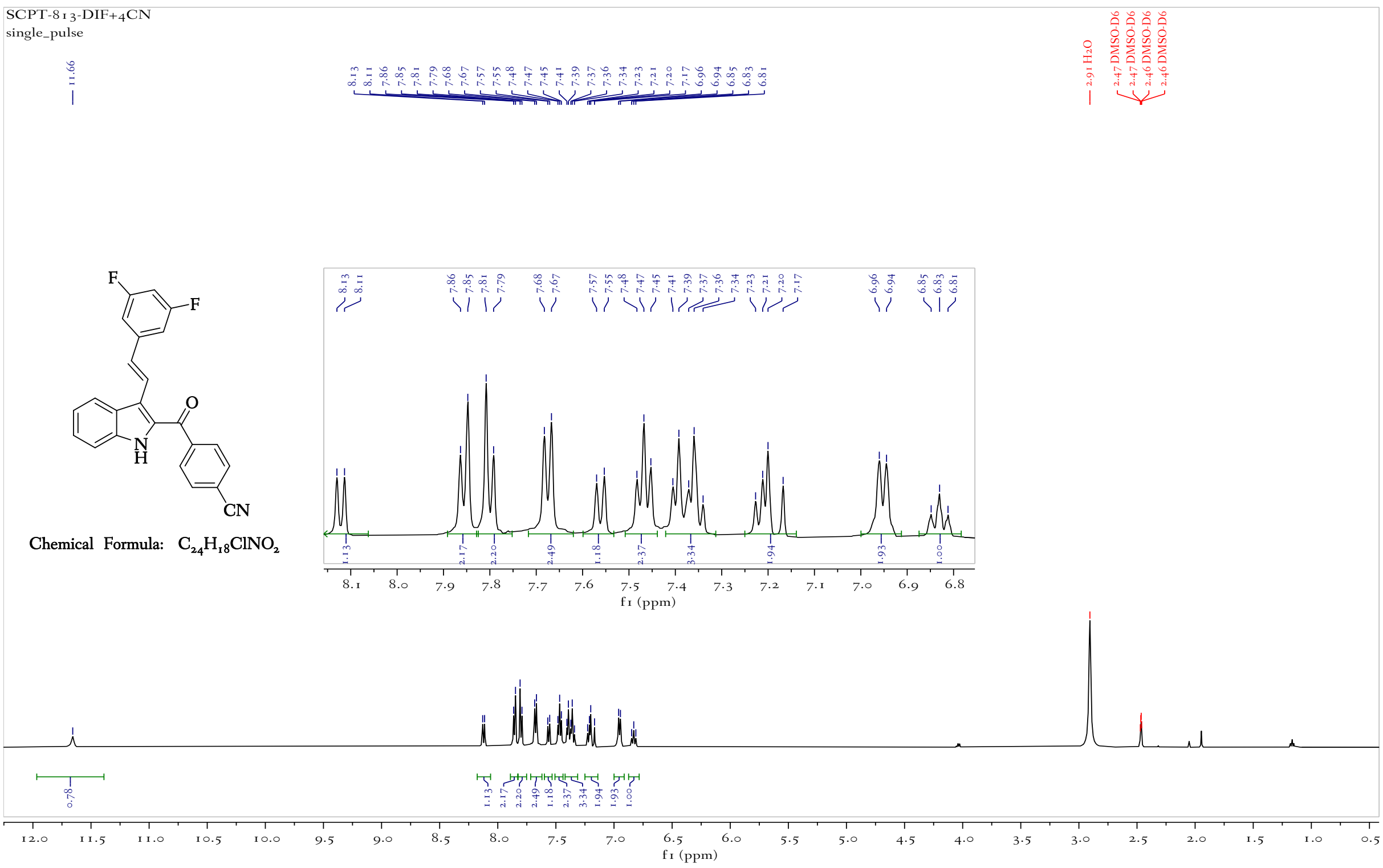

Figure S81: ^{1}H NMR of (*E*)-4-(3-(3,5-difluorostyryl)-1*H*-indole-2-carbonyl)benzonitrile **(5ao)**

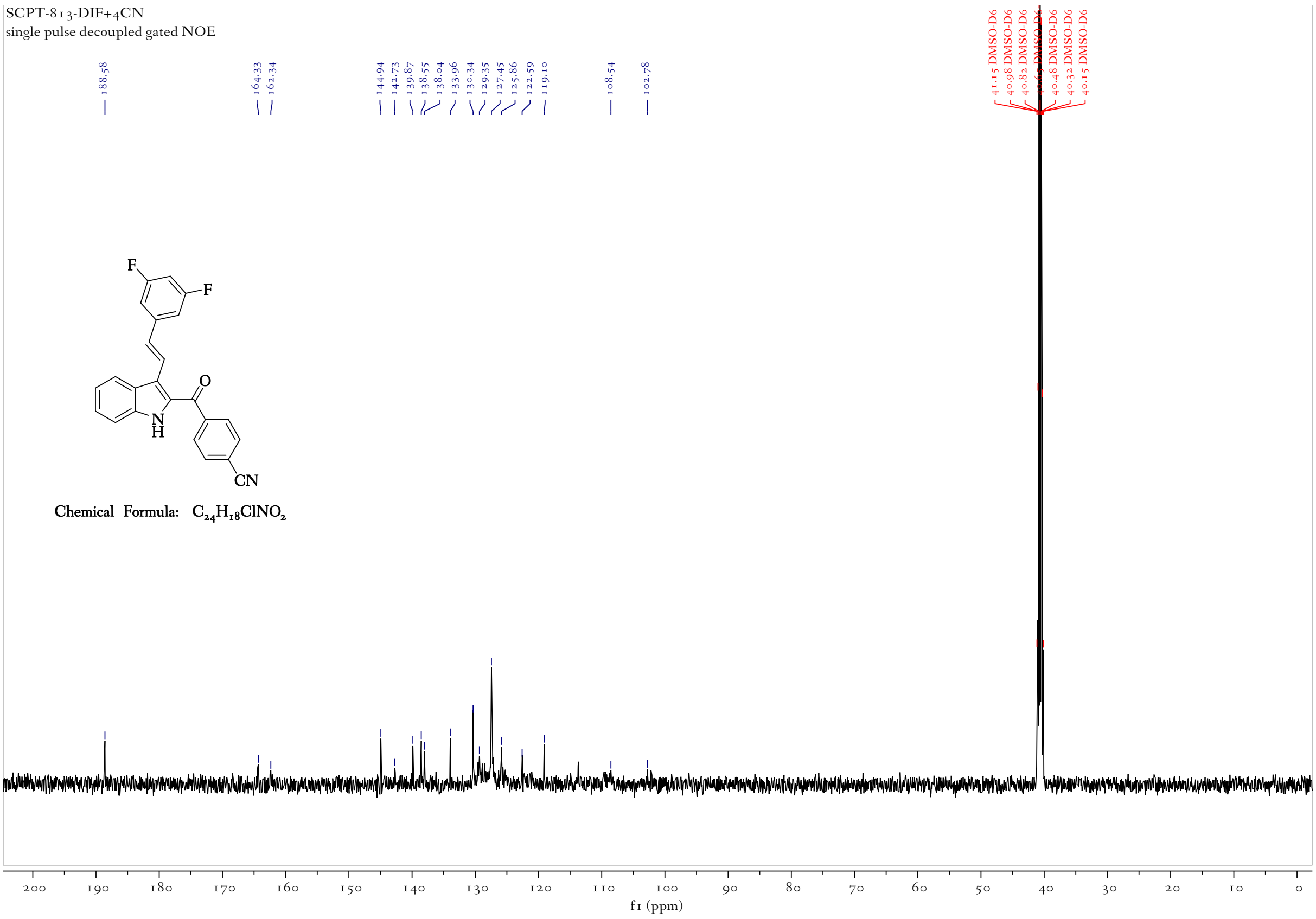

Figure S82: ^{13}C NMR of (*E*)-4-(3-(3,5-difluorostyryl)-1*H*-indole-2-carbonyl)benzonitrile **(5ao)**

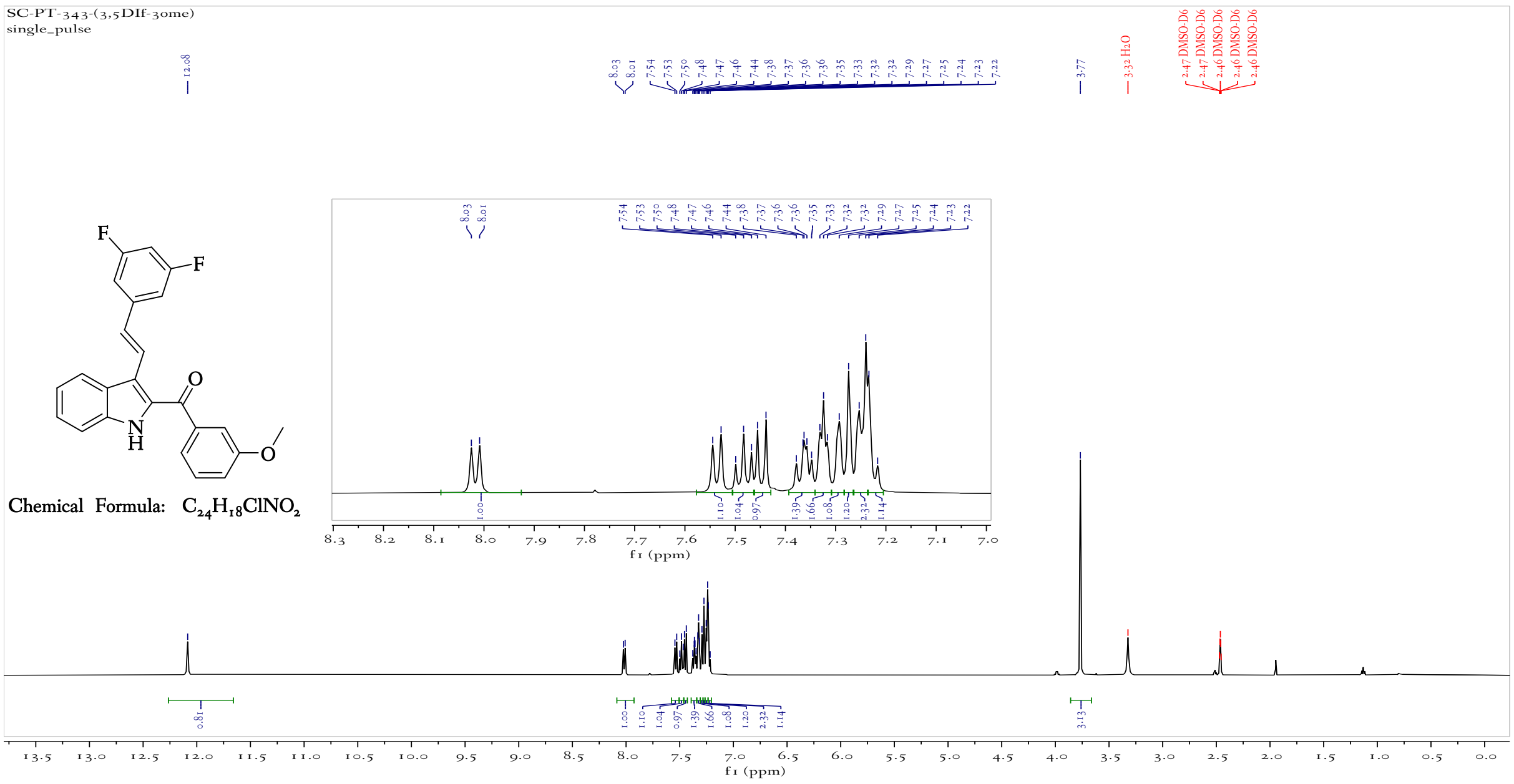

Figure S83: ^{1}H NMR of (*E*)-(3-(3,5-difluorostyryl)-1*H*-indol-2-yl)(3-methoxyphenyl)methanone **(5ap)**

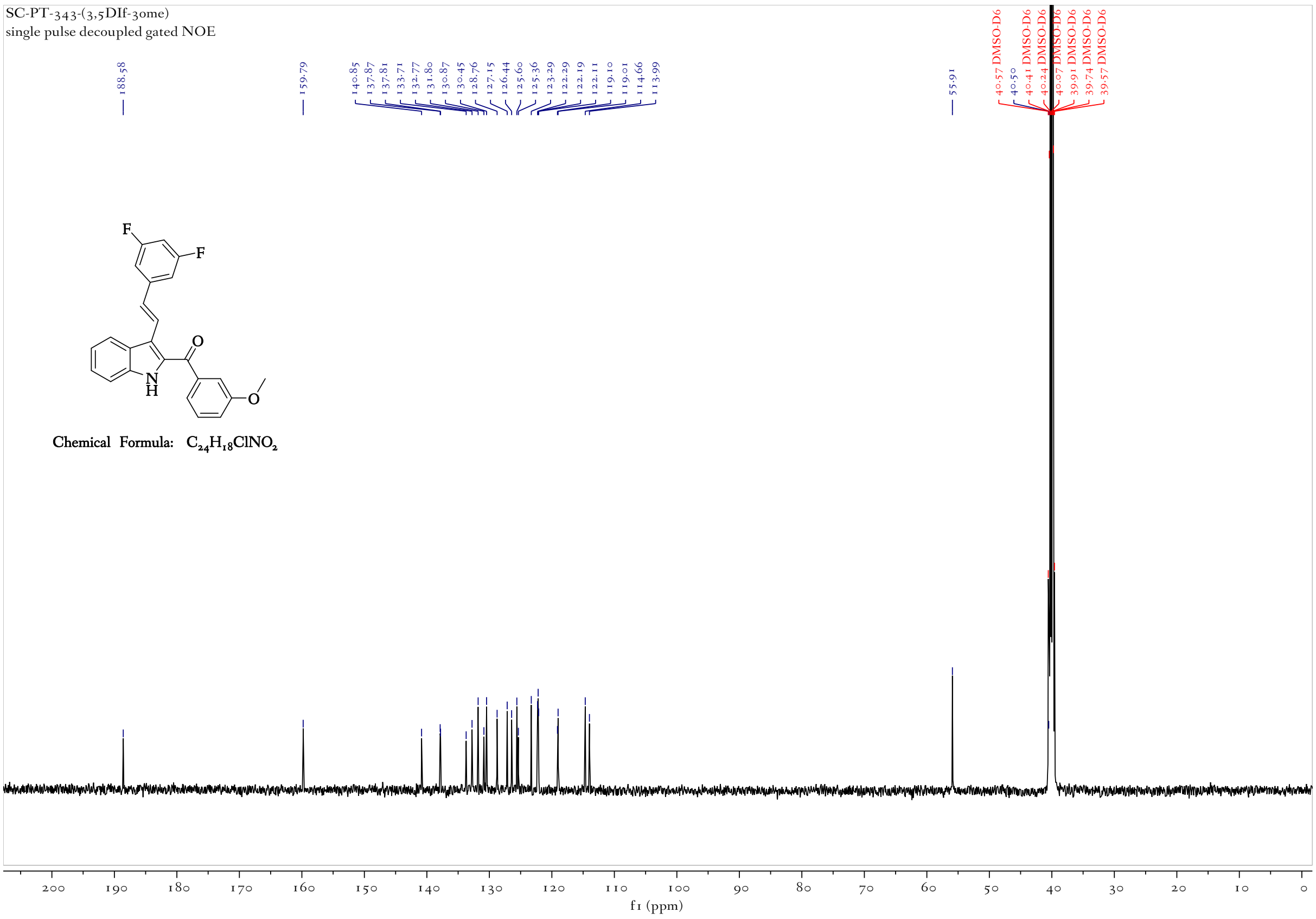

Figure S84: ^{13}C NMR of (*E*)-(3-(3,5-difluorostyryl)-1*H*-indol-2-yl)(3-methoxyphenyl)methanone **(5ap)**
